# High Throughput Sequencing of MicroRNA in Rainbow Trout Plasma, Mucus, and Surrounding Water Following Acute Stress

**DOI:** 10.3389/fphys.2020.588313

**Published:** 2021-01-13

**Authors:** Heather Ikert, Michael D. J. Lynch, Andrew C. Doxey, John P. Giesy, Mark R. Servos, Barbara A. Katzenback, Paul M. Craig

**Affiliations:** ^1^Department of Biology, University of Waterloo, Waterloo, ON, Canada; ^2^Department of Veterinary Biomedical Sciences, Toxicology Centre, University of Saskatchewan, Saskatoon, SK, Canada; ^3^Department of Environmental Science, Baylor University, Waco, TX, United States

**Keywords:** microRNA, high throughput sequencing, acute stress, non-lethal, blood plasma, mucus, water, *Oncorhynchus mykiss*

## Abstract

Circulating plasma microRNAs (miRNAs) are well established as biomarkers of several diseases in humans and have recently been used as indicators of environmental exposures in fish. However, the role of plasma miRNAs in regulating acute stress responses in fish is largely unknown. Tissue and plasma miRNAs have recently been associated with excreted miRNAs; however, external miRNAs have never been measured in fish. The objective of this study was to identify the altered plasma miRNAs in response to acute stress in rainbow trout (*Oncorhynchus mykiss*), as well as altered miRNAs in fish epidermal mucus and the surrounding ambient water. Small RNA was extracted and sequenced from plasma, mucus, and water collected from rainbow trout pre- and 1 h-post a 3-min air stressor. Following small RNA-Seq and pathway analysis, we identified differentially expressed plasma miRNAs that targeted biosynthetic, degradation, and metabolic pathways. We successfully isolated miRNA from trout mucus and the surrounding water and detected differences in miRNA expression 1-h post air stress. The expressed miRNA profiles in mucus and water were different from the altered plasma miRNA profile, which indicated that the plasma miRNA response was not associated with or immediately reflected in external samples, which was further validated through qPCR. This research expands understanding of the role of plasma miRNA in the acute stress response of fish and is the first report of successful isolation and profiling of miRNA from fish mucus or samples of ambient water. Measurements of miRNA from plasma, mucus, or water can be further studied and have potential to be applied as non-lethal indicators of acute stress in fish.

## Introduction

Fish experience acute stress when they are exposed to air. This causes a cholinergic response within seconds to minutes and an adrenergic response within minutes to hours (Wendelaar Bonga, 1997; Tort, 2013; Balasch and Tort, 2019; Cadonic et al., 2020). Molecular regulation of this stress response is well studied in fish, however, the role of miRNA in this response is a nascent field of study. MicroRNAs are short, 22-nucleotide long, non-coding RNA that decrease stability and translation of mRNA (Lee et al., 1993; Ruvkun et al., 1993; Bartel et al., 2003). MicroRNAs regulate the response of fish in a tissue-specific manner to a variety of stressors, such as hypoxia in tilapia (Qiang et al., 2020), contaminant exposure in carp (Liu et al., 2020a, b), and overcrowding in rainbow trout (Gonçalves et al., 2020). Additionally, miRNAs are measurable in blood plasma of fish and specific miRNAs are differentially expressed following exposure of rainbow trout to air (Cadonic et al., 2020). However, the entire plasma miRNA response to acute stress has not yet been characterized. It is also unknown how changes in profiles of miRNAs in blood plasma of fishes target known genes and molecular pathways of the acute stress response.

Non-lethal sampling of plasma from fish can conservatively occur up to a maximum of 1 mL per kg of body mass (Canadian Council on Animal Care, 2005; Hawkins et al., 2011). Therefore, non-lethal measurements of miRNA in blood plasma of large fishes can be made. However, when measuring miRNA from smaller or endangered species, an alternative method would be more appropriate. Epidermal mucus of fishes has been used as a sampling location for indicators of acute stress. These include cortisol, stress-related proteins, enzymes, glucose, and lactate levels (Easy and Ross, 2010; Guardiola et al., 2016; Fernández-Alacid et al., 2019). Furthermore, miRNAs are known to be excreted from tissue and fluids in exosomes (Valadi et al., 2007; Skog et al., 2008; Zernecke et al., 2009; Kosaka et al., 2010; Zhang et al., 2010), associated with proteins (Wang et al., 2010; Arroyo et al., 2011; Turchinovich et al., 2011; Vickers et al., 2011), or by passively leaking from a site of injury (Chen et al., 2008; Mitchell et al., 2008). Therefore, in order to provide a representation of the stress status of smaller fishes, mucus is a potential matrix for non-lethal sampling of miRNA. However, miRNAs have never been measured in mucus of fishes and if present, it is unknown whether patterns of relative concentrations of miRNAs change following acute stress, as it does in blood plasma.

Collection of mucus requires fish to be handled and animal guidelines recommend minimizing the removal of epidermal mucus (Canadian Council on Animal Care, 2005). Therefore, to measure changes in miRNA both non-lethally and non-invasively, one of the study objectives was to determine if miRNA can also be measured from ambient water samples. Nucleic acids, in the form of environmental DNA (eDNA), can be measured from water samples and that DNA originates from epithelia, blood, urine, feces, and gametes of fishes, which are all locations that contain miRNA (Höss et al., 1992; Valiere and Taberlet, 2000; Ficetola et al., 2008; Jerde et al., 2011). In humans, profiles of relative concentrations of miRNA in tissues are reflected in excreted fluids (Cui and Cui, 2020; Park et al., 2020). Therefore, profiles of miRNA present in water external to fishes might be related to miRNA profiles in blood. If this supposition is correct, there might be no need to capture fish or collect samples of blood or mucus to assess status and trends in the physiological state of stress. A concern when measuring RNA in the environment is its instability, as mRNA have an average half-life of 5 min (Moran et al., 2013). However, miRNAs have a half-life of 5 days and are protected by exosomes or associated proteins (Gantier et al., 2011). Therefore, there is potential for miRNA to be measured in water and their abundance to be altered following stress, however, this has not yet been measured.

The objective of this study was to determine how miRNA profiles of rainbow trout are altered in non-lethal samples in response to acute stress. First, it was hypothesized that the pattern of relative concentrations of miRNAs in plasma of rainbow trout would be altered following acute stress and this response would be linked to known molecular responses to stress. Second, it was hypothesized that rainbow trout miRNAs would be present and measurable in epidermal mucus and water and miRNA abundances would be altered following acute stress. Third, it was hypothesized that there would be differentially expressed miRNAs in common in plasma, mucus, and water, which would provide evidence that external profiles of miRNA are representative of internal changes in miRNA. To test these hypotheses, samples of blood plasma, epidermal mucus, and water were collected from rainbow trout (*Oncorhynchus mykiss*) prior to and following exposure to 3 min of air. RNA was extracted and differentially expressed miRNAs identified in all three matrices, by use of high throughput sequencing, followed by pathway analysis of altered plasma miRNA targets, and qPCR validation. MicroRNA was further developed as a non-lethal measure of stress in fishes and the foundation was laid for further development of mucus and water miRNA as non-lethal measures of fish stress.

## Materials and Methods

### Animals and Experimental Design

Rainbow trout (*Oncorhynchus mykiss*) were procured from Silver Creek Aquaculture (Erin, ON, Canada) or donated by the Ontario Ministry of Natural Resources and Forestry. All experimental procedures were approved and conducted per the University of Waterloo and the Canadian Council of Animal Care guidelines (Animal Utilization Project Protocol #40315). Fish were housed in the University of Waterloo Aquatic Facility (Waterloo, ON, Canada), with a 12 h:12 h light-dark cycle, and fed daily with EWOS Vita (Floating Complete Fish Food for Salmonids) to satiety. Water was maintained at 13.5 ± 0.7°C, pH 8.85, ∼2000 μS, in well-aerated water. Fish were maintained at a density of four fish per 200 L tank. Experimental fish had a mean length of 30.5 ± 3.9 cm and a mass of 322 ± 138 g. Water flow was maintained at 3.6 ± 0.1 L/min. Flow through loading of the tanks was 0.350 ± 0.057 kg/L/min.

**FIGURE 1:**
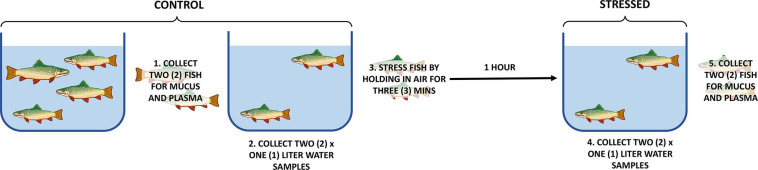
Experimental design. This experiment was performed in triplicate. Plasma and mucus from one pre-stress fish and one post-stress fish were sequenced from each experimental replicate. One pre-stress water sample and one post-stress water sample were sequenced from each experimental replicate. The experimental order of sample collection was organized so that the same density of fish was present in the tank when the water collections occurred.

Control samples of plasma and epidermal mucus were collected from two rainbow trout and then water samples were collected from a tank containing two remaining fish (control samples). The remaining two rainbow trout were held out of water for 3 min. One hour following the 3-min in air, water was collected from the tank containing the stressed fish (1 h post-stress water sample). Immediately following collection of water, blood plasma and epidermal mucus were obtained (1 h post-stress mucus and blood samples). To ensure that both the adrenergic and cholinergic stress responses had occurred or were occurring, samples were collected 1-h post stress. Also, due to the water flow maintained in the tanks, an entire tank turnover was completed after 1 h. Three experimental replicates of the air stress experiment were performed (Figure 1).

For collection of blood and mucus, fish were euthanized in a buffered MS-222 solution (0.5 g/L MS-222 in 1 g/L NaHCO_3_). Mucus was collected by scraping epidermal mucus from the left side of rainbow trout, being cautious not to introduce any foreign DNA or RNA. This was accomplished by using a new pair of gloves cleaned with 70% ethanol, using one hand to secure the trout by holding by the mouth and gill operculum, and using the other hand to scrape the mucus into a 2 mL cryotube. Mucus was placed on dry ice and stored at −80°C until analysis. Blood was collected by caudal puncture using a 23 G needle fitted onto a 1 mL syringe and pre-coated with 0.5 M EDTA. One mL of blood was added to a 2 mL microcentrifuge tube containing 10 μL of 0.5 M EDTA to prevent coagulation. Blood was stored on ice until centrifugation at 1,300 × *g* for 10 min at 4°C to collect plasma. Plasma was aliquoted into 200 μL and stored at −80°C until RNA extraction. Only samples with no visible lysis were used for RNA extraction and sequencing.

Water was collected in 1 L amber bottles sterilized with 10% bleach for 20 min, rinsed three times with ultrapure water, and autoclaved. Care was taken to collect water from the tank without introducing DNA or RNA. For example, the net used to capture and move fish during the 3 min air stress was placed into the water before control water samples were collected so that there would be no new nucleic acids introduced from the net. Samples of water were stored at 4°C until filtered within 3 h of collection. Samples were filtered onto 0.45 μm cellulose nitrate filters (Thermo Scientific^TM^ 1450045) in a sterilized laminar flow hood using a peristaltic pump. Filters were placed in 5 mL tubes on dry ice and stored at −80°C.

### Stress Assessment

To validate that rainbow trout experienced a physiological response to the acute stress treatment, cortisol, lactate, and glucose in plasma were measured. To measure plasma cortisol, plasma samples were thawed on ice, diluted by a factor of ten, and cortisol was quantified using the Cortisol Saliva ELISA kit (TECO, Switzerland). To measure plasma lactate, plasma was thawed on ice, samples and standards were deproteinized by adding 200 μL ice cold 6% perchloric acid, incubating on ice for 5 min, and centrifuging for 2 min at 18,000 × *g* at 4°C. The supernatant was brought to a pH between 6.5 and 8.0 using potassium hydroxide and centrifuged at 18,000 × *g* for 15 min at 4°C. The supernatant was added to the 96 well plate in triplicate and the reaction cocktail (320 mM glycine, 320 mM hydrazine monohydrate, 2.4 mM NAD+, 2 U/mL LDH) was added to a 96 well plate. A kinetic assay at 340 nm was run and lactate quantified (Bergmeyer, 1974). To measure plasma glucose, plasma was brought to room temperature, 4 μL was placed on a cleaned glass slide and an Accu-Chek Aviva Nano glucose monitor was used per the manufacturer’s directions. Duplicate measurements were conducted on each sample. Data were tested for normality and equal variance. One-tailed *t*-tests were conducted on this data comparing control and stressed plasma samples. These data were tested with 95% confidence (*n* = 3).

### MicroRNA Extraction and Sequencing

MicroRNAs were extracted from blood plasma by use of the Norgen Plasma/Serum RNA Purification Mini Kit (CAT 55000). Directions from the manufacturer were followed with a few modifications. Aliquots of plasma were thawed on ice and centrifuged for 2 min at 400 × *g* and 200 μL of cleared supernatant was extracted. During elution, 25 μL of Solution A was applied to the column and the eluate was transferred to the column again for maximum recovery of RNA. Following this, the extracted RNA was further processed using the Norgen RNA Clean-Up and Concentration Micro-Elute Kit (CAT 61000). With this kit, 75 μL RNase free water was added to the 25 μL of extracted RNA to bring the starting volume to 100 μL. Samples were vortexed for 10 s and spun at 14,000 × *g*, where no specified directions were given. Two separate elutions were performed using 15 μL of eluate. This ensured maximum recovery. RNA quantification occurred using the SoftMax SpectraDrop microplate.

MicroRNAs in mucus were extracted using the Qiagen miRNeasy Serum/Plasma Advanced Kit (CAT 217204). The manufacturer’s directions were followed and 200 μL of mucus was used for the extraction. For any non-specified centrifuge speed, 12,000 × *g* was used. RNA quantification was performed by Norgen Biotek using the RiboGreen^TM^ RNA Assay on a fluorescent microplate reader.

Environmental miRNAs were extracted from water by use of the Norgen Water RNA/DNA Purification Kit (CAT 26480). The manufacturer’s directions were followed with the following exceptions. Beads were transferred to the 5 mL tube where filters had been stored, and 1 mL lysis buffer E was added. Tubes were vortexed for 5 min on a flatbed vortex mixer at maximum speed. The initial 1 min spin at 20,000 × *g* was replaced with a 7 min spin at 3000 × *g* (maximum allowable speed for 5 mL tubes in centrifuge). Due to this modification, visual separation of lysate and filter was ensured before removing the supernatant for use in the remainder of the protocol. The optional DNase I treatment was applied (Norgen RNase-Free DNase I Kit – CAT 25710) to remove potential DNA contamination prior to sequencing. An optional addition of 50 μL elution solution to maximize yield was performed. RNA quantification was performed by Norgen Biotek using the RiboGreen^TM^ RNA Assay on a fluorescent microplate reader.

Library prep and sequencing were performed by Norgen Biotek (Thorold, ON, Canada) using the Norgen Biotek Small RNA Library Prep Kit (CAT 63600). Samples (*n* = 3 for plasma, mucus, and water at both control and 1 h post-stress = total 18 samples) were sequenced using the Illumina NextSeq500 with a minimum 10 million read depth. The NextSeq 500/550 High Output Kit v2 (51 cycles using a 75-Cycle Kit) (Illumina, CAT FC-404-2005) was used as the sequencing reagent.

### Bioinformatic Analysis

Bioinformatic analysis was accomplished using the miARma-Seq analysis workflow (version 1.7.2; Andrés-León et al., 2016; Andrés-León and Rojas, 2019). However, adapter removal, quality filtering, and differential expression analysis were performed independently of the miARma-Seq tool. Adapter removal and quality filtering, performed by cutadapt (version 2.10; used under python3.8), was conducted separately from the miARma-Seq tool due to the incompatibility of the version of cutadapt present in the miARma-Seq pipeline with the two-color chemistry of the Illumina NextSeq (Martin, 2011). The differential expression analysis was performed independently from the miARma-Seq tool because two methods of differential expression analysis, DESeq2 and edgeR, were used. edgeR is included within the miARma-Seq tool but not DESeq2, which was therefore performed independently. Similarly, additional analysis options were needed in edgeR than those included within the miARma-Seq tool, so this analysis was also performed separately. The overall bioinformatic analysis followed the miARma-Seq tool workflow but only sequence quality checks, alignment, annotation, and read counting were performed using the miARma-Seq tool directly.

#### Quality Control of Raw Reads

In order to ensure that there were no issues with the raw fastq sequencing reads, quality control was performed using FastQC (version 0.11.9, Andrews, 2019). Metrics that were required to pass in this step for the reads to be included in downstream analysis were “per base sequence quality,” “per tile sequence quality,” “per sequence quality scores,” “per base N content,” and “sequence length distribution.” FastQC metrics that were expected to fail for raw reads or for RNA-Seq were “per base sequence content,” “per sequence GC content,” “sequence duplication levels,” “overrepresented sequences,” and “adapter content.” Highly abundant sequences in RNA-Seq caused the percentage of each base to deviate from the expected 25% frequency (Sheng et al., 2017). The per sequence GC content was narrower due to overexpression of certain sequences, a characteristic of RNA-Seq. More than one peak in GC content was observed as there are different types, and therefore lengths, of small RNA (Sheng et al., 2017). The miARma-Seq tool only annotates miRNAs therefore other small RNA were excluded from our analysis (Sheng et al., 2017). The level of duplication of sequences and overrepresented sequences metric failed as expected due to the large sequence overrepresentation and few unique sequences present in RNA-Seq reads. Refer to the Supplementary Methods for the configuration file used to run this analysis (precutadapt_fastqc_configfile.ini).

#### Adapter Removal and Quality Filtering of Raw Reads

Adapter removal and filtering was performed using cutadapt (version 2.10; used under python3.8; Martin, 2011). The adapter was removed and any bases less than a quality score of 28 (high quality) were trimmed (refer to may7_cutadapt2.10adapter_removal_and_trimming.sh in the Supplementary Methods). Next, sequencing files were further filtered to remove any sequences shorter than 18 bases or longer than 35 bases, as they are not considered useful in miRNA analysis (Andrés-León and Rojas, 2019; refer to may7_cutadapt2.10sizetrim.sh in the Supplementary Methods).

#### Quality Control of Trimmed Reads

FastQC analysis was performed again on the trimmed reads to ensure that only sequences with quality scores greater than 28, with lengths between 18 and 35 bases, and with no adapters were present. The configuration file is available in the Supplementary Methods (postcutadapt_fastqc_configfile.ini).

#### Alignment, Annotation, and Read Counting

The *de novo* approach of the miARma-Seq pipeline was used since no rainbow trout miRNAs are present in the mirbase.org database. Reads were aligned to the rainbow trout genome, annotated using *Salmo salar* miRNA (closest salmonid relative in mirbase.org), due to the conservation in miRNA sequence identity between fish species (Salem et al., 2010), and counted. Viral miRNAs were excluded from differential expression analysis though they can share a seed sequence with eukaryotic miRNAs, as there is not full sequence similarity (Kincaid et al., 2012; Guo and Steitz, 2014; Mishra et al., 2020). Therefore, they would not be annotated and counted. Alignment was performed within miARma-Seq using bowtie1 (version 1.1.1) and annotation and read counting using mirDeep2 (2.0.1.2) (Langmead et al., 2009; Friedländer et al., 2012). Workflow and parameters are outlined in the miARma-Seq configuration file (Supplementary Methods – miARmaseq_denovo_ssa_configfile.ini).

For alignment and annotation, a bowtie1 index was created from the rainbow trout genome (GCF_002163495.1_Omyk_1.0_genomic.fna; downloaded April 9, 2019). The bowtie index is available at https://doi.org/10.6084/m9.figshare.12459905.v2 (Ikert, 2020a). Additional bowtie parameters were added to allow for only one mismatch (default is two) and to only report the best alignment, according to recommendations by Tam et al. (2015). The miARma-Seq script defaults were used for all remaining miRDeep2 tools (for description of arguments)^1^. Also, it is crucial to note that miRDeep2 will count reads that originate from different precursors twice. miARma-Seq corrects for this by averaging the number of counts of mature miRNA from different precursors so that all the tags (miRNAs) are unique in the read count file. This allows for more accurate differential expression analysis. Quality control of the alignment, annotation, and read counting was performed by referring to the stats and log files, ensuring that no errors were present, and that the percentage of reads aligned versus unaligned were reasonable.

#### Differential Expression of MicroRNA

In order to identify miRNAs that were differentially expressed, the read count file from miARma-Seq was analyzed by using both edgeR and DESeq2 (Robinson and Smyth, 2007, 2008; Robinson and Oshlack, 2010; Robinson et al., 2010; McCarthy et al., 2012; Chen et al., 2014; Love et al., 2014; Zhou et al., 2014; Lun et al., 2016). These are tools recommended and validated for differential expression analysis for studies with a sample size of three and are able to detect effect sizes of a log-fold change greater than two (2.0) (Schurch et al., 2016). Since they both have well-developed models for analyzing RNA-Seq files and to avoid missing true positives, which is a concern in multiple comparison testing, both tools were used to create a list of differentially expressed miRNA for each matrix (Yendrek et al., 2012). It was expected that each tool would produce unique results because each conduct their analysis differently. edgeR normalizes sequencing reads by using the TMM (trimmed mean of *m*-values) method and filters using a nominal, user-defined CPM (counts-per-million) (Robinson and Oshlack, 2010). DESeq2 normalizes sequencing reads using a median of ratios method and filters by determining the optimal CPM threshold from several tested threshold values (Anders and Huber, 2010; Love et al., 2014). Scripts used for each of these tools and versions of packages for each are found in the Supplementary Methods (script_deseq2.R and script_edgeR.R).

For edgeR analysis, the read count file was split by sample type so that library normalization would not be affected by types of matrices. Samples were normalized by treatment groups and internally calculated lowly expressed genes were filtered. MDS plots were used to ensure that samples separated by treatment. Exact edgeR tests were conducted for mucus and plasma because they have two treatment groups (control vs. stressed) and samples are not paired since they were collected from separate fish in each treatment (Figure 1). For water, since the control and stressed samples were collected from the same tank of water, samples were paired. Therefore, data for water were analyzed by use of a generalized linear model using the fish number as the blocking factor. All results were exported after being sorted by false discovery rate (FDR) to identify significantly differentially expressed miRNA.

For analysis using DESeq2, the read count file was split by sample type so that library normalization would not be affected by types of samples. Following this, the DESeq2 data set was created from the matrix of read counts. This step filters and normalizes the read counts. For plasma and mucus, the design was solely based on the treatment (control vs. stressed), but due to the paired nature of the water samples, the fish number was added in as the blocking factor. These DESeq2 datasets were used to conduct differential expression analysis. Results were obtained and ordered via adjusted *p*-value (equivalent to false discovery rate) and exported to identify significant results.

Statistical significance for differentially expressed miRNA was defined as miRNA with *p* values ≤ 0.05, adjusted *p* values or false discovery rates (FDR) <0.1, and log fold changes greater than 2 and less than −2. These cutoffs were chosen based on acceptable thresholds for RNA-Seq data and by the amount of false positives we allowed to ensure reliable data while minimizing false negative results (Renthal et al., 2018). The data produced in this study have been deposited in NCBI’s Gene Expression Omnibus and are accessible through GEO Series accession number GSE151138 (Edgar et al., 2002; Barrett et al., 2013).

#### Target Prediction and Pathway Analysis of Differentially Expressed Plasma MicroRNA

The differentially expressed miRNAs in plasma samples were used to predict 3′UTR targets *in silico* for *Salmo salar* (the closest related species present in KEGG and DAVID databases) using the miRanda command line tool (Enright et al., 2003; Berthelot et al., 2014; Lien et al., 2016). miRanda uses sequence alignment based on complementarity and RNA stability of the binding (Zuker and Stiegler, 1981; McCaskill, 1990; Hofacker et al., 1994; Enright et al., 2003). Each known, significantly differentially expressed miRNA sequence in plasma was input into the miRanda tool and compared against a curated list of *Salmo salar* 3′ untranslated regions (UTRs) (Lynch, 2020)^2^. Since predicted miRNAs were not validated, novel differentially expressed miRNAs were omitted to improve reliability of the *in silico* predictions. Only mRNA with a complementarity score greater than 140 and an energy score (Δ G) less than −20 were used for pathway analysis (Kostyniuk et al., 2019; Cadonic et al., 2020). The script can be accessed in the Supplementary Methods (one-miRNA-all-3UTR.miranda_analysis.sh).

All mRNA targets identified using miRanda were combined into a common list and converted to UniProt IDs^3^. This list was submitted to KEGG Search & Color pathway (specifying *Salmo salar* and UniProt IDs; Kanehisa and Goto, 2000; Kanehisa, 2019; Kanehisa et al., 2019). These results were analyzed for known responses to acute stress and to curate a list of percentages of proteins targeted in pathways to identify enriched pathways. The combined *Salmo salar* UniProt ID list was also uploaded to the DAVID Functional Annotation tool (Huang et al., 2009a, b)^4^. The recommended options for functional clustering and medium stringency, as well as a cutoff of an enrichment score of −1.3 or less (equivalent to *p* ≤ 0.05) were used (Huang et al., 2007). This analysis was only performed using altered plasma miRNA, as the biological role of mucus and water miRNA is unknown and pathway analysis of targets would be speculative.

### Validation of Differentially Expressed MicroRNA Using RT-qPCR

The known, differentially expressed microRNA in water samples identified using RNA-Seq and subsequent analysis were validated by measuring expression via RT-qPCR. RNA from separate, non-sequenced plasma, mucus, and water samples was extracted using the RNA extraction techniques previously mentioned with the exception of the addition of 3.5 μL of 1.6 × 10^8^ copies of RNA spike-in (cel-miR-39, Qiagen) following the lysis step. MicroRNA was quantified using the Qubit microRNA Assay kit (Thermo Fisher Scientific). To synthesize cDNA, two methods were used, converting a common volume of extracted miRNA to cDNA (common volume cDNA) and converting a common amount of miRNA to cDNA (common amount cDNA) to assess different methods of gene expression normalization (endogenous control U6, cel-39 spike-in RNA, or total amount of small RNA). For common volume cDNA, 12 μL extracted plasma RNA, 5 μL extracted mucus RNA, and 12 μL extracted water RNA were added to their respective reactions (HiSpec buffer; Qiagen miScript II RT kit). For common amount cDNA, 3 ng plasma miRNA and 140 ng mucus miRNA were added to their respective reactions (HiSpec buffer; Qiagen miScript II RT kit). No common amount cDNA was made for water samples. The qPCR reactions consisted of 5 μL 2× Bio-Rad SsoAdvanced SYBR, 1 μL of 5 μM forward primer (see Table 1), 1 μL of 5 μM Qiagen Universal Reverse Primer, 2 μL RNase-free water, and 1 μL 1:10 diluted cDNA performed in triplicate. The qPCR protocol consisted of a one-time polymerase activation step at 95°C for 30 s, followed by denaturing at 95°C for 10 s, annealing and extension at 60°C for 20 s, and a plate read, which was repeated for a total of 40 cycles. Each qPCR run also included a melt curve analysis which was performed by increasing the temperature from 65 to 95°C every 5 s in 0.5°C increments with a plate read at each increment. Non-template controls were used to ensure no contamination or primer-dimerization occurred. Expression was calculated by using the relative quantity (as calculated by the Bio-Rad Maestro software) and then, as appropriate, normalized to one of the following: amount of miRNA input (only possible for common volume cDNA), synthetic cel-39 spike-in, and/or the relative quantity of endogenous control U6. Statistical analysis included testing for normality and equal variances and performing a one-tailed *t*-test (*p* < 0.05, *n* = 3). For miR-26a-5p, no statistical analysis was performed as it was a presence/absence test. Pearson correlation coefficients were calculated to compare RNA-Seq and qPCR results. These values were calculated between the normalized read counts from the tool (DESeq2, edgeR) that identified the miRNA as differentially expressed and the RT-qPCR expression values.

**TABLE 1:** Primers used to measure microRNA expression via RT-qPCR.

Name	Sample	Accession	Sequence (5′ to 3′)
ssa-miR-16b-5p	Plasma	MIMAT0032408	TAGCAGCACGTAAATATTGGTG
ssa-miR-30b-5p	Water, Mucus	MIMAT0032604	TGTAAACATCCCCGACTGGAAGCT
ssa-miR-26a-5p	Water	MIMAT0032563	TTCAAGTAATCCAGGATAGGCT
U6 – Forward	Plasma, Mucus	XR_005037161.1	CTCGCTTCGGCAGCACATA
U6 – Reverse			AGGAACGCTTCACGAATTTGC
Ce_miR-39_1 miScript Primer Assay		MIMAT0000010	n/a
Qiagen Universal Reverse Primer			GAATCGAGCACCAGTTACGC

*The sample within which the targets were measured are indicated by the sample column. Where applicable, the miRbase.org or NCBI accession number is included. The miRNA sequences were all used as forward primers, with the Qiagen Universal Reverse primer as the reverse primer.*

## Results

### Validation of Stress Response

**FIGURE 2:**
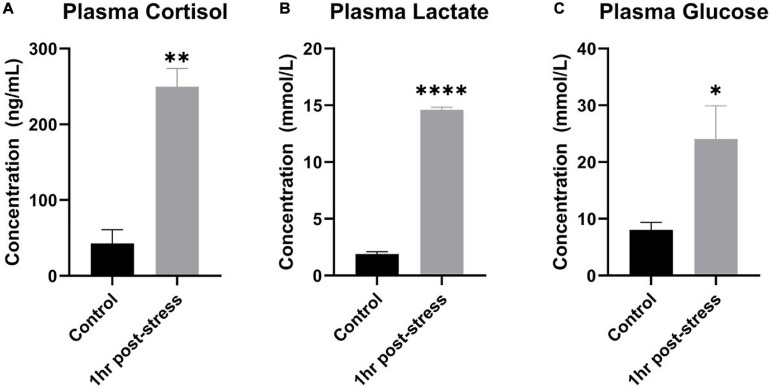
Plasma cortisol, lactate, and glucose increased following acute stress. Mean ± SEM of plasma cortisol **(A)**, lactate **(B)**, and glucose **(C)** before (control) and 1 h after a 3-min air exposure stressor (1 h post-stress) in rainbow trout. Asterisks indicate a significant difference in the measure (*n* = 3; one-tailed *t*-test). A single asterisk indicates *p* ≤ 0.05, double asterisks indicate *p* ≤ 0.01, and four asterisks indicate *p* ≤ 0.0001.

To demonstrate that 3 min out of water in ambient air caused a physiological response in rainbow trout, plasma cortisol, lactate, and glucose in blood plasma were measured. One-hour post air exposure, a statistically significant 5-fold increase in plasma cortisol, a significant 7-fold increase in plasma lactate, and a significant 3-fold increase in plasma glucose were measured (one-tailed *t*-test, *n* = 3, *p* < 0.05; Figure 2). Data were normally distributed and had equal variance.

### Sequencing Metadata and *in silico* Validation

**FIGURE 3:**
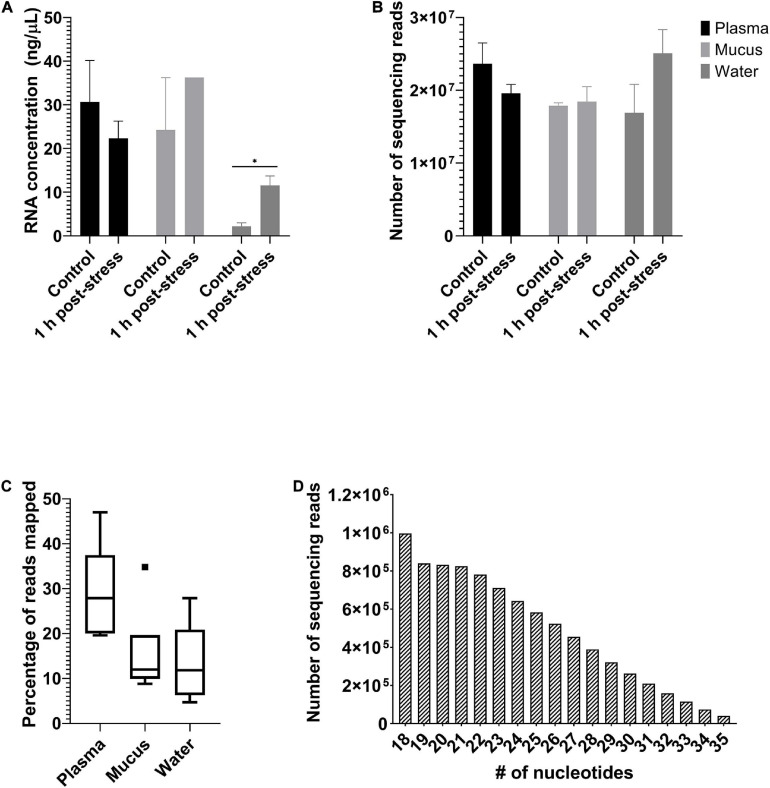
Sequencing metadata. Mean ± SEM of **(A)** extracted RNA concentrations (ng/μL) and **(B)** number of sequencing reads from plasma, mucus, and water samples. Extracted RNA concentrations did not differ between treatments within each sample, but did differ as a function of sample type, with water being significantly less than those of plasma and mucus. Number of reads did not differ among treatments or sample type. Significant differences among samples indicated by an asterisk (two-way ANOVA, *n*_*sample*_ = 6, *n*_*treatment*_ = 3, *p* ≤ 0.05). Box plot **(C)** of the percentage of sequencing reads mapped to the rainbow trout genome by miRDeep2. Minimum, first quartile, median, third quartile, and maximum are indicated by the horizontal lines (when reading from bottom to top). Outliers are indicated by a filled square. Histogram **(D)** of the number of sequencing reads at each read length between 18 and 35 nucleotides.

Eighteen samples were sequenced, which includes six samples of plasma, six samples of mucus, and six samples of water (*n* = 3 for each treatment). Water had less total RNA than did plasma or mucus. However, there was no significant difference (two-way ANOVA; *n*_*sample*_ = 6, *n*_*treatment*_ = 3; *p* < 0.05; Figure 3A) between samples from control or stressed individuals. Sequencing depth of the samples was between 12 and 30 million reads and did not differ based on sample type or treatment (two-way ANOVA; *n*_*sample*_ = 6, *n*_*treatment*_ = 3; *p* < 0.05; Figure 3B). Detailed RNA concentrations and read counts for each sample are available in the Supplementary Results (RNA concentrations and Read counts tabs). Heat maps for each sample type and analysis are provided (Heat maps_edgeR and Heat maps_DESeq2 tabs). Quality analysis was performed on raw and processed samples and the sequences passed all requirements for use in this analysis. Complete FastQC reports can be accessed in the Supplementary Quality Control File.

All sample types were mapped to the rainbow trout genome, with some variation in the amount mapped by sample type (Figure 3C). Overall, there were between 94 and 182 unique known miRNAs annotated in each sample (Supplementary Results – Number of microRNA tab). The miRNAs had a median read length of 23 nucleotides (Figure 3D). The log file from the alignment, annotation, and read counting through miARma-Seq indicated that all steps occurred without any errors. Complete stats and log files from the alignment, annotation, and read counting using miARma-Seq (which uses bowtie1 and miRDeep2) are available in the Supplementary Results (Stats file and Log file tabs).

### Identification of Differentially Expressed MicroRNA Using High Throughput Sequencing

High throughput sequencing and bioinformatic analysis were used to identify altered miRNA in rainbow trout plasma, mucus, and water in which the fish were held, following acute stress. The statistical tools used, edgeR and DESeq2, identified differentially expressed miRNA and though there were some shared results, most differentially expressed miRNAs identified by each tool are unique to that tool. The read sequences for all known and novel miRNAs identified in this study are deposited in figshare (Ikert, 2020b)^5^.

#### Differentially Expressed Plasma MicroRNA and *in silico* Target Prediction

Ten miRNAs were significantly altered in plasma following air exposure (Table 2). Plasma miRNAs increased with a log 2-fold change between 11 and 21 and miRNAs decreased with a log 2-fold change of −21. Both edgeR and DESeq2 identified miR-16b-5p as significantly altered with an increase of approximately 12-fold following acute stress.

**TABLE 2:** List of novel and known differentially expressed microRNA (miRNA) in plasma.

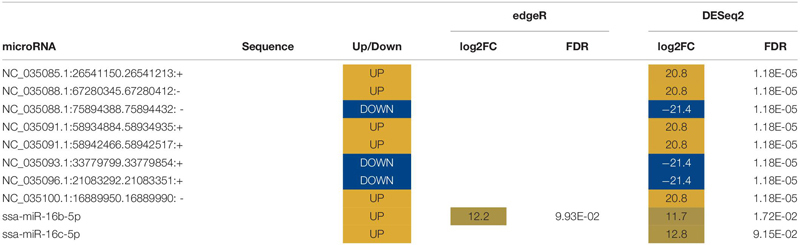

*The miRNAs referred to as *Oncorhynchus mykiss* genomic locations are novel and miRNAs referred to as ssa-miR-## are known. MicroRNAs that increase following the stressor are indicated by UP (yellow or lighter shade background) and those that decrease following the stressor are indicated by DOWN (blue background or darker shade background). If the differentially expressed miRNA was identified using edgeR or DESeq2, there is a log2 fold change and false discovery rate (FDR) indicated in their respective column. MicroRNAs with data in both edgeR and DESeq2 were determined to be significantly altered using both tools (FDR < 0.1). MicroRNAs are ordered from smallest to largest FDR.*

Two known miRNAs, ssa-16b-5p and ssa-16c-5p, were upregulated with an approximate log 2-fold change of 12 following stress (Table 2). These two known miRNAs were used to predict mRNA targets *in silico*. The miRanda algorithm predicted that miR-16b-5p targeted 2517 mRNA and miR-16c-5p targeted 3171 mRNA. These results were combined for gene enrichment (DAVID) and pathway analysis (KEGG) as both miRNAs were increased so the targeted mRNA would all be predicted to decrease. DAVID functional enrichment analysis indicated a statistically significant decrease in transmembrane proteins and their activity (Supplementary Results – DAVID results tab). Biosynthetic, degradation, and metabolic KEGG pathways were predicted to be targeted by altered plasma miRNAs (Table 3). Thirteen mRNA in the adrenergic signaling in cardiomyocytes KEGG pathway were targeted. Specifically, the beta-2-adrenergic receptor, which binds adrenaline and noradrenaline, was predicted to be downregulated via miR-16b-5p and miR-16c-5p. Complete results from miRanda target prediction, KEGG pathway analysis, and DAVID functional enrichment analysis can be found in the Supplementary Results (miRanda results 16b-5p, miRanda results 16c-5p, miRanda combined UniProt, KEGG results, KEGG percentages of pathways tabs).

**TABLE 3:** Altered plasma miRNAs target biosynthetic, degradation, and metabolic KEGG pathways.

KEGG pathway	% of pathway targeted	Number of mRNA targeted	Number of mRNA in pathway
sasa00280 Valine, leucine, and isoleucine degradation	30.00	3	10
sasa00232 Caffeine metabolism	25.00	1	4
sasa00514 Other types of O-glycan biosynthesis	16.36	9	55
sasa05132 Salmonella infection	9.81	21	214
sasa00515 Mannose type O-glycan biosynthesis	9.72	7	72
sasa00533 Glycosaminoglycan biosynthesis – keratan sulfate	9.43	5	53
sasa00601 Glycosphingolipid biosynthesis – lacto and neolacto series	8.99	8	89
sasa00072 Synthesis and degradation of ketone bodies	7.14	1	14
sasa00563 Glycosylphosphatidylinositol (GPI)-anchor biosynthesis	6.25	2	32
sasa00260 Glycine, serine and threonine metabolism	6.10	5	82
sasa00511 Other glycan degradation	5.88	2	34
sasa00512 Mucin type O-glycan biosynthesis	5.62	5	89
sasa00534 Glycosaminoglycan biosynthesis – heparan sulfate/heparin	5.13	4	78
sasa00603 Glycosphingolipid biosynthesis – globo and isoglobo series	5.08	3	59

*The number of targeted mRNA in each KEGG pathway was compared to the total number of mRNA in each pathway to identify pathways with large percentages of mRNA targeted. Only pathways with five percent or more of pathway targeted where included.*

#### Differentially Expressed MicroRNA in Mucus

Abundances of sixteen miRNAs were significantly altered in mucus following the stress caused by being out of water (Table 4). Seven known miRNAs were altered following stress (Table 4). Differentially expressed mucus miRNAs increased with a magnitude between a log2 fold change of 8 and 30 and decreased with a magnitude between a log2 fold change of −9 and −28. One miRNA, miR-26a5p, was identified by both edgeR and DESeq2 to increase by a log2 fold change of 15.

**TABLE 4:** List of novel and known differentially expressed microRNA (miRNA) in mucus.

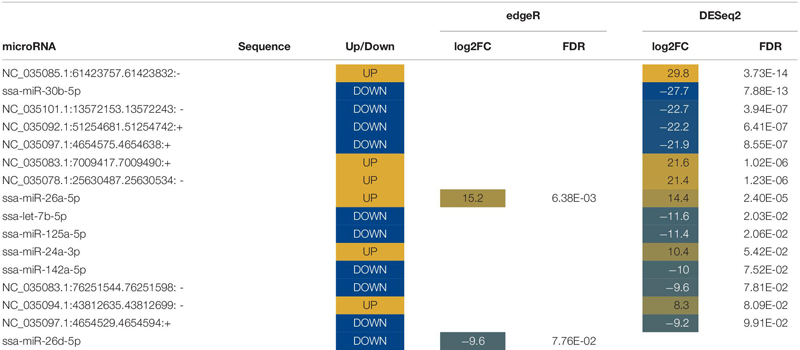

*The miRNAs referred to as *Oncorhynchus mykiss* genomic locations are novel and miRNAs referred to as ssa-miR-## are known. MicroRNAs that increase following the stressor are indicated by UP (yellow or lighter shade background) and those that decrease following the stressor are indicated by DOWN (blue background or darker shade background). If the differentially expressed miRNA was identified using edgeR or DESeq2, there is a log2 fold change and false discovery rate (FDR) indicated in their respective column. MicroRNAs with data in both edgeR and DESeq2 were determined to be significantly altered using both tools (FDR < 0.1). MicroRNAs are ordered from smallest to largest FDR.*

#### Differentially Expressed MicroRNA in Water

Abundances of seventy miRNAs were significantly altered in water following stress (Tables 5, 6). Twelve known miRNAs were upregulated following stress (Table 5). Differentially expressed miRNAs in water increased between a log2 fold change between 7 and 20 and decreased with a log2 fold change between −3 and −30. Six miRNAs, five known and one novel, were identified by both edgeR and DESeq2 to be differentially expressed.

**TABLE 5:** List of known differentially expressed microRNA (miRNA) in water.

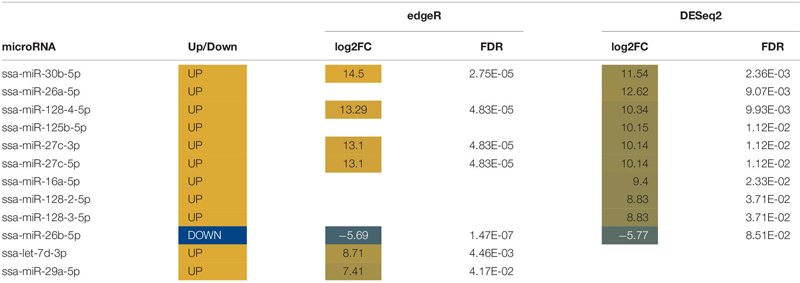

*MicroRNAs that increase following the stressor are indicated by UP (yellow or lighter shade background) and those that decrease following the stressor are indicated by DOWN (blue background or darker shade background). If the differentially expressed miRNA was identified using edgeR or DESeq2, there is a log2 fold change and false discovery rate (FDR) indicated in their respective column. MicroRNAs with data in both edgeR and DESeq2 were determined to be significantly altered using both tools. MicroRNAs are ordered from smallest to largest FDR.*

**TABLE 6:** List of novel differentially expressed microRNA (miRNA) in water.

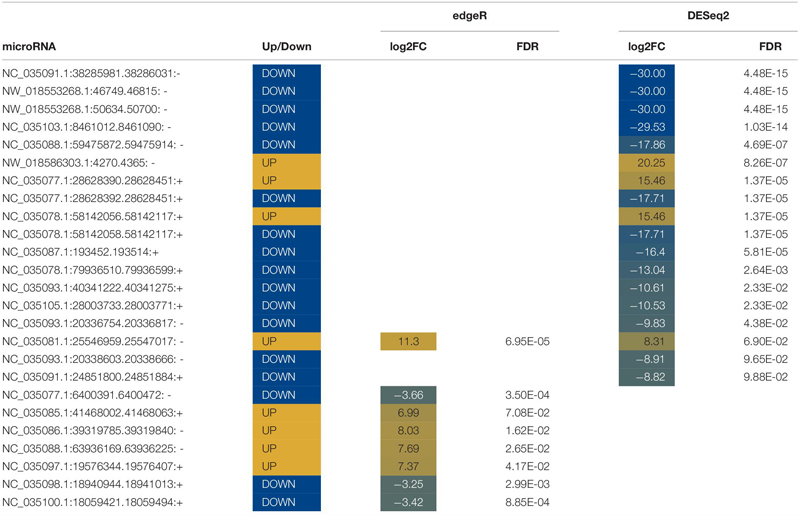

*MicroRNAs that increase following the stressor are indicated by UP (yellow or lighter shade background) and those that decrease following the stressor are indicated by DOWN (blue or darker shade background). If the differentially expressed miRNA was identified using edgeR or DESeq2, there is a log2 fold change and false discovery rate (FDR) indicated in their respective column. MicroRNAs with data in both edgeR and DESeq2 were determined to be significantly altered using both tools. MicroRNAs are ordered from smallest to largest FDR.*

#### Comparison of Known Differentially Expressed MicroRNA in All Sample Types

**FIGURE 4:**
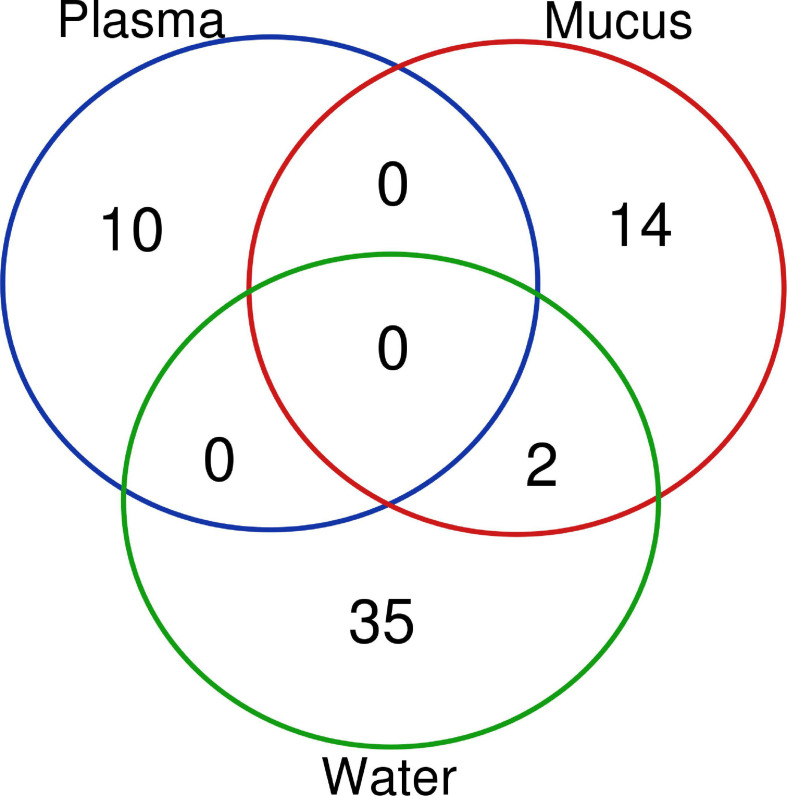
A Venn diagram of all differentially expressed microRNA in each sample type. Differentially expressed miRNAs (*p* ≤ 0.05, FDR < 0.1, log fold change < |2|) shared between each sample type are indicated by the overlap of the circle and by the number of miRNAs that are shared. Altered miRNA profiles were mostly unique to matrix type.

Differentially expressed miRNAs were compared across sample types (plasma, mucus, water) to understand whether altered miRNA profiles are conserved between sample types. No altered miRNAs were shared between plasma and the other sample types (Figure 4). Only two differentially expressed miRNAs, miR-30b-5p and miR-26a-5p, were shared between water and mucus (Figure 4). Mucus and water had inverse expressions of miR-30b-5p (decreased in mucus, increased in water), whereas miR-26a-5p was increased in both mucus and water samples taken from 1 h post air-stressed fish (Tables 4, 5).

### Validation of Differentially Expressed MicroRNA Using RT-qPCR

Differentially expressed miRNAs identified via RNA-Seq were measured via RT-qPCR in un-sequenced samples to validate the RNA-Seq results and to develop a RT-qPCR assay for measuring miRNA expression changes in water. Several normalization methods for miRNA expression were tested during RT-qPCR validation. Expression of ssa-mir-16b-5p in plasma samples was normalized by using a common miRNA input amount (3 ng) into the cDNA reaction. Expression of ssa-miR-30b-5p in mucus samples was normalized by using a common miRNA input amount (140 ng) into the cDNA reaction and normalizing to cel-miR-39 expression (corrected for input volume of spike-in). Expression of ssa-miR-30b-5p and ssa-miR-26a-5p in water samples was normalized by using a common miRNA volume input (12 μL) and normalizing to miRNA input (quantified by Qubit).

**FIGURE 5:**
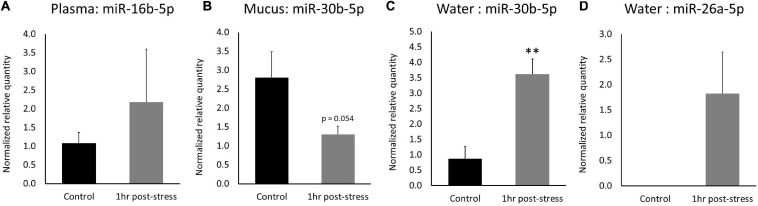
RT-qPCR expression of differentially expressed miRNAs. Mean expression ± SEM of **(A)** ssa-miR-16b-5p in plasma, **(B)** ssa-miR-30b-5p in mucus, **(C)** ssa-miR-30b-5p in water, and **(D)** ssa-miR-26a-5p in water. Significant different between treatments indicated by asterisks (one-tailed *t*-test, *n* = 3, *p* < 0.05).

In plasma samples, ssa-miR-16b-5p expression increased by a log2 fold change of 1.01 1-h post-stress (*p* = 0.24; Figure 5A). Correlation between RNA-Seq and RT-qPCR values in plasma was 0.76 (DESeq2) and 0.89 (edgeR). In mucus samples, ssa-miR-30b-5p expression decreased by a log2 fold change of −1.40 1-h post stress (*p* = 0.054; Figure 5B). Correlation between RNA-Seq and RT-qPCR values in mucus was −0.17 (DESeq2). In water samples, ssa-miR-30b-5p expression increased by a log2 fold change of 2.05 (*p* = 0.0061) 1-h post-stress (Figure 5C). In addition, ssa-miR-26a-5p was only measured in the stressed samples (present) and was absent in the control samples (Figure 5D). Correlation between RNA-Seq and RT-qPCR values in water was 0.93 (DESeq2) and 0.94 (edgeR) for miR-30b and 0.98 (DESeq2) for miR-26a. Relative expression and calculations are found in the Supplementary Results (qPCR_results tab).

## Discussion

The objective of this study was to understand how microRNAs of rainbow trout are altered in plasma, mucus, and water in response to acute stress. The changes in plasma miRNA expression 1-h post stressor were determined. MicroRNAs were isolated from epidermal mucus and surrounding water. Alterations in relative amounts of miRNAs were observed in both mucus and water following acute stress. One-hour after acute stress there were only two significantly altered miRNAs that were identified in both mucus and adjacent water.

### Role of Plasma MicroRNA in Physiological Responses to Stress

Following acute stress, seven miRNAs in plasma were upregulated, and three miRNAs were downregulated. This is the first identification of all miRNAs in blood plasma following acute stress of a teleost. The current study was restricted to examining the profile of miRNAs in plasma at a single time. Additional studies are required to further describe the dynamics or responses of miRNAs to stressor time points within 1 h, when cortisol is increasing, and after 1 h, as the stress response is decreasing.

The altered profile of miRNAs in plasma was used to perform pathway analyses *in silico* to identify putative targets of miRNA. That analysis identified targeted pathways of biosynthesis, degradation, and metabolism. None of the targets are associated with well-known molecular stress response signaling, such as the glucocorticoid receptor (Ducouret et al., 1995). However, miR-16b-5p and miR-16c-5p were predicted to cause a decrease in thirteen targets of the adrenergic signaling in cardiomyocytes, including receptors for adrenaline and noradrenaline, the catecholamines that increase within seconds to minutes of the acute stress response, which would be predicted to be less active at 1-h post stress (Randall and Ferry, 1992; Wendelaar Bonga, 1997; Tort, 2013). These results are *in silico* predictions and the ability of miR-16b-5p and miR-16c-5p to target the mRNA would need to be validated by use of 3′UTR luciferase assays (Zeng and Cullen, 2003). Predictions made *in silico* during the current study are useful in developing future hypotheses to further understanding of how acute responses to stress are regulated in fishes.

### Benefits and Limitations of Each Non-lethal Sampling Location

MicroRNAs in plasma, mucus, and water can all be collected non-lethally, but each sample type has benefits and limitations. These are discussed here in order to provide applications and advise areas of future study of altered miRNAs in each of these sample types. For both plasma and mucus, miRNA can be associated with specific fish being sampled. However, fish must be caught and handled, which is invasive and time consuming and can confound the acute stress response (Brydges et al., 2009). Measurements of miRNA in plasma have the benefit of being associated with internal tissue responses as biomarkers of disease states (Cui and Cui, 2020). However, non-lethal sampling of blood from fish for identification and quantification of miRNA conservatively requires a minimum fish mass of 500 g. This is due to the most conservative, non-lethal blood sampling guideline to take no more than 1 mL blood per 1 kg of total fish mass and the requirement of 0.5 mL of blood to collect the 0.2 mL plasma required to extract miRNA (Canadian Council on Animal Care, 2005; Hawkins et al., 2011; Lawrence et al., 2020). Additionally, many sentinel species are small-bodied fish, which would require lethal sampling to collect blood plasma, so less invasive methods are advised (Bahamonde et al., 2015; Thornton et al., 2017; Arlos et al., 2018). Mucus can be collected non-lethally from fish smaller than 500 g, though the amount of mucus that can be collected from a fish without causing unacceptable stress or resulting in infections is not yet defined (Canadian Council on Animal Care, 2005). Though changes in miRNAs in mucus following acute stress have been suggested as a safe and effective way to monitor for stress responses, the biological roles of miRNAs associated with mucus are not yet understood. Collection of water in which fish have resided is completely non-invasive, requires limited training, and can be collected regardless of season. However, miRNA measured in the water column are not associated with one organism and can only be associated with a particular fish species if there is only a single species present in the water. Within environmental samples, plant and animal miRNA can be differentiated easily based on sequence (Hertel and Stadler, 2015). However, due to conservation of miRNA between fish and other vertebrates, measurement of miRNA from the aquatic environment would be indicative of the entire fish or vertebrate community (Hertel and Stadler, 2015). Though this could be detrimental when attempting to study the health status of a particular fish species, this can be leveraged as a method to understand the health status of the entire aquatic community; removing the need to use only fish or a particular fish species as sentinels of environmental health.

### Unknown Sources of MicroRNAs in Mucus and Water

Two of the sixteen differentially expressed mucus miRNAs were altered in water, which indicates mucus is a possible source of some of the miRNAs. This was expected since one source of DNA in water (eDNA) is mucus of fishes, but the actual source or sources of miRNAs in water is not known and mucus could be only one source of the 37 altered miRNA in water. None of the altered plasma miRNA profile is shared with the mucus or water miRNA profiles indicating that perhaps secretion of miRNA from plasma into mucus or water does not occur immediately. These unique profiles of miRNA indicate that the source of altered miRNA in water is not known 1 h after acute stress. Sources of mucus itself are goblet, sacciform, and club cells (Fasulo et al., 1993; Shephard, 1993; Zaccone et al., 2001). MicroRNAs in mucus could be packaged in exosomes or associated with proteins and secreted from these cells or epidermal cells themselves. eDNA of fishes originates from feces, urine, gametes, mucus, scales, blood, and secretions (Höss et al., 1992; Valiere and Taberlet, 2000; Ficetola et al., 2008; Jerde et al., 2011). Therefore, miRNA can be sampled from these locations and compared to profiles of miRNA in mucus and water to better understand sources of environmental miRNA. Differentially expressed miRNA in each of the samples could be fluorescently labeled to track paths of secretion (Manca et al., 2018). Identifying the destination of plasma miRNA and the source of mucus and water miRNA would allow us to understand the role of miRNA in responding to stress and facilitate the development of miRNA as a reliable, non-lethal biomarker of stress.

### Considerations for RNA-Seq Analysis of MicroRNA and RT-qPCR Validation

To identify differentially expressed miRNA in sequenced samples from rainbow trout, the miARma-Seq pipeline was applied (Andrés-León et al., 2016; Andrés-León and Rojas, 2019). We have tested the pipeline and the results that it produced *in silico*. Since rainbow trout are not a model organism and their miRNAs are not present in miRbase, even though they have been sequenced (Juanchich et al., 2016), alternative references and indexes had to be curated for this analysis and can be used in future analyses. The customized options have been outlined in the methods and resources are available in the Supplementary Material. These options and resources can be useful to others analyzing rainbow trout miRNA via RNA-Seq.

A concern when analyzing these RNA-Seq data was the low mapping percentages of reads to the rainbow trout genome and Atlantic salmon miRNA. When comparing plasma mapping percentages, it was found to be within range of human plasma miRNA mapping (Dufourd et al., 2019). The mapping percentages were less for mucus and water and because miRNA in these samples had not been measured previously, there is no comparison to ensure this is acceptable. The observed lesser percentages can be attributed to other environmental sources of RNA present. Therefore, future sequencing of environmental samples could focus on enriching miRNA prior to sequencing as well as further *in silico* filtering of extraneous RNA. The unmapped RNA can be identified and analyzed to determine if other small RNA are altered following acute stress. These mapping percentages of miRNA present in mucus and water can be used as a threshold for future research.

To validate the RNA-Seq results and the miRNAs as biomarkers, miRNAs (ssa-miR-16b-5p, ssa-miR-30b-5p, ssa-miR-26a-5p) were measured via RT-qPCR in un-sequenced plasma, mucus, and water samples. The RNA-Seq and RT-qPCR results are highly correlated when comparing statistically significant results and using Pearson’s correlation coefficient. However, though miRNA expression has extensively been measured in plasma (Mitchell et al., 2008; Wu et al., 2012; Sourvinou et al., 2013; Brunet-Vega et al., 2015; Vigneron et al., 2016; Zhao et al., 2016; Scrutinio et al., 2017; Binderup et al., 2018; Poel et al., 2018), and has been measured once in mucus (Zhao et al., 2020) using RT-qPCR, there is still much debate as to the appropriate methods of normalization. We tested a number of methods (common miRNA input amount and volume, synthetic spike-in, and a small RNA reference gene) during our validation. However, more robust methods should be established. Here we have measured miRNA expression from water samples via RT-qPCR for the first time and determined that normalizing to miRNA input amount is a successful method in this sample type. Therefore, the RT-qPCR validation confirms the RNA-Seq results, extending the usefulness of these miRNAs as biomarkers since the qPCR validation was performed on separate samples, and highlighting the potential to develop more robust methods of measuring miRNA expression in plasma, mucus, and water samples.

### Future Development of MicroRNA as Non-lethal Indicators of Stress

Since changes in expression patterns of miRNA in blood, mucus, and water following acute stress in rainbow trout were observed, this work can be expanded by measuring changes in miRNA in response to other stressors and in other aquatic organisms to test the conservation of the miRNA response. Due to the ability to non-lethally measure miRNA and the potential functional relationships to internal mRNA changes, blood, mucus, and/or water miRNA could be developed as biomarkers of stress in fish and the aquatic environment. The benefit to developing miRNA as indicators of fish stress is that in tissues, specific miRNAs are altered in response to specific stressors (ex., Gonçalves et al., 2020; Liu et al., 2020a, b; Qiang et al., 2020). Therefore, if this is true in non-lethal samples, non-lethally collected miRNA can provide not only an early indicator of fish stress but also provide an indicator of what type of fish stress (ex., temp, metals) is being experienced, allowing for earlier, targeted remediation to be implemented. Due to the current collection and analysis of DNA in the water being termed environmental DNA (eDNA), we propose that measurements of miRNA in water be termed environmental miRNA (e-microRNA or e-miRNA). E-miRNA can currently be extracted from the same water samples or filter used in eDNA collection and can potentially be developed as a method of simultaneously measuring fish stress as well as fish species present.

## Data Availability Statement

The datasets presented in this study can be found in online repositories. The names of the repository/repositories and accession number(s) can be found below: the NCBI Gene Expression Omnibus (GSE151138).

## Ethics Statement

The animal study was reviewed and approved by the Animal Care Committee – University of Waterloo.

## Author Contributions

HI conducted the experimental design, collected and analyzed data, and wrote the manuscript. ML assisted in bioinformatic data analysis and edited the manuscript. MS, AD, and JG acquired grant funding. BK acquired funding and edited the manuscript. PC acquired funding, assisted in experimental design and manuscript writing, and edited the manuscript. All authors contributed to the article and approved the submitted version.

## Conflict of Interest

The authors declare that the research was conducted in the absence of any commercial or financial relationships that could be construed as a potential conflict of interest.

## Footnotes

1 https://github.com/rajewsky-lab/mirdeep2

2 https://doi.org/10.6084/m9.figshare.12461969.v1

3 https://www.uniprot.org/uploadlists/

4 https://david.ncifcrf.gov/

5 https://doi.org/10.6084/m9.figshare.12974792.v1
