# Supplementary material for: High Throughput Sequencing of MicroRNA in Rainbow Trout Plasma, Mucus, and Surrounding Water Following Acute Stress
Source: Front Physiol. 2021 Jan 13;11:588313. doi: 10.3389/fphys.2020.588313 (PMC7838646; doi:10.3389/fphys.2020.588313)
Supplement: Supplementary file 2 [file Data_Sheet_1.ZIP › Supplemental Quality Control/FastQC_processed_files/mucus_control_1_fastqc_processed.html]

size\_trimmed\_adapterless\_SV18263\_0007\_S19\_R1\_001.fastq FastQC Report 

FastQC Report

Fri 8 May 2020  
size\_trimmed\_adapterless\_SV18263\_0007\_S19\_R1\_001.fastq

## Summary

- Basic Statistics
- Per base sequence quality
- Per tile sequence quality
- Per sequence quality scores
- Per base sequence content
- Per sequence GC content
- Per base N content
- Sequence Length Distribution
- Sequence Duplication Levels
- Overrepresented sequences
- Adapter Content

## Basic Statistics

| Measure | Value |
| --- | --- |
| Filename | size\_trimmed\_adapterless\_SV18263\_0007\_S19\_R1\_001.fastq |
| File type | Conventional base calls |
| Encoding | Sanger / Illumina 1.9 |
| Total Sequences | 16566625 |
| Sequences flagged as poor quality | 0 |
| Sequence length | 18-35 |
| %GC | 54 |

## Per base sequence quality

## Per tile sequence quality

## Per sequence quality scores

## Per base sequence content

## Per sequence GC content

## Per base N content

## Sequence Length Distribution

## Sequence Duplication Levels

## Overrepresented sequences

| Sequence | Count | Percentage | Possible Source |
| --- | --- | --- | --- |
| TTGGCAGGTGAGTAGAGCCGTTCGTGACA | 2067546 | 12.480188330453547 | No Hit |
| TTGGCAGGTGAGTAGAGCCGTTCGTGA | 1448366 | 8.742673900085261 | No Hit |
| GCATTGGTGGTTCAGTGGTAGAATTCTCGCC | 934808 | 5.642718417299843 | No Hit |
| GCATTGGTGGTTCAGTGGTAGAATTCTCGC | 916947 | 5.534905268876431 | No Hit |
| CTTTTGGCAGGTGAGTAGAGCCGTTCGTGACA | 861828 | 5.202194170508477 | No Hit |
| CCGAGAAGACGATCAAACTTGA | 615104 | 3.7129107467574114 | No Hit |
| CGGCGACTCTGGACGCGTGCC | 460386 | 2.778996929066723 | No Hit |
| AGCGGCGACTCTGGACGCGTGCC | 393627 | 2.376024084567617 | No Hit |
| CTTTTGGCAGGTGAGTAGAGCCGTTCGTGA | 306109 | 1.8477450899021375 | No Hit |
| GCGGCGACTCTGGACGCGTGCC | 302791 | 1.827716870515268 | No Hit |
| TTTTGGCAGGTGAGTAGAGCCGTTCGTGACA | 257758 | 1.5558872129960086 | No Hit |
| TGAGAACTGAATTCCATAGATGG | 249863 | 1.5082311575230318 | No Hit |
| GGCGACTCTGGACGCGTGCC | 239375 | 1.444923151215169 | No Hit |
| GCCGAGAAGACGATCAAACTTGA | 214021 | 1.2918805127779498 | No Hit |
| CGTGGAGCTTCGGTTGGCCCGGGATAGCCTGCCT | 192090 | 1.159499898138577 | No Hit |
| GCATTGGTGGTTCAGTGGTAGAATTC | 188188 | 1.1359465189801785 | No Hit |
| TTTTGGCAGGTGAGTAGAGCCGTTCGTGA | 177325 | 1.0703749254902553 | No Hit |
| GCATTGGTGGTTCAGTGGTAGAATTCTC | 125165 | 0.7555250390468788 | No Hit |
| GCAGCGGCGACTCTGGACGCGTGCC | 115698 | 0.6983800261067055 | No Hit |
| GGAATACCAGGTGCTGTAAGCTT | 94150 | 0.5683112885092769 | No Hit |
| CAGGTGAGTAGAGCCGTTCGTGACA | 79006 | 0.47689858374895305 | No Hit |
| GATCGGGGGCCTGAGTCCT | 77446 | 0.46748206107158213 | No Hit |
| GCATTGGTGGTTCAGTGGTAGAATTCTCG | 76231 | 0.4601480386017067 | No Hit |
| TTGGCAGGTGAGTAGAGCCGTTCGTGAC | 71753 | 0.4331177895316638 | No Hit |
| TGGGAATACCAGGTGCTGTAAGCTT | 71687 | 0.43271939818762123 | No Hit |
| TGATGCGCACCGCATGTTTGTGGAGAACC | 69340 | 0.4185523605441664 | No Hit |
| TTTGGCAGGTGAGTAGAGCCGTTCGTGACA | 69030 | 0.4166811284736631 | No Hit |
| GCATTGGTGGTTCAGTGGTAGAATTCTCGCCT | 65772 | 0.39701508303592314 | No Hit |
| CTTTTGGCAGGTGAGTAGAGCCGTTCGTGACAG | 64101 | 0.38692853855266235 | No Hit |
| GTCTGGCGGGCACGGGAAATGTGGTGTATA | 60104 | 0.3628017173081421 | No Hit |
| AGCGGCGACTCTGGACGC | 58502 | 0.3531316728663805 | No Hit |
| CTCCGGGGATGCGTGCATTTATCAGATC | 54290 | 0.327707061637479 | No Hit |
| CCCCCCACTGCTAAATTTGACTGGCTT | 53291 | 0.32167686538447027 | No Hit |
| GTGGTTGGCAGCGGCGACTCTGGACGCGTGCC | 52299 | 0.31568892275886007 | No Hit |
| TTTGGCAGGTGAGTAGAGCCGTTCGTGA | 52030 | 0.31406517622026214 | No Hit |
| CAGGTGAGTAGAGCCGTTCGTGA | 50892 | 0.3071959436517697 | No Hit |
| GCGTGTCGGCTGAGGTGGGATCCCGAC | 45275 | 0.2732904257807489 | No Hit |
| CGTGGAGCTTCGGTTGGCCCGGGATAGCCTGCC | 44980 | 0.2715097371975282 | No Hit |
| CAACGGAATCCCAAAAGCAGCT | 44269 | 0.2672179759003418 | No Hit |
| AAATTGATTTTTGGAATAGGGA | 43838 | 0.2646163596990938 | No Hit |
| GGTGAGTAGAGCCGTTCGTGACA | 42486 | 0.2564553733787057 | No Hit |
| TCCCATATGGTCTAGCGGTTAGGATTCC | 42186 | 0.25464450363305746 | No Hit |
| TTGGCAGGTGAGTAGAGCCGTTCGTGACAG | 40606 | 0.24510725630597663 | No Hit |
| TCCCATATGGTCTAGCGGTTAGGATTCCT | 40063 | 0.24182958206635327 | No Hit |
| CAGCGGCGACTCTGGACGCGTGCC | 39603 | 0.23905291512302598 | No Hit |
| GCCCGGCTAGCTCAGTCGGTAGAGCATGAGA | 38670 | 0.2334211102140599 | No Hit |
| CAACGGAATCCCAAAAGCAGCTG | 38501 | 0.23240098692401137 | No Hit |
| GAGGTGTAGAATAAGTGGGAGGCCC | 36333 | 0.21931443489546001 | No Hit |
| TCCTGTACTGAGCTGCCCCGAGA | 31566 | 0.19053971463710925 | No Hit |
| TTCAAGTAATCCAGGATAGGCT | 31287 | 0.18885560577365637 | No Hit |
| AATTGATTTTTGGAATAGGGA | 30849 | 0.18621173594500992 | No Hit |
| CTGATGCGCACCGCATGTTTGTGGAGAACC | 30298 | 0.18288577184550264 | No Hit |
| CTTTTGGCAGGTGAGTAGAGCCGTTCGTGAC | 29903 | 0.18050146001373243 | No Hit |
| GCCCGGATAGCTCAGTCGGTAGAGCATC | 28914 | 0.17453162608557868 | No Hit |
| CACCCGTAGAACCGACCTTGCG | 27950 | 0.1687126979695623 | No Hit |
| TCCCTGGTGGTCTAGTGGTTAGGATTCGG | 27923 | 0.16854971969245397 | No Hit |
| TGAGGTAGTAGATTGTATAGTT | 26118 | 0.15765432005613697 | No Hit |
| TACCCTGTAGAACCGAATTTGT | 23329 | 0.1408192676540937 | No Hit |
| GTGGAGCTTCGGTTGGCCCGGGATAGCCTGCCT | 22838 | 0.13785547750371605 | No Hit |
| TAAATTGATTTTTGGAATAGGGA | 22438 | 0.1354409845095184 | No Hit |
| CGAGCGGGCTCTCGCTTCTGGTTTCAAGCAC | 22250 | 0.1343061728022455 | No Hit |
| TGAGGTAGTAGGTTGTATAGTT | 21900 | 0.1321934914323225 | No Hit |
| TCCTGTACTGAGCTGCCCCGAG | 21502 | 0.12979107090309583 | No Hit |
| TCCTGTACTGAGCTGCCCCGAGT | 20320 | 0.1226562441052417 | No Hit |
| TGGCGGGCACGGGAAATGTGGTGTATA | 19347 | 0.11678298989685587 | No Hit |
| GCAGCGGCGACTCTGGACGC | 19147 | 0.11557574339975704 | No Hit |
| TTAAATTGATTTTTGGAATAGGGA | 18276 | 0.11031818490489162 | No Hit |
| CTGGCGGAGCGCCGAGAAGACGATCAAACTTGA | 18150 | 0.10955761961171935 | No Hit |
| GGCTGGTCCGATGGTAGTGGGTTATCAGAACT | 17591 | 0.1061833656523281 | No Hit |
| CCGAGAAGACGATCAAACTTGAC | 17563 | 0.10601435114273428 | No Hit |
| TGAGAACTGAATTCCATAGGCTGT | 16673 | 0.10064210423064443 | No Hit |

## Adapter Content

Produced by FastQC (version 0.11.9)
