# Supplementary material for: High Throughput Sequencing of MicroRNA in Rainbow Trout Plasma, Mucus, and Surrounding Water Following Acute Stress
Source: Front Physiol. 2021 Jan 13;11:588313. doi: 10.3389/fphys.2020.588313 (PMC7838646; doi:10.3389/fphys.2020.588313)
Supplement: Supplementary file 2 [file Data_Sheet_1.ZIP › Supplemental Quality Control/FastQC_processed_files/mucus_control_2_fastqc_processed.html]

size\_trimmed\_adapterless\_SV18263\_0009\_S21\_R1\_001.fastq FastQC Report 

FastQC Report

Fri 8 May 2020  
size\_trimmed\_adapterless\_SV18263\_0009\_S21\_R1\_001.fastq

## Summary

- Basic Statistics
- Per base sequence quality
- Per tile sequence quality
- Per sequence quality scores
- Per base sequence content
- Per sequence GC content
- Per base N content
- Sequence Length Distribution
- Sequence Duplication Levels
- Overrepresented sequences
- Adapter Content

## Basic Statistics

| Measure | Value |
| --- | --- |
| Filename | size\_trimmed\_adapterless\_SV18263\_0009\_S21\_R1\_001.fastq |
| File type | Conventional base calls |
| Encoding | Sanger / Illumina 1.9 |
| Total Sequences | 11875192 |
| Sequences flagged as poor quality | 0 |
| Sequence length | 18-35 |
| %GC | 55 |

## Per base sequence quality

## Per tile sequence quality

## Per sequence quality scores

## Per base sequence content

## Per sequence GC content

## Per base N content

## Sequence Length Distribution

## Sequence Duplication Levels

## Overrepresented sequences

| Sequence | Count | Percentage | Possible Source |
| --- | --- | --- | --- |
| TGAGAACTGAATTCCATAGATGG | 572716 | 4.822793601989762 | No Hit |
| GCGGCGACTCTGGACGCGTGCC | 326144 | 2.746431384014675 | No Hit |
| CGGCGACTCTGGACGCGTGCC | 261899 | 2.2054296048434416 | No Hit |
| GGCGACTCTGGACGCGTGCC | 144187 | 1.2141866843079254 | No Hit |
| GCATTGGTGGTTCAGTGGTAGAATTCTCGC | 140248 | 1.1810166942985005 | No Hit |
| GCAGCGGCGACTCTGGACGCGTGCC | 111692 | 0.9405490033340091 | No Hit |
| TAACACTGTCTGGTAACGATG | 111458 | 0.9385785088780038 | No Hit |
| TGAGAACTGAATTCCATAGATG | 107790 | 0.9076905872342947 | No Hit |
| AGCGGCGACTCTGGACGCGTGCC | 104695 | 0.8816278507328554 | No Hit |
| GCATTGGTGGTTCAGTGGTAGAATTC | 101819 | 0.8574092949402418 | No Hit |
| CTTTTGGCAGGTGAGTAGAGCCGTTCGTGACA | 94241 | 0.7935955898649892 | No Hit |
| GCGACTCTGGACGCGTGCC | 93551 | 0.7877851574947168 | No Hit |
| GCATTGGTGGTTCAGTGGTAGAATTCTCGCC | 86747 | 0.7304892417739436 | No Hit |
| TGTCAACCGGGTCGGACTGTCCTCAGTGCGTA | 76565 | 0.6447474701882715 | No Hit |
| TTGGCAGGTGAGTAGAGCCGTTCGTGA | 74497 | 0.6273330149104116 | No Hit |
| GAGGTGTAGAATAAGTGGGAGGCCC | 67145 | 0.5654224369593351 | No Hit |
| CCGAGAAGACGATCAAACTTGA | 61732 | 0.5198400160603719 | No Hit |
| AGCGGCGACTCTGGACGC | 60505 | 0.5095075515410613 | No Hit |
| TACCCTGTAGAACCGAATTTGT | 58584 | 0.49333097098556383 | No Hit |
| TCTTTTGGCAGGTGAGTAGAGCCGTTCGTGACA | 55359 | 0.4661735153418993 | No Hit |
| GCATTGGTGGTTCAGTGGTAGAATTCTC | 51950 | 0.43746661106616214 | No Hit |
| AGGTGTAGAATAAGTGGGAGGCCCCG | 45751 | 0.3852653498149756 | No Hit |
| GCCGAGAAGACGATCAAACTTGA | 43812 | 0.36893719276286224 | No Hit |
| CAGGTGAGTAGAGCCGTTCGTGACA | 41648 | 0.3507143295030514 | No Hit |
| AGCGGCGACTCTGGACGCGTGCCGGG | 41472 | 0.349232248202808 | No Hit |
| CTTTTGGCAGGTGAGTAGAGCCGTTCGTGA | 40139 | 0.33800716653676 | No Hit |
| TGTCAACCGGGTCGGACTGTCCTCAGTGCGTAC | 39696 | 0.33427670053671554 | No Hit |
| ATCTCGTGGGCTCTCGTTTGTGG | 38647 | 0.32544315915060573 | No Hit |
| GGTGAGTAGAGCCGTTCGTGACA | 37380 | 0.314773857972149 | No Hit |
| GGCGACTCTGGACGCGTGCCGG | 37219 | 0.3134180904190854 | No Hit |
| GGAATACCAGGTGCTGTAAGCTT | 36449 | 0.3069339847305205 | No Hit |
| AGGTGTAGAATAAGTGGGAGGCCC | 35317 | 0.29740150727668235 | No Hit |
| TCTCGCAAGGGGCTGCTT | 34587 | 0.2912542382472637 | No Hit |
| GCAGCGGCGACTCTGGACGC | 33451 | 0.28168807712751087 | No Hit |
| CAGCGGCGACTCTGGACGCGTGCC | 33320 | 0.28058493706880694 | No Hit |
| GCATTGGTGGTTCAGTGGTAGAATTCTCGCCT | 30673 | 0.2582947711498054 | No Hit |
| TGAGAACTGAATTCCATAGAT | 30438 | 0.25631585577732136 | No Hit |
| TGGCGGGCACGGGAAATGTGGTGTATA | 30414 | 0.2561137537818336 | No Hit |
| TCGAGCCGCGGCTGGGGGAGC | 29920 | 0.2519538210413777 | No Hit |
| TAACGGAACCCATAATGCAGCTG | 29236 | 0.24619391416997718 | No Hit |
| TCCCATATGGTCTAGCGGTTAGGATTCCTG | 28589 | 0.24074558120828699 | No Hit |
| TGTGGTCGGATCCCCTCGTGG | 27856 | 0.23457304942943236 | No Hit |
| TAGCTTATCAGACTGGTGTTGG | 27679 | 0.23308254721271035 | No Hit |
| TGAGAACTGAATTCCATAGATGGT | 27480 | 0.23140678483345783 | No Hit |
| GTGGTTGGCAGCGGCGACTCTGGACGCGTGCC | 27460 | 0.2312383665038847 | No Hit |
| TCTTTTGGCAGGTGAGTAGAGCCGTTCGTGA | 27417 | 0.23087626709530257 | No Hit |
| CTCAGTCGGTAGAGCATC | 26829 | 0.225924768205853 | No Hit |
| TCGAGCCGCGGCTGGGGGAGCAGTT | 26569 | 0.22373532992140252 | No Hit |
| TTGGCAGGTGAGTAGAGCCGTTCGTGACA | 25750 | 0.21683859932538352 | No Hit |
| TAACACTGTCTGGTAATGATG | 25316 | 0.213183921573647 | No Hit |
| GTCTGGCGGGCACGGGAAATGTGGTGTATA | 24829 | 0.20908293524854166 | No Hit |
| GAGGTGTAGAATAAGTGGGAGGCCCCG | 24792 | 0.20877136133883142 | No Hit |
| TCCCTGGTGGTCTAGTGGTTAGGATTCGGC | 24718 | 0.2081482135194109 | No Hit |
| TTTTGGCAGGTGAGTAGAGCCGTTCGTGACA | 24584 | 0.20701981071127104 | No Hit |
| GGGGAATTAGCTCAAATGGTAGA | 23693 | 0.19951677412878882 | No Hit |
| TCGGGCTGGGGTGCGAAGCGGGGCT | 21887 | 0.18430859896833668 | No Hit |
| TCCCATATGGTCTAGCGGTTAGGATTCCT | 21268 | 0.17909605166804882 | No Hit |
| GTGGTTGGCAGCGGCGACTCTGGACGC | 20728 | 0.17454875676957476 | No Hit |
| TAATACTGCCTGGTAATGATGA | 20725 | 0.17452349402013878 | No Hit |
| CGCGTGTCGGCTGAGGTGGGATCCCG | 20671 | 0.17406876453029138 | No Hit |
| CGAGAAGACGATCAAACTTGA | 20265 | 0.1706498724399572 | No Hit |
| AACCCGTAGATCCGAACTTGTG | 20131 | 0.16952146963181733 | No Hit |
| GCATTGGTGGTTCAGTGGTAGAATTCTCG | 20127 | 0.1694877859659027 | No Hit |
| CGACTCTGGACGCGTGCC | 19852 | 0.16717203393427238 | No Hit |
| AACCCGTAGATCCGAACTTGT | 19055 | 0.16046056350078383 | No Hit |
| GCCTGTGAATAGACGCTGTAG | 18500 | 0.15578695485512992 | No Hit |
| TTCAAGTAATCCAGGATAGGCT | 18069 | 0.15215753985282934 | No Hit |
| GATCGGGGGCCTGAGTCCT | 17823 | 0.15008599439908002 | No Hit |
| TAGCTTATCAGACTGGTGTTGGC | 17683 | 0.14890706609206825 | No Hit |
| GGTTGGCAGCGGCGACTCTGGACGCGTGCC | 17343 | 0.1460439544893253 | No Hit |
| GAGCCGCGGCTGGGGGAGC | 17286 | 0.14556396225004195 | No Hit |
| CATCTCGTGGGCTCTCGTTTGTGG | 17040 | 0.14349241679629263 | No Hit |
| GCGGCGACTCTGGACGCGTGCCG | 16725 | 0.1408398281055161 | No Hit |
| GTTTCCGTAGTGTAGTGGTTATCACGTTC | 16533 | 0.1392230121416142 | No Hit |
| CAGGTGAGTAGAGCCGTTCGTGA | 16375 | 0.13789250733798664 | No Hit |
| TGAAATGTTTAGGACCACTCG | 16295 | 0.13721883401969417 | No Hit |
| CTTTTGGCAGGTGAGTAGAGCCGTTCGTGAC | 16229 | 0.1366630535321029 | No Hit |
| AGCGGCGACTCTGGACGCGTGCCG | 16061 | 0.13524833956368873 | No Hit |
| AGCGGCGACTCTGGACGCGTGCCGG | 16036 | 0.13503781665172235 | No Hit |
| GCGGCGACTCTGGACGCGTGCCGG | 15791 | 0.1329746921144517 | No Hit |
| TGTGGGCACTCGAAGATA | 15442 | 0.13003579226340087 | No Hit |
| TTTTGGCAGGTGAGTAGAGCCGTTCGTGA | 15421 | 0.1298589530173491 | No Hit |
| TGAGAACTGAATTCCATAGA | 14576 | 0.12274327859288507 | No Hit |
| CTAATGGATAAGGCACTG | 14506 | 0.12215381443937917 | No Hit |
| TCGAGCCGCGGCTGGGGGAGCAGTTG | 14328 | 0.12065489130617846 | No Hit |
| CTCAGTCGGTAGAGCATCA | 14051 | 0.11832229744159084 | No Hit |
| TAACGGAACCCATAAAGCAGCTG | 13481 | 0.1135223750487571 | No Hit |
| GCCACTGCTGGAAGTTCG | 13074 | 0.11009506204194425 | No Hit |
| GCAGCGGCGACTCTGGACGCGTGCCGG | 12890 | 0.10854561340987161 | No Hit |
| GCCCGGCTAGCTCAGTCGGTAGAGCATGA | 12466 | 0.1049751448229216 | No Hit |
| GGACTGTCCTCAGTGCGTA | 12431 | 0.10468041274616866 | No Hit |
| CCCGAAAGATGGTGAACTATGC | 12210 | 0.10281939020438575 | No Hit |
| TTGTGCGGCCCATCTCGTGG | 12187 | 0.10262570912537668 | No Hit |
| TAATACTGCCTGGTAATGATGAT | 12029 | 0.10129520432174907 | No Hit |

## Adapter Content

Produced by FastQC (version 0.11.9)
