# Supplementary material for: High Throughput Sequencing of MicroRNA in Rainbow Trout Plasma, Mucus, and Surrounding Water Following Acute Stress
Source: Front Physiol. 2021 Jan 13;11:588313. doi: 10.3389/fphys.2020.588313 (PMC7838646; doi:10.3389/fphys.2020.588313)
Supplement: Supplementary file 2 [file Data_Sheet_1.ZIP › Supplemental Quality Control/FastQC_processed_files/mucus_control_3_fastqc_processed.html]

size\_trimmed\_adapterless\_SV18263\_0011\_S23\_R1\_001.fastq FastQC Report 

FastQC Report

Fri 8 May 2020  
size\_trimmed\_adapterless\_SV18263\_0011\_S23\_R1\_001.fastq

## Summary

- Basic Statistics
- Per base sequence quality
- Per tile sequence quality
- Per sequence quality scores
- Per base sequence content
- Per sequence GC content
- Per base N content
- Sequence Length Distribution
- Sequence Duplication Levels
- Overrepresented sequences
- Adapter Content

## Basic Statistics

| Measure | Value |
| --- | --- |
| Filename | size\_trimmed\_adapterless\_SV18263\_0011\_S23\_R1\_001.fastq |
| File type | Conventional base calls |
| Encoding | Sanger / Illumina 1.9 |
| Total Sequences | 16029580 |
| Sequences flagged as poor quality | 0 |
| Sequence length | 18-35 |
| %GC | 57 |

## Per base sequence quality

## Per tile sequence quality

## Per sequence quality scores

## Per base sequence content

## Per sequence GC content

## Per base N content

## Sequence Length Distribution

## Sequence Duplication Levels

## Overrepresented sequences

| Sequence | Count | Percentage | Possible Source |
| --- | --- | --- | --- |
| CTTTTGGCAGGTGAGTAGAGCCGTTCGTGACA | 1450615 | 9.04961327745331 | No Hit |
| CCGAGAAGACGATCAAACTTGA | 1210678 | 7.552774308497166 | No Hit |
| AGCGGCGACTCTGGACGCGTGCC | 837577 | 5.225196168583332 | No Hit |
| GCGGCGACTCTGGACGCGTGCC | 704531 | 4.3951931366885475 | No Hit |
| CGGCGACTCTGGACGCGTGCC | 631513 | 3.939672779948071 | No Hit |
| GCATTGGTGGTTCAGTGGTAGAATTCTCGCC | 586397 | 3.6582181192520333 | No Hit |
| GCAGCGGCGACTCTGGACGCGTGCC | 448324 | 2.796854315584064 | No Hit |
| GCATTGGTGGTTCAGTGGTAGAATTCTCGC | 433539 | 2.7046185863884147 | No Hit |
| TTGGCAGGTGAGTAGAGCCGTTCGTGACA | 392559 | 2.4489662236939456 | No Hit |
| GGCGACTCTGGACGCGTGCC | 365808 | 2.2820810027461733 | No Hit |
| TGAGAACTGAATTCCATAGATGG | 279895 | 1.7461156187498363 | No Hit |
| GTGGTTGGCAGCGGCGACTCTGGACGCGTGCC | 229287 | 1.4303992992954275 | No Hit |
| GCCGAGAAGACGATCAAACTTGA | 219142 | 1.3671100552852913 | No Hit |
| TTTTGGCAGGTGAGTAGAGCCGTTCGTGACA | 158925 | 0.991448309937004 | No Hit |
| CTTTTGGCAGGTGAGTAGAGCCGTTCGTGA | 158197 | 0.9869067062268632 | No Hit |
| CTTTTGGCAGGTGAGTAGAGCCGTTCGTGACAG | 154622 | 0.9646041880074212 | No Hit |
| CGAGAAGACGATCAAACTTGA | 120748 | 0.7532823692199048 | No Hit |
| GCATTGGTGGTTCAGTGGTAGAATTCTCGCCT | 111940 | 0.6983339551005079 | No Hit |
| TTGGCAGGTGAGTAGAGCCGTTCGTGA | 108907 | 0.6794126857971325 | No Hit |
| GCATTGGTGGTTCAGTGGTAGAATTC | 104518 | 0.6520320557369563 | No Hit |
| CAGCGGCGACTCTGGACGCGTGCC | 85831 | 0.5354538297322824 | No Hit |
| AGCGGCGACTCTGGACGC | 69238 | 0.4319389528608984 | No Hit |
| GCATTGGTGGTTCAGTGGTAGAATTCTC | 67689 | 0.42227556804357946 | No Hit |
| CCGAGAAGACGATCAAACTTGAC | 66840 | 0.4169791098706267 | No Hit |
| CAGGTGAGTAGAGCCGTTCGTGACA | 66025 | 0.4118947595632574 | No Hit |
| CCGAGAAGACGATCAAACTTGACTAT | 64527 | 0.4025495365443137 | No Hit |
| GGTTGGCAGCGGCGACTCTGGACGCGTGCC | 63552 | 0.39646703157537505 | No Hit |
| GCGTGTCGGCTGAGGTGGGATCCCGAC | 56798 | 0.3543324279238757 | No Hit |
| GGAATACCAGGTGCTGTAAGCTT | 53055 | 0.330981847309786 | No Hit |
| GATCGGGGGCCTGAGTCCT | 52168 | 0.3254483274047106 | No Hit |
| CGAGAAGACGATCAAACTTGAC | 45237 | 0.28220951515885007 | No Hit |
| CTTTTGGCAGGTGAGTAGAGCCGTTCGTGAC | 44825 | 0.2796392669053088 | No Hit |
| CGAGAAGACGATCAAACTTGACTAT | 44153 | 0.2754470173267172 | No Hit |
| GCATTGGTGGTTCAGTGGTAGAATTCTCG | 42215 | 0.26335686898845756 | No Hit |
| GCAGCGGCGACTCTGGACGC | 42110 | 0.2627018299918027 | No Hit |
| AGCGGCGACTCTGGACGCGTGCCG | 41588 | 0.2594453504084324 | No Hit |
| GGTGAGTAGAGCCGTTCGTGACA | 41061 | 0.2561576784918881 | No Hit |
| CGTGGAGCTTCGGTTGGCCCGGGATAGCCTGCCT | 37804 | 0.23583899266231556 | No Hit |
| TCGGGCTGGGGTGCGAAGC | 35915 | 0.22405452918916155 | No Hit |
| TCTTTTGGCAGGTGAGTAGAGCCGTTCGTGACA | 35427 | 0.22101015747137479 | No Hit |
| TGGGAATACCAGGTGCTGTAAGCTT | 32048 | 0.19993037871235553 | No Hit |
| TTTTGGCAGGTGAGTAGAGCCGTTCGTGA | 30598 | 0.19088460209188265 | No Hit |
| GCGGCGACTCTGGACGCGTGCCG | 28427 | 0.1773408910277125 | No Hit |
| GCAGCGGCGACTCTGGACGCGTG | 27790 | 0.17336698778133924 | No Hit |
| GCGACTCTGGACGCGTGCC | 25151 | 0.15690367433207858 | No Hit |
| GTGTCCGTCGGCGTCCCGAAGGTGGATC | 24391 | 0.1521624396896238 | No Hit |
| TTTGGCAGGTGAGTAGAGCCGTTCGTGACA | 23562 | 0.14699075084936725 | No Hit |
| GGCGGCGACTCTGGACGCGTGCC | 22778 | 0.14209979300767706 | No Hit |
| CCGAGAAGACGATCAAACT | 22194 | 0.13845652849294865 | No Hit |
| CGAGCGGGCTCTCGCTTCTGGTTTCAAGCAC | 21848 | 0.1362980190373048 | No Hit |
| CTGGCGGAGCGCCGAGAAGACGATCAAACTTGA | 21548 | 0.1344264790468621 | No Hit |
| GCCCGGCTAGCTCAGTCGGTAGAGCATGAGA | 21375 | 0.13334722431904017 | No Hit |
| GCAGCGGCGACTCTGGACGCGTGC | 21347 | 0.13317254725326552 | No Hit |
| CCTGGCGGAGCGCCGAGAAGACGATCAAACTTGA | 20962 | 0.1307707375988641 | No Hit |
| AGCGGCGACTCTGGACGCGTGC | 19950 | 0.1244574093644375 | No Hit |
| CCGAGAAGACGATCAAACTTGT | 19620 | 0.12239871537495058 | No Hit |
| GAGAAGACGATCAAACTTGA | 19434 | 0.1212383605808761 | No Hit |
| TACCCTGTAGAACCGAATTTGT | 19219 | 0.11989709025439219 | No Hit |
| CCGAGAAGACGATCAAACTTG | 18741 | 0.11691510320295355 | No Hit |
| TGGTTGGCAGCGGCGACTCTGGACGCGTGCC | 18383 | 0.11468173214769196 | No Hit |
| TTGGCAGGTGAGTAGAGCCGTTCGTGACAG | 17597 | 0.10977829737273218 | No Hit |
| GTGGTTGGCAGCGGCGACTCTGGACGC | 16671 | 0.10400147726889912 | No Hit |
| CCGAGAAGACGATCAAAC | 16583 | 0.10345249220503594 | No Hit |
| GTGGTTGGCAGCGGCGACTCTGGACGCGTGC | 16460 | 0.10268516080895446 | No Hit |

## Adapter Content

Produced by FastQC (version 0.11.9)
