# Supplementary material for: High Throughput Sequencing of MicroRNA in Rainbow Trout Plasma, Mucus, and Surrounding Water Following Acute Stress
Source: Front Physiol. 2021 Jan 13;11:588313. doi: 10.3389/fphys.2020.588313 (PMC7838646; doi:10.3389/fphys.2020.588313)
Supplement: Supplementary file 2 [file Data_Sheet_1.ZIP › Supplemental Quality Control/FastQC_processed_files/mucus_stressed_1_fastqc_processed.html]

size\_trimmed\_adapterless\_SV18263\_0008\_S20\_R1\_001.fastq FastQC Report 

FastQC Report

Fri 8 May 2020  
size\_trimmed\_adapterless\_SV18263\_0008\_S20\_R1\_001.fastq

## Summary

- Basic Statistics
- Per base sequence quality
- Per tile sequence quality
- Per sequence quality scores
- Per base sequence content
- Per sequence GC content
- Per base N content
- Sequence Length Distribution
- Sequence Duplication Levels
- Overrepresented sequences
- Adapter Content

## Basic Statistics

| Measure | Value |
| --- | --- |
| Filename | size\_trimmed\_adapterless\_SV18263\_0008\_S20\_R1\_001.fastq |
| File type | Conventional base calls |
| Encoding | Sanger / Illumina 1.9 |
| Total Sequences | 18576882 |
| Sequences flagged as poor quality | 0 |
| Sequence length | 18-35 |
| %GC | 58 |

## Per base sequence quality

## Per tile sequence quality

## Per sequence quality scores

## Per base sequence content

## Per sequence GC content

## Per base N content

## Sequence Length Distribution

## Sequence Duplication Levels

## Overrepresented sequences

| Sequence | Count | Percentage | Possible Source |
| --- | --- | --- | --- |
| CCGAGAAGACGATCAAACTTGA | 2401989 | 12.929990081220305 | No Hit |
| AGCGGCGACTCTGGACGCGTGCC | 1713953 | 9.226268434067677 | No Hit |
| GCGGCGACTCTGGACGCGTGCC | 1190874 | 6.410516038159687 | No Hit |
| GCAGCGGCGACTCTGGACGCGTGCC | 907613 | 4.885712252465187 | No Hit |
| CGGCGACTCTGGACGCGTGCC | 812757 | 4.375099115125994 | No Hit |
| GGCGACTCTGGACGCGTGCC | 571180 | 3.0746817469153327 | No Hit |
| GCATTGGTGGTTCAGTGGTAGAATTCTCGCC | 446966 | 2.4060334775233003 | No Hit |
| TGAGAACTGAATTCCATAGATGG | 440031 | 2.3687021320370127 | No Hit |
| CGAGAAGACGATCAAACTTGA | 386664 | 2.0814257204196056 | No Hit |
| GCATTGGTGGTTCAGTGGTAGAATTCTCGC | 295526 | 1.5908267060101906 | No Hit |
| GTGGTTGGCAGCGGCGACTCTGGACGCGTGCC | 273087 | 1.4700367908888048 | No Hit |
| GCCGAGAAGACGATCAAACTTGA | 258913 | 1.3937376573743645 | No Hit |
| CTTTTGGCAGGTGAGTAGAGCCGTTCGTGACA | 252906 | 1.3614017680685058 | No Hit |
| CAGCGGCGACTCTGGACGCGTGCC | 168662 | 0.907913394723614 | No Hit |
| CCGAGAAGACGATCAAACTTGAC | 166202 | 0.8946711294177354 | No Hit |
| TTGGCAGGTGAGTAGAGCCGTTCGTGACA | 155330 | 0.8361467764073647 | No Hit |
| CCGAGAAGACGATCAAACTTGACTAT | 140354 | 0.7555304490818211 | No Hit |
| CGAGAAGACGATCAAACTTGAC | 130305 | 0.701436333610775 | No Hit |
| GGTTGGCAGCGGCGACTCTGGACGCGTGCC | 124711 | 0.6713236376265942 | No Hit |
| GCATTGGTGGTTCAGTGGTAGAATTCTCGCCT | 107575 | 0.5790799553983279 | No Hit |
| CGAGAAGACGATCAAACTTGACTAT | 104485 | 0.5624463782458219 | No Hit |
| CCGAGAAGACGATCAAACT | 97652 | 0.5256641022966071 | No Hit |
| AGCGGCGACTCTGGACGCGTGCCG | 81108 | 0.43660717659723525 | No Hit |
| GCATTGGTGGTTCAGTGGTAGAATTC | 77536 | 0.41737897673032537 | No Hit |
| AGCGGCGACTCTGGACGC | 75452 | 0.4061607324630689 | No Hit |
| CCGAGAAGACGATCAAAC | 69644 | 0.37489606705797024 | No Hit |
| CGAGAAGACGATCAAACT | 57881 | 0.3115754301502265 | No Hit |
| GGCGGCGACTCTGGACGCGTGCC | 54564 | 0.2937199041259992 | No Hit |
| GCGGCGACTCTGGACGCGTGCCG | 46232 | 0.24886845919568204 | No Hit |
| AGCGGCGACTCTGGACGCGTGC | 45782 | 0.24644609359094813 | No Hit |
| GCAGCGGCGACTCTGGACGCGTG | 44466 | 0.23936201995577083 | No Hit |
| TTGGCAGGTGAGTAGAGCCGTTCGTGA | 44213 | 0.23800011218244269 | No Hit |
| GGAATACCAGGTGCTGTAAGCTT | 43525 | 0.23429658432453843 | No Hit |
| GCAGCGGCGACTCTGGACGCGTGC | 43093 | 0.23197111334399392 | No Hit |
| TCGGGCTGGGGTGCGAAGC | 38269 | 0.20600335406124665 | No Hit |
| GCAGCGGCGACTCTGGACGC | 38149 | 0.20535738989998428 | No Hit |
| TTTTGGCAGGTGAGTAGAGCCGTTCGTGACA | 34221 | 0.1842128296879961 | No Hit |
| GAGAAGACGATCAAACTTGA | 33464 | 0.18013787243736598 | No Hit |
| CCGAGAAGACGATCAAACTTGT | 32898 | 0.17709107481007846 | No Hit |
| GCATTGGTGGTTCAGTGGTAGAATTCTC | 31197 | 0.16793453282418438 | No Hit |
| CTTTTGGCAGGTGAGTAGAGCCGTTCGTGACAG | 30374 | 0.16350429528485996 | No Hit |
| GCGTGTCGGCTGAGGTGGGATCCCGAC | 29718 | 0.15997302453662568 | No Hit |
| CTTTTGGCAGGTGAGTAGAGCCGTTCGTGA | 27931 | 0.15035354156849356 | No Hit |
| CCGAGAAGACGATCAAACTTG | 25167 | 0.13547483372075034 | No Hit |
| CAGCGGCGACTCTGGACGCGTGC | 24849 | 0.13376302869340506 | No Hit |
| TGGTTGGCAGCGGCGACTCTGGACGCGTGCC | 24767 | 0.13332161984987578 | No Hit |
| GCGACTCTGGACGCGTGCC | 23580 | 0.1269319576880555 | No Hit |
| TGGGAATACCAGGTGCTGTAAGCTT | 22906 | 0.12330379231563188 | No Hit |
| GCATTGGTGGTTCAGTGGTAGAATTCTCG | 20886 | 0.11243006226771533 | No Hit |
| GTGTCCGTCGGCGTCCCGAAGGTGGATC | 19858 | 0.1068963026195677 | No Hit |
| CAGGTGAGTAGAGCCGTTCGTGACA | 19269 | 0.1037256951947049 | No Hit |
| GGTGAGTAGAGCCGTTCGTGACA | 19009 | 0.10232610617863644 | No Hit |
| AGGATTGGCTCTAAGGGC | 18670 | 0.10050125742307026 | No Hit |

## Adapter Content

Produced by FastQC (version 0.11.9)
