# Supplementary material for: High Throughput Sequencing of MicroRNA in Rainbow Trout Plasma, Mucus, and Surrounding Water Following Acute Stress
Source: Front Physiol. 2021 Jan 13;11:588313. doi: 10.3389/fphys.2020.588313 (PMC7838646; doi:10.3389/fphys.2020.588313)
Supplement: Supplementary file 2 [file Data_Sheet_1.ZIP › Supplemental Quality Control/FastQC_processed_files/mucus_stressed_2_fastqc_processed.html]

size\_trimmed\_adapterless\_SV18263\_0010\_S22\_R1\_001.fastq FastQC Report 

FastQC Report

Fri 8 May 2020  
size\_trimmed\_adapterless\_SV18263\_0010\_S22\_R1\_001.fastq

## Summary

- Basic Statistics
- Per base sequence quality
- Per tile sequence quality
- Per sequence quality scores
- Per base sequence content
- Per sequence GC content
- Per base N content
- Sequence Length Distribution
- Sequence Duplication Levels
- Overrepresented sequences
- Adapter Content

## Basic Statistics

| Measure | Value |
| --- | --- |
| Filename | size\_trimmed\_adapterless\_SV18263\_0010\_S22\_R1\_001.fastq |
| File type | Conventional base calls |
| Encoding | Sanger / Illumina 1.9 |
| Total Sequences | 12601098 |
| Sequences flagged as poor quality | 0 |
| Sequence length | 18-35 |
| %GC | 52 |

## Per base sequence quality

## Per tile sequence quality

## Per sequence quality scores

## Per base sequence content

## Per sequence GC content

## Per base N content

## Sequence Length Distribution

## Sequence Duplication Levels

## Overrepresented sequences

| Sequence | Count | Percentage | Possible Source |
| --- | --- | --- | --- |
| CCGAGAAGACGATCAAACTTGA | 2068632 | 16.416283723846924 | No Hit |
| GAGGTGTAGAATAAGTGGGAGGCCC | 736200 | 5.842348023957912 | No Hit |
| AGGTGTAGAATAAGTGGGAGGCCC | 709576 | 5.631064848475903 | No Hit |
| TTGGCAGGTGAGTAGAGCCGTTCGTGA | 551665 | 4.377912147020839 | No Hit |
| GCCGAGAAGACGATCAAACTTGA | 400876 | 3.181278329872524 | No Hit |
| TTGGCAGGTGAGTAGAGCCGTTCGTGACA | 241839 | 1.91918989916593 | No Hit |
| GCATTGGTGGTTCAGTGGTAGAATTCTCGCC | 184763 | 1.4662452430732622 | No Hit |
| GCATTGGTGGTTCAGTGGTAGAATTCTCGC | 160791 | 1.276007852649031 | No Hit |
| TGAGAACTGAATTCCATAGATGG | 158113 | 1.2547557363651962 | No Hit |
| AGCGGCGACTCTGGACGCGTGCC | 137753 | 1.0931825147300656 | No Hit |
| TTTTGGCAGGTGAGTAGAGCCGTTCGTGA | 136335 | 1.0819295270935914 | No Hit |
| GCGGCGACTCTGGACGCGTGCC | 127490 | 1.0117372311523964 | No Hit |
| CGGCGACTCTGGACGCGTGCC | 99779 | 0.7918278232579414 | No Hit |
| GGCGACTCTGGACGCGTGCC | 99479 | 0.7894470783419032 | No Hit |
| GATCGGGGGCCTGAGTCCT | 88081 | 0.6989946431652225 | No Hit |
| CCGAGAAGACGATCAAACTTG | 76145 | 0.6042727387724467 | No Hit |
| CCGAGAAGACGATCAAACTT | 73757 | 0.585322009240782 | No Hit |
| GCAGCGGCGACTCTGGACGCGTGCC | 69595 | 0.5522931414389445 | No Hit |
| CTTTTGGCAGGTGAGTAGAGCCGTTCGTGA | 64241 | 0.5098047805040481 | No Hit |
| CCGAGAAGACGATCAAACTTGAC | 58830 | 0.4668640780351046 | No Hit |
| GTGGTTGGCAGCGGCGACTCTGGACGCGTGCC | 51458 | 0.40836123963165744 | No Hit |
| TTTTGGCAGGTGAGTAGAGCCGTTCGTGACA | 51175 | 0.40611540359419473 | No Hit |
| TTGGCAGGTGAGTAGAGCCGTTCGT | 47257 | 0.37502287499073494 | No Hit |
| TTTGGCAGGTGAGTAGAGCCGTTCGTGA | 46355 | 0.36786476860984657 | No Hit |
| GCGTGTCGGCTGAGGTGGGATCCCG | 43919 | 0.3485331198916158 | No Hit |
| AGGTGAGTAGAGCCGTTCGTGACA | 42648 | 0.33844669726400034 | No Hit |
| AGGTGAGTAGAGCCGTTCGTGA | 42592 | 0.3380022915463399 | No Hit |
| GCATTGGTGGTTCAGTGGTAGAATTC | 34185 | 0.27128588318256075 | No Hit |
| TGATGCGCACCGCATGTTTGTGGAGAACC | 32490 | 0.2578346744069445 | No Hit |
| GCATTGGTGGTTCAGTGGTAGAATTCTCGCCT | 31234 | 0.24786728902513097 | No Hit |
| CTTTTGGCAGGTGAGTAGAGCCGTTCGTGACA | 31047 | 0.24638329136080048 | No Hit |
| GGTTGGCAGCGGCGACTCTGGACGCGTGCC | 27687 | 0.21971894830117183 | No Hit |
| TGTGGTCGGATCCCCTCGTGG | 27375 | 0.21724297358849204 | No Hit |
| TTGGCAGGTGAGTAGAGCCGTTCGTGAC | 26859 | 0.21314809233290624 | No Hit |
| GATCGGCTCACGTAAACTGGC | 26236 | 0.20820407872393343 | No Hit |
| TCTTTTGGCAGGTGAGTAGAGCCGTTCGTGA | 24542 | 0.194760805764704 | No Hit |
| AAATTGATTTTTGGAATAGGGA | 24044 | 0.1908087692040805 | No Hit |
| GCATTGGTGGTTCAGTGGTAGAATTCTC | 23902 | 0.1896818832771557 | No Hit |
| TGGGAATACCAGGTGCTGTAAGCTT | 23750 | 0.18847563918636295 | No Hit |
| CCGCCGGTGAAATACCACTACTCTTAT | 22166 | 0.1759053060296809 | No Hit |
| ATCGGGGGCCTGAGTCCT | 21964 | 0.1743022711195485 | No Hit |
| TTGGCAGGTGAGTAGAGCCGTTC | 21161 | 0.1679298105609527 | No Hit |
| CGAGAAGACGATCAAACTTGA | 20902 | 0.165874434116773 | No Hit |
| AATTGATTTTTGGAATAGGGA | 19421 | 0.15412149004793074 | No Hit |
| TTTGGCAGGTGAGTAGAGCCGTTCGTGACA | 19399 | 0.15394690208742126 | No Hit |
| GGAATACCAGGTGCTGTAAGCTT | 19216 | 0.15249464768863794 | No Hit |
| CTCCGGGGATGCGTGCATTTATCAGATC | 19102 | 0.1515899646205434 | No Hit |
| CCGAGAAGACGATCAAACT | 18811 | 0.14928064205198627 | No Hit |
| CCGAGAAGACGATCAAACTTGT | 18645 | 0.14796329653177842 | No Hit |
| CAGCGGCGACTCTGGACGCGTGCC | 18427 | 0.14623328855945728 | No Hit |
| CCGAGAAGACGATCAAACTTGACTAT | 18345 | 0.14558255161574016 | No Hit |
| TACCCTGTAGAACCGAATTTGT | 18253 | 0.1448524565081551 | No Hit |
| CGTTTTTTCACTTACCCGGTGAGGCGGGGAG | 17923 | 0.14223363710051298 | No Hit |
| CCGCCGGTGAAATACCACTACTCTTA | 17314 | 0.13740072492095529 | No Hit |
| CGAGAAGACGATCAAACTTGAC | 16840 | 0.13363914795361484 | No Hit |
| AGCGGCGACTCTGGACGCGTGCCG | 16771 | 0.13309157662292603 | No Hit |
| GCGGCGACTCTGGACGCGTGCCG | 16662 | 0.13222657263676546 | No Hit |
| ATGCGCACCGCATGTTTGTGGAGAACC | 16172 | 0.12833802260723629 | No Hit |
| TTGGCAGCGGCGACTCTGGACGCGTGC | 15900 | 0.12617948055002826 | No Hit |
| GCATTGGTGGTTCAGTGGTAGAATTCTCG | 15727 | 0.12480658431511286 | No Hit |
| TGATGCGCACCGCATGTTTGTGGAGAAC | 15675 | 0.12439392186299955 | No Hit |
| TGCGCACCGCATGTTTGTGGAGAAC | 15325 | 0.12161638612762159 | No Hit |
| GTGAGGTCCTCGGATCGGCC | 14986 | 0.11892614437249832 | No Hit |
| CGTGGAGCTTCGGTTGGCCCGGGATAGCCTGCC | 14884 | 0.11811669110104532 | No Hit |
| GAGGTGTAGAATAAGTGGGAGGCCCCG | 14312 | 0.11357740412779901 | No Hit |
| GGACTGTCCTCAGTGCGTA | 14257 | 0.11314093422652534 | No Hit |
| TTGGCAGGTGAGTAGAGCCGTTCGTG | 14166 | 0.11241877493532706 | No Hit |
| TGGCAGCGGCGACTCTGGACGCGTGC | 14060 | 0.11157757839832688 | No Hit |
| AGCGGCGACTCTGGACGC | 13518 | 0.1072763659166844 | No Hit |
| CGTGGAGCTTCGGTTGGCCCGGGATAGCCTGCCT | 13327 | 0.1057606249868067 | No Hit |
| TGAGAACTGAATTCCATAGATG | 13283 | 0.10541144906578775 | No Hit |
| TTTTGGCAGGTGAGTAGAGCCGTTCGT | 13092 | 0.10389570813591005 | No Hit |

## Adapter Content

Produced by FastQC (version 0.11.9)
