# Supplementary material for: High Throughput Sequencing of MicroRNA in Rainbow Trout Plasma, Mucus, and Surrounding Water Following Acute Stress
Source: Front Physiol. 2021 Jan 13;11:588313. doi: 10.3389/fphys.2020.588313 (PMC7838646; doi:10.3389/fphys.2020.588313)
Supplement: Supplementary file 2 [file Data_Sheet_1.ZIP › Supplemental Quality Control/FastQC_processed_files/mucus_stressed_3_fastqc_processed.html]

size\_trimmed\_adapterless\_SV18263\_0012\_S24\_R1\_001.fastq FastQC Report 

FastQC Report

Fri 8 May 2020  
size\_trimmed\_adapterless\_SV18263\_0012\_S24\_R1\_001.fastq

## Summary

- Basic Statistics
- Per base sequence quality
- Per tile sequence quality
- Per sequence quality scores
- Per base sequence content
- Per sequence GC content
- Per base N content
- Sequence Length Distribution
- Sequence Duplication Levels
- Overrepresented sequences
- Adapter Content

## Basic Statistics

| Measure | Value |
| --- | --- |
| Filename | size\_trimmed\_adapterless\_SV18263\_0012\_S24\_R1\_001.fastq |
| File type | Conventional base calls |
| Encoding | Sanger / Illumina 1.9 |
| Total Sequences | 19528092 |
| Sequences flagged as poor quality | 0 |
| Sequence length | 18-35 |
| %GC | 52 |

## Per base sequence quality

## Per tile sequence quality

## Per sequence quality scores

## Per base sequence content

## Per sequence GC content

## Per base N content

## Sequence Length Distribution

## Sequence Duplication Levels

## Overrepresented sequences

| Sequence | Count | Percentage | Possible Source |
| --- | --- | --- | --- |
| GCATTGGTGGTTCAGTGGTAGAATTCTCGCC | 1856081 | 9.504671526537258 | No Hit |
| GCATTGGTGGTTCAGTGGTAGAATTCTCGC | 1582556 | 8.103997052041747 | No Hit |
| CCGAGAAGACGATCAAACTTGA | 1139715 | 5.836284466500874 | No Hit |
| CTTTTGGCAGGTGAGTAGAGCCGTTCGTGACA | 945939 | 4.843990902951502 | No Hit |
| CGAGAAGACGATCAAACTTGA | 834382 | 4.2727266954703 | No Hit |
| GCATTGGTGGTTCAGTGGTAGAATTCTCGCCT | 537261 | 2.751221163849494 | No Hit |
| TGAGAACTGAATTCCATAGATGG | 500385 | 2.562385511088334 | No Hit |
| AGCGGCGACTCTGGACGCGTGCC | 379245 | 1.9420484090304366 | No Hit |
| CGAGAAGACGATCAAACTTGACTAT | 336482 | 1.7230664419237682 | No Hit |
| CGAGAAGACGATCAAACTTGAC | 325091 | 1.6647350903508649 | No Hit |
| CCGAGAAGACGATCAAACTTGACTAT | 294610 | 1.5086471325514033 | No Hit |
| TTGGCAGGTGAGTAGAGCCGTTCGTGACA | 234331 | 1.1999687424659817 | No Hit |
| GCGGCGACTCTGGACGCGTGCC | 225206 | 1.153241187106247 | No Hit |
| CCGAGAAGACGATCAAACTTGAC | 205385 | 1.0517412556229253 | No Hit |
| GCATTGGTGGTTCAGTGGTAGAATTC | 200493 | 1.0266901651221225 | No Hit |
| GGAATACCAGGTGCTGTAAGCTT | 195340 | 1.0003025385173319 | No Hit |
| GCATTGGTGGTTCAGTGGTAGAATTCTC | 192281 | 0.9846379257123533 | No Hit |
| GCATTGGTGGTTCAGTGGTAGAATTCTCG | 188062 | 0.9630331524452055 | No Hit |
| GCAGCGGCGACTCTGGACGCGTGCC | 179745 | 0.9204432260970504 | No Hit |
| GCCGAGAAGACGATCAAACTTGA | 179723 | 0.9203305678813886 | No Hit |
| GAGAAGACGATCAAACTTGA | 135309 | 0.6928941137720981 | No Hit |
| GTGGTTGGCAGCGGCGACTCTGGACGCGTGCC | 126987 | 0.6502785832840198 | No Hit |
| TACCCTGTAGAACCGAATTTGT | 126795 | 0.6492953843109711 | No Hit |
| CGGCGACTCTGGACGCGTGCC | 124071 | 0.635346248880843 | No Hit |
| GGCGACTCTGGACGCGTGCC | 102664 | 0.5257246842139007 | No Hit |
| CGAGAAGACGATCAAACT | 95879 | 0.4909798663381963 | No Hit |
| CCGAGAAGACGATCAAACT | 92893 | 0.47568907397609556 | No Hit |
| GGTGAGTAGAGCCGTTCGTGACA | 87276 | 0.44692538318643726 | No Hit |
| CTGGCGGAGCGCCGAGAAGACGATCAAAC | 84983 | 0.4351833246176841 | No Hit |
| CAGGTGAGTAGAGCCGTTCGTGACA | 79366 | 0.4064196338280258 | No Hit |
| TTTTGGCAGGTGAGTAGAGCCGTTCGTGACA | 78539 | 0.402184709084738 | No Hit |
| CTTTTGGCAGGTGAGTAGAGCCGTTCGTGACAG | 77567 | 0.3972072642836791 | No Hit |
| AGCGGCGACTCTGGACGC | 66114 | 0.3385584213757289 | No Hit |
| CCGAGAAGACGATCAAAC | 65866 | 0.33728845603554103 | No Hit |
| TGGCGGAGCGCCGAGAAGACGATCAAAC | 64898 | 0.3323314945464206 | No Hit |
| GAGAAGACGATCAAACTTGAC | 59594 | 0.30517062291595104 | No Hit |
| TTGGCAGGTGAGTAGAGCCGTTCGTGA | 58401 | 0.29906147513028924 | No Hit |
| CTGGCGGAGCGCCGAGAAGACGATCAAACTTGA | 57556 | 0.2947343754832782 | No Hit |
| CTTTTGGCAGGTGAGTAGAGCCGTTCGTGA | 55053 | 0.281916943037753 | No Hit |
| AGGTGAGTAGAGCCGTTCGTGACA | 51905 | 0.26579657654214245 | No Hit |
| CTGGCGGAGCGCCGAGAAGACGATCAAACT | 51871 | 0.2656224683906651 | No Hit |
| GAAGACGATCAAACTTGA | 48879 | 0.25030095106065664 | No Hit |
| CCTGGCGGAGCGCCGAGAAGACGATCAAAC | 46288 | 0.23703288575248416 | No Hit |
| GCGTGTCGGCTGAGGTGGGATCCCGAC | 46145 | 0.2363006073506823 | No Hit |
| AGAAGACGATCAAACTTGA | 45080 | 0.23084692554705294 | No Hit |
| CAGCGGCGACTCTGGACGCGTGCC | 44403 | 0.22738012500145943 | No Hit |
| GGTTGGCAGCGGCGACTCTGGACGCGTGCC | 43325 | 0.2218598724340299 | No Hit |
| TGGGAATACCAGGTGCTGTAAGCTT | 42353 | 0.21688242763297103 | No Hit |
| TCCCATATGGTCTAGCGGTTAGGATTCC | 40782 | 0.20883760686911962 | No Hit |
| TCCCTGGTGGTCTAGTGGTTAGGATTCGG | 38396 | 0.196619311297796 | No Hit |
| GCATTGGTGGTTCAGTGGTAGAATTCT | 35899 | 0.18383260382017866 | No Hit |
| GGAGCGCCGAGAAGACGATCAAACTTGA | 33894 | 0.1735653437109985 | No Hit |
| TAACACTGTCTGGTAACGATG | 33030 | 0.16914094833227947 | No Hit |
| TAGCTTATCAGACTGGTGTTGGC | 31766 | 0.16266822175970905 | No Hit |
| GCAGCGGCGACTCTGGACGC | 31525 | 0.16143410221541357 | No Hit |
| TCTCGCAAGGGGCTGCTTATGGGGGTTCATTG | 31326 | 0.1604150574464725 | No Hit |
| GTGAAATGTTTAGGACCACTTG | 30753 | 0.15748082301128036 | No Hit |
| AGCGCCGAGAAGACGATCAAACTTGA | 30719 | 0.157306714859803 | No Hit |
| TGGCGGAGCGCCGAGAAGACGATCAAACTTGA | 30415 | 0.15574998315247593 | No Hit |
| TCCCATATGGTCTAGCGGTTAGGATTCCT | 29740 | 0.1522934242628517 | No Hit |
| CCTGGCGGAGCGCCGAGAAGACGATCAAACTTGA | 29654 | 0.15185303305617362 | No Hit |
| TCTTTTGGCAGGTGAGTAGAGCCGTTCGTGACA | 29574 | 0.15144336681740336 | No Hit |
| GAGAAGACGATCAAACTTGACTAT | 28426 | 0.14556465629104984 | No Hit |
| TAGCTTATCAGACTGGTGTTGG | 27918 | 0.14296327567485856 | No Hit |
| TGGCGGAGCGCCGAGAAGACGATCAAACT | 27869 | 0.14271235510361174 | No Hit |
| AACCCGTAGATCCGAACTTGT | 27780 | 0.1422566014129798 | No Hit |
| TGAGAACTGAATTCCATAGATG | 26008 | 0.1331824942242181 | No Hit |
| AGACGATCAAACTTGACTAT | 25601 | 0.13109831723447432 | No Hit |
| TCTCGCAAGGGGCTGCTTATGGG | 25127 | 0.1286710447697604 | No Hit |
| GAGGTGTAGAATAAGTGGGAGGCCC | 22917 | 0.11735401492373142 | No Hit |
| AGCGGCGACTCTGGACGCGTGC | 22782 | 0.11666270314580657 | No Hit |
| GTTGTCGTGGCCGAGTGGTTAAGG | 22017 | 0.11274526973756575 | No Hit |
| CCTGGCGGAGCGCCGAGAAGACGATCAAACT | 21911 | 0.11220246197119514 | No Hit |
| TCTCGCAAGGGGCTGCTTATGGGGG | 21875 | 0.1120181121637485 | No Hit |
| TGAGGTAGTAGATTGAATAGTT | 21772 | 0.11149066688133177 | No Hit |
| CTTTTGGCAGGTGAGTAGAGCCGTTCGTGAC | 21018 | 0.10762956258092188 | No Hit |
| CGAGCGGGCTCTCGCTTCTGGTTTCAAGCAC | 20739 | 0.10620085157321053 | No Hit |
| CGAGAAGACGATCAAACTTGACTATC | 19790 | 0.10134118581579807 | No Hit |
| TCCCTGGTGGTCTAGTGGTTAGGATTCGGC | 19667 | 0.10071132397368877 | No Hit |
| TCGGGCTGGGGTGCGAAGC | 19625 | 0.10049624919833437 | No Hit |

## Adapter Content

Produced by FastQC (version 0.11.9)
