# Supplementary material for: High Throughput Sequencing of MicroRNA in Rainbow Trout Plasma, Mucus, and Surrounding Water Following Acute Stress
Source: Front Physiol. 2021 Jan 13;11:588313. doi: 10.3389/fphys.2020.588313 (PMC7838646; doi:10.3389/fphys.2020.588313)
Supplement: Supplementary file 2 [file Data_Sheet_1.ZIP › Supplemental Quality Control/FastQC_processed_files/plasma_control_1_fastqc_processed.html]

size\_trimmed\_adapterless\_SV18263\_0019\_S9\_R1\_001.fastq FastQC Report 

FastQC Report

Fri 8 May 2020  
size\_trimmed\_adapterless\_SV18263\_0019\_S9\_R1\_001.fastq

## Summary

- Basic Statistics
- Per base sequence quality
- Per tile sequence quality
- Per sequence quality scores
- Per base sequence content
- Per sequence GC content
- Per base N content
- Sequence Length Distribution
- Sequence Duplication Levels
- Overrepresented sequences
- Adapter Content

## Basic Statistics

| Measure | Value |
| --- | --- |
| Filename | size\_trimmed\_adapterless\_SV18263\_0019\_S9\_R1\_001.fastq |
| File type | Conventional base calls |
| Encoding | Sanger / Illumina 1.9 |
| Total Sequences | 22464973 |
| Sequences flagged as poor quality | 0 |
| Sequence length | 18-35 |
| %GC | 52 |

## Per base sequence quality

## Per tile sequence quality

## Per sequence quality scores

## Per base sequence content

## Per sequence GC content

## Per base N content

## Sequence Length Distribution

## Sequence Duplication Levels

## Overrepresented sequences

| Sequence | Count | Percentage | Possible Source |
| --- | --- | --- | --- |
| GCATTGGTGGTTCAGTGGTAGAATTCTCGCCT | 2919659 | 12.996494587373864 | No Hit |
| GCATTGGTGGTTCAGTGGTAGAATTCTCGCC | 1602718 | 7.13429746832992 | No Hit |
| TGAGAACTGAATTCCATAGATGG | 891999 | 3.970621286747151 | No Hit |
| TACCCTGTAGAACCGAATTTGT | 488368 | 2.173908688873118 | No Hit |
| GGTTGGCAGCGGCGACTCTGGACGC | 344042 | 1.531459663895434 | No Hit |
| AACCCGTAGATCCGAACTTGTG | 316308 | 1.408005253333712 | No Hit |
| GAGCCGCGGCTGGGGGAGCA | 274713 | 1.2228503457360043 | No Hit |
| TAACGGAACCCATAATGCAGCTG | 259186 | 1.1537338593729893 | No Hit |
| AACCCGTAGATCCGAACTTGT | 252518 | 1.1240520965682887 | No Hit |
| TCCCTGGTGGTCTAGTGGTTAGGATTCGGCGCT | 248270 | 1.105142659196608 | No Hit |
| GCATTGGTGGTTCAGTGGTAGAATTCTCGCCTG | 232566 | 1.035238279609773 | No Hit |
| GCATTGGTGGTTCAGTGGTAGAATTCTCGC | 232507 | 1.0349756485351662 | No Hit |
| CGAGCCGCGGCTGGGGGAGCA | 217537 | 0.9683385775714041 | No Hit |
| GCATTGTGGTTCAGTGGTAGAATTCTCGCCT | 181193 | 0.8065578356136907 | No Hit |
| TGAGGTAGTAGGTTGTATAGTT | 168263 | 0.7490015679075154 | No Hit |
| TCCCTGGTCTAGTGGTTAGGATTCGGCGCT | 151902 | 0.6761726355068399 | No Hit |
| GAGCCGCGGCTGGGGGAGCAGTT | 148367 | 0.6604370279011686 | No Hit |
| TTCAAGTAATCCAGGATAGGCT | 147612 | 0.6570762404210323 | No Hit |
| AACCCGTAGATCCGAACTTGTGA | 115213 | 0.5128561694687993 | No Hit |
| GTTTCCGTAGTGTAGTGGTTATCACGTTCGCCT | 107623 | 0.47907023970160123 | No Hit |
| CGAGCCGCGGCTGGGGGAGCAGTT | 107414 | 0.4781399025051132 | No Hit |
| TCGCCACTGCTGGAAGTTCGT | 105219 | 0.4683691362549156 | No Hit |
| TGAGAACTGAATTCCATAGATGGT | 102602 | 0.45671989011515834 | No Hit |
| TAGCTTATCAGACTGGTGTTGG | 102111 | 0.45453426540953334 | No Hit |
| TCGTACCGTGAGTAATAATGCA | 90052 | 0.40085514458441596 | No Hit |
| GCATTGTGGTTCAGTGGTAGAATTCTCGCC | 89173 | 0.396942386710191 | No Hit |
| TGAGGTAGTAGATTGAATAGTT | 84448 | 0.3759096438709274 | No Hit |
| TGAGGTAGTAGGTTGTATAGT | 82470 | 0.3671048258103849 | No Hit |
| GCATTGGTGGTTCAGTGGTAGAATTCTC | 80768 | 0.3595285870141041 | No Hit |
| CGAGCCGCGGCTGGGGGAGCAG | 79619 | 0.35441395812049276 | No Hit |
| TAGCTTATCAGACTGGTGTTGGC | 77086 | 0.3431386274089891 | No Hit |
| GAGCCGCGGCTGGGGGAGCAG | 74701 | 0.33252210007107513 | No Hit |
| GTAGGTAATCGTCAGGCT | 67409 | 0.3000626797993481 | No Hit |
| CCGTGTGAAAGTAGGTAATCGTCAGGCT | 66388 | 0.2955178267964088 | No Hit |
| GTTTCCGTAGTGTAGTGGTTATCACGTTCGCC | 64743 | 0.2881953163264429 | No Hit |
| AAGTAGGTAATCGTCAGGCT | 62944 | 0.28018729423801225 | No Hit |
| AAAGTAGGTAATCGTCAGGCT | 62514 | 0.2782732033552856 | No Hit |
| TAACGGAACCCATAAAGCAGCTG | 59905 | 0.26665956820869535 | No Hit |
| TGAAAGTAGGTAATCGTCAGGCT | 59322 | 0.26406441708164974 | No Hit |
| GGTTGGCAGCGGCGACTCTGGACG | 59127 | 0.2631963991232039 | No Hit |
| AGTAGGTAATCGTCAGGCT | 56996 | 0.2537105208183424 | No Hit |
| GAAAGTAGGTAATCGTCAGGCT | 55798 | 0.24837777459158308 | No Hit |
| CCCGTGTGAAAGTAGGTAATCGTCAGGCT | 55027 | 0.24494576512511276 | No Hit |
| GTGAAAGTAGGTAATCGTCAGGCT | 53599 | 0.238589202844802 | No Hit |
| AACCCGTAGATCCGAACTTGTGT | 52322 | 0.23290479806051845 | No Hit |
| TGAGAACTGAATTCCATAGATG | 48142 | 0.21429805413075725 | No Hit |
| GTGTGAAAGTAGGTAATCGTCAGGCT | 42410 | 0.18878277752659664 | No Hit |
| TGTGAAAGTAGGTAATCGTCAGGCT | 41880 | 0.1864235492292824 | No Hit |
| GTGCGAAGCGGGGCTGGGCT | 41716 | 0.18569352386935875 | No Hit |
| AACATTCAACGCTGTCGGTGAG | 36113 | 0.16075247453001615 | No Hit |
| GAGCCGCGGCTGGGGGAGC | 36001 | 0.16025392062567803 | No Hit |
| AACCCGTAGATCCGATCTTGT | 35907 | 0.1598354914559657 | No Hit |
| TGCGAGTTCGAGTCTCGCCGTCGGCACCA | 35756 | 0.15916333395993842 | No Hit |
| TAACGGAACCCATAATGCAGCT | 34709 | 0.15450274522920637 | No Hit |
| CGTGTGAAAGTAGGTAATCGTCAGGCT | 32265 | 0.1436235868166857 | No Hit |
| AGCGCCGAGAAGACGATCAAAC | 31871 | 0.1418697454032106 | No Hit |
| AAGCTGCCAGCTGAAGAACTGT | 31322 | 0.13942594099712471 | No Hit |
| GTTCGATTCCGGCCCTGGGCACCA | 30674 | 0.13654145055059713 | No Hit |
| TACCCTGTAGATCCGGATTTGT | 29725 | 0.1323170964861609 | No Hit |
| TGAGGTAGTAGATTGAATAGT | 29158 | 0.1297931673454493 | No Hit |
| AAAGTTAGGGGATGAGCTG | 28762 | 0.12803042318368243 | No Hit |
| TCCCTGTGGTCTAGTGGTTAGGATTCGGCGCT | 28461 | 0.1266905595657738 | No Hit |
| TATTGCACTTGTCCCGGCCTGT | 28219 | 0.12561332702247183 | No Hit |
| CGAGTCTCGCCGTCGGCACCA | 28182 | 0.12544862617907443 | No Hit |
| ACGTTGAAAAGTTAGGGGATGAGCTG | 28116 | 0.1251548354854466 | No Hit |
| TGAGATGAAGCACTGTAGCT | 27488 | 0.12235937252183654 | No Hit |
| CGAGTTCGAGTCTCGCCGTCGGCACCA | 27053 | 0.12042302476838054 | No Hit |
| TGGAATGTAAGGAAGTGTGTGG | 27004 | 0.12020490743523261 | No Hit |
| AAGTTAGGGGATGAGCTG | 26948 | 0.11995563048306357 | No Hit |
| ACCCTGTAGAACCGAATTTGT | 26771 | 0.11916773725924354 | No Hit |
| GTTGAAAAGTTAGGGGATGAGCTG | 25967 | 0.11558883244595931 | No Hit |
| AACGTTGAAAAGTTAGGGGATGAGCTG | 25873 | 0.11517040327624697 | No Hit |
| GCATTGGTGGTTCAGTGGTAGAATTCTCGCCC | 25774 | 0.11472971723580527 | No Hit |
| ACCCTGTAGAACCGAATTTGTGT | 25600 | 0.11395517813442287 | No Hit |
| TGAACTGAAGAGTTTGATCCTGGCTC | 25528 | 0.1136346791959198 | No Hit |
| AAAAGTTAGGGGATGAGCTG | 24682 | 0.10986881666850878 | No Hit |
| ACCCGGCGATCTAGGCATGACC | 22831 | 0.10162932312449252 | No Hit |
| TGACGGGGGCCTGCACAAGC | 22761 | 0.1013177269342812 | No Hit |

## Adapter Content

Produced by FastQC (version 0.11.9)
