# Supplementary material for: High Throughput Sequencing of MicroRNA in Rainbow Trout Plasma, Mucus, and Surrounding Water Following Acute Stress
Source: Front Physiol. 2021 Jan 13;11:588313. doi: 10.3389/fphys.2020.588313 (PMC7838646; doi:10.3389/fphys.2020.588313)
Supplement: Supplementary file 2 [file Data_Sheet_1.ZIP › Supplemental Quality Control/FastQC_processed_files/plasma_control_2_fastqc_processed.html]

size\_trimmed\_adapterless\_SV18263\_0021\_S11\_R1\_001.fastq FastQC Report 

FastQC Report

Fri 8 May 2020  
size\_trimmed\_adapterless\_SV18263\_0021\_S11\_R1\_001.fastq

## Summary

- Basic Statistics
- Per base sequence quality
- Per tile sequence quality
- Per sequence quality scores
- Per base sequence content
- Per sequence GC content
- Per base N content
- Sequence Length Distribution
- Sequence Duplication Levels
- Overrepresented sequences
- Adapter Content

## Basic Statistics

| Measure | Value |
| --- | --- |
| Filename | size\_trimmed\_adapterless\_SV18263\_0021\_S11\_R1\_001.fastq |
| File type | Conventional base calls |
| Encoding | Sanger / Illumina 1.9 |
| Total Sequences | 24258448 |
| Sequences flagged as poor quality | 0 |
| Sequence length | 18-35 |
| %GC | 49 |

## Per base sequence quality

## Per tile sequence quality

## Per sequence quality scores

## Per base sequence content

## Per sequence GC content

## Per base N content

## Sequence Length Distribution

## Sequence Duplication Levels

## Overrepresented sequences

| Sequence | Count | Percentage | Possible Source |
| --- | --- | --- | --- |
| GCATTGGTGGTTCAGTGGTAGAATTCTCGCCT | 2271350 | 9.36312990839315 | No Hit |
| TGAGAACTGAATTCCATAGATGG | 1100753 | 4.5376068576192505 | No Hit |
| GCATTGGTGGTTCAGTGGTAGAATTCTCGCC | 1072753 | 4.422183150381262 | No Hit |
| TAACGGAACCCATAATGCAGCTG | 658220 | 2.7133640206496312 | No Hit |
| AACCCGTAGATCCGAACTTGTG | 604125 | 2.490369540541093 | No Hit |
| TTCAAGTAATCCAGGATAGGCT | 583561 | 2.4055990721253067 | No Hit |
| TGAGGTAGTAGGTTGTATAGTT | 429840 | 1.771918797113484 | No Hit |
| AACCCGTAGATCCGAACTTGT | 332602 | 1.3710769955274962 | No Hit |
| TACCCTGTAGAACCGAATTTGT | 303687 | 1.2518814064279793 | No Hit |
| AAACCGTTACCATTACTGAGA | 289371 | 1.1928669138272985 | No Hit |
| TGAGGTAGTAGATTGAATAGTT | 253672 | 1.0457058093741198 | No Hit |
| TGAGAACTGAATTCCATAGATGGT | 250818 | 1.0339408357863618 | No Hit |
| GCATTGGTGGTTCAGTGGTAGAATTCTCGCCTG | 219555 | 0.9050661443798877 | No Hit |
| GCATTGGTGGTTCAGTGGTAGAATTCTCGC | 197626 | 0.8146687702362493 | No Hit |
| TAACGGAACCCATAATGCAGCT | 177186 | 0.7304094639525167 | No Hit |
| TCCCTGGTGGTCTAGTGGTTAGGATTCGGCGCT | 168414 | 0.6942488653849579 | No Hit |
| TGAGGTAGTAGGTTGTATAGT | 167631 | 0.6910211238575525 | No Hit |
| GCCCGGCTAGCTCAGTCGGTAGAGCATGAGA | 145939 | 0.6016007289501785 | No Hit |
| AACCCGTAGATCCGAACTTGTGA | 131137 | 0.5405828105738669 | No Hit |
| GCATTGTGGTTCAGTGGTAGAATTCTCGCCT | 122601 | 0.5053950689673139 | No Hit |
| AAACCGTTACCATTACTGAGT | 118710 | 0.4893552959364919 | No Hit |
| TAGCTTATCAGACTGGTGTTGGC | 115775 | 0.47725641805279545 | No Hit |
| TATGGCACTGGTAGAATTCACTG | 114386 | 0.47153057771873946 | No Hit |
| CGAGCCGCGGCTGGGGGAGCA | 113364 | 0.46731761240455283 | No Hit |
| GAGCCGCGGCTGGGGGAGCA | 108723 | 0.448186132929856 | No Hit |
| TAGCTTATCAGACTGGTGTTGG | 107234 | 0.44204806506995004 | No Hit |
| TGAGGTAGTAGGTTGTATAGTTT | 91342 | 0.37653686666187386 | No Hit |
| AAACCGTTACCATTACTGAG | 88360 | 0.36424424184102794 | No Hit |
| TAACGGAACCCATAAAGCAGCTG | 82996 | 0.34213235735443587 | No Hit |
| AACATTCAACGCTGTCGGTGAG | 82465 | 0.3399434291921726 | No Hit |
| TATGGCACTGGTAGAATTCACT | 81435 | 0.3356974856759179 | No Hit |
| TGAGAACTGAATTCCATAGATG | 80066 | 0.3300540908470319 | No Hit |
| TGAGGTAGTAGATTGAATAGT | 79084 | 0.3260060165431853 | No Hit |
| GCCCGGCTAGCTCAGTCGGTAGAGCATGA | 77942 | 0.3212983781979787 | No Hit |
| GTTTCCGTAGTGTAGTGGTTATCACGTTCGCCT | 75179 | 0.3099085316587442 | No Hit |
| GGTTGGCAGCGGCGACTCTGGACGC | 75030 | 0.3092943126452277 | No Hit |
| CCGTGTGAAAGTAGGTAATCGTCAGGCT | 73587 | 0.3033458694472128 | No Hit |
| TAACCGTTACCATTACTGAGA | 67943 | 0.28007974788824086 | No Hit |
| TCGTACCGTGAGTAATAATGCA | 66533 | 0.27426733977375634 | No Hit |
| TCGCCACTGCTGGAAGTTCGT | 65740 | 0.27099837549376615 | No Hit |
| GTTTCCGTAGTGTAGTGGTTATCACGTTCGCC | 63123 | 0.2602103811422726 | No Hit |
| TATTGCACTTGTCCCGGCCTGT | 61979 | 0.255494498246549 | No Hit |
| TCCCTGGTCTAGTGGTTAGGATTCGGCGCT | 61038 | 0.2516154372283008 | No Hit |
| AAAGTAGGTAATCGTCAGGCT | 59193 | 0.244009839376369 | No Hit |
| AACCCGTAGATCCGAACTTGTGT | 58875 | 0.2426989558441661 | No Hit |
| TGAGGTAGTAGTTTGTATAGTT | 58287 | 0.24027505799216836 | No Hit |
| GCATTGTGGTTCAGTGGTAGAATTCTCGCC | 57858 | 0.23850660190627201 | No Hit |
| CCCGTGTGAAAGTAGGTAATCGTCAGGCT | 57447 | 0.23681234677502866 | No Hit |
| TGAAAGTAGGTAATCGTCAGGCT | 56038 | 0.23100406093580264 | No Hit |
| GAAAGTAGGTAATCGTCAGGCT | 54289 | 0.2237942015086868 | No Hit |
| AAGCTGCCAGCTGAAGAACTGT | 53993 | 0.2225740080321709 | No Hit |
| GAGCCGCGGCTGGGGGAGCAGTT | 52090 | 0.21472931821524607 | No Hit |
| AAGTAGGTAATCGTCAGGCT | 51594 | 0.21268466968703026 | No Hit |
| GTAGGTAATCGTCAGGCT | 51419 | 0.2119632715167928 | No Hit |
| GTGAAAGTAGGTAATCGTCAGGCT | 49737 | 0.20502960453199645 | No Hit |
| CGAGCCGCGGCTGGGGGAGCAG | 49344 | 0.2034095503554061 | No Hit |
| AACCCGTAGATCCGATCTTGT | 47476 | 0.19570914017252877 | No Hit |
| AGTAGGTAATCGTCAGGCT | 46704 | 0.19252674367296704 | No Hit |
| CGAGCCGCGGCTGGGGGAGCAGTT | 45817 | 0.18887028551867788 | No Hit |
| TAGCAGCACGTAAATATTGGAG | 45050 | 0.18570850039540865 | No Hit |
| GCCCGGCTAGCTCAGTCGGTAGAGCATGAG | 44536 | 0.18358965091253984 | No Hit |
| TTCAAGTAATCCAGGATAGGC | 43774 | 0.1804484771655631 | No Hit |
| GCTGTGTGAGGTCGGACCTATC | 43063 | 0.17751753945676985 | No Hit |
| TAACGGAACCCATAATGCAGC | 42137 | 0.1737003125673992 | No Hit |
| GTGTGAAAGTAGGTAATCGTCAGGCT | 40838 | 0.16834547700660818 | No Hit |
| TTTGGCAATGGTAGAACTCACACT | 40081 | 0.16522491463592395 | No Hit |
| GAGCCGCGGCTGGGGGAGCAG | 37077 | 0.15284159975939104 | No Hit |
| TGTGAAAGTAGGTAATCGTCAGGCT | 36914 | 0.1521696688922556 | No Hit |
| TTTGGCAATGGTAGAACTCACAC | 36178 | 0.14913567430199987 | No Hit |
| CGTGTGAAAGTAGGTAATCGTCAGGCT | 35116 | 0.14475781797747325 | No Hit |
| GGAATACCAGGTGCTGTAAGCTT | 34988 | 0.1442301667443853 | No Hit |
| TAACGGAACCCATAAAGCAGCT | 33360 | 0.13751910262354788 | No Hit |
| TATTGCACTTGTCCCGGCCTGTAT | 31817 | 0.13115843189968296 | No Hit |
| GAATACCAGGTGCTGTAAGCTT | 31805 | 0.13110896459658095 | No Hit |
| TCCCTGTGGTCTAGTGGTTAGGATTCGGCGCT | 31025 | 0.12789358989495125 | No Hit |
| GCCCGGCTAGCTCAGTCGGTAGAGCATGAGAC | 30271 | 0.1247853943500425 | No Hit |
| TGCGAGTTCGAGTCTCGCCGTCGGCACCA | 30065 | 0.12393620564679157 | No Hit |
| AAACCGTTACCATTACTGA | 29971 | 0.12354871177249262 | No Hit |
| TACCCTGTAGATCCGGATTTGT | 29827 | 0.12295510413526867 | No Hit |
| GCATTGGTGGTTCAGTGGTAGAATTCTC | 29029 | 0.11966552847898597 | No Hit |
| TCACAGTGAACCGGTCTCTTT | 28647 | 0.1180908193302391 | No Hit |
| TCCCTGAGACCCTTAACCTGT | 27783 | 0.11452917350689541 | No Hit |
| TAACCGTTACCATTACTGAGT | 26986 | 0.11124372012587119 | No Hit |
| TACAGTACTGTGATAACTGAAG | 26786 | 0.11041926507417128 | No Hit |
| TGAGAACTGAATTCCAAGGGTT | 26596 | 0.10963603277505635 | No Hit |
| TGAGATGAAGCACTGTAGCT | 26487 | 0.10918670477187987 | No Hit |
| GTGCGAAGCGGGGCTGGGCT | 25568 | 0.10539833380931872 | No Hit |
| TTTGGCAATGGTAGAACTCACA | 25022 | 0.10314757151817791 | No Hit |
| TCGTTTCCCGGCCAATGCACCA | 24835 | 0.10237670604483849 | No Hit |
| TGAGGTAGTAGGTTGTATAG | 24590 | 0.10136674860650607 | No Hit |
| CGAGTCTCGCCGTCGGCACCA | 24460 | 0.10083085282290112 | No Hit |

## Adapter Content

Produced by FastQC (version 0.11.9)
