# Supplementary material for: High Throughput Sequencing of MicroRNA in Rainbow Trout Plasma, Mucus, and Surrounding Water Following Acute Stress
Source: Front Physiol. 2021 Jan 13;11:588313. doi: 10.3389/fphys.2020.588313 (PMC7838646; doi:10.3389/fphys.2020.588313)
Supplement: Supplementary file 2 [file Data_Sheet_1.ZIP › Supplemental Quality Control/FastQC_processed_files/plasma_control_3_fastqc_processed.html]

size\_trimmed\_adapterless\_SV18263\_0023\_S13\_R1\_001.fastq FastQC Report 

FastQC Report

Fri 8 May 2020  
size\_trimmed\_adapterless\_SV18263\_0023\_S13\_R1\_001.fastq

## Summary

- Basic Statistics
- Per base sequence quality
- Per tile sequence quality
- Per sequence quality scores
- Per base sequence content
- Per sequence GC content
- Per base N content
- Sequence Length Distribution
- Sequence Duplication Levels
- Overrepresented sequences
- Adapter Content

## Basic Statistics

| Measure | Value |
| --- | --- |
| Filename | size\_trimmed\_adapterless\_SV18263\_0023\_S13\_R1\_001.fastq |
| File type | Conventional base calls |
| Encoding | Sanger / Illumina 1.9 |
| Total Sequences | 16363363 |
| Sequences flagged as poor quality | 0 |
| Sequence length | 18-35 |
| %GC | 50 |

## Per base sequence quality

## Per tile sequence quality

## Per sequence quality scores

## Per base sequence content

## Per sequence GC content

## Per base N content

## Sequence Length Distribution

## Sequence Duplication Levels

## Overrepresented sequences

| Sequence | Count | Percentage | Possible Source |
| --- | --- | --- | --- |
| AACCCGTAGATCCGAACTTGTG | 1167996 | 7.137872575460191 | No Hit |
| GCATTGGTGGTTCAGTGGTAGAATTCTCGCCT | 1082085 | 6.612852138035439 | No Hit |
| TGAGAACTGAATTCCATAGATGG | 1052491 | 6.4319968945258985 | No Hit |
| TCCCTGGTGGTCTAGTGGTTAGGATTCGGCGCT | 613351 | 3.748318728857876 | No Hit |
| GCATTGGTGGTTCAGTGGTAGAATTCTCGCC | 524905 | 3.2078063659652356 | No Hit |
| TCCCTGGTCTAGTGGTTAGGATTCGGCGCT | 432084 | 2.640557445312434 | No Hit |
| TTCAAGTAATCCAGGATAGGCT | 407688 | 2.491468288028567 | No Hit |
| AACCCGTAGATCCGAACTTGT | 310310 | 1.89637056881278 | No Hit |
| TGAGGTAGTAGATTGAATAGTT | 287544 | 1.7572426890486998 | No Hit |
| TGAGGTAGTAGGTTGTATAGTT | 276445 | 1.6894143337161196 | No Hit |
| TAACGGAACCCATAATGCAGCTG | 235229 | 1.4375345703691838 | No Hit |
| TGAGAACTGAATTCCATAGATGGT | 197561 | 1.2073373914640897 | No Hit |
| TACCCTGTAGAACCGAATTTGT | 179164 | 1.0949094021809576 | No Hit |
| GGTTGGCAGCGGCGACTCTGGACGC | 160311 | 0.9796946996775663 | No Hit |
| AACATTCAACGCTGTCGGTGAG | 137334 | 0.8392773539277958 | No Hit |
| GAGCCGCGGCTGGGGGAGCA | 127664 | 0.7801819222613346 | No Hit |
| GCATTGTGGTTCAGTGGTAGAATTCTCGCCT | 115526 | 0.7060040164115408 | No Hit |
| AACCCGTAGATCCGAACTTGTGA | 114817 | 0.7016711662510939 | No Hit |
| AACCCGTAGATCCGAACTTGTGT | 114583 | 0.7002411423617505 | No Hit |
| GCATTGGTGGTTCAGTGGTAGAATTCTCGC | 108737 | 0.6645149899809715 | No Hit |
| CGAGCCGCGGCTGGGGGAGCA | 105382 | 0.6440118696871786 | No Hit |
| TCGCCACTGCTGGAAGTTCGT | 86614 | 0.5293166203059848 | No Hit |
| GCATTGGTGGTTCAGTGGTAGAATTCTCGCCTG | 85773 | 0.5241770900028313 | No Hit |
| TAGCTTATCAGACTGGTGTTGGC | 82797 | 0.5059901195127188 | No Hit |
| TGAGAACTGAATTCCATAGATG | 76689 | 0.46866282927293124 | No Hit |
| GAGCCGCGGCTGGGGGAGCAGTT | 74102 | 0.45285312071852224 | No Hit |
| GCCCGGCTAGCTCAGTCGGTAGAGCATGAGA | 72754 | 0.4446152053217911 | No Hit |
| TGAGGTAGTAGGTTGTATAGT | 70410 | 0.4302905215755466 | No Hit |
| TAGCTTATCAGACTGGTGTTGG | 67831 | 0.41452970272675604 | No Hit |
| GCCCGGCTAGCTCAGTCGGTAGAGCATGA | 67128 | 0.4102335198455232 | No Hit |
| GTTTCCGTAGTGTAGTGGTTATCACGTTCGCCT | 64567 | 0.3945827028343746 | No Hit |
| CGAGCCGCGGCTGGGGGAGCAGTT | 62843 | 0.3840469712735701 | No Hit |
| TAACGGAACCCATAAAGCAGCTG | 62332 | 0.38092414132718316 | No Hit |
| AACCCGTAGATCCGAACTTGTGC | 58420 | 0.35701707527969645 | No Hit |
| GCATTGTGGTTCAGTGGTAGAATTCTCGCC | 53465 | 0.32673601386218715 | No Hit |
| TCGTACCGTGAGTAATAATGCA | 48937 | 0.29906444048206965 | No Hit |
| TGAGGTAGTAGATTGAATAGT | 47625 | 0.29104652876062215 | No Hit |
| CGAGCCGCGGCTGGGGGAGCAG | 45328 | 0.2770090720348867 | No Hit |
| TCCCTGGTGTCTAGTGGTTAGGATTCGGCGCT | 40652 | 0.24843303910082543 | No Hit |
| GAGCCGCGGCTGGGGGAGCAG | 38011 | 0.23229332503349095 | No Hit |
| TAGCAGCACGTAAATATTGGAG | 37741 | 0.2306432974688638 | No Hit |
| TCGTTTCCCGGCCAATGCACCA | 37112 | 0.2267993443646028 | No Hit |
| TAACGGAACCCATAATGCAGCT | 37107 | 0.22676878829859118 | No Hit |
| GCATTGGTGGTTCAGTGGTAGAATTCTC | 34782 | 0.21256021760319072 | No Hit |
| TATTGCACTTGTCCCGGCCTGT | 31654 | 0.1934443427063251 | No Hit |
| TGAGGTAGTAGGTTGTATAGTTT | 31183 | 0.19056596128803108 | No Hit |
| GTTTCCGTAGTGTAGTGGTTATCACGTTCGCC | 28987 | 0.17714573709573025 | No Hit |
| TCCCTGTGGTCTAGTGGTTAGGATTCGGCGCT | 28946 | 0.17689517735443502 | No Hit |
| GGTTGGCAGCGGCGACTCTGGACG | 27899 | 0.17049673713160307 | No Hit |
| TGAGGTAGTAGTTTGTATAGTT | 27700 | 0.16928060570434084 | No Hit |
| GGAATACCAGGTGCTGTAAGCTT | 27093 | 0.16557109929053093 | No Hit |
| ACCATCGACCGTTGATTGTACC | 26375 | 0.16118324821126317 | No Hit |
| AACCCGTAGATCCGAACTTG | 26149 | 0.15980211402753822 | No Hit |
| TTCAAGTAATCCAGGATAGGC | 25033 | 0.15298200009374602 | No Hit |
| GCCCGGCTAGCTCAGTCGGTAGAGCATGAG | 24787 | 0.1514786416459746 | No Hit |
| TCCCTGGTGGTCTAGTGGTTAGGATTCGGCGCTC | 20825 | 0.12726601493837178 | No Hit |
| AAAGTAGGTAATCGTCAGGCT | 20227 | 0.12361150944338276 | No Hit |
| CCGTGTGAAAGTAGGTAATCGTCAGGCT | 20071 | 0.1226581601838204 | No Hit |
| TCGATTCCCGGCCAATGCACCA | 19677 | 0.12025034218210522 | No Hit |
| GTAGGTAATCGTCAGGCT | 19354 | 0.11827642031775497 | No Hit |
| AACATTCATTGCTGTCGGTGGG | 18249 | 0.11152352972918832 | No Hit |
| CTAAGACTGAGATACGAGACGAGCC | 18177 | 0.11108352237862106 | No Hit |
| AAACCGTTACCATTACTGAGA | 18058 | 0.11035628800754466 | No Hit |
| TCCCTGGTGGTCTAGTGGTTAGGATTCGGCGC | 17842 | 0.10903626595584294 | No Hit |
| AAGTAGGTAATCGTCAGGCT | 17789 | 0.10871237165611984 | No Hit |
| CCCGTGTGAAAGTAGGTAATCGTCAGGCT | 17649 | 0.10785680180779465 | No Hit |
| AAGCTGCCAGCTGAAGAACTGT | 17628 | 0.10772846633054586 | No Hit |
| TAACGGAACCCATAAAGCAGCT | 17539 | 0.10718456835553915 | No Hit |
| GAGCCGCGGCTGGGGGAGC | 17310 | 0.10578510053220723 | No Hit |
| TATTGCACTTGTCCCGGCCTGTAT | 17107 | 0.1045445242521357 | No Hit |
| GTGCGAAGCGGGGCTGGGCT | 17079 | 0.10437341028247066 | No Hit |
| CCCTGAGACCCTTAACCTGTGA | 16826 | 0.102827273342283 | No Hit |
| GAAAGTAGGTAATCGTCAGGCT | 16787 | 0.10258893602739241 | No Hit |
| TCCCTGAGACCCTAACTTGTG | 16758 | 0.10241171084452505 | No Hit |
| AGTAGGTAATCGTCAGGCT | 16566 | 0.10123835790967908 | No Hit |
| GGATTCCTGGAAATACTGTTCT | 16463 | 0.10060890294983982 | No Hit |
| TGAGAACTGAATTCCATAGAT | 16455 | 0.10056001324422126 | No Hit |

## Adapter Content

Produced by FastQC (version 0.11.9)
