# Supplementary material for: High Throughput Sequencing of MicroRNA in Rainbow Trout Plasma, Mucus, and Surrounding Water Following Acute Stress
Source: Front Physiol. 2021 Jan 13;11:588313. doi: 10.3389/fphys.2020.588313 (PMC7838646; doi:10.3389/fphys.2020.588313)
Supplement: Supplementary file 2 [file Data_Sheet_1.ZIP › Supplemental Quality Control/FastQC_processed_files/plasma_stressed_1_fastqc_processed.html]

size\_trimmed\_adapterless\_SV18263\_0020\_S10\_R1\_001.fastq FastQC Report 

FastQC Report

Fri 8 May 2020  
size\_trimmed\_adapterless\_SV18263\_0020\_S10\_R1\_001.fastq

## Summary

- Basic Statistics
- Per base sequence quality
- Per tile sequence quality
- Per sequence quality scores
- Per base sequence content
- Per sequence GC content
- Per base N content
- Sequence Length Distribution
- Sequence Duplication Levels
- Overrepresented sequences
- Adapter Content

## Basic Statistics

| Measure | Value |
| --- | --- |
| Filename | size\_trimmed\_adapterless\_SV18263\_0020\_S10\_R1\_001.fastq |
| File type | Conventional base calls |
| Encoding | Sanger / Illumina 1.9 |
| Total Sequences | 15966963 |
| Sequences flagged as poor quality | 0 |
| Sequence length | 18-35 |
| %GC | 50 |

## Per base sequence quality

## Per tile sequence quality

## Per sequence quality scores

## Per base sequence content

## Per sequence GC content

## Per base N content

## Sequence Length Distribution

## Sequence Duplication Levels

## Overrepresented sequences

| Sequence | Count | Percentage | Possible Source |
| --- | --- | --- | --- |
| GCATTGGTGGTTCAGTGGTAGAATTCTCGCC | 3108115 | 19.465912208852743 | No Hit |
| GCATTGGTGGTTCAGTGGTAGAATTCTCGCCT | 2266841 | 14.197070538711715 | No Hit |
| GCATTGGTGGTTCAGTGGTAGAATTCTCGC | 486223 | 3.045181478782158 | No Hit |
| TGAGAACTGAATTCCATAGATGG | 458813 | 2.8735145186971374 | No Hit |
| AACCCGTAGATCCGAACTTGTG | 379242 | 2.375166774044632 | No Hit |
| TTCAAGTAATCCAGGATAGGCT | 209126 | 1.3097418713878148 | No Hit |
| TCCCTGGTGGTCTAGTGGTTAGGATTCGGCGCT | 198718 | 1.2445572774233897 | No Hit |
| TAACGGAACCCATAATGCAGCTG | 197504 | 1.2369540782426813 | No Hit |
| GCATTGTGGTTCAGTGGTAGAATTCTCGCC | 186569 | 1.1684689192302882 | No Hit |
| GTTTCCGTAGTGTAGTGGTTATCACGTTCGCC | 159628 | 0.9997392741500059 | No Hit |
| AACCCGTAGATCCGAACTTGT | 151300 | 0.9475815782876181 | No Hit |
| TACCCTGTAGAACCGAATTTGT | 148687 | 0.9312165375469337 | No Hit |
| GCATTGTGGTTCAGTGGTAGAATTCTCGCCT | 133598 | 0.8367151599211448 | No Hit |
| TGAGGTAGTAGGTTGTATAGTT | 127420 | 0.7980227673853819 | No Hit |
| TCCCTGGTCTAGTGGTTAGGATTCGGCGCT | 99370 | 0.6223475309612729 | No Hit |
| GTTTCCGTAGTGTAGTGGTTATCACGTTCGCCT | 96219 | 0.6026130329230425 | No Hit |
| TGAGGTAGTAGATTGAATAGTT | 86567 | 0.5421632153841653 | No Hit |
| GCCCGGCTAGCTCAGTCGGTAGAGCATGA | 75250 | 0.4712856164318787 | No Hit |
| TGAGAACTGAATTCCATAGATGGT | 70514 | 0.44162437152262457 | No Hit |
| TAGCTTATCAGACTGGTGTTGG | 67170 | 0.4206811276508876 | No Hit |
| AACATTCAACGCTGTCGGTGAG | 59350 | 0.3717050011326512 | No Hit |
| GAGCCGCGGCTGGGGGAGCA | 58002 | 0.36326256909344623 | No Hit |
| AACCCGTAGATCCGAACTTGTGA | 55816 | 0.3495718002227474 | No Hit |
| GCATTGGTGGTTCAGTGG | 55063 | 0.3448558125925387 | No Hit |
| TAGCTTATCAGACTGGTGTTGGC | 54835 | 0.3434278641467385 | No Hit |
| GCATTGGTGGTTCAGTGGTAGAATTCTCGCCTG | 53523 | 0.3352108976516073 | No Hit |
| TGAGGTAGTAGGTTGTATAGT | 51483 | 0.32243451682076296 | No Hit |
| TCGTACCGTGAGTAATAATGCA | 46581 | 0.2917336252360577 | No Hit |
| GCCCGGCTAGCTCAGTCGGTAGAGCATGAGA | 44663 | 0.2797213220823522 | No Hit |
| AAGCTGCCAGCTGAAGAACTGT | 44536 | 0.2789259297463143 | No Hit |
| TGAGAACTGAATTCCATAGATG | 43350 | 0.2714980926554411 | No Hit |
| TAACGGAACCCATAATGCAGCT | 42049 | 0.263350018409888 | No Hit |
| AAACCGTTACCATTACTGAGA | 37041 | 0.2319852560565212 | No Hit |
| TAACGGAACCCATAAAGCAGCTG | 35927 | 0.22500835005379544 | No Hit |
| CCGTGTGAAAGTAGGTAATCGTCAGGCT | 35837 | 0.22444468619361116 | No Hit |
| CGAGCCGCGGCTGGGGGAGCA | 35033 | 0.21940928904263132 | No Hit |
| TATTGCACTTGTCCCGGCCTGT | 34889 | 0.21850742686633645 | No Hit |
| AAAGTAGGTAATCGTCAGGCT | 32247 | 0.20196076110403713 | No Hit |
| GTAGGTAATCGTCAGGCT | 32075 | 0.20088353683790713 | No Hit |
| CCCGTGTGAAAGTAGGTAATCGTCAGGCT | 31719 | 0.1986539331242892 | No Hit |
| TGAAAGTAGGTAATCGTCAGGCT | 30877 | 0.19338054456567602 | No Hit |
| GAGCCGCGGCTGGGGGAGC | 30717 | 0.1923784754809039 | No Hit |
| GAAAGTAGGTAATCGTCAGGCT | 30403 | 0.19041191490203865 | No Hit |
| GCATTGTGGTTCAGTGGTAGAATTCTCGC | 29453 | 0.18446212971120432 | No Hit |
| AAGTAGGTAATCGTCAGGCT | 29393 | 0.18408635380441477 | No Hit |
| GCATTGGTGGTTCAGTGGTAGAATTCTCG | 28966 | 0.18141208193442923 | No Hit |
| AGTAGGTAATCGTCAGGCT | 28361 | 0.1776230082076347 | No Hit |
| TACCCTGTAGATCCGGATTTGT | 27448 | 0.17190495149265392 | No Hit |
| GTGAAAGTAGGTAATCGTCAGGCT | 27386 | 0.17151664972230474 | No Hit |
| CGAGCCGCGGCTGGGGGAGCAG | 27206 | 0.17038932200193613 | No Hit |
| AACCCGTAGATCCGAACTTGTGT | 26661 | 0.16697602418193114 | No Hit |
| TGAGGTAGTAGATTGAATAGT | 25595 | 0.16029973890463703 | No Hit |
| GAGCCGCGGCTGGGGGAGCAG | 24996 | 0.15654824276852147 | No Hit |
| GTGTGAAAGTAGGTAATCGTCAGGCT | 23341 | 0.14618309067291005 | No Hit |
| GCATTGGTGGTTCAGTGGTAGAATTCTC | 22689 | 0.14209965915246375 | No Hit |
| AACTCTTAGCGGTGGATCACTCGG | 22673 | 0.14199945224398655 | No Hit |
| AACCCGTAGATCCGATCTTGT | 22572 | 0.14136689613422415 | No Hit |
| TCCCTGTGGTCTAGTGGTTAGGATTCGGCGCT | 21766 | 0.1363189731196847 | No Hit |
| CCCAGTGTTCAGACTACCTGTTC | 21719 | 0.13602461532603288 | No Hit |
| GCATTGGTGGTTCAGTGGTAGAATTCTCGCCC | 21696 | 0.1358805678950969 | No Hit |
| GCCCGGCTAGCTCAGTCGGTAGAGCATGAG | 20277 | 0.12699346769952433 | No Hit |
| TGTGAAAGTAGGTAATCGTCAGGCT | 19799 | 0.12399978630876766 | No Hit |
| CGTGTGAAAGTAGGTAATCGTCAGGCT | 19204 | 0.12027334189977143 | No Hit |
| AAACCGTTACCATTACTGAGT | 19198 | 0.12023576430909247 | No Hit |
| GGATTCCTGGAAATACTGTTCT | 19002 | 0.11900822968024663 | No Hit |
| ACGGAAAGGATTGACAGAT | 18279 | 0.11448013000343273 | No Hit |
| GGTTGGCAGCGGCGACTCTGGACGC | 18021 | 0.1128642936042377 | No Hit |
| TCCCTGAGACCCTTAACCTGT | 17671 | 0.11067226748129873 | No Hit |
| TGGTTCAGTGGTAGAATTCTCGCC | 17553 | 0.1099332415312793 | No Hit |
| GTGGTTCAGTGGTAGAATTCTCGCC | 16932 | 0.1060439608960076 | No Hit |
| TATTGCACTTGTCCCGGCCTGTAT | 16735 | 0.10481016333538193 | No Hit |
| TAGCAGCACGTAAATATTGGAG | 16492 | 0.10328827091288431 | No Hit |
| TAACCGTTACCATTACTGAGA | 16084 | 0.10073299474671546 | No Hit |

## Adapter Content

Produced by FastQC (version 0.11.9)
