# Supplementary material for: High Throughput Sequencing of MicroRNA in Rainbow Trout Plasma, Mucus, and Surrounding Water Following Acute Stress
Source: Front Physiol. 2021 Jan 13;11:588313. doi: 10.3389/fphys.2020.588313 (PMC7838646; doi:10.3389/fphys.2020.588313)
Supplement: Supplementary file 2 [file Data_Sheet_1.ZIP › Supplemental Quality Control/FastQC_processed_files/plasma_stressed_2_fastqc_processed.html]

size\_trimmed\_adapterless\_SV18263\_0022\_S12\_R1\_001.fastq FastQC Report 

FastQC Report

Fri 8 May 2020  
size\_trimmed\_adapterless\_SV18263\_0022\_S12\_R1\_001.fastq

## Summary

- Basic Statistics
- Per base sequence quality
- Per tile sequence quality
- Per sequence quality scores
- Per base sequence content
- Per sequence GC content
- Per base N content
- Sequence Length Distribution
- Sequence Duplication Levels
- Overrepresented sequences
- Adapter Content

## Basic Statistics

| Measure | Value |
| --- | --- |
| Filename | size\_trimmed\_adapterless\_SV18263\_0022\_S12\_R1\_001.fastq |
| File type | Conventional base calls |
| Encoding | Sanger / Illumina 1.9 |
| Total Sequences | 20592483 |
| Sequences flagged as poor quality | 0 |
| Sequence length | 18-35 |
| %GC | 47 |

## Per base sequence quality

## Per tile sequence quality

## Per sequence quality scores

## Per base sequence content

## Per sequence GC content

## Per base N content

## Sequence Length Distribution

## Sequence Duplication Levels

## Overrepresented sequences

| Sequence | Count | Percentage | Possible Source |
| --- | --- | --- | --- |
| AACCCGTAGATCCGAACTTGTG | 3218221 | 15.628134790738931 | No Hit |
| GCATTGGTGGTTCAGTGGTAGAATTCTCGCC | 2256162 | 10.956240682583058 | No Hit |
| TGAGAACTGAATTCCATAGATGG | 1136948 | 5.521179743113057 | No Hit |
| TTCAAGTAATCCAGGATAGGCT | 1006228 | 4.886384997865483 | No Hit |
| GCATTGGTGGTTCAGTGGTAGAATTCTCGCCT | 939737 | 4.563495329824966 | No Hit |
| AACCCGTAGATCCGAACTTGT | 762258 | 3.70163228980206 | No Hit |
| TGAGGTAGTAGATTGAATAGTT | 709133 | 3.443649801726193 | No Hit |
| TGAGGTAGTAGGTTGTATAGTT | 597290 | 2.900524429229831 | No Hit |
| GCATTGGTGGTTCAGTGGTAGAATTCTCGC | 419124 | 2.0353252203728904 | No Hit |
| AACATTCAACGCTGTCGGTGAG | 406029 | 1.9717340546062365 | No Hit |
| TAACGGAACCCATAATGCAGCTG | 339893 | 1.650568316603685 | No Hit |
| TCCCTGGTGGTCTAGTGGTTAGGATTCGGCGCT | 288357 | 1.4003022365005715 | No Hit |
| AACCCGTAGATCCGAACTTGTGT | 268980 | 1.3062047932733512 | No Hit |
| AACCCGTAGATCCGAACTTGTGA | 251193 | 1.219828614159837 | No Hit |
| GCATTGTGGTTCAGTGGTAGAATTCTCGCC | 210789 | 1.0236210951345692 | No Hit |
| TGAGAACTGAATTCCATAGATGGT | 198541 | 0.9641430807542734 | No Hit |
| TCCCTGGTCTAGTGGTTAGGATTCGGCGCT | 181757 | 0.8826376110156313 | No Hit |
| AACCCGTAGATCCGAACTTGTGC | 162266 | 0.7879865677198811 | No Hit |
| TACCCTGTAGAACCGAATTTGT | 157020 | 0.7625112522856035 | No Hit |
| TGAGGTAGTAGGTTGTATAGT | 139148 | 0.6757223011911677 | No Hit |
| TGAGGTAGTAGATTGAATAGT | 107544 | 0.5222488225436437 | No Hit |
| TAGCTTATCAGACTGGTGTTGG | 105976 | 0.5146343935308821 | No Hit |
| GTTTCCGTAGTGTAGTGGTTATCACGTTCGCC | 97387 | 0.47292499889401385 | No Hit |
| GCATTGTGGTTCAGTGGTAGAATTCTCGCCT | 94606 | 0.4594200709064565 | No Hit |
| TAGCTTATCAGACTGGTGTTGGC | 92716 | 0.4502419645071456 | No Hit |
| TGAGAACTGAATTCCATAGATG | 91085 | 0.442321598614407 | No Hit |
| TAGCAGCACGTAAATATTGGAG | 80340 | 0.3901423640849916 | No Hit |
| AACCCGTAGATCCGAACTTG | 78413 | 0.38078458047045616 | No Hit |
| TAACGGAACCCATAAAGCAGCTG | 73123 | 0.35509559483428976 | No Hit |
| TATTGCACTTGTCCCGGCCTGT | 65666 | 0.31888335175510407 | No Hit |
| GTTTCCGTAGTGTAGTGGTTATCACGTTCGCCT | 64900 | 0.31516354778585953 | No Hit |
| ACCATCGACCGTTGATTGTACC | 64215 | 0.3118370912337283 | No Hit |
| TCGTACCGTGAGTAATAATGCA | 62979 | 0.30583490101703614 | No Hit |
| TAACGGAACCCATAATGCAGCT | 61687 | 0.2995607669070311 | No Hit |
| TTCAAGTAATCCAGGATAGGC | 54758 | 0.26591256625050996 | No Hit |
| AACATTCATTGCTGTCGGTGGG | 51958 | 0.2523153715848642 | No Hit |
| GGATTCCTGGAAATACTGTTCT | 49047 | 0.2381791452735447 | No Hit |
| TGAGGTAGTAGTTTGTATAGTT | 43966 | 0.21350509309634977 | No Hit |
| TGAGGTAGTAGGTTGTATAGTTT | 42937 | 0.20850812405672497 | No Hit |
| GCATTGTGGTTCAGTGGTAGAATTCTCGC | 42252 | 0.20518166750459377 | No Hit |
| TCCCTGAGACCCTAACTTGTG | 40647 | 0.19738756127660756 | No Hit |
| GCCCGGCTAGCTCAGTCGGTAGAGCATGA | 36385 | 0.17669068853911402 | No Hit |
| GCATTGGTGGTTCAGTGG | 35843 | 0.17405866014312116 | No Hit |
| TCCATAAAGTAGAAAGCACTA | 35824 | 0.17396639346503284 | No Hit |
| GCATTGGTGGTTCAGTGGTAGAATTCTCGCCTG | 33046 | 0.1604760339003315 | No Hit |
| TTCACAGTGGTTAAGTTCTGC | 30837 | 0.14974881853732744 | No Hit |
| TATTGCACTTGTCCCGGCCTGTAT | 28584 | 0.13880793297243466 | No Hit |
| AACATTCAACGCTGTCGGTGA | 26684 | 0.12958126516360363 | No Hit |
| CCCTGAGACCCTTAACCTGTGA | 26048 | 0.12649275951812125 | No Hit |
| GACCATCGACCGTTGATTGTACC | 25856 | 0.1255603804553341 | No Hit |
| TGAGATGAAGCACTGTAGCT | 25477 | 0.12371990303451992 | No Hit |
| ATCACATTGCCAGGGATTTCC | 24424 | 0.11860638661204674 | No Hit |
| AAGCTGCCAGCTGAAGAACTGT | 24351 | 0.11825188832254954 | No Hit |
| TAACGGAACCCATAAAGCAGCT | 24323 | 0.11811591637589308 | No Hit |
| GCCCGGCTAGCTCAGTCGGTAGAGCATGAGA | 24011 | 0.116600800398864 | No Hit |
| TCCCTGAGACCCTTAACCTGT | 23395 | 0.11360941757242193 | No Hit |
| TCCCTGAGACCCTTAACCTGTG | 23326 | 0.11327434384673281 | No Hit |
| TGAGAACTGAATTCCATAGAT | 23069 | 0.11202631562206461 | No Hit |
| AACATTCAACGCTGTCGGTGAGT | 23060 | 0.11198261035349646 | No Hit |
| TCCCTGAGACCCTAACTTGTGA | 22644 | 0.10996245571745766 | No Hit |
| TGAGGTAGTAGATTGAATAGTTT | 22326 | 0.10841820289471649 | No Hit |
| TCGTTTCCCGGCCAATGCACCA | 21172 | 0.10281421623608966 | No Hit |

## Adapter Content

Produced by FastQC (version 0.11.9)
