# Supplementary material for: High Throughput Sequencing of MicroRNA in Rainbow Trout Plasma, Mucus, and Surrounding Water Following Acute Stress
Source: Front Physiol. 2021 Jan 13;11:588313. doi: 10.3389/fphys.2020.588313 (PMC7838646; doi:10.3389/fphys.2020.588313)
Supplement: Supplementary file 2 [file Data_Sheet_1.ZIP › Supplemental Quality Control/FastQC_processed_files/plasma_stressed_3_fastqc_processed.html]

size\_trimmed\_adapterless\_SV18263\_0024\_S14\_R1\_001.fastq FastQC Report 

FastQC Report

Fri 8 May 2020  
size\_trimmed\_adapterless\_SV18263\_0024\_S14\_R1\_001.fastq

## Summary

- Basic Statistics
- Per base sequence quality
- Per tile sequence quality
- Per sequence quality scores
- Per base sequence content
- Per sequence GC content
- Per base N content
- Sequence Length Distribution
- Sequence Duplication Levels
- Overrepresented sequences
- Adapter Content

## Basic Statistics

| Measure | Value |
| --- | --- |
| Filename | size\_trimmed\_adapterless\_SV18263\_0024\_S14\_R1\_001.fastq |
| File type | Conventional base calls |
| Encoding | Sanger / Illumina 1.9 |
| Total Sequences | 16893425 |
| Sequences flagged as poor quality | 0 |
| Sequence length | 18-35 |
| %GC | 50 |

## Per base sequence quality

## Per tile sequence quality

## Per sequence quality scores

## Per base sequence content

## Per sequence GC content

## Per base N content

## Sequence Length Distribution

## Sequence Duplication Levels

## Overrepresented sequences

| Sequence | Count | Percentage | Possible Source |
| --- | --- | --- | --- |
| GCATTGGTGGTTCAGTGGTAGAATTCTCGCC | 2410466 | 14.268663696082942 | No Hit |
| GCATTGGTGGTTCAGTGGTAGAATTCTCGCCT | 1427004 | 8.447097021474331 | No Hit |
| AACCCGTAGATCCGAACTTGTG | 735676 | 4.354806677745928 | No Hit |
| GCATTGGTGGTTCAGTGGTAGAATTCTCGC | 698412 | 4.1342238178462924 | No Hit |
| TGAGAACTGAATTCCATAGATGG | 558275 | 3.3046880665110834 | No Hit |
| TGAGGTAGTAGGTTGTATAGTT | 410847 | 2.4319935122688263 | No Hit |
| TTCAAGTAATCCAGGATAGGCT | 380431 | 2.2519471332781835 | No Hit |
| TGAGGTAGTAGATTGAATAGTT | 320269 | 1.8958204153391038 | No Hit |
| TCCCTGGTGGTCTAGTGGTTAGGATTCGGCGCT | 246699 | 1.4603255408539122 | No Hit |
| TAACGGAACCCATAATGCAGCTG | 241802 | 1.4313379317693127 | No Hit |
| GCATTGTGGTTCAGTGGTAGAATTCTCGCC | 183320 | 1.0851559112494948 | No Hit |
| AACCCGTAGATCCGAACTTGT | 158824 | 0.9401527517362525 | No Hit |
| GTTTCCGTAGTGTAGTGGTTATCACGTTCGCC | 156407 | 0.9258454102705639 | No Hit |
| TCCCTGGTCTAGTGGTTAGGATTCGGCGCT | 139405 | 0.8252027046025302 | No Hit |
| AACATTCAACGCTGTCGGTGAG | 118383 | 0.7007637586812621 | No Hit |
| GCATTGTGGTTCAGTGGTAGAATTCTCGCCT | 111747 | 0.6614822038751763 | No Hit |
| TGAGGTAGTAGGTTGTATAGT | 109102 | 0.6458252249025879 | No Hit |
| TGAGAACTGAATTCCATAGATGGT | 107575 | 0.6367862052840084 | No Hit |
| TACCCTGTAGAACCGAATTTGT | 101681 | 0.6018968918380968 | No Hit |
| GTTTCCGTAGTGTAGTGGTTATCACGTTCGCCT | 82382 | 0.4876571802343219 | No Hit |
| TAGCTTATCAGACTGGTGTTGG | 78057 | 0.46205550384247124 | No Hit |
| TAGCTTATCAGACTGGTGTTGGC | 61499 | 0.3640410396352427 | No Hit |
| AACCCGTAGATCCGAACTTGTGA | 59081 | 0.34972777870680455 | No Hit |
| AACCCGTAGATCCGAACTTGTGT | 56623 | 0.335177739268384 | No Hit |
| GCCCGGCTAGCTCAGTCGGTAGAGCATGA | 54759 | 0.3241438607032026 | No Hit |
| GCATTGTGGTTCAGTGGTAGAATTCTCGC | 53285 | 0.3154185726103499 | No Hit |
| TGAGGTAGTAGATTGAATAGT | 50461 | 0.2987020098055901 | No Hit |
| TAACGGAACCCATAAAGCAGCTG | 50149 | 0.29685513742772707 | No Hit |
| TGAGAACTGAATTCCATAGATG | 49475 | 0.29286541953452305 | No Hit |
| TGAGGTAGTAGGTTGTATAGTTT | 45728 | 0.27068519261191853 | No Hit |
| GAGCCGCGGCTGGGGGAGCA | 45173 | 0.2673998907859123 | No Hit |
| GCATTGGTGGTTCAGTGGTAGAATTCTCGCCTG | 44719 | 0.2647124546976117 | No Hit |
| TAGCAGCACGTAAATATTGGAG | 37908 | 0.2243949939103527 | No Hit |
| CGAGCCGCGGCTGGGGGAGCA | 37906 | 0.22438315498485356 | No Hit |
| TAACGGAACCCATAATGCAGCT | 37512 | 0.22205088666152661 | No Hit |
| TATTGCACTTGTCCCGGCCTGT | 35919 | 0.21262118250147616 | No Hit |
| TCGTACCGTGAGTAATAATGCA | 34893 | 0.20654781372042674 | No Hit |
| CCGTGTGAAAGTAGGTAATCGTCAGGCT | 34564 | 0.20460031047582122 | No Hit |
| AAGCTGCCAGCTGAAGAACTGT | 33732 | 0.19967531746818656 | No Hit |
| AACCCGTAGATCCGAACTTGTGC | 32892 | 0.19470296875855547 | No Hit |
| GCATTGGTGGTTCAGTGGTAGAATTCTCG | 32821 | 0.19428268690333664 | No Hit |
| GCCCGGCTAGCTCAGTCGGTAGAGCATGAGA | 32630 | 0.19315206951817054 | No Hit |
| CCCGTGTGAAAGTAGGTAATCGTCAGGCT | 29947 | 0.17727015096109877 | No Hit |
| GCATTGGTGGTTCAGTGGTAGAATTCTC | 29877 | 0.17685578856862952 | No Hit |
| AAAGTAGGTAATCGTCAGGCT | 28717 | 0.16998921177913892 | No Hit |
| GGTTGGCAGCGGCGACTCTGGACGC | 27507 | 0.1628266618521703 | No Hit |
| TGAAAGTAGGTAATCGTCAGGCT | 27364 | 0.1619801786789831 | No Hit |
| TGAGGTAGTAGTTTGTATAGTT | 26898 | 0.15922170903768773 | No Hit |
| GAAAGTAGGTAATCGTCAGGCT | 25916 | 0.15340879661761897 | No Hit |
| GTAGGTAATCGTCAGGCT | 25083 | 0.14847788414723478 | No Hit |
| ACCATCGACCGTTGATTGTACC | 25078 | 0.14844828683348701 | No Hit |
| AAGTAGGTAATCGTCAGGCT | 24372 | 0.14426914613229702 | No Hit |
| GCATTGGTGGTTCAGTGG | 23929 | 0.1416468241342416 | No Hit |
| AACATTCATTGCTGTCGGTGGG | 23923 | 0.14161130735774421 | No Hit |
| GTGAAAGTAGGTAATCGTCAGGCT | 22750 | 0.13466777755250933 | No Hit |
| AGTAGGTAATCGTCAGGCT | 21035 | 0.12451589893701248 | No Hit |
| GGAATACCAGGTGCTGTAAGCTT | 20994 | 0.12427320096428049 | No Hit |
| TCCCTGTGGTCTAGTGGTTAGGATTCGGCGCT | 20254 | 0.11989279852960547 | No Hit |
| TCCCTGGTGGTCTAGTGGTTAGGATTCGGCGC | 20088 | 0.11891016771317835 | No Hit |
| GAGCCGCGGCTGGGGGAGC | 20004 | 0.11841293284221524 | No Hit |
| GTGTGAAAGTAGGTAATCGTCAGGCT | 19987 | 0.11831230197547271 | No Hit |
| TTCAAGTAATCCAGGATAGGC | 19564 | 0.11580836923240846 | No Hit |
| CGAGCCGCGGCTGGGGGAGCAG | 18899 | 0.11187192650395052 | No Hit |
| TATTGCACTTGTCCCGGCCTGTAT | 18844 | 0.11154635605272464 | No Hit |
| GGATTCCTGGAAATACTGTTCT | 18557 | 0.10984747024360068 | No Hit |
| CGTGTGAAAGTAGGTAATCGTCAGGCT | 18269 | 0.10814266497172717 | No Hit |
| AACCCGTAGATCCGAACTTG | 17867 | 0.10576304094640371 | No Hit |
| GAATACCAGGTGCTGTAAGCTT | 17642 | 0.10443116182775251 | No Hit |
| TGTGAAAGTAGGTAATCGTCAGGCT | 16921 | 0.10016322918531914 | No Hit |

## Adapter Content

Produced by FastQC (version 0.11.9)
