# Supplementary material for: High Throughput Sequencing of MicroRNA in Rainbow Trout Plasma, Mucus, and Surrounding Water Following Acute Stress
Source: Front Physiol. 2021 Jan 13;11:588313. doi: 10.3389/fphys.2020.588313 (PMC7838646; doi:10.3389/fphys.2020.588313)
Supplement: Supplementary file 2 [file Data_Sheet_1.ZIP › Supplemental Quality Control/FastQC_processed_files/water_control_1_fastqc_processed.html]

size\_trimmed\_adapterless\_SV18263\_0013\_S25\_R1\_001.fastq FastQC Report 

FastQC Report

Fri 8 May 2020  
size\_trimmed\_adapterless\_SV18263\_0013\_S25\_R1\_001.fastq

## Summary

- Basic Statistics
- Per base sequence quality
- Per tile sequence quality
- Per sequence quality scores
- Per base sequence content
- Per sequence GC content
- Per base N content
- Sequence Length Distribution
- Sequence Duplication Levels
- Overrepresented sequences
- Adapter Content

## Basic Statistics

| Measure | Value |
| --- | --- |
| Filename | size\_trimmed\_adapterless\_SV18263\_0013\_S25\_R1\_001.fastq |
| File type | Conventional base calls |
| Encoding | Sanger / Illumina 1.9 |
| Total Sequences | 11716657 |
| Sequences flagged as poor quality | 0 |
| Sequence length | 18-35 |
| %GC | 51 |

## Per base sequence quality

## Per tile sequence quality

## Per sequence quality scores

## Per base sequence content

## Per sequence GC content

## Per base N content

## Sequence Length Distribution

## Sequence Duplication Levels

## Overrepresented sequences

| Sequence | Count | Percentage | Possible Source |
| --- | --- | --- | --- |
| TGAGAACTGAATTCCATAGATGG | 222562 | 1.8995349953489293 | No Hit |
| CTTCGGAGTCTGTGGTAGGAAACC | 170620 | 1.4562174176473717 | No Hit |
| TCTTTTGGCAGGTGAGTAGAGCCGTTCGTGAC | 141914 | 1.211215793037212 | No Hit |
| TCTTTTGGCAGGTGAGTAGAGCCGTTCGTGA | 112245 | 0.9579951004795993 | No Hit |
| TTTTGGCAGGTGAGTAGAGCCGTTCGTGAC | 96983 | 0.827736102541877 | No Hit |
| GAGGTGTAGAATAAGTGGGAGGCCC | 94247 | 0.8043847319248143 | No Hit |
| TTTTGGCAGGTGAGTAGAGCCGTTCGTGA | 91724 | 0.7828512859939487 | No Hit |
| AGGTGAGTAGAGCCGTTCGTGACA | 84955 | 0.7250788343466912 | No Hit |
| CCGAGAAGACGATCAAACTTGA | 84567 | 0.7217673095662014 | No Hit |
| AGGTGAGTAGAGCCGTTCGTGAC | 83990 | 0.7168426966838749 | No Hit |
| TTTGGCAGGTGAGTAGAGCCGTTCGTGAC | 78091 | 0.6664955712196747 | No Hit |
| TTGGCAGGTGAGTAGAGCCGTTCGTGA | 77475 | 0.661238098887763 | No Hit |
| AGGTGTAGAATAAGTGGGAGGCCC | 66151 | 0.5645893705004764 | No Hit |
| TTTGGCAGGTGAGTAGAGCCGTTCGTGA | 62841 | 0.5363389915741325 | No Hit |
| GGAATACCAGGTGCTGTAAGCTT | 62617 | 0.5344271834534372 | No Hit |
| GCCGAGAAGACGATCAAACTTGA | 60057 | 0.5125779477883495 | No Hit |
| CTTTTGGCAGGTGAGTAGAGCCGTTCGTGACA | 56047 | 0.4783531684848331 | No Hit |
| CTTTTGGCAGGTGAGTAGAGCCGTTCGTGAC | 52479 | 0.44790079627661716 | No Hit |
| AGGTGTAGAATAAGTGGGAGGCCCCG | 51376 | 0.43848684825372974 | No Hit |
| GAGGTGTAGAATAAGTGGGAGGCCCCG | 49900 | 0.42588939831557754 | No Hit |
| TCTTTTGGCAGGTGAGTAGAGCCGTTCGTGACA | 47172 | 0.40260630655996843 | No Hit |
| TTGGCAGGTGAGTAGAGCCGTTCGTGACA | 45435 | 0.3877812587668991 | No Hit |
| TTTTGGCAGGTGAGTAGAGCCGTTCGTGACA | 43801 | 0.37383530131504233 | No Hit |
| CTTTTGGCAGGTGAGTAGAGCCGTTCGTGA | 40173 | 0.3428708376459258 | No Hit |
| CGTCTGGCGGGCACGGGAAATGTGGTGTATA | 38085 | 0.3250500548065886 | No Hit |
| TTTGGCAGGTGAGTAGAGCCGTTCGTGACA | 37522 | 0.32024492993180564 | No Hit |
| CTCCGGGGATGCGTGCATTTATCAGATC | 37417 | 0.3193487698752298 | No Hit |
| CAGGTGAGTAGAGCCGTTCGTGACA | 37072 | 0.3164042439750519 | No Hit |
| AGGTGAGTAGAGCCGTTCGTGA | 35937 | 0.3067171805063509 | No Hit |
| CTTCGGAGTCTGTGGTAGGAAACCT | 35759 | 0.30519797583901276 | No Hit |
| TAGCTTATCAGACTGGTGTTGG | 30723 | 0.262216432554098 | No Hit |
| CAGGTGAGTAGAGCCGTTCGTGAC | 28498 | 0.2432263742123713 | No Hit |
| TTGGCAGGTGAGTAGAGCCGTTCGTGAC | 27101 | 0.2313031780310715 | No Hit |
| GGTGAGTAGAGCCGTTCGTGACA | 26078 | 0.22257201862271808 | No Hit |
| CCGAGAAGACGATCAAACTTG | 25865 | 0.22075409393652132 | No Hit |
| GAATACCAGGTGCTGTAAGCTT | 24505 | 0.20914668748944343 | No Hit |
| TGTCAACCGGGTCGGACTGTCCTCAGTGCGTAC | 24146 | 0.2060826735817222 | No Hit |
| ACGGGAAATGTGGTGTATA | 23268 | 0.1985890685372116 | No Hit |
| GGTGAGTAGAGCCGTTCGTGAC | 23116 | 0.19729177016959704 | No Hit |
| ATCGACGATGAAATACCACTACTCCTG | 22468 | 0.19176118239187168 | No Hit |
| CCGAGAAGACGATCAAACT | 22279 | 0.19014809429003512 | No Hit |
| ACGGGAAATGTGGTGTATAGAAGAC | 21422 | 0.18283372125683972 | No Hit |
| CAGGTGAGTAGAGCCGTTCGTGA | 20783 | 0.17737994719824948 | No Hit |
| GTCTGGCGGGCACGGGAAATGTGGTGTATA | 19174 | 0.16364736118843456 | No Hit |
| AGGTGTAGAATAAGTGGGAGGCCCCGG | 18099 | 0.15447238918063405 | No Hit |
| CGAGAAGACGATCAAACTTGA | 16930 | 0.14449514055075607 | No Hit |
| TAGCTTATCAGACTGGTGTTGGC | 16555 | 0.14129456892012798 | No Hit |
| ACGGGAAATGTGGTGTATAGA | 16300 | 0.13911818021130087 | No Hit |
| ACGGGAAATGTGGTGTATAGAAGA | 16022 | 0.13674548977579526 | No Hit |
| GAGGTGTAGAATAAGTGGGAGGCCCCGG | 15851 | 0.13528602911222887 | No Hit |
| GCGCGTGTCGGCTGAGGTGGGATCCCGA | 15270 | 0.13032727679917572 | No Hit |
| GTGAGTAGAGCCGTTCGTGA | 15249 | 0.13014804478786055 | No Hit |
| TGGCACTGTGAAGAGACATGAG | 15217 | 0.12987492934204697 | No Hit |
| TGAGAACTGAATTCCATAGATGGT | 15130 | 0.12913239672374127 | No Hit |
| CGCGTGTCGGCTGAGGTGGGATCCCG | 14709 | 0.12553922163975612 | No Hit |
| TAACGGAACCCATAATGCAGCTG | 14291 | 0.121971651128816 | No Hit |
| TGAGAACTGAATTCCATAGATG | 14144 | 0.12071702704960979 | No Hit |
| TGTCAACCGGGTCGGACTGTCCTCAGTGCGTA | 14023 | 0.11968430927012713 | No Hit |
| AATACCGAAGCTCTGGATGTCCGG | 13749 | 0.11734575826534821 | No Hit |
| CCGAGAAGACGATCAAACTTGAC | 13697 | 0.11690194566590112 | No Hit |
| GCATTGGTGGTTCAGTGGTAGAATTCTCGCCT | 13660 | 0.11658615593167915 | No Hit |
| AGGTGTAGAATAAGTGGGAGGCCCC | 13658 | 0.1165690862163158 | No Hit |
| TACCCTGTAGAACCGAATTTGT | 13519 | 0.115382740998563 | No Hit |
| CGAGAAGACGATCAAACTTGACTAT | 13242 | 0.11301858542073903 | No Hit |
| GAGGTGTAGAATAAGTGGGAGGCCCC | 13208 | 0.11272840025956209 | No Hit |
| GAGACAACCCGCTGAATTTAAG | 12260 | 0.10463735517733429 | No Hit |
| GCATTGGTGGTTCAGTGGTAGAATTCTC | 11991 | 0.10234147846096373 | No Hit |

## Adapter Content

Produced by FastQC (version 0.11.9)
