# Supplementary material for: High Throughput Sequencing of MicroRNA in Rainbow Trout Plasma, Mucus, and Surrounding Water Following Acute Stress
Source: Front Physiol. 2021 Jan 13;11:588313. doi: 10.3389/fphys.2020.588313 (PMC7838646; doi:10.3389/fphys.2020.588313)
Supplement: Supplementary file 2 [file Data_Sheet_1.ZIP › Supplemental Quality Control/FastQC_processed_files/water_control_2_fastqc_processed.html]

size\_trimmed\_adapterless\_SV18263\_0015\_S27\_R1\_001.fastq FastQC Report 

FastQC Report

Fri 8 May 2020  
size\_trimmed\_adapterless\_SV18263\_0015\_S27\_R1\_001.fastq

## Summary

- Basic Statistics
- Per base sequence quality
- Per tile sequence quality
- Per sequence quality scores
- Per base sequence content
- Per sequence GC content
- Per base N content
- Sequence Length Distribution
- Sequence Duplication Levels
- Overrepresented sequences
- Adapter Content

## Basic Statistics

| Measure | Value |
| --- | --- |
| Filename | size\_trimmed\_adapterless\_SV18263\_0015\_S27\_R1\_001.fastq |
| File type | Conventional base calls |
| Encoding | Sanger / Illumina 1.9 |
| Total Sequences | 22204316 |
| Sequences flagged as poor quality | 0 |
| Sequence length | 18-35 |
| %GC | 51 |

## Per base sequence quality

## Per tile sequence quality

## Per sequence quality scores

## Per base sequence content

## Per sequence GC content

## Per base N content

## Sequence Length Distribution

## Sequence Duplication Levels

## Overrepresented sequences

| Sequence | Count | Percentage | Possible Source |
| --- | --- | --- | --- |
| AGAATAGTGGAAGGCTCTGGAAAGTGC | 419055 | 1.8872682229887199 | No Hit |
| GAGAATAGTGGAAGGCTCTGGAAAGTGC | 388952 | 1.751695481184829 | No Hit |
| ATCAAGGCCGAGAACTGATGACGAGTT | 284868 | 1.2829397672056189 | No Hit |
| AGATTAGCGGAACGCTCTGGAAAGTGC | 237566 | 1.0699091113637547 | No Hit |
| ATTTGGAATTGTACAGTCAAGGTGT | 227839 | 1.0261023127215447 | No Hit |
| TCAAGGCCGAGAACTGATGACGAGTT | 218224 | 0.9827999205199566 | No Hit |
| GAGATTAGCGGAACGCTCTGGAAAGTGC | 205889 | 0.927247657617555 | No Hit |
| TCTTTTGGCAGGTGAGTAGAGCCGTTCGTGAC | 204153 | 0.9194293577879183 | No Hit |
| GCCGAGAACTGATGACGAGTT | 169161 | 0.7618383741251025 | No Hit |
| AGGTGAGTAGAGCCGTTCGTGAC | 164957 | 0.7429051180860514 | No Hit |
| ATCAAGGCCGAGAACTGATGACGAGTTAT | 160487 | 0.7227738967505236 | No Hit |
| TGAGAACTGAATTCCATAGATGG | 145678 | 0.656079655865103 | No Hit |
| AAGGCCGAGAACTGATGACGAGTT | 144463 | 0.6506077467101441 | No Hit |
| TCAAGGCCGAGAACTGATGACGAGTTAT | 137218 | 0.6179789550824264 | No Hit |
| CAAGGCCGAGAACTGATGACGAGTT | 129556 | 0.5834721501891795 | No Hit |
| GAATTAGTGGAAGGCTCTGGAAAGTGC | 119711 | 0.539133923332743 | No Hit |
| ATCAAGGCCGAGAACTGATGACGAGTTA | 111391 | 0.5016637306008436 | No Hit |
| AGGTGAGTAGAGCCGTTCGTGACA | 109220 | 0.49188635218486354 | No Hit |
| GGAATACCAGGTGCTGTAAGCTT | 91564 | 0.412370279723996 | No Hit |
| TCAAGGCCGAGAACTGATGACGAGTTA | 90214 | 0.40629038066293055 | No Hit |
| CTAAGACTGAGATACGAGACGAGCC | 86818 | 0.3909960568026505 | No Hit |
| CCGAGAAGACGATCAAACT | 84602 | 0.3810160150846349 | No Hit |
| TGAGATTAGCGGAACGCTCTGGAAAGTGC | 77470 | 0.348896133526473 | No Hit |
| CAAGGCCGAGAACTGATGACGAGTTAT | 73864 | 0.3326560475900271 | No Hit |
| CCGAGAAGACGATCAAACTTGA | 70831 | 0.3189965410328335 | No Hit |
| GCACCGAAGCTGTGGACTTGC | 70549 | 0.3177265176734109 | No Hit |
| TCTTTTGGCAGGTGAGTAGAGCCGTTCGTGA | 68547 | 0.30871025254729756 | No Hit |
| GCCGAGAGCTGATGACGAGTT | 65441 | 0.294721981077913 | No Hit |
| AGGCCGAGAACTGATGACGAGTT | 65134 | 0.29333936699513735 | No Hit |
| AAGGCCGAGAACTGATGACGAGTTAT | 63421 | 0.28562465063098547 | No Hit |
| GCCGAGAACTGATGACGAGTTAT | 61024 | 0.2748294520758937 | No Hit |
| AGACTGAGATACGAGACGAGCC | 59384 | 0.26744350062393274 | No Hit |
| TCTTTTGGCAGGTGAGTAGAGCCGTTCGTGACA | 58601 | 0.26391715916851477 | No Hit |
| GCCGAGAAGACGATCAAACTTGA | 52944 | 0.23844013028818364 | No Hit |
| AGGTGAGTAGAGCCGTTCGTGA | 52275 | 0.23542720253125565 | No Hit |
| CAAGGCCGAGAACTGATGACGAGTTA | 49402 | 0.22248827660352158 | No Hit |
| AAGGCCGAGAACTGATGACGAGTTA | 48561 | 0.21870072466992452 | No Hit |
| TGAGAATAGTGGAAGGCTCTGGAAAGTGC | 43737 | 0.19697521869171739 | No Hit |
| GCCGAGAACTGATGACGAGTTA | 42827 | 0.1928769163616659 | No Hit |
| AGGTGTAGAATAAGTGGGAGGCCC | 42788 | 0.19270127483323513 | No Hit |
| CTAAGGCCGAGAGCTGATGACGAGTC | 41181 | 0.1854639431361002 | No Hit |
| GCACCCGTAGCTCAGCTGGA | 40396 | 0.18192859442281403 | No Hit |
| CTAAGGCCGAGAGCTGATGACGAGTCAT | 40239 | 0.1812215246801568 | No Hit |
| GAGGTGTAGAATAAGTGGGAGGCCC | 39953 | 0.17993348680499774 | No Hit |
| AGAATTAGTGGAAGGCTCTGGAAAGTGC | 36954 | 0.16642710363156424 | No Hit |
| CTAAGGCCGAGAGCTGATGACGAGTCA | 34857 | 0.15698299375670927 | No Hit |
| AATTAGTGGAAGGCTCTGGAAAGTGC | 34014 | 0.15318643456524397 | No Hit |
| GTTAAGCCGGGAACTTTAAGGATACTGCC | 32019 | 0.14420169484166953 | No Hit |
| CAGGTGAGTAGAGCCGTTCGTGAC | 31372 | 0.14128784692129223 | No Hit |
| CTCCGGGGATGCGTGCATTTATCAGATC | 31215 | 0.14058077717863499 | No Hit |
| CTTAATGCCGAGAACTGATGACGATCCT | 30745 | 0.1384640715795974 | No Hit |
| AAGACTGAGATACGAGACGAGCC | 30729 | 0.13839201351665145 | No Hit |
| CCACAAGATTAAGAATGAGGCAATGAT | 30394 | 0.1368832978237204 | No Hit |
| GCCGAGAGCTGATGACGAGTC | 29795 | 0.13418562409218102 | No Hit |
| CCTAAGACTGAGATACGAGACGAGCC | 29747 | 0.1339694499033431 | No Hit |
| CTTTCGAGGCCCTGTAATTGGAATGAGTA | 29532 | 0.13300116968250678 | No Hit |
| TGACTGTAAATGGTGATTAAATGCAT | 29343 | 0.1321499838139576 | No Hit |
| GGCCGAGAACTGATGACGAGTT | 28430 | 0.12803817059710373 | No Hit |
| AGGCCGAGAACTGATGACGAGTTAT | 28174 | 0.12688524158996836 | No Hit |
| TTAATGCCGAGAACTGATGACGATCCT | 27980 | 0.1260115375767486 | No Hit |
| AGGTGTAGAATAAGTGGGAGGCCCCG | 27957 | 0.12590795411126376 | No Hit |
| TGCTTGACGACCATAGAGAA | 27771 | 0.125070279129517 | No Hit |
| CGTCTGGCGGGCACGGGAAATGTGGTGTATA | 27757 | 0.12500722832443925 | No Hit |
| TTTTGGCAGGTGAGTAGAGCCGTTCGTGA | 26433 | 0.11904442361566102 | No Hit |
| GTAAGGCGAACCAGGGGAACTGAAAC | 26155 | 0.11779241477197495 | No Hit |
| CGAGAAGACGATCAAACTTGA | 26136 | 0.11770684582222664 | No Hit |
| GGTGAGTAGAGCCGTTCGTGAC | 25746 | 0.11595043053791886 | No Hit |
| TGGCACTGTGAAGAGACATGAG | 25354 | 0.11418500799574281 | No Hit |
| GTAAGGCGAACCAGGGGAACTGAAA | 25249 | 0.11371212695765993 | No Hit |
| GCGCGTGTCGGCTGAGGTGGGATCCCGA | 25057 | 0.11284743020230842 | No Hit |
| GAATACCAGGTGCTGTAAGCTT | 24736 | 0.11140176531445507 | No Hit |
| TAGTGGAAGGCTCTGGAAAGTGC | 24308 | 0.10947421213065064 | No Hit |
| TTTTGGCAGGTGAGTAGAGCCGTTCGTGAC | 23908 | 0.10767276055700162 | No Hit |
| AGGCCGAGAACTGATGACGAGTTA | 23613 | 0.10634419002143547 | No Hit |
| TTGGCAGGTGAGTAGAGCCGTTCGTGA | 23248 | 0.10470036546048075 | No Hit |
| CTTAATGCCGAGAACTGATGACGATCCTT | 23160 | 0.10430404611427796 | No Hit |
| GCACCGAAGCTGTGGACTT | 22811 | 0.1027322796162692 | No Hit |
| TAAGACTGAGATACGAGACGAGCC | 22305 | 0.1004534433756032 | No Hit |

## Adapter Content

Produced by FastQC (version 0.11.9)
