# Supplementary material for: High Throughput Sequencing of MicroRNA in Rainbow Trout Plasma, Mucus, and Surrounding Water Following Acute Stress
Source: Front Physiol. 2021 Jan 13;11:588313. doi: 10.3389/fphys.2020.588313 (PMC7838646; doi:10.3389/fphys.2020.588313)
Supplement: Supplementary file 2 [file Data_Sheet_1.ZIP › Supplemental Quality Control/FastQC_processed_files/water_control_3_fastqc_processed.html]

size\_trimmed\_adapterless\_SV18263\_0017\_S29\_R1\_001.fastq FastQC Report 

FastQC Report

Fri 8 May 2020  
size\_trimmed\_adapterless\_SV18263\_0017\_S29\_R1\_001.fastq

## Summary

- Basic Statistics
- Per base sequence quality
- Per tile sequence quality
- Per sequence quality scores
- Per base sequence content
- Per sequence GC content
- Per base N content
- Sequence Length Distribution
- Sequence Duplication Levels
- Overrepresented sequences
- Adapter Content

## Basic Statistics

| Measure | Value |
| --- | --- |
| Filename | size\_trimmed\_adapterless\_SV18263\_0017\_S29\_R1\_001.fastq |
| File type | Conventional base calls |
| Encoding | Sanger / Illumina 1.9 |
| Total Sequences | 10847696 |
| Sequences flagged as poor quality | 0 |
| Sequence length | 18-35 |
| %GC | 51 |

## Per base sequence quality

## Per tile sequence quality

## Per sequence quality scores

## Per base sequence content

## Per sequence GC content

## Per base N content

## Sequence Length Distribution

## Sequence Duplication Levels

## Overrepresented sequences

| Sequence | Count | Percentage | Possible Source |
| --- | --- | --- | --- |
| AGGTGAGTAGAGCCGTTCGTGAC | 116659 | 1.0754265237521405 | No Hit |
| TGAGAACTGAATTCCATAGATGG | 89555 | 0.8255670144148581 | No Hit |
| GGAATACCAGGTGCTGTAAGCTT | 88790 | 0.8185148256367066 | No Hit |
| TCTTTTGGCAGGTGAGTAGAGCCGTTCGTGAC | 78520 | 0.7238403436084493 | No Hit |
| ATTTGGAATTGTACAGTCAAGGTGT | 76425 | 0.7045274867584785 | No Hit |
| CCGAGAAGACGATCAAACT | 70649 | 0.6512811568465783 | No Hit |
| GCACCGAAGCTGTGGACTTGC | 53870 | 0.49660314964578656 | No Hit |
| CTAAGACTGAGATACGAGACGAGCC | 49169 | 0.45326675821298823 | No Hit |
| AGGTGAGTAGAGCCGTTCGTGACA | 47031 | 0.4335575038238535 | No Hit |
| AGGTGAGTAGAGCCGTTCGTGA | 40829 | 0.37638407271000224 | No Hit |
| AGAATAGTGGAAGGCTCTGGAAAGTGC | 40511 | 0.3734525746296725 | No Hit |
| ATCAAGGCCGAGAACTGATGACGAGTT | 36144 | 0.33319517803596266 | No Hit |
| GAGAATAGTGGAAGGCTCTGGAAAGTGC | 35967 | 0.33156349514219424 | No Hit |
| GAATTAGTGGAAGGCTCTGGAAAGTGC | 33882 | 0.31234282376644773 | No Hit |
| ATCAAGGCCGAGAACTGATGACGAGTTAT | 31085 | 0.28655854662593794 | No Hit |
| CCGAGAAGACGATCAAACTTGA | 29807 | 0.2747772430200846 | No Hit |
| TCAAGGCCGAGAACTGATGACGAGTT | 29093 | 0.26819520016047643 | No Hit |
| AGACTGAGATACGAGACGAGCC | 28683 | 0.2644155957172841 | No Hit |
| CTGTGAGGATCTGATAGTATGGCGACT | 27328 | 0.2519244639599045 | No Hit |
| TTCTATACCGAGATCTGATAGCAAGCT | 26818 | 0.24722300477447007 | No Hit |
| GCCGAGAAGACGATCAAACTTGA | 25615 | 0.23613309222529835 | No Hit |
| TCAAGGCCGAGAACTGATGACGAGTTAT | 25142 | 0.2317727192944935 | No Hit |
| ATCAAGGCCGAGAACTGATGACGAGTTA | 24012 | 0.22135576070715846 | No Hit |
| TCTTTTGGCAGGTGAGTAGAGCCGTTCGTGA | 23487 | 0.21651602331038775 | No Hit |
| TACCGAGATCTGATAGCAAGCT | 23175 | 0.2136398365145926 | No Hit |
| TCAAGGCCGAGAACTGATGACGAGTTA | 21395 | 0.1972308221026843 | No Hit |
| AGATTAGCGGAACGCTCTGGAAAGTGC | 20850 | 0.19220671375746518 | No Hit |
| TAAGCCGAGCAATACTAATGAATC | 20537 | 0.1893213084142476 | No Hit |
| CGAGAAGACGATCAAACTTGAC | 20520 | 0.18916459310806646 | No Hit |
| CTCCGGGGATGCGTGCATTTATCAGATC | 20408 | 0.18813211579675534 | No Hit |
| CGAGAAGACGATCAAACTTGA | 20373 | 0.18780946663697065 | No Hit |
| CGAGAAGACGATCAAACTTGACTAT | 19394 | 0.1787845087104211 | No Hit |
| GAATACCAGGTGCTGTAAGCTT | 19204 | 0.1770329847001612 | No Hit |
| CCTAAGACTGAGATACGAGACGAGCC | 18827 | 0.1735575923219087 | No Hit |
| CGTCTGGCGGGCACGGGAA | 18327 | 0.16894831861069853 | No Hit |
| AAGGCCGAGAACTGATGACGAGTT | 17789 | 0.16398874009743636 | No Hit |
| CGTCTGGCGGGCACGGGAAATGTGGTGTATA | 17282 | 0.1593149365542692 | No Hit |
| TCTTTTGGCAGGTGAGTAGAGCCGTTCGTGACA | 16664 | 0.1536178742472134 | No Hit |
| AAGACTGAGATACGAGACGAGCC | 16426 | 0.15142385996067736 | No Hit |
| CAGGTGAGTAGAGCCGTTCGTGAC | 15919 | 0.14675005641751024 | No Hit |
| AGGTGTAGAATAAGTGGGAGGCCC | 15234 | 0.14043535143315225 | No Hit |
| GAGATTAGCGGAACGCTCTGGAAAGTGC | 14995 | 0.13823211859919377 | No Hit |
| GGTGAGTAGAGCCGTTCGTGAC | 14166 | 0.13058994278600727 | No Hit |
| CAAGGCCGAGAACTGATGACGAGTT | 13887 | 0.12801796805515198 | No Hit |
| AGGCCGAGAACTGATGACGAGTT | 13804 | 0.1272528286190911 | No Hit |
| CTTTCGAGGCCCTGTAATTGGAATGAGTA | 13193 | 0.12162029614399225 | No Hit |
| GCCGAGAAGACGATCAAACT | 13093 | 0.1206984414017502 | No Hit |
| GTGAGTAGAGCCGTTCGTGA | 13016 | 0.11998861325022382 | No Hit |
| GGAGAGATGACTGAGAGGCCGAAAGTAGCCGTTT | 12856 | 0.11851364566263657 | No Hit |
| ATACCGAGATCTGATAGCAAGCT | 12686 | 0.1169464926008251 | No Hit |
| TTTAAGTTGAACAGATTGGGAAGTCT | 12379 | 0.11411639854214203 | No Hit |
| GCGCGTGTCGGCTGAGGTGGGATCCCGA | 12212 | 0.11257690112259783 | No Hit |
| TACCCTGTAGAACCGAATTTGT | 11999 | 0.11061335052162229 | No Hit |
| TAAGACTGAGATACGAGACGAGCC | 11851 | 0.10924900550310407 | No Hit |
| TAGCTTATCAGACTGGTGTTGG | 11743 | 0.10825340238148266 | No Hit |
| GCCGAGAACTGATGACGAGTT | 11539 | 0.10637281870730891 | No Hit |
| GCACCGAAGCTGTGGACTTGCA | 11314 | 0.10429864553726431 | No Hit |
| AGGATTCAACTCGGCGGGTCAGGG | 11266 | 0.10385615526098814 | No Hit |
| TGGCACTGTGAAGAGACATGAG | 11211 | 0.10334913515275503 | No Hit |
| GAGGTGTAGAATAAGTGGGAGGCCC | 11031 | 0.10168979661671934 | No Hit |
| GTCTGGCGGGCACGGGAAATGTGGTGTATA | 10938 | 0.10083247170643425 | No Hit |

## Adapter Content

Produced by FastQC (version 0.11.9)
