# Supplementary material for: High Throughput Sequencing of MicroRNA in Rainbow Trout Plasma, Mucus, and Surrounding Water Following Acute Stress
Source: Front Physiol. 2021 Jan 13;11:588313. doi: 10.3389/fphys.2020.588313 (PMC7838646; doi:10.3389/fphys.2020.588313)
Supplement: Supplementary file 2 [file Data_Sheet_1.ZIP › Supplemental Quality Control/FastQC_processed_files/water_stressed_1_fastqc_processed.html]

size\_trimmed\_adapterless\_SV18263\_0014\_S26\_R1\_001.fastq FastQC Report 

FastQC Report

Fri 8 May 2020  
size\_trimmed\_adapterless\_SV18263\_0014\_S26\_R1\_001.fastq

## Summary

- Basic Statistics
- Per base sequence quality
- Per tile sequence quality
- Per sequence quality scores
- Per base sequence content
- Per sequence GC content
- Per base N content
- Sequence Length Distribution
- Sequence Duplication Levels
- Overrepresented sequences
- Adapter Content

## Basic Statistics

| Measure | Value |
| --- | --- |
| Filename | size\_trimmed\_adapterless\_SV18263\_0014\_S26\_R1\_001.fastq |
| File type | Conventional base calls |
| Encoding | Sanger / Illumina 1.9 |
| Total Sequences | 17065802 |
| Sequences flagged as poor quality | 0 |
| Sequence length | 18-35 |
| %GC | 50 |

## Per base sequence quality

## Per tile sequence quality

## Per sequence quality scores

## Per base sequence content

## Per sequence GC content

## Per base N content

## Sequence Length Distribution

## Sequence Duplication Levels

## Overrepresented sequences

| Sequence | Count | Percentage | Possible Source |
| --- | --- | --- | --- |
| TGAGAACTGAATTCCATAGATGG | 1468743 | 8.606352048383076 | No Hit |
| CTTTTGGCAGGTGAGTAGAGCCGTTCGTGACA | 413903 | 2.425335767987933 | No Hit |
| TTGGCAGGTGAGTAGAGCCGTTCGTGA | 296483 | 1.7372930964510196 | No Hit |
| CCGAGAAGACGATCAAACTTGA | 270994 | 1.5879359200346985 | No Hit |
| CAGGTGAGTAGAGCCGTTCGTGACA | 238276 | 1.396219175635578 | No Hit |
| GAGGTGTAGAATAAGTGGGAGGCCC | 220107 | 1.2897547973426622 | No Hit |
| GCCGAGAAGACGATCAAACTTGA | 215016 | 1.2599232078281466 | No Hit |
| TAGCTTATCAGACTGGTGTTGG | 187262 | 1.0972938746154444 | No Hit |
| TACCCTGTAGAACCGAATTTGT | 174282 | 1.0212353336807727 | No Hit |
| GGTGAGTAGAGCCGTTCGTGACA | 161278 | 0.9450361606211065 | No Hit |
| TTGGCAGGTGAGTAGAGCCGTTCGTGACA | 157558 | 0.9232381812469171 | No Hit |
| TTTTGGCAGGTGAGTAGAGCCGTTCGTGACA | 115198 | 0.6750224806311476 | No Hit |
| TTTTGGCAGGTGAGTAGAGCCGTTCGTGA | 111562 | 0.65371671369444 | No Hit |
| GGAATACCAGGTGCTGTAAGCTT | 110576 | 0.6479390772259047 | No Hit |
| AGGTGTAGAATAAGTGGGAGGCCC | 99959 | 0.5857269409313433 | No Hit |
| AACCCGTAGATCCGAACTTGT | 99515 | 0.5831252466189399 | No Hit |
| GATCGGGGGCCTGAGTCCT | 97888 | 0.5735915604786695 | No Hit |
| TGAGAACTGAATTCCATAGATG | 96444 | 0.5651301942914843 | No Hit |
| TAGCTTATCAGACTGGTGTTGGC | 89211 | 0.5227471876211853 | No Hit |
| TCTTTTGGCAGGTGAGTAGAGCCGTTCGTGA | 88465 | 0.5183758724025979 | No Hit |
| TAACGGAACCCATAATGCAGCTG | 87592 | 0.5132603788559131 | No Hit |
| AGGTGAGTAGAGCCGTTCGTGACA | 86961 | 0.5095629259029256 | No Hit |
| AACCCGTAGATCCGAACTTGTG | 82206 | 0.48170018613833676 | No Hit |
| CTTTTGGCAGGTGAGTAGAGCCGTTCGTGA | 78044 | 0.45731223179549374 | No Hit |
| TAACACTGTCTGGTAACGATG | 77088 | 0.451710385483202 | No Hit |
| TGAGAACTGAATTCCATAGATGGT | 76748 | 0.4497180970457761 | No Hit |
| CAGGTGAGTAGAGCCGTTCGTGA | 71903 | 0.4213279868124568 | No Hit |
| TCTTTTGGCAGGTGAGTAGAGCCGTTCGTGAC | 70312 | 0.41200524885967854 | No Hit |
| TTTTGGCAGGTGAGTAGAGCCGTTCGTGAC | 67969 | 0.3982760376570641 | No Hit |
| TGAAATGTTTAGGACCACTCG | 63145 | 0.3700089805331153 | No Hit |
| GAATACCAGGTGCTGTAAGCTT | 62428 | 0.36580759579889655 | No Hit |
| CTCCGGGGATGCGTGCATTTATCAGATC | 58466 | 0.34259157583100985 | No Hit |
| CTTTTGGCAGGTGAGTAGAGCCGTTCGTGAC | 54144 | 0.31726607398820167 | No Hit |
| TGGACGGAGAACTGATAAGG | 51632 | 0.30254657823874903 | No Hit |
| TCTTTTGGCAGGTGAGTAGAGCCGTTCGTGACA | 49274 | 0.2887294719580129 | No Hit |
| GCATTGGTGGTTCAGTGGTAGAATTCTCGCCT | 45499 | 0.26660921063071047 | No Hit |
| TAATACTGCCTGGTAATGATGA | 45057 | 0.26401923566205676 | No Hit |
| AGGTGAGTAGAGCCGTTCGTGAC | 44896 | 0.2630758284902169 | No Hit |
| TAACGGAACCCATAAAGCAGCTG | 44092 | 0.2583646523028921 | No Hit |
| TGTCAACCGGGTCGGACTGTCCTCAGTGCGTAC | 41385 | 0.24250252053785695 | No Hit |
| CTTCGGAGTCTGTGGTAGGAAACC | 40637 | 0.23811948597551993 | No Hit |
| TGGCGGGCACGGGAAATGTGGTGTATA | 40289 | 0.23608032016309574 | No Hit |
| GTCTGGCGGGCACGGGAAATGTGGTGTATA | 39143 | 0.22936513619459548 | No Hit |
| TTTGGCAGGTGAGTAGAGCCGTTCGTGA | 39035 | 0.228732291632119 | No Hit |
| GCATTGGTGGTTCAGTGGTAGAATTCTCGCC | 36829 | 0.21580585547634973 | No Hit |
| GTGGTTGGCAGCGGCGACTCTGGACGCGTGCC | 35538 | 0.208241019086006 | No Hit |
| GTGAAATGTTTAGGACCACTTG | 34851 | 0.2042154245080307 | No Hit |
| GCATTGGTGGTTCAGTGGTAGAATTCTCGC | 33403 | 0.1957306196333463 | No Hit |
| TGAGGTAGTAGATTGAATAGTT | 32367 | 0.18965999957107202 | No Hit |
| CAGGTGAGTAGAGCCGTTCGTGAC | 31976 | 0.1873688678680322 | No Hit |
| TGAGGTAGTAGGTTGTATAGTT | 31177 | 0.18268699004008132 | No Hit |
| TTTGGCAGGTGAGTAGAGCCGTTCGTGACA | 30193 | 0.17692107291529574 | No Hit |
| GTAGAGCCGTTCGTGACA | 29966 | 0.1755909273997202 | No Hit |
| GTGAAATGTTTAGGACCACTCG | 29205 | 0.17113171710301103 | No Hit |
| CCGAGAAGACGATCAAACTTG | 29078 | 0.17038753877491372 | No Hit |
| TGAAATGTTTAGGACCACTCGA | 27926 | 0.16363719677516475 | No Hit |
| TTCAAGTAATCCAGGATAGGCT | 27755 | 0.162635192884577 | No Hit |
| CGGATTGAATTAGAATAACTTGGAAAAGT | 27698 | 0.16230119158771442 | No Hit |
| AGGTGAGTAGAGCCGTTCGTGA | 27666 | 0.1621136820877214 | No Hit |
| GGTGAGTAGAGCCGTTCGTGA | 26500 | 0.1552813046817255 | No Hit |
| GGTGAGTAGAGCCGTTCGTGAC | 26201 | 0.15352926279116563 | No Hit |
| GCATTGGTGGTTCAGTGGTAGAATTCTC | 24498 | 0.14355024158841173 | No Hit |
| TGAGGTAGTAGGTTGTATAGT | 24326 | 0.1425423780259492 | No Hit |
| TGAGGTAGTAGATTGAATAGT | 23711 | 0.1389386798229582 | No Hit |
| AAATTGATTTTTGGAATAGGGA | 23691 | 0.13882148638546257 | No Hit |
| GGCTTAGGCTGGCGGATCGTTTGAGC | 22976 | 0.13463182099499338 | No Hit |
| CGGGAAATGTGGTGTATAGAAGAC | 22613 | 0.13250476010444748 | No Hit |
| TGAGAACTGAATTCCATAGATGT | 22036 | 0.1291237294326982 | No Hit |
| AACATTCAACGCTGTCGGTGAG | 21755 | 0.12747716163588443 | No Hit |
| AGAATTAGTGGAAGGCTCTGGAAAGTGC | 21689 | 0.12709042329214884 | No Hit |
| CTTTTGGCAGGTGAGTAGAGCCGTTCGTGACAG | 21660 | 0.12692049280778014 | No Hit |
| CTGATGCGCACCGCATGTTTGTGGAGAACC | 21408 | 0.12544385549533504 | No Hit |
| AATTGATTTTTGGAATAGGGA | 21273 | 0.12465279979223948 | No Hit |
| TTTGGCAGGTGAGTAGAGCCGTTCGTGAC | 21149 | 0.12392620047976649 | No Hit |
| CGTCTGGCGGGCACGGGAAATGTGGTGTATA | 20954 | 0.122783564464184 | No Hit |
| TCGCGGATCTCCCCAGCTACGGTGCTCGCTGGC | 20845 | 0.12214486022983273 | No Hit |
| TAATACTGCCTGGTAATGATGAT | 20713 | 0.1213713835423615 | No Hit |
| TGGACGGAGAACTGATAAGGG | 19808 | 0.11606838049568372 | No Hit |
| CGAGAAGACGATCAAACTTGA | 18670 | 0.10940007390218168 | No Hit |
| TGTCAACCGGGTCGGACTGTCCTCAGTGCGTA | 18526 | 0.10855628115221308 | No Hit |
| CGGGAAATGTGGTGTATA | 18367 | 0.1076245933241227 | No Hit |
| GGGAATACCAGGTGCTGTAAGCTT | 18186 | 0.10656399271478714 | No Hit |
| GAGGTGTAGAATAAGTGGGAGGCCCCG | 17847 | 0.10457756394923602 | No Hit |

## Adapter Content

Produced by FastQC (version 0.11.9)
