# Supplementary material for: High Throughput Sequencing of MicroRNA in Rainbow Trout Plasma, Mucus, and Surrounding Water Following Acute Stress
Source: Front Physiol. 2021 Jan 13;11:588313. doi: 10.3389/fphys.2020.588313 (PMC7838646; doi:10.3389/fphys.2020.588313)
Supplement: Supplementary file 2 [file Data_Sheet_1.ZIP › Supplemental Quality Control/FastQC_processed_files/water_stressed_2_fastqc_processed.html]

size\_trimmed\_adapterless\_SV18263\_0016\_S28\_R1\_001.fastq FastQC Report 

FastQC Report

Fri 8 May 2020  
size\_trimmed\_adapterless\_SV18263\_0016\_S28\_R1\_001.fastq

## Summary

- Basic Statistics
- Per base sequence quality
- Per tile sequence quality
- Per sequence quality scores
- Per base sequence content
- Per sequence GC content
- Per base N content
- Sequence Length Distribution
- Sequence Duplication Levels
- Overrepresented sequences
- Adapter Content

## Basic Statistics

| Measure | Value |
| --- | --- |
| Filename | size\_trimmed\_adapterless\_SV18263\_0016\_S28\_R1\_001.fastq |
| File type | Conventional base calls |
| Encoding | Sanger / Illumina 1.9 |
| Total Sequences | 24251897 |
| Sequences flagged as poor quality | 0 |
| Sequence length | 18-35 |
| %GC | 50 |

## Per base sequence quality

## Per tile sequence quality

## Per sequence quality scores

## Per base sequence content

## Per sequence GC content

## Per base N content

## Sequence Length Distribution

## Sequence Duplication Levels

## Overrepresented sequences

| Sequence | Count | Percentage | Possible Source |
| --- | --- | --- | --- |
| TGAGAACTGAATTCCATAGATGG | 693189 | 2.85828774549059 | No Hit |
| AGAATAGTGGAAGGCTCTGGAAAGTGC | 452462 | 1.8656767344839045 | No Hit |
| GAGAATAGTGGAAGGCTCTGGAAAGTGC | 358365 | 1.4776782203882854 | No Hit |
| TAGCTTATCAGACTGGTGTTGG | 211009 | 0.8700721432224455 | No Hit |
| AGATTAGCGGAACGCTCTGGAAAGTGC | 202152 | 0.8335512887919654 | No Hit |
| GAGATTAGCGGAACGCTCTGGAAAGTGC | 186702 | 0.7698449321304639 | No Hit |
| ATCAAGGCCGAGAACTGATGACGAGTT | 161481 | 0.6658489436929408 | No Hit |
| GAATTAGTGGAAGGCTCTGGAAAGTGC | 148252 | 0.6113006335133289 | No Hit |
| TCTTTTGGCAGGTGAGTAGAGCCGTTCGTGAC | 140244 | 0.5782805361576457 | No Hit |
| TCAAGGCCGAGAACTGATGACGAGTT | 135682 | 0.5594696365401849 | No Hit |
| GCCGAGAACTGATGACGAGTT | 118879 | 0.49018433485842366 | No Hit |
| AGGTGAGTAGAGCCGTTCGTGAC | 116870 | 0.4819004467980381 | No Hit |
| AGGTGAGTAGAGCCGTTCGTGACA | 105774 | 0.43614732488761604 | No Hit |
| GGAATACCAGGTGCTGTAAGCTT | 104985 | 0.4328939711396597 | No Hit |
| TAACGGAACCCATAATGCAGCTG | 104249 | 0.42985915699707944 | No Hit |
| AAGGCCGAGAACTGATGACGAGTT | 102213 | 0.4214639374396156 | No Hit |
| TACCCTGTAGAACCGAATTTGT | 99621 | 0.4107761137200937 | No Hit |
| GCCGAGAAGACGATCAAACTTGA | 98218 | 0.4049909992608001 | No Hit |
| CCGAGAAGACGATCAAACTTGA | 92089 | 0.3797187494240141 | No Hit |
| TCTTTTGGCAGGTGAGTAGAGCCGTTCGTGA | 90684 | 0.37392538818715915 | No Hit |
| ATCAAGGCCGAGAACTGATGACGAGTTAT | 90485 | 0.37310483381980386 | No Hit |
| CTTTTGGCAGGTGAGTAGAGCCGTTCGTGACA | 90173 | 0.3718183365202318 | No Hit |
| CAAGGCCGAGAACTGATGACGAGTT | 88937 | 0.3667218279873117 | No Hit |
| ATTTGGAATTGTACAGTCAAGGTGT | 84351 | 0.3478119670391145 | No Hit |
| TTTTGGCAGGTGAGTAGAGCCGTTCGTGA | 81362 | 0.33548715797366285 | No Hit |
| CTAAGACTGAGATACGAGACGAGCC | 80858 | 0.33340897002820025 | No Hit |
| TCAAGGCCGAGAACTGATGACGAGTTAT | 80151 | 0.33049373416025973 | No Hit |
| AACCCGTAGATCCGAACTTGT | 77925 | 0.3213150707344667 | No Hit |
| CAGGTGAGTAGAGCCGTTCGTGACA | 75972 | 0.31326209244579917 | No Hit |
| AACCCGTAGATCCGAACTTGTG | 69546 | 0.28676519614115137 | No Hit |
| GAGGTGTAGAATAAGTGGGAGGCCC | 68376 | 0.2819408312677561 | No Hit |
| TTTTGGCAGGTGAGTAGAGCCGTTCGTGAC | 66458 | 0.2740321715864124 | No Hit |
| TAGCTTATCAGACTGGTGTTGGC | 66371 | 0.2736734367624932 | No Hit |
| GCACCGAAGCTGTGGACTTGC | 62806 | 0.2589735557593701 | No Hit |
| ATCAAGGCCGAGAACTGATGACGAGTTA | 58870 | 0.24274389751861472 | No Hit |
| GGTGAGTAGAGCCGTTCGTGACA | 58235 | 0.2401255456428831 | No Hit |
| TTGGCAGGTGAGTAGAGCCGTTCGTGA | 55809 | 0.23012220446095413 | No Hit |
| TGAGATTAGCGGAACGCTCTGGAAAGTGC | 54314 | 0.22395773823383797 | No Hit |
| AGGCCGAGAACTGATGACGAGTT | 53831 | 0.22196614145276963 | No Hit |
| TCAAGGCCGAGAACTGATGACGAGTTA | 53606 | 0.2210383789771167 | No Hit |
| TCTTTTGGCAGGTGAGTAGAGCCGTTCGTGACA | 53519 | 0.2206796441531976 | No Hit |
| TAACACTGTCTGGTAACGATG | 51903 | 0.21401624788361917 | No Hit |
| GAATACCAGGTGCTGTAAGCTT | 50199 | 0.20698999340134092 | No Hit |
| CTTTTGGCAGGTGAGTAGAGCCGTTCGTGA | 47311 | 0.1950816466027379 | No Hit |
| TAACGGAACCCATAAAGCAGCTG | 46239 | 0.19066137382984927 | No Hit |
| CAAGGCCGAGAACTGATGACGAGTTAT | 45736 | 0.1885873092731674 | No Hit |
| TGAGAACTGAATTCCATAGATG | 44404 | 0.18309495541730197 | No Hit |
| AGGTGTAGAATAAGTGGGAGGCCC | 43180 | 0.17804792754975002 | No Hit |
| TGAGGTAGTAGGTTGTATAGTT | 42434 | 0.17497187951936297 | No Hit |
| CAGGTGAGTAGAGCCGTTCGTGAC | 41783 | 0.17228755342314045 | No Hit |
| TTTTGGCAGGTGAGTAGAGCCGTTCGTGACA | 41217 | 0.16995371537327575 | No Hit |
| TGAGAATAGTGGAAGGCTCTGGAAAGTGC | 40897 | 0.16863423096345825 | No Hit |
| AGAATTAGTGGAAGGCTCTGGAAAGTGC | 39561 | 0.16312538355247014 | No Hit |
| CTTTTGGCAGGTGAGTAGAGCCGTTCGTGAC | 39336 | 0.1621976210768172 | No Hit |
| TAAGCCGAGCAATACTAATGAATC | 38932 | 0.1605317720094226 | No Hit |
| CATATACCGAGGCTGTTGATCGAGCGA | 38926 | 0.16050703167673852 | No Hit |
| TGGACGGAGAACTGATAAGG | 38450 | 0.15854429861713498 | No Hit |
| AGGTGAGTAGAGCCGTTCGTGA | 38315 | 0.1579876411317432 | No Hit |
| CATATACCGAGGCTGTTGATCGAGCG | 36322 | 0.14976972729184854 | No Hit |
| TGAAATGTTTAGGACCACTCG | 36192 | 0.1492336867503602 | No Hit |
| GGTGAGTAGAGCCGTTCGTGAC | 36014 | 0.14849972354739918 | No Hit |
| AAGGCCGAGAACTGATGACGAGTTAT | 35819 | 0.14769566273516666 | No Hit |
| AATTAGTGGAAGGCTCTGGAAAGTGC | 35214 | 0.14520101252285542 | No Hit |
| TGAGAACTGAATTCCATAGATGGT | 34760 | 0.14332899401642685 | No Hit |
| TAATACTGCCTGGTAATGATGA | 34483 | 0.14218681532417857 | No Hit |
| CGGATTGAATTAGAATAACTTGGAAAAGT | 34449 | 0.14204662010563546 | No Hit |
| CAGGTGAGTAGAGCCGTTCGTGA | 33921 | 0.13986947082943657 | No Hit |
| TTGGCAGGTGAGTAGAGCCGTTCGTGACA | 33058 | 0.13631098631170996 | No Hit |
| TTAATGCCGAGAACTGATGACGATCCT | 32266 | 0.13304526239741163 | No Hit |
| CTCCGGGGATGCGTGCATTTATCAGATC | 32252 | 0.13298753495448212 | No Hit |
| TTTAAGTTGAACAGATTGGGAAGTCT | 32243 | 0.132950424455456 | No Hit |
| CTTAATGCCGAGAACTGATGACGATCCT | 32206 | 0.13279785907057085 | No Hit |
| TGAGGTAGTAGGTTGTATAGT | 31910 | 0.13157733599148966 | No Hit |
| CAAGGCCGAGAACTGATGACGAGTTA | 31867 | 0.13140003027392042 | No Hit |
| CGAGAAGACGATCAAACTTGA | 31623 | 0.13039392341143458 | No Hit |
| AGACTGAGATACGAGACGAGCC | 31487 | 0.12983314253726214 | No Hit |
| AAGGCCGAGAACTGATGACGAGTTA | 30120 | 0.12419647007407297 | No Hit |
| GCCGAGAACTGATGACGAGTTAT | 30058 | 0.12394081996967082 | No Hit |
| TTTGGCAGGTGAGTAGAGCCGTTCGTGA | 29769 | 0.1227491606120544 | No Hit |
| GCATTGGTGGTTCAGTGGTAGAATTCTCGCCT | 28161 | 0.11611875145272142 | No Hit |
| TAGTGGAAGGCTCTGGAAAGTGC | 27407 | 0.1130097163120889 | No Hit |
| GCCGAGAGCTGATGACGAGTT | 26546 | 0.10945947857192367 | No Hit |
| TGGCGAATTGTAGTCTATTGAGGCT | 25803 | 0.10639580070787864 | No Hit |
| TTAATGCCGAGAACTGATGACGATCCTT | 25728 | 0.10608654654932767 | No Hit |
| CTTAATGCCGAGAACTGATGACGATCCTT | 25310 | 0.10436297003900354 | No Hit |
| AAGCCGAGTAATACTAATGAATC | 25283 | 0.10425163854192519 | No Hit |
| CCTAAGACTGAGATACGAGACGAGCC | 25101 | 0.10350118178384148 | No Hit |
| ATGGTGACGGATCTCAAAGGTAGTTTG | 25070 | 0.10337335673164041 | No Hit |
| TGAGGTAGTAGATTGAATAGTT | 24925 | 0.10277546535844187 | No Hit |

## Adapter Content

Produced by FastQC (version 0.11.9)
