# Supplementary material for: High Throughput Sequencing of MicroRNA in Rainbow Trout Plasma, Mucus, and Surrounding Water Following Acute Stress
Source: Front Physiol. 2021 Jan 13;11:588313. doi: 10.3389/fphys.2020.588313 (PMC7838646; doi:10.3389/fphys.2020.588313)
Supplement: Supplementary file 2 [file Data_Sheet_1.ZIP › Supplemental Quality Control/FastQC_processed_files/water_stressed_3_fastqc_processed.html]

size\_trimmed\_adapterless\_SV18263\_0018\_S30\_R1\_001.fastq FastQC Report 

FastQC Report

Fri 8 May 2020  
size\_trimmed\_adapterless\_SV18263\_0018\_S30\_R1\_001.fastq

## Summary

- Basic Statistics
- Per base sequence quality
- Per tile sequence quality
- Per sequence quality scores
- Per base sequence content
- Per sequence GC content
- Per base N content
- Sequence Length Distribution
- Sequence Duplication Levels
- Overrepresented sequences
- Adapter Content

## Basic Statistics

| Measure | Value |
| --- | --- |
| Filename | size\_trimmed\_adapterless\_SV18263\_0018\_S30\_R1\_001.fastq |
| File type | Conventional base calls |
| Encoding | Sanger / Illumina 1.9 |
| Total Sequences | 26910169 |
| Sequences flagged as poor quality | 0 |
| Sequence length | 18-35 |
| %GC | 49 |

## Per base sequence quality

## Per tile sequence quality

## Per sequence quality scores

## Per base sequence content

## Per sequence GC content

## Per base N content

## Sequence Length Distribution

## Sequence Duplication Levels

## Overrepresented sequences

| Sequence | Count | Percentage | Possible Source |
| --- | --- | --- | --- |
| TGAGAACTGAATTCCATAGATGG | 1274983 | 4.737922678969426 | No Hit |
| TACCCTGTAGAACCGAATTTGT | 444768 | 1.652787836449485 | No Hit |
| AACCCGTAGATCCGAACTTGT | 286159 | 1.0633861125138233 | No Hit |
| CCGAGAAGACGATCAAACTTGA | 219551 | 0.8158662994647117 | No Hit |
| GAATTAGTGGAAGGCTCTGGAAAGTGC | 209374 | 0.7780478821965034 | No Hit |
| GCCGAGAAGACGATCAAACTTGA | 207622 | 0.7715373322255984 | No Hit |
| GGAATACCAGGTGCTGTAAGCTT | 206903 | 0.7688654798117396 | No Hit |
| CTAAGACTGAGATACGAGACGAGCC | 190957 | 0.7096090700879657 | No Hit |
| TACCGAGATCTGATAGCAAGCT | 184753 | 0.6865545883416786 | No Hit |
| TAGCTTATCAGACTGGTGTTGG | 177407 | 0.6592563576988312 | No Hit |
| GAGAATAGTGGAAGGCTCTGGAAAGTGC | 150670 | 0.5598998653631644 | No Hit |
| AGAATAGTGGAAGGCTCTGGAAAGTGC | 147899 | 0.549602642777903 | No Hit |
| GCACCGAAGCTGTGGACTTGC | 146764 | 0.5453849063526878 | No Hit |
| TCAAGGCCGAGAACTGATGACGAGTT | 134579 | 0.5001046258758167 | No Hit |
| TCAAGGCCGAGAACTGATGACGAGTTAT | 124132 | 0.4612828704271608 | No Hit |
| ATCAAGGCCGAGAACTGATGACGAGTT | 119010 | 0.4422491735373345 | No Hit |
| TAACGGAACCCATAATGCAGCTG | 117656 | 0.43721761836575607 | No Hit |
| CTTTTGGCAGGTGAGTAGAGCCGTTCGTGACA | 113485 | 0.4217179015115067 | No Hit |
| CCGAGAAGACGATCAAACT | 113018 | 0.41998249806606563 | No Hit |
| AACCCGTAGATCCGAACTTGTG | 113002 | 0.4199230409887058 | No Hit |
| TCAAGGCCGAGAACTGATGACGAGTTA | 109773 | 0.4079238595640184 | No Hit |
| TGGACGGAGAACTGATAAGG | 104084 | 0.3867831524952519 | No Hit |
| GAGATTAGCGGAACGCTCTGGAAAGTGC | 103791 | 0.38569434476609943 | No Hit |
| AAGGCCGAGAACTGATGACGAGTT | 102580 | 0.38119418722342474 | No Hit |
| AGGCCGAGAACTGATGACGAGTT | 99653 | 0.3703172581339047 | No Hit |
| TCTTTTGGCAGGTGAGTAGAGCCGTTCGTGAC | 99055 | 0.3680950498675798 | No Hit |
| ATACCGAGATCTGATAGCAAGCT | 93908 | 0.3489684512943787 | No Hit |
| ATCAAGGCCGAGAACTGATGACGAGTTA | 92762 | 0.3447098381284785 | No Hit |
| ATTTGGAATTGTACAGTCAAGGTGT | 91708 | 0.34079310315739747 | No Hit |
| AGACTGAGATACGAGACGAGCC | 90919 | 0.3378611260300892 | No Hit |
| AGGTGAGTAGAGCCGTTCGTGAC | 90576 | 0.3365865149341871 | No Hit |
| TCTTTTGGCAGGTGAGTAGAGCCGTTCGTGA | 89263 | 0.33170731852334334 | No Hit |
| ATCAAGGCCGAGAACTGATGACGAGTTAT | 85579 | 0.31801732646123476 | No Hit |
| AGGTGAGTAGAGCCGTTCGTGACA | 83575 | 0.3105703275219119 | No Hit |
| CAGGTGAGTAGAGCCGTTCGTGACA | 81779 | 0.30389627058826724 | No Hit |
| TTTTGGCAGGTGAGTAGAGCCGTTCGTGA | 81517 | 0.30292266094649944 | No Hit |
| TAGCTTATCAGACTGGTGTTGGC | 77183 | 0.2868172251166464 | No Hit |
| AGAATTAGTGGAAGGCTCTGGAAAGTGC | 77154 | 0.28670945916393165 | No Hit |
| CAAGGCCGAGAACTGATGACGAGTT | 76281 | 0.28346533238048416 | No Hit |
| TAACACTGTCTGGTAACGATG | 76261 | 0.2833910110337843 | No Hit |
| TTCTATACCGAGATCTGATAGCAAGCT | 75941 | 0.282201869486587 | No Hit |
| GAATACCAGGTGCTGTAAGCTT | 75089 | 0.2790357801171743 | No Hit |
| GCCGAGAACTGATGACGAGTT | 68680 | 0.255219504567214 | No Hit |
| TGAGAACTGAATTCCATAGATG | 68100 | 0.2530641855129189 | No Hit |
| TGAGAACTGAATTCCATAGATGGT | 64565 | 0.23992788748372407 | No Hit |
| CAAGGCCGAGAACTGATGACGAGTTAT | 61702 | 0.22928878670364353 | No Hit |
| AGATTAGCGGAACGCTCTGGAAAGTGC | 60925 | 0.22640140238435516 | No Hit |
| CCTAAGACTGAGATACGAGACGAGCC | 60272 | 0.22397481041460574 | No Hit |
| TAAGCCGAGCAATACTAATGAATC | 59006 | 0.2192702691685065 | No Hit |
| GGTGAGTAGAGCCGTTCGTGACA | 57394 | 0.21327996862450024 | No Hit |
| AATTAGTGGAAGGCTCTGGAAAGTGC | 56441 | 0.2097385564542534 | No Hit |
| GCATTGGTGGTTCAGTGGTAGAATTCTCGCCT | 54765 | 0.20351042760080768 | No Hit |
| TTGGCAGGTGAGTAGAGCCGTTCGTGA | 54119 | 0.2011098481024032 | No Hit |
| GCACCGAAGCTGTGGACTTGCA | 52198 | 0.1939712827518846 | No Hit |
| TTTTATACCGAGATCTGATAGCAAGCT | 51045 | 0.18968665711463945 | No Hit |
| CAAGGCCGAGAACTGATGACGAGTTA | 50718 | 0.18847150309609725 | No Hit |
| CGAGAAGACGATCAAACTTGA | 50159 | 0.186394221455837 | No Hit |
| TCTTTTGGCAGGTGAGTAGAGCCGTTCGTGACA | 49188 | 0.1827859200735603 | No Hit |
| TGAAATGTTTAGGACCACTCG | 48927 | 0.18181602649912754 | No Hit |
| TTTTGGCAGGTGAGTAGAGCCGTTCGTGAC | 48748 | 0.18115085044616405 | No Hit |
| CTTTTGGCAGGTGAGTAGAGCCGTTCGTGA | 47859 | 0.17784726658535666 | No Hit |
| AAGGCCGAGAACTGATGACGAGTTA | 47640 | 0.1770334478389935 | No Hit |
| ATTAGTGGAAGGCTCTGGAAAGTGC | 47222 | 0.17548013169296708 | No Hit |
| AGGCCGAGAACTGATGACGAGTTA | 46045 | 0.1711063204396821 | No Hit |
| TTTTGGCAGGTGAGTAGAGCCGTTCGTGACA | 45721 | 0.1699023146231449 | No Hit |
| AAGACTGAGATACGAGACGAGCC | 45467 | 0.16895843352005704 | No Hit |
| TAACGGAACCCATAAAGCAGCTG | 45284 | 0.16827839319775362 | No Hit |
| GGAATACCAGGTGCTGTAAGCT | 41649 | 0.1547704884350596 | No Hit |
| CGAGAAGACGATCAAACTTGACTAT | 41472 | 0.15411274451676615 | No Hit |
| TAAGACTGAGATACGAGACGAGCC | 38599 | 0.14343648306333565 | No Hit |
| AAGCCGAGCAATACTAATGAATC | 36655 | 0.13621244816411224 | No Hit |
| TTGGCAGGTGAGTAGAGCCGTTCGTGACA | 36176 | 0.1344324519106513 | No Hit |
| AACCCGTAGATCCGAACTTGTGT | 35995 | 0.13375984372301786 | No Hit |
| GCCGAGAACTGATGACGATCC | 35811 | 0.13307608733337944 | No Hit |
| AAGCCGAGTAATACTAATGAATC | 35730 | 0.13277508587924514 | No Hit |
| AAGGCCGAGAACTGATGACGAGTTAT | 35694 | 0.13264130745518543 | No Hit |
| TACCGAGATCTGATAGCAA | 35215 | 0.13086131120172453 | No Hit |
| TGAGGTAGTAGGTTGTATAGTT | 34532 | 0.12832323721192535 | No Hit |
| TAATACTGCCTGGTAATGATGA | 33701 | 0.12523518525654745 | No Hit |
| CTTTTGGCAGGTGAGTAGAGCCGTTCGTGAC | 33592 | 0.12483013391703338 | No Hit |
| GCATTGGTGGTTCAGTGGTAGAATTCTCGCC | 33340 | 0.12389368494861552 | No Hit |
| CAGGTGAGTAGAGCCGTTCGTGAC | 33311 | 0.12378591899590077 | No Hit |
| GAGGTGTAGAATAAGTGGGAGGCCC | 33202 | 0.12338086765638671 | No Hit |
| AGGCCGAGAACTGATGACGAGTTAT | 32961 | 0.12248529542865376 | No Hit |
| CAGGTGAGTAGAGCCGTTCGTGA | 32799 | 0.12188329252038514 | No Hit |
| TTTCATACCGAGATCTGATAGCAAGCT | 32358 | 0.1202445068256539 | No Hit |
| CGAGAAGACGATCAAACTTGAC | 32237 | 0.11979486267811994 | No Hit |
| ACCGAGATCTGATAGCAAGCT | 32030 | 0.11902563673977669 | No Hit |
| AACCCGTAGATCCGAACTTGTGA | 31514 | 0.11710814599492111 | No Hit |
| TTCAAGTAATCCAGGATAGGCT | 31304 | 0.11632777185457288 | No Hit |
| TACCGAGATCTGATAGCAAGC | 30759 | 0.11430251515700254 | No Hit |
| TTTGGCAGGTGAGTAGAGCCGTTCGTGA | 30487 | 0.11329174484188487 | No Hit |
| TTAATGCCGAGAACTGATGACGATCC | 30441 | 0.11312080574447526 | No Hit |
| ATTCTATACCGAGATCTGATAGCAAGCT | 30311 | 0.11263771699092638 | No Hit |
| GCACCCGTAGCTCAGCTGGA | 30077 | 0.11176815723453837 | No Hit |
| ACCCTGTAGAACCGAATTTGT | 29850 | 0.1109246099494953 | No Hit |
| TGGACGGAGAACTGATAAGGG | 29599 | 0.10999187704841246 | No Hit |
| CCGAGAAGACGATCAAACTTGACTAT | 29346 | 0.1090517120126596 | No Hit |
| GCCGAGAACTGATGACGATCCT | 29142 | 0.10829363427632135 | No Hit |
| CCGAGAAGACGATCAAACTT | 28559 | 0.10612716702002131 | No Hit |
| TGAGGTAGTAGATTGAATAGTT | 28363 | 0.10539881782236299 | No Hit |
| TTAATGCCGAGAACTGATGACGATCCT | 28339 | 0.1053096322063232 | No Hit |
| CTCCGGGGATGCGTGCATTTATCAGATC | 27604 | 0.10257832271510446 | No Hit |
| TGAGGTAGTAGGTTGTATAGT | 27537 | 0.10232934620366003 | No Hit |
| TTTAAGTTGAACAGATTGGGAAGTCT | 27458 | 0.10203577688419571 | No Hit |
| GCCGAGAAGACGATCAAACT | 26984 | 0.10027436096740976 | No Hit |

## Adapter Content

Produced by FastQC (version 0.11.9)
