# Supplementary material for: High Throughput Sequencing of MicroRNA in Rainbow Trout Plasma, Mucus, and Surrounding Water Following Acute Stress
Source: Front Physiol. 2021 Jan 13;11:588313. doi: 10.3389/fphys.2020.588313 (PMC7838646; doi:10.3389/fphys.2020.588313)
Supplement: Supplementary file 2 [file Data_Sheet_1.ZIP › Supplemental Quality Control/FastQC_raw_files/mucus_control_1_fastqc_raw.html]

SV18263\_0007\_S19\_R1\_001.fastq FastQC Report 

FastQC Report

Thu 7 May 2020  
SV18263\_0007\_S19\_R1\_001.fastq

## Summary

- Basic Statistics
- Per base sequence quality
- Per tile sequence quality
- Per sequence quality scores
- Per base sequence content
- Per sequence GC content
- Per base N content
- Sequence Length Distribution
- Sequence Duplication Levels
- Overrepresented sequences
- Adapter Content

## Basic Statistics

| Measure | Value |
| --- | --- |
| Filename | SV18263\_0007\_S19\_R1\_001.fastq |
| File type | Conventional base calls |
| Encoding | Sanger / Illumina 1.9 |
| Total Sequences | 17386047 |
| Sequences flagged as poor quality | 0 |
| Sequence length | 51 |
| %GC | 54 |

## Per base sequence quality

## Per tile sequence quality

## Per sequence quality scores

## Per base sequence content

## Per sequence GC content

## Per base N content

## Sequence Length Distribution

## Sequence Duplication Levels

## Overrepresented sequences

| Sequence | Count | Percentage | Possible Source |
| --- | --- | --- | --- |
| TTGGCAGGTGAGTAGAGCCGTTCGTGACATGGAATTCTCGGGTGCCAAGGA | 2004414 | 11.528865647263004 | RNA PCR Primer, Index 1 (100% over 22bp) |
| TTGGCAGGTGAGTAGAGCCGTTCGTGATGGAATTCTCGGGTGCCAAGGAAC | 1380758 | 7.941759273974124 | RNA PCR Primer, Index 1 (100% over 24bp) |
| GCATTGGTGGTTCAGTGGTAGAATTCTCGCCTGGAATTCTCGGGTGCCAAG | 907797 | 5.221411169543025 | No Hit |
| GCATTGGTGGTTCAGTGGTAGAATTCTCGCTGGAATTCTCGGGTGCCAAGG | 884094 | 5.085077706277914 | Illumina Small RNA Adapter 2 (100% over 21bp) |
| CTTTTGGCAGGTGAGTAGAGCCGTTCGTGACATGGAATTCTCGGGTGCCAA | 839611 | 4.829223112073723 | No Hit |
| CCGAGAAGACGATCAAACTTGATGGAATTCTCGGGTGCCAAGGAACTCCAG | 585139 | 3.365566652385099 | RNA PCR Primer, Index 1 (100% over 29bp) |
| CGGCGACTCTGGACGCGTGCCTGGAATTCTCGGGTGCCAAGGAACTCCAGT | 439769 | 2.5294363922978005 | RNA PCR Primer, Index 1 (100% over 30bp) |
| AGCGGCGACTCTGGACGCGTGCCTGGAATTCTCGGGTGCCAAGGAACTCCA | 377580 | 2.1717415120297328 | RNA PCR Primer, Index 1 (100% over 28bp) |
| CTTTTGGCAGGTGAGTAGAGCCGTTCGTGATGGAATTCTCGGGTGCCAAGG | 294874 | 1.6960382081102163 | Illumina Small RNA Adapter 2 (100% over 21bp) |
| GCGGCGACTCTGGACGCGTGCCTGGAATTCTCGGGTGCCAAGGAACTCCAG | 290254 | 1.6694651751487846 | RNA PCR Primer, Index 1 (100% over 29bp) |
| TTTTGGCAGGTGAGTAGAGCCGTTCGTGACATGGAATTCTCGGGTGCCAAG | 250428 | 1.4403964282392656 | No Hit |
| TGAGAACTGAATTCCATAGATGGTGGAATTCTCGGGTGCCAAGGAACTCCA | 238600 | 1.3723648624670115 | RNA PCR Primer, Index 1 (100% over 28bp) |
| GGCGACTCTGGACGCGTGCCTGGAATTCTCGGGTGCCAAGGAACTCCAGTC | 227710 | 1.3097284276293513 | RNA PCR Primer, Index 1 (100% over 31bp) |
| GCCGAGAAGACGATCAAACTTGATGGAATTCTCGGGTGCCAAGGAACTCCA | 203958 | 1.173113129166164 | RNA PCR Primer, Index 1 (100% over 28bp) |
| CGTGGAGCTTCGGTTGGCCCGGGATAGCCTGCCTTGGAATTCTCGGGTGCC | 187590 | 1.0789686695313776 | No Hit |
| GCATTGGTGGTTCAGTGGTAGAATTCTGGAATTCTCGGGTGCCAAGGAACT | 179805 | 1.0341913834697445 | RNA PCR Primer, Index 1 (100% over 25bp) |
| TTTTGGCAGGTGAGTAGAGCCGTTCGTGATGGAATTCTCGGGTGCCAAGGA | 170883 | 0.9828743704650056 | RNA PCR Primer, Index 1 (100% over 22bp) |
| GCATTGGTGGTTCAGTGGTAGAATTCTCTGGAATTCTCGGGTGCCAAGGAA | 120603 | 0.6936769468068273 | RNA PCR Primer, Index 1 (100% over 23bp) |
| GCAGCGGCGACTCTGGACGCGTGCCTGGAATTCTCGGGTGCCAAGGAACTC | 111111 | 0.6390814427224314 | RNA PCR Primer, Index 1 (100% over 26bp) |
| GGAATACCAGGTGCTGTAAGCTTTGGAATTCTCGGGTGCCAAGGAACTCCA | 89601 | 0.5153615425058957 | RNA PCR Primer, Index 1 (100% over 28bp) |
| CAGGTGAGTAGAGCCGTTCGTGACATGGAATTCTCGGGTGCCAAGGAACTC | 75745 | 0.4356654505765457 | RNA PCR Primer, Index 1 (100% over 26bp) |
| GCATTGGTGGTTCAGTGGTAGAATTCTCGTGGAATTCTCGGGTGCCAAGGA | 73632 | 0.4235120266268692 | RNA PCR Primer, Index 1 (100% over 22bp) |
| GATCGGGGGCCTGAGTCCTTGGAATTCTCGGGTGCCAAGGAACTCCAGTCA | 72380 | 0.416310849729096 | RNA PCR Primer, Index 1 (100% over 32bp) |
| TTGGCAGGTGAGTAGAGCCGTTCGTGACTGGAATTCTCGGGTGCCAAGGAA | 69116 | 0.3975371744940066 | RNA PCR Primer, Index 1 (100% over 23bp) |
| TGGGAATACCAGGTGCTGTAAGCTTTGGAATTCTCGGGTGCCAAGGAACTC | 68604 | 0.39459228426105136 | RNA PCR Primer, Index 1 (100% over 26bp) |
| TTTGGCAGGTGAGTAGAGCCGTTCGTGACATGGAATTCTCGGGTGCCAAGG | 67015 | 0.38545277140916506 | Illumina Small RNA Adapter 2 (100% over 21bp) |
| TGATGCGCACCGCATGTTTGTGGAGAACCTGGAATTCTCGGGTGCCAAGGA | 66871 | 0.3846245210311464 | RNA PCR Primer, Index 1 (100% over 22bp) |
| GCATTGGTGGTTCAGTGGTAGAATTCTCGCCTTGGAATTCTCGGGTGCCAA | 63668 | 0.3662017018589677 | No Hit |
| CTTTTGGCAGGTGAGTAGAGCCGTTCGTGACAGTGGAATTCTCGGGTGCCA | 62381 | 0.35879921410542603 | No Hit |
| GTCTGGCGGGCACGGGAAATGTGGTGTATATGGAATTCTCGGGTGCCAAGG | 58144 | 0.33442909707997454 | Illumina Small RNA Adapter 2 (100% over 21bp) |
| AGCGGCGACTCTGGACGCTGGAATTCTCGGGTGCCAAGGAACTCCAGTCAC | 55044 | 0.31659870699762865 | RNA PCR Primer, Index 1 (100% over 33bp) |
| CTCCGGGGATGCGTGCATTTATCAGATCTGGAATTCTCGGGTGCCAAGGAA | 52398 | 0.30137960630153593 | RNA PCR Primer, Index 1 (100% over 23bp) |
| CCCCCCACTGCTAAATTTGACTGGCTTTGGAATTCTCGGGTGCCAAGGAAC | 51350 | 0.29535178410595575 | RNA PCR Primer, Index 1 (100% over 24bp) |
| GTGGTTGGCAGCGGCGACTCTGGACGCGTGCCTGGAATTCTCGGGTGCCAA | 50998 | 0.29332717207079906 | No Hit |
| TTTGGCAGGTGAGTAGAGCCGTTCGTGATGGAATTCTCGGGTGCCAAGGAA | 50071 | 0.287995310262304 | RNA PCR Primer, Index 1 (100% over 23bp) |
| CAGGTGAGTAGAGCCGTTCGTGATGGAATTCTCGGGTGCCAAGGAACTCCA | 48332 | 0.2779930365999816 | RNA PCR Primer, Index 1 (100% over 28bp) |
| CGTGGAGCTTCGGTTGGCCCGGGATAGCCTGCCTGGAATTCTCGGGTGCCA | 43689 | 0.2512877136476164 | No Hit |
| GCGTGTCGGCTGAGGTGGGATCCCGACTGGAATTCTCGGGTGCCAAGGAAC | 43493 | 0.25016037285531323 | RNA PCR Primer, Index 1 (100% over 24bp) |
| CAACGGAATCCCAAAAGCAGCTTGGAATTCTCGGGTGCCAAGGAACTCCAG | 42552 | 0.2447479867045108 | RNA PCR Primer, Index 1 (100% over 29bp) |
| AAATTGATTTTTGGAATAGGGATGGAATTCTCGGGTGCCAAGGAACTCCAG | 41462 | 0.23847859148200856 | RNA PCR Primer, Index 1 (100% over 29bp) |
| GGTGAGTAGAGCCGTTCGTGACATGGAATTCTCGGGTGCCAAGGAACTCCA | 40662 | 0.23387720049301602 | RNA PCR Primer, Index 1 (100% over 28bp) |
| TCCCATATGGTCTAGCGGTTAGGATTCCTGGAATTCTCGGGTGCCAAGGAA | 40563 | 0.23330777835812821 | RNA PCR Primer, Index 1 (100% over 23bp) |
| TTGGCAGGTGAGTAGAGCCGTTCGTGACAGTGGAATTCTCGGGTGCCAAGG | 39448 | 0.22689458966721993 | Illumina Small RNA Adapter 2 (100% over 21bp) |
| TCCCATATGGTCTAGCGGTTAGGATTCCTTGGAATTCTCGGGTGCCAAGGA | 38712 | 0.22266130995734684 | RNA PCR Primer, Index 1 (100% over 22bp) |
| CAGCGGCGACTCTGGACGCGTGCCTGGAATTCTCGGGTGCCAAGGAACTCC | 37911 | 0.21805416722961807 | RNA PCR Primer, Index 1 (100% over 27bp) |
| GCCCGGCTAGCTCAGTCGGTAGAGCATGAGATGGAATTCTCGGGTGCCAAG | 37524 | 0.21582824433869296 | No Hit |
| CAACGGAATCCCAAAAGCAGCTGTGGAATTCTCGGGTGCCAAGGAACTCCA | 37102 | 0.21340101059199945 | RNA PCR Primer, Index 1 (100% over 28bp) |
| GAGGTGTAGAATAAGTGGGAGGCCCTGGAATTCTCGGGTGCCAAGGAACTC | 34661 | 0.1993610163368361 | RNA PCR Primer, Index 1 (100% over 26bp) |
| TCCTGTACTGAGCTGCCCCGAGATGGAATTCTCGGGTGCCAAGGAACTCCA | 30709 | 0.17663014485121314 | RNA PCR Primer, Index 1 (100% over 28bp) |
| TTCAAGTAATCCAGGATAGGCTTGGAATTCTCGGGTGCCAAGGAACTCCAG | 30131 | 0.17330563986166608 | RNA PCR Primer, Index 1 (100% over 29bp) |
| CTGATGCGCACCGCATGTTTGTGGAGAACCTGGAATTCTCGGGTGCCAAGG | 29265 | 0.1683246341160817 | Illumina Small RNA Adapter 2 (100% over 21bp) |
| AATTGATTTTTGGAATAGGGATGGAATTCTCGGGTGCCAAGGAACTCCAGT | 29170 | 0.16777821893613884 | RNA PCR Primer, Index 1 (100% over 30bp) |
| CTTTTGGCAGGTGAGTAGAGCCGTTCGTGACTGGAATTCTCGGGTGCCAAG | 28897 | 0.16620799426114516 | No Hit |
| GCCCGGATAGCTCAGTCGGTAGAGCATCTGGAATTCTCGGGTGCCAAGGAA | 27762 | 0.15967977079551207 | RNA PCR Primer, Index 1 (100% over 23bp) |
| CACCCGTAGAACCGACCTTGCGTGGAATTCTCGGGTGCCAAGGAACTCCAG | 27002 | 0.15530844935596919 | RNA PCR Primer, Index 1 (100% over 29bp) |
| TCCCTGGTGGTCTAGTGGTTAGGATTCGGTGGAATTCTCGGGTGCCAAGGA | 26930 | 0.15489432416695986 | RNA PCR Primer, Index 1 (100% over 22bp) |
| TGAGGTAGTAGATTGTATAGTTTGGAATTCTCGGGTGCCAAGGAACTCCAG | 25199 | 0.14493806441452733 | RNA PCR Primer, Index 1 (100% over 29bp) |
| TACCCTGTAGAACCGAATTTGTTGGAATTCTCGGGTGCCAAGGAACTCCAG | 22243 | 0.12793592471020007 | RNA PCR Primer, Index 1 (100% over 29bp) |
| GTGGAGCTTCGGTTGGCCCGGGATAGCCTGCCTTGGAATTCTCGGGTGCCA | 22224 | 0.1278266416742115 | No Hit |
| CGAGCGGGCTCTCGCTTCTGGTTTCAAGCACTGGAATTCTCGGGTGCCAAG | 21529 | 0.12382918325252429 | No Hit |
| TAAATTGATTTTTGGAATAGGGATGGAATTCTCGGGTGCCAAGGAACTCCA | 21273 | 0.1223567381360467 | RNA PCR Primer, Index 1 (100% over 28bp) |
| TGGAATTCTCGGGTGCCAAGGAACTCCAGTCACCAGGCGATCTCGTATGCC | 21153 | 0.12166652948769781 | RNA PCR Primer, Index 33 (100% over 51bp) |
| TGAGGTAGTAGGTTGTATAGTTTGGAATTCTCGGGTGCCAAGGAACTCCAG | 21074 | 0.1212121421275348 | RNA PCR Primer, Index 1 (100% over 29bp) |
| TCCTGTACTGAGCTGCCCCGAGTGGAATTCTCGGGTGCCAAGGAACTCCAG | 20830 | 0.11980871787589209 | RNA PCR Primer, Index 1 (100% over 29bp) |
| TCCTGTACTGAGCTGCCCCGAGTTGGAATTCTCGGGTGCCAAGGAACTCCA | 19734 | 0.11350481222097238 | RNA PCR Primer, Index 1 (100% over 28bp) |
| GACTCTGGACGCGTGCCTGGAATTCTCGGGTGCCAAGGAACTCCAGTCACC | 19259 | 0.11077273632125809 | RNA PCR Primer, Index 2 (100% over 34bp) |
| TGGCGGGCACGGGAAATGTGGTGTATATGGAATTCTCGGGTGCCAAGGAAC | 18610 | 0.10703985788143792 | RNA PCR Primer, Index 1 (100% over 24bp) |
| GTGTCCGTCGGCGTCCTGGAATTCTCGGGTGCCAAGGAACTCCAGTCACCA | 18343 | 0.10550414363886167 | RNA PCR Primer, Index 7 (100% over 35bp) |
| GCAGCGGCGACTCTGGACGCTGGAATTCTCGGGTGCCAAGGAACTCCAGTC | 18158 | 0.10444007197265716 | RNA PCR Primer, Index 1 (100% over 31bp) |
| CTGGCGGAGCGCCGAGAAGACGATCAAACTTGATGGAATTCTCGGGTGCCA | 17698 | 0.10179427215398647 | No Hit |

## Adapter Content

Produced by FastQC (version 0.11.9)
