# Supplementary material for: High Throughput Sequencing of MicroRNA in Rainbow Trout Plasma, Mucus, and Surrounding Water Following Acute Stress
Source: Front Physiol. 2021 Jan 13;11:588313. doi: 10.3389/fphys.2020.588313 (PMC7838646; doi:10.3389/fphys.2020.588313)
Supplement: Supplementary file 2 [file Data_Sheet_1.ZIP › Supplemental Quality Control/FastQC_raw_files/mucus_control_2_fastqc_raw.html]

SV18263\_0009\_S21\_R1\_001.fastq FastQC Report 

FastQC Report

Thu 7 May 2020  
SV18263\_0009\_S21\_R1\_001.fastq

## Summary

- Basic Statistics
- Per base sequence quality
- Per tile sequence quality
- Per sequence quality scores
- Per base sequence content
- Per sequence GC content
- Per base N content
- Sequence Length Distribution
- Sequence Duplication Levels
- Overrepresented sequences
- Adapter Content

## Basic Statistics

| Measure | Value |
| --- | --- |
| Filename | SV18263\_0009\_S21\_R1\_001.fastq |
| File type | Conventional base calls |
| Encoding | Sanger / Illumina 1.9 |
| Total Sequences | 18648203 |
| Sequences flagged as poor quality | 0 |
| Sequence length | 51 |
| %GC | 54 |

## Per base sequence quality

## Per tile sequence quality

## Per sequence quality scores

## Per base sequence content

## Per sequence GC content

## Per base N content

## Sequence Length Distribution

## Sequence Duplication Levels

## Overrepresented sequences

| Sequence | Count | Percentage | Possible Source |
| --- | --- | --- | --- |
| TGAGAACTGAATTCCATAGATGGTGGAATTCTCGGGTGCCAAGGAACTCCA | 555011 | 2.9762170649901227 | RNA PCR Primer, Index 1 (100% over 28bp) |
| GCGGCGACTCTGGACGCGTGCCTGGAATTCTCGGGTGCCAAGGAACTCCAG | 316579 | 1.6976381048619 | RNA PCR Primer, Index 1 (100% over 29bp) |
| CGGCGACTCTGGACGCGTGCCTGGAATTCTCGGGTGCCAAGGAACTCCAGT | 253516 | 1.3594661104879648 | RNA PCR Primer, Index 1 (100% over 30bp) |
| GTGTCCGTCGGCGTCCTGGAATTCTCGGGTGCCAAGGAACTCCAGTCACCA | 207798 | 1.1143057591125538 | RNA PCR Primer, Index 7 (100% over 35bp) |
| TGTCAACCGGGTCTGGAATTCTCGGGTGCCAAGGAACTCCAGTCACCATTT | 171661 | 0.9205230123245655 | RNA PCR Primer, Index 35 (100% over 38bp) |
| GGCGACTCTGGACGCGTGCCTGGAATTCTCGGGTGCCAAGGAACTCCAGTC | 138957 | 0.7451495460447315 | RNA PCR Primer, Index 1 (100% over 31bp) |
| GCATTGGTGGTTCAGTGGTAGAATTCTCGCTGGAATTCTCGGGTGCCAAGG | 136381 | 0.7313358826048815 | Illumina Small RNA Adapter 2 (100% over 21bp) |
| GTGTCCGTCGGCGTCTGGAATTCTCGGGTGCCAAGGAACTCCAGTCACCAT | 127721 | 0.6848970916929636 | RNA PCR Primer, Index 9 (97% over 36bp) |
| GCAGCGGCGACTCTGGACGCGTGCCTGGAATTCTCGGGTGCCAAGGAACTC | 108471 | 0.5816699871832155 | RNA PCR Primer, Index 1 (100% over 26bp) |
| TAACACTGTCTGGTAACGATGTGGAATTCTCGGGTGCCAAGGAACTCCAGT | 106807 | 0.5727468753959831 | RNA PCR Primer, Index 1 (100% over 30bp) |
| TGAGAACTGAATTCCATAGATGTGGAATTCTCGGGTGCCAAGGAACTCCAG | 104463 | 0.5601772996572377 | RNA PCR Primer, Index 1 (100% over 29bp) |
| CTGTCAACCGGGTCTGGAATTCTCGGGTGCCAAGGAACTCCAGTCACCATT | 102656 | 0.5504873579507902 | RNA PCR Primer, Index 32 (97% over 37bp) |
| AGCGGCGACTCTGGACGCGTGCCTGGAATTCTCGGGTGCCAAGGAACTCCA | 101432 | 0.5439237228380664 | RNA PCR Primer, Index 1 (100% over 28bp) |
| GCATTGGTGGTTCAGTGGTAGAATTCTGGAATTCTCGGGTGCCAAGGAACT | 98276 | 0.5269998401454553 | RNA PCR Primer, Index 1 (100% over 25bp) |
| CTTTTGGCAGGTGAGTAGAGCCGTTCGTGACATGGAATTCTCGGGTGCCAA | 92149 | 0.494144127452924 | No Hit |
| GCGACTCTGGACGCGTGCCTGGAATTCTCGGGTGCCAAGGAACTCCAGTCA | 89951 | 0.4823574689743564 | RNA PCR Primer, Index 1 (100% over 32bp) |
| ATGCGGGCCTGGAATTCTCGGGTGCCAAGGAACTCCAGTCACCATTTTATC | 89199 | 0.4783249088397419 | RNA PCR Primer, Index 35 (100% over 42bp) |
| TCTCGCAAGGGGCTGCTTGGAATTCTCGGGTGCCAAGGAACTCCAGTCACC | 87766 | 0.47064052230662656 | RNA PCR Primer, Index 2 (100% over 34bp) |
| GCATTGGTGGTTCAGTGGTAGAATTCTCGCCTGGAATTCTCGGGTGCCAAG | 84716 | 0.4542850589946924 | No Hit |
| TGTCAACCGGGTCGGACTGTCCTCAGTGCGTATGGAATTCTCGGGTGCCAA | 74964 | 0.40199047597240334 | No Hit |
| GACTCTGGACGCGTGCCTGGAATTCTCGGGTGCCAAGGAACTCCAGTCACC | 73241 | 0.3927509798129074 | RNA PCR Primer, Index 2 (100% over 34bp) |
| TTGGCAGGTGAGTAGAGCCGTTCGTGATGGAATTCTCGGGTGCCAAGGAAC | 71852 | 0.38530254094724303 | RNA PCR Primer, Index 1 (100% over 24bp) |
| GAGGTGTAGAATAAGTGGGAGGCCCTGGAATTCTCGGGTGCCAAGGAACTC | 64826 | 0.3476259884129318 | RNA PCR Primer, Index 1 (100% over 26bp) |
| CAGTCGGTAGAGCATCTGGAATTCTCGGGTGCCAAGGAACTCCAGTCACCA | 61727 | 0.3310077651986092 | RNA PCR Primer, Index 7 (100% over 35bp) |
| CCGAGAAGACGATCAAACTTGATGGAATTCTCGGGTGCCAAGGAACTCCAG | 59432 | 0.31870094936225224 | RNA PCR Primer, Index 1 (100% over 29bp) |
| AGCGGCGACTCTGGACGCTGGAATTCTCGGGTGCCAAGGAACTCCAGTCAC | 57947 | 0.31073771558578595 | RNA PCR Primer, Index 1 (100% over 33bp) |
| TACCCTGTAGAACCGAATTTGTTGGAATTCTCGGGTGCCAAGGAACTCCAG | 56640 | 0.3037289973730981 | RNA PCR Primer, Index 1 (100% over 29bp) |
| TCTTTTGGCAGGTGAGTAGAGCCGTTCGTGACATGGAATTCTCGGGTGCCA | 54152 | 0.29038722926814986 | No Hit |
| TGTCCGTCGGCGTCCTGGAATTCTCGGGTGCCAAGGAACTCCAGTCACCAT | 51106 | 0.2740532157441658 | RNA PCR Primer, Index 9 (97% over 36bp) |
| GCATTGGTGGTTCAGTGGTAGAATTCTCTGGAATTCTCGGGTGCCAAGGAA | 50590 | 0.2712861930986058 | RNA PCR Primer, Index 1 (100% over 23bp) |
| AGGTGTAGAATAAGTGGGAGGCCCCGTGGAATTCTCGGGTGCCAAGGAACT | 44358 | 0.23786742347238496 | RNA PCR Primer, Index 1 (100% over 25bp) |
| TGTCCGTCGGCGTCTGGAATTCTCGGGTGCCAAGGAACTCCAGTCACCATT | 42862 | 0.22984520277905596 | RNA PCR Primer, Index 32 (97% over 37bp) |
| GCCGAGAAGACGATCAAACTTGATGGAATTCTCGGGTGCCAAGGAACTCCA | 42188 | 0.2262309135094679 | RNA PCR Primer, Index 1 (100% over 28bp) |
| AGCGGCGACTCTGGACGCGTGCCGGGTGGAATTCTCGGGTGCCAAGGAACT | 40346 | 0.2163532861584572 | RNA PCR Primer, Index 1 (100% over 25bp) |
| CAGGTGAGTAGAGCCGTTCGTGACATGGAATTCTCGGGTGCCAAGGAACTC | 40321 | 0.2162192249837692 | RNA PCR Primer, Index 1 (100% over 26bp) |
| TGTCAACCGGGTCGGACTGTCCTCAGTGCGTACTGGAATTCTCGGGTGCCA | 38912 | 0.2086635371783544 | No Hit |
| CTTTTGGCAGGTGAGTAGAGCCGTTCGTGATGGAATTCTCGGGTGCCAAGG | 38848 | 0.20832034057115317 | Illumina Small RNA Adapter 2 (100% over 21bp) |
| ATCTCGTGGGCTCTCGTTTGTGGTGGAATTCTCGGGTGCCAAGGAACTCCA | 37092 | 0.19890388366106912 | RNA PCR Primer, Index 1 (100% over 28bp) |
| GGTGAGTAGAGCCGTTCGTGACATGGAATTCTCGGGTGCCAAGGAACTCCA | 36204 | 0.19414203073615188 | RNA PCR Primer, Index 1 (100% over 28bp) |
| GGCGACTCTGGACGCGTGCCGGTGGAATTCTCGGGTGCCAAGGAACTCCAG | 36000 | 0.19304809155069796 | RNA PCR Primer, Index 1 (100% over 29bp) |
| GGAATACCAGGTGCTGTAAGCTTTGGAATTCTCGGGTGCCAAGGAACTCCA | 35158 | 0.1885329111872066 | RNA PCR Primer, Index 1 (100% over 28bp) |
| AGGTGTAGAATAAGTGGGAGGCCCTGGAATTCTCGGGTGCCAAGGAACTCC | 33987 | 0.18225348576482142 | RNA PCR Primer, Index 1 (100% over 27bp) |
| TCTCGCAAGGGGCTGCTTTGGAATTCTCGGGTGCCAAGGAACTCCAGTCAC | 33087 | 0.17742728347605397 | RNA PCR Primer, Index 1 (100% over 33bp) |
| CAGCGGCGACTCTGGACGCGTGCCTGGAATTCTCGGGTGCCAAGGAACTCC | 32309 | 0.1732552997197639 | RNA PCR Primer, Index 1 (100% over 27bp) |
| GCAGCGGCGACTCTGGACGCTGGAATTCTCGGGTGCCAAGGAACTCCAGTC | 32134 | 0.172316871496948 | RNA PCR Primer, Index 1 (100% over 31bp) |
| GCATTGGTGGTTCAGTGGTAGAATTCTCGCCTTGGAATTCTCGGGTGCCAA | 29967 | 0.16069644887499349 | No Hit |
| TGGCGGGCACGGGAAATGTGGTGTATATGGAATTCTCGGGTGCCAAGGAAC | 29622 | 0.15884640466429928 | RNA PCR Primer, Index 1 (100% over 24bp) |
| CATGCGGGCCTGGAATTCTCGGGTGCCAAGGAACTCCAGTCACCATTTTAT | 29214 | 0.15665852629339136 | RNA PCR Primer, Index 35 (100% over 41bp) |
| TCGAGCCGCGGCTGGGGGAGCTGGAATTCTCGGGTGCCAAGGAACTCCAGT | 28863 | 0.15477630740077208 | RNA PCR Primer, Index 1 (100% over 30bp) |
| TGAGAACTGAATTCCATAGATTGGAATTCTCGGGTGCCAAGGAACTCCAGT | 28741 | 0.1541220888682947 | RNA PCR Primer, Index 1 (100% over 30bp) |
| TCGAGCTGGCGGTCCTGGAATTCTCGGGTGCCAAGGAACTCCAGTCACCAT | 28725 | 0.1540362897164944 | RNA PCR Primer, Index 9 (97% over 36bp) |
| TAACGGAACCCATAATGCAGCTGTGGAATTCTCGGGTGCCAAGGAACTCCA | 28269 | 0.15159101389018556 | RNA PCR Primer, Index 1 (100% over 28bp) |
| TCCCATATGGTCTAGCGGTTAGGATTCCTGTGGAATTCTCGGGTGCCAAGG | 27925 | 0.1497463321264789 | Illumina Small RNA Adapter 2 (100% over 21bp) |
| GTTGTGCGGCCCTGGAATTCTCGGGTGCCAAGGAACTCCAGTCACCATTTT | 26993 | 0.14474853153411082 | RNA PCR Primer, Index 35 (100% over 39bp) |
| TGTGGTCGGATCCCCTCGTGGTGGAATTCTCGGGTGCCAAGGAACTCCAGT | 26928 | 0.14439997247992206 | RNA PCR Primer, Index 1 (100% over 30bp) |
| GCTACGGTGCTCGTGGAATTCTCGGGTGCCAAGGAACTCCAGTCACCATTT | 26910 | 0.1443034484341467 | RNA PCR Primer, Index 35 (100% over 38bp) |
| GTGGTTGGCAGCGGCGACTCTGGACGCGTGCCTGGAATTCTCGGGTGCCAA | 26893 | 0.1442122868353589 | No Hit |
| TAGCTTATCAGACTGGTGTTGGTGGAATTCTCGGGTGCCAAGGAACTCCAG | 26711 | 0.14323632148363036 | RNA PCR Primer, Index 1 (100% over 29bp) |
| TCTTTTGGCAGGTGAGTAGAGCCGTTCGTGATGGAATTCTCGGGTGCCAAG | 26634 | 0.14282341306559138 | No Hit |
| TCAGTCGGTAGAGCATCTGGAATTCTCGGGTGCCAAGGAACTCCAGTCACC | 26534 | 0.14228716836683944 | RNA PCR Primer, Index 2 (100% over 34bp) |
| TGAGAACTGAATTCCATAGATGGTTGGAATTCTCGGGTGCCAAGGAACTCC | 26394 | 0.14153642578858672 | RNA PCR Primer, Index 1 (100% over 27bp) |
| TCGAGCCGCGGCTGGGGGAGCAGTTTGGAATTCTCGGGTGCCAAGGAACTC | 25771 | 0.13819562131536212 | RNA PCR Primer, Index 1 (100% over 26bp) |
| CTCAGTCGGTAGAGCATCTGGAATTCTCGGGTGCCAAGGAACTCCAGTCAC | 25707 | 0.1378524247081609 | RNA PCR Primer, Index 1 (100% over 33bp) |
| TTGGCAGGTGAGTAGAGCCGTTCGTGACATGGAATTCTCGGGTGCCAAGGA | 25139 | 0.13480655481924989 | RNA PCR Primer, Index 1 (100% over 22bp) |
| CTCGCAAGGGGCTGCTTGGAATTCTCGGGTGCCAAGGAACTCCAGTCACCA | 25104 | 0.13461886917468668 | RNA PCR Primer, Index 7 (100% over 35bp) |
| TAACACTGTCTGGTAATGATGTGGAATTCTCGGGTGCCAAGGAACTCCAGT | 24259 | 0.13008760147023282 | RNA PCR Primer, Index 1 (100% over 30bp) |
| GTCTGGCGGGCACGGGAAATGTGGTGTATATGGAATTCTCGGGTGCCAAGG | 24255 | 0.13006615168228275 | Illumina Small RNA Adapter 2 (100% over 21bp) |
| TCCCTGGTGGTCTAGTGGTTAGGATTCGGCTGGAATTCTCGGGTGCCAAGG | 24100 | 0.12923497239921725 | Illumina Small RNA Adapter 2 (100% over 21bp) |
| GAGGTGTAGAATAAGTGGGAGGCCCCGTGGAATTCTCGGGTGCCAAGGAAC | 24039 | 0.12890786313297856 | RNA PCR Primer, Index 1 (100% over 24bp) |
| TTTTGGCAGGTGAGTAGAGCCGTTCGTGACATGGAATTCTCGGGTGCCAAG | 23997 | 0.12868264035950275 | No Hit |
| GGGGAATTAGCTCAAATGGTAGATGGAATTCTCGGGTGCCAAGGAACTCCA | 22926 | 0.12293945963586948 | RNA PCR Primer, Index 1 (100% over 28bp) |
| GCCGCGGCTGGGGGAGCTGGAATTCTCGGGTGCCAAGGAACTCCAGTCACC | 21743 | 0.11659568484963403 | RNA PCR Primer, Index 2 (100% over 34bp) |
| ATGCGGGCCTGGAATTCTCGGGTGCCAAGGAACTCCAGTCACCATTTTATG | 21695 | 0.1163382873942331 | RNA PCR Primer, Index 35 (97% over 41bp) |
| TCGGGCTGGGGTGCGAAGCGGGGCTTGGAATTCTCGGGTGCCAAGGAACTC | 21278 | 0.11410214700043751 | RNA PCR Primer, Index 1 (100% over 26bp) |
| TCCCATATGGTCTAGCGGTTAGGATTCCTTGGAATTCTCGGGTGCCAAGGA | 20744 | 0.11123860030910217 | RNA PCR Primer, Index 1 (100% over 22bp) |
| GTGGTTGGCAGCGGCGACTCTGGACGCTGGAATTCTCGGGTGCCAAGGAAC | 20150 | 0.10805330679851566 | RNA PCR Primer, Index 1 (100% over 24bp) |
| CGCGTGTCGGCTGAGGTGGGATCCCGTGGAATTCTCGGGTGCCAAGGAACT | 20047 | 0.10750097475880115 | RNA PCR Primer, Index 1 (100% over 25bp) |
| TAATACTGCCTGGTAATGATGATGGAATTCTCGGGTGCCAAGGAACTCCAG | 19948 | 0.10697009250703673 | RNA PCR Primer, Index 1 (100% over 29bp) |
| GCATTGGTGGTTCAGTGGTAGAATTCTCGTGGAATTCTCGGGTGCCAAGGA | 19568 | 0.10493236265177935 | RNA PCR Primer, Index 1 (100% over 22bp) |
| AACCCGTAGATCCGAACTTGTGTGGAATTCTCGGGTGCCAAGGAACTCCAG | 19461 | 0.1043585808241148 | RNA PCR Primer, Index 1 (100% over 29bp) |
| CTACGGTGCTCGCTGGAATTCTCGGGTGCCAAGGAACTCCAGTCACCATTT | 19437 | 0.10422988209641433 | RNA PCR Primer, Index 35 (100% over 38bp) |
| CGAGAAGACGATCAAACTTGATGGAATTCTCGGGTGCCAAGGAACTCCAGT | 19394 | 0.10399929687595098 | RNA PCR Primer, Index 1 (100% over 30bp) |
| CGACTCTGGACGCGTGCCTGGAATTCTCGGGTGCCAAGGAACTCCAGTCAC | 19067 | 0.10224577671103215 | RNA PCR Primer, Index 1 (100% over 33bp) |

## Adapter Content

Produced by FastQC (version 0.11.9)
