# Supplementary material for: High Throughput Sequencing of MicroRNA in Rainbow Trout Plasma, Mucus, and Surrounding Water Following Acute Stress
Source: Front Physiol. 2021 Jan 13;11:588313. doi: 10.3389/fphys.2020.588313 (PMC7838646; doi:10.3389/fphys.2020.588313)
Supplement: Supplementary file 2 [file Data_Sheet_1.ZIP › Supplemental Quality Control/FastQC_raw_files/mucus_control_3_fastqc_raw.html]

SV18263\_0011\_S23\_R1\_001.fastq FastQC Report 

FastQC Report

Thu 7 May 2020  
SV18263\_0011\_S23\_R1\_001.fastq

## Summary

- Basic Statistics
- Per base sequence quality
- Per tile sequence quality
- Per sequence quality scores
- Per base sequence content
- Per sequence GC content
- Per base N content
- Sequence Length Distribution
- Sequence Duplication Levels
- Overrepresented sequences
- Adapter Content

## Basic Statistics

| Measure | Value |
| --- | --- |
| Filename | SV18263\_0011\_S23\_R1\_001.fastq |
| File type | Conventional base calls |
| Encoding | Sanger / Illumina 1.9 |
| Total Sequences | 17616935 |
| Sequences flagged as poor quality | 0 |
| Sequence length | 51 |
| %GC | 55 |

## Per base sequence quality

## Per tile sequence quality

## Per sequence quality scores

## Per base sequence content

## Per sequence GC content

## Per base N content

## Sequence Length Distribution

## Sequence Duplication Levels

## Overrepresented sequences

| Sequence | Count | Percentage | Possible Source |
| --- | --- | --- | --- |
| CTTTTGGCAGGTGAGTAGAGCCGTTCGTGACATGGAATTCTCGGGTGCCAA | 1414314 | 8.028150186170297 | No Hit |
| CCGAGAAGACGATCAAACTTGATGGAATTCTCGGGTGCCAAGGAACTCCAG | 1156761 | 6.566187591655416 | RNA PCR Primer, Index 1 (100% over 29bp) |
| AGCGGCGACTCTGGACGCGTGCCTGGAATTCTCGGGTGCCAAGGAACTCCA | 806814 | 4.579763733021664 | RNA PCR Primer, Index 1 (100% over 28bp) |
| GCGGCGACTCTGGACGCGTGCCTGGAATTCTCGGGTGCCAAGGAACTCCAG | 678483 | 3.8513112524965325 | RNA PCR Primer, Index 1 (100% over 29bp) |
| CGGCGACTCTGGACGCGTGCCTGGAATTCTCGGGTGCCAAGGAACTCCAGT | 606798 | 3.444401651024994 | RNA PCR Primer, Index 1 (100% over 30bp) |
| GCATTGGTGGTTCAGTGGTAGAATTCTCGCCTGGAATTCTCGGGTGCCAAG | 570102 | 3.236102080185912 | No Hit |
| GCAGCGGCGACTCTGGACGCGTGCCTGGAATTCTCGGGTGCCAAGGAACTC | 432153 | 2.4530544047531535 | RNA PCR Primer, Index 1 (100% over 26bp) |
| GCATTGGTGGTTCAGTGGTAGAATTCTCGCTGGAATTCTCGGGTGCCAAGG | 417088 | 2.367540097071369 | Illumina Small RNA Adapter 2 (100% over 21bp) |
| TTGGCAGGTGAGTAGAGCCGTTCGTGACATGGAATTCTCGGGTGCCAAGGA | 380398 | 2.1592745843700962 | RNA PCR Primer, Index 1 (100% over 22bp) |
| GGCGACTCTGGACGCGTGCCTGGAATTCTCGGGTGCCAAGGAACTCCAGTC | 349708 | 1.9850672094776984 | RNA PCR Primer, Index 1 (100% over 31bp) |
| TGAGAACTGAATTCCATAGATGGTGGAATTCTCGGGTGCCAAGGAACTCCA | 268126 | 1.52197870969042 | RNA PCR Primer, Index 1 (100% over 28bp) |
| GTGGTTGGCAGCGGCGACTCTGGACGCGTGCCTGGAATTCTCGGGTGCCAA | 223789 | 1.2703061003517355 | No Hit |
| GCCGAGAAGACGATCAAACTTGATGGAATTCTCGGGTGCCAAGGAACTCCA | 209445 | 1.1888844455633172 | RNA PCR Primer, Index 1 (100% over 28bp) |
| TTTTGGCAGGTGAGTAGAGCCGTTCGTGACATGGAATTCTCGGGTGCCAAG | 154588 | 0.8774965679330713 | No Hit |
| CTTTTGGCAGGTGAGTAGAGCCGTTCGTGATGGAATTCTCGGGTGCCAAGG | 151601 | 0.8605412916605527 | Illumina Small RNA Adapter 2 (100% over 21bp) |
| CTTTTGGCAGGTGAGTAGAGCCGTTCGTGACAGTGGAATTCTCGGGTGCCA | 150582 | 0.8547570845893453 | No Hit |
| CGAGAAGACGATCAAACTTGATGGAATTCTCGGGTGCCAAGGAACTCCAGT | 114632 | 0.650692075551167 | RNA PCR Primer, Index 1 (100% over 30bp) |
| GCATTGGTGGTTCAGTGGTAGAATTCTCGCCTTGGAATTCTCGGGTGCCAA | 108506 | 0.6159187168483053 | No Hit |
| TTGGCAGGTGAGTAGAGCCGTTCGTGATGGAATTCTCGGGTGCCAAGGAAC | 103634 | 0.5882635089474986 | RNA PCR Primer, Index 1 (100% over 24bp) |
| GCATTGGTGGTTCAGTGGTAGAATTCTGGAATTCTCGGGTGCCAAGGAACT | 99961 | 0.5674142522521652 | RNA PCR Primer, Index 1 (100% over 25bp) |
| CAGCGGCGACTCTGGACGCGTGCCTGGAATTCTCGGGTGCCAAGGAACTCC | 82583 | 0.4687705324450593 | RNA PCR Primer, Index 1 (100% over 27bp) |
| GACTCTGGACGCGTGCCTGGAATTCTCGGGTGCCAAGGAACTCCAGTCACC | 76805 | 0.43597254573511224 | RNA PCR Primer, Index 2 (100% over 34bp) |
| AGCGGCGACTCTGGACGCTGGAATTCTCGGGTGCCAAGGAACTCCAGTCAC | 65513 | 0.3718751303788088 | RNA PCR Primer, Index 1 (100% over 33bp) |
| GCATTGGTGGTTCAGTGGTAGAATTCTCTGGAATTCTCGGGTGCCAAGGAA | 65233 | 0.3702857506143946 | RNA PCR Primer, Index 1 (100% over 23bp) |
| CCGAGAAGACGATCAAACTTGACTGGAATTCTCGGGTGCCAAGGAACTCCA | 64045 | 0.3635422393282373 | RNA PCR Primer, Index 1 (100% over 28bp) |
| CAGGTGAGTAGAGCCGTTCGTGACATGGAATTCTCGGGTGCCAAGGAACTC | 63463 | 0.3602385999607764 | RNA PCR Primer, Index 1 (100% over 26bp) |
| CCGAGAAGACGATCAAACTTGACTATTGGAATTCTCGGGTGCCAAGGAACT | 61817 | 0.3508953174885416 | RNA PCR Primer, Index 1 (100% over 25bp) |
| GGTTGGCAGCGGCGACTCTGGACGCGTGCCTGGAATTCTCGGGTGCCAAGG | 61766 | 0.3506058233171661 | Illumina Small RNA Adapter 2 (100% over 21bp) |
| GCGTGTCGGCTGAGGTGGGATCCCGACTGGAATTCTCGGGTGCCAAGGAAC | 54701 | 0.3105023660472154 | RNA PCR Primer, Index 1 (100% over 24bp) |
| GGAATACCAGGTGCTGTAAGCTTTGGAATTCTCGGGTGCCAAGGAACTCCA | 50625 | 0.28736553776238605 | RNA PCR Primer, Index 1 (100% over 28bp) |
| GATCGGGGGCCTGAGTCCTTGGAATTCTCGGGTGCCAAGGAACTCCAGTCA | 49232 | 0.2794583734344254 | RNA PCR Primer, Index 1 (100% over 32bp) |
| CTTTTGGCAGGTGAGTAGAGCCGTTCGTGACTGGAATTCTCGGGTGCCAAG | 43293 | 0.2457464933599403 | No Hit |
| CGAGAAGACGATCAAACTTGACTGGAATTCTCGGGTGCCAAGGAACTCCAG | 43277 | 0.24565567165911664 | RNA PCR Primer, Index 1 (100% over 29bp) |
| CGAGAAGACGATCAAACTTGACTATTGGAATTCTCGGGTGCCAAGGAACTC | 42161 | 0.2393208580266658 | RNA PCR Primer, Index 1 (100% over 26bp) |
| GCATTGGTGGTTCAGTGGTAGAATTCTCGTGGAATTCTCGGGTGCCAAGGA | 40814 | 0.2316748060885733 | RNA PCR Primer, Index 1 (100% over 22bp) |
| GCAGCGGCGACTCTGGACGCTGGAATTCTCGGGTGCCAAGGAACTCCAGTC | 39955 | 0.22679881602560262 | RNA PCR Primer, Index 1 (100% over 31bp) |
| AGCGGCGACTCTGGACGCGTGCCGTGGAATTCTCGGGTGCCAAGGAACTCC | 39931 | 0.22666258347436713 | RNA PCR Primer, Index 1 (100% over 27bp) |
| GGTGAGTAGAGCCGTTCGTGACATGGAATTCTCGGGTGCCAAGGAACTCCA | 39368 | 0.22346679487663434 | RNA PCR Primer, Index 1 (100% over 28bp) |
| CGTGGAGCTTCGGTTGGCCCGGGATAGCCTGCCTTGGAATTCTCGGGTGCC | 36871 | 0.20929293319184067 | No Hit |
| TCTTTTGGCAGGTGAGTAGAGCCGTTCGTGACATGGAATTCTCGGGTGCCA | 34548 | 0.19610675750350443 | No Hit |
| TCGGGCTGGGGTGCGAAGCTGGAATTCTCGGGTGCCAAGGAACTCCAGTCA | 34226 | 0.19427897077442813 | RNA PCR Primer, Index 1 (100% over 32bp) |
| TGGGAATACCAGGTGCTGTAAGCTTTGGAATTCTCGGGTGCCAAGGAACTC | 30793 | 0.17479203959144993 | RNA PCR Primer, Index 1 (100% over 26bp) |
| TTTTGGCAGGTGAGTAGAGCCGTTCGTGATGGAATTCTCGGGTGCCAAGGA | 29420 | 0.16699840238951894 | RNA PCR Primer, Index 1 (100% over 22bp) |
| GCGGCGACTCTGGACGCGTGCCGTGGAATTCTCGGGTGCCAAGGAACTCCA | 27368 | 0.15535051925888357 | RNA PCR Primer, Index 1 (100% over 28bp) |
| GCAGCGGCGACTCTGGACGCGTGTGGAATTCTCGGGTGCCAAGGAACTCCA | 26580 | 0.15087755049331794 | RNA PCR Primer, Index 1 (100% over 28bp) |
| TCTGGACGCGTGCCTGGAATTCTCGGGTGCCAAGGAACTCCAGTCACCGGA | 26254 | 0.1490270583390357 | RNA PCR Primer, Index 7 (97% over 37bp) |
| GCGACTCTGGACGCGTGCCTGGAATTCTCGGGTGCCAAGGAACTCCAGTCA | 23940 | 0.13589196985741278 | RNA PCR Primer, Index 1 (100% over 32bp) |
| GTGTCCGTCGGCGTCCCGAAGGTGGATCTGGAATTCTCGGGTGCCAAGGAA | 23560 | 0.13373495446285066 | RNA PCR Primer, Index 1 (100% over 23bp) |
| TTTGGCAGGTGAGTAGAGCCGTTCGTGACATGGAATTCTCGGGTGCCAAGG | 22896 | 0.12996585387866846 | Illumina Small RNA Adapter 2 (100% over 21bp) |
| GGCGGCGACTCTGGACGCGTGCCTGGAATTCTCGGGTGCCAAGGAACTCCA | 21473 | 0.1218883988616635 | RNA PCR Primer, Index 1 (100% over 28bp) |
| CGAGCGGGCTCTCGCTTCTGGTTTCAAGCACTGGAATTCTCGGGTGCCAAG | 21111 | 0.11983355788052802 | No Hit |
| CTGGCGGAGCGCCGAGAAGACGATCAAACTTGATGGAATTCTCGGGTGCCA | 21011 | 0.1192659222503801 | No Hit |
| GCCCGGCTAGCTCAGTCGGTAGAGCATGAGATGGAATTCTCGGGTGCCAAG | 20723 | 0.1176311316355541 | No Hit |
| CCGAGAAGACGATCAAACTTGGAATTCTCGGGTGCCAAGGAACTCCAGTCA | 20624 | 0.11706917236170764 | RNA PCR Primer, Index 1 (100% over 32bp) |
| CCTGGCGGAGCGCCGAGAAGACGATCAAACTTGATGGAATTCTCGGGTGCC | 20458 | 0.11612689721566208 | No Hit |
| GCAGCGGCGACTCTGGACGCGTGCTGGAATTCTCGGGTGCCAAGGAACTCC | 20367 | 0.11561034879222748 | RNA PCR Primer, Index 1 (100% over 27bp) |
| AGCGGCGACTCTGGACGCGTGCTGGAATTCTCGGGTGCCAAGGAACTCCAG | 18836 | 0.10691984729466278 | RNA PCR Primer, Index 1 (100% over 29bp) |
| CCGAGAAGACGATCAAACTTGTTGGAATTCTCGGGTGCCAAGGAACTCCAG | 18804 | 0.10673820389301544 | RNA PCR Primer, Index 1 (100% over 29bp) |
| TACCCTGTAGAACCGAATTTGTTGGAATTCTCGGGTGCCAAGGAACTCCAG | 18374 | 0.10429737068337938 | RNA PCR Primer, Index 1 (100% over 29bp) |
| GAGAAGACGATCAAACTTGATGGAATTCTCGGGTGCCAAGGAACTCCAGTC | 18299 | 0.10387164396076844 | RNA PCR Primer, Index 1 (100% over 31bp) |
| TGGTTGGCAGCGGCGACTCTGGACGCGTGCCTGGAATTCTCGGGTGCCAAG | 17912 | 0.10167489407209597 | No Hit |
| CCGAGAAGACGATCAAACTTGTGGAATTCTCGGGTGCCAAGGAACTCCAGT | 17848 | 0.1013116072688013 | RNA PCR Primer, Index 1 (100% over 30bp) |

## Adapter Content

Produced by FastQC (version 0.11.9)
