# Supplementary material for: High Throughput Sequencing of MicroRNA in Rainbow Trout Plasma, Mucus, and Surrounding Water Following Acute Stress
Source: Front Physiol. 2021 Jan 13;11:588313. doi: 10.3389/fphys.2020.588313 (PMC7838646; doi:10.3389/fphys.2020.588313)
Supplement: Supplementary file 2 [file Data_Sheet_1.ZIP › Supplemental Quality Control/FastQC_raw_files/mucus_stressed_1_fastqc_raw.html]

SV18263\_0008\_S20\_R1\_001.fastq FastQC Report 

FastQC Report

Thu 7 May 2020  
SV18263\_0008\_S20\_R1\_001.fastq

## Summary

- Basic Statistics
- Per base sequence quality
- Per tile sequence quality
- Per sequence quality scores
- Per base sequence content
- Per sequence GC content
- Per base N content
- Sequence Length Distribution
- Sequence Duplication Levels
- Overrepresented sequences
- Adapter Content

## Basic Statistics

| Measure | Value |
| --- | --- |
| Filename | SV18263\_0008\_S20\_R1\_001.fastq |
| File type | Conventional base calls |
| Encoding | Sanger / Illumina 1.9 |
| Total Sequences | 20280680 |
| Sequences flagged as poor quality | 0 |
| Sequence length | 51 |
| %GC | 56 |

## Per base sequence quality

## Per tile sequence quality

## Per sequence quality scores

## Per base sequence content

## Per sequence GC content

## Per base N content

## Sequence Length Distribution

## Sequence Duplication Levels

## Overrepresented sequences

| Sequence | Count | Percentage | Possible Source |
| --- | --- | --- | --- |
| CCGAGAAGACGATCAAACTTGATGGAATTCTCGGGTGCCAAGGAACTCCAG | 2301459 | 11.348036653603332 | RNA PCR Primer, Index 1 (100% over 29bp) |
| AGCGGCGACTCTGGACGCGTGCCTGGAATTCTCGGGTGCCAAGGAACTCCA | 1651743 | 8.144416262176614 | RNA PCR Primer, Index 1 (100% over 28bp) |
| GCGGCGACTCTGGACGCGTGCCTGGAATTCTCGGGTGCCAAGGAACTCCAG | 1146296 | 5.652157619961461 | RNA PCR Primer, Index 1 (100% over 29bp) |
| GCAGCGGCGACTCTGGACGCGTGCCTGGAATTCTCGGGTGCCAAGGAACTC | 873853 | 4.308795365835859 | RNA PCR Primer, Index 1 (100% over 26bp) |
| CGGCGACTCTGGACGCGTGCCTGGAATTCTCGGGTGCCAAGGAACTCCAGT | 779580 | 3.8439539502620224 | RNA PCR Primer, Index 1 (100% over 30bp) |
| GGCGACTCTGGACGCGTGCCTGGAATTCTCGGGTGCCAAGGAACTCCAGTC | 545388 | 2.689199770421899 | RNA PCR Primer, Index 1 (100% over 31bp) |
| GCATTGGTGGTTCAGTGGTAGAATTCTCGCCTGGAATTCTCGGGTGCCAAG | 434205 | 2.1409785076240047 | No Hit |
| TGAGAACTGAATTCCATAGATGGTGGAATTCTCGGGTGCCAAGGAACTCCA | 422304 | 2.0822970432944063 | RNA PCR Primer, Index 1 (100% over 28bp) |
| CGAGAAGACGATCAAACTTGATGGAATTCTCGGGTGCCAAGGAACTCCAGT | 368267 | 1.8158513422626854 | RNA PCR Primer, Index 1 (100% over 30bp) |
| GCATTGGTGGTTCAGTGGTAGAATTCTCGCTGGAATTCTCGGGTGCCAAGG | 284217 | 1.4014175067108203 | Illumina Small RNA Adapter 2 (100% over 21bp) |
| GTGGTTGGCAGCGGCGACTCTGGACGCGTGCCTGGAATTCTCGGGTGCCAA | 266404 | 1.3135851460601913 | No Hit |
| GCCGAGAAGACGATCAAACTTGATGGAATTCTCGGGTGCCAAGGAACTCCA | 248170 | 1.2236769181309501 | RNA PCR Primer, Index 1 (100% over 28bp) |
| CTTTTGGCAGGTGAGTAGAGCCGTTCGTGACATGGAATTCTCGGGTGCCAA | 246287 | 1.21439221959027 | No Hit |
| CAGCGGCGACTCTGGACGCGTGCCTGGAATTCTCGGGTGCCAAGGAACTCC | 162238 | 0.7999633148395419 | RNA PCR Primer, Index 1 (100% over 27bp) |
| CCGAGAAGACGATCAAACTTGACTGGAATTCTCGGGTGCCAAGGAACTCCA | 159711 | 0.7875031803667334 | RNA PCR Primer, Index 1 (100% over 28bp) |
| TTGGCAGGTGAGTAGAGCCGTTCGTGACATGGAATTCTCGGGTGCCAAGGA | 150309 | 0.7411437880781118 | RNA PCR Primer, Index 1 (100% over 22bp) |
| CCGAGAAGACGATCAAACTTGACTATTGGAATTCTCGGGTGCCAAGGAACT | 134602 | 0.6636956946216793 | RNA PCR Primer, Index 1 (100% over 25bp) |
| CGAGAAGACGATCAAACTTGACTGGAATTCTCGGGTGCCAAGGAACTCCAG | 124828 | 0.6155020443101513 | RNA PCR Primer, Index 1 (100% over 29bp) |
| GGTTGGCAGCGGCGACTCTGGACGCGTGCCTGGAATTCTCGGGTGCCAAGG | 120987 | 0.5965628371435278 | Illumina Small RNA Adapter 2 (100% over 21bp) |
| GCATTGGTGGTTCAGTGGTAGAATTCTCGCCTTGGAATTCTCGGGTGCCAA | 104526 | 0.5153969196299137 | No Hit |
| CGAGAAGACGATCAAACTTGACTATTGGAATTCTCGGGTGCCAAGGAACTC | 99803 | 0.49210874586059244 | RNA PCR Primer, Index 1 (100% over 26bp) |
| CCGAGAAGACGATCAAACTTGGAATTCTCGGGTGCCAAGGAACTCCAGTCA | 91484 | 0.45108941120317464 | RNA PCR Primer, Index 1 (100% over 32bp) |
| GACTCTGGACGCGTGCCTGGAATTCTCGGGTGCCAAGGAACTCCAGTCACC | 82936 | 0.4089409230854192 | RNA PCR Primer, Index 2 (100% over 34bp) |
| AGCGGCGACTCTGGACGCGTGCCGTGGAATTCTCGGGTGCCAAGGAACTCC | 78016 | 0.3846813814921393 | RNA PCR Primer, Index 1 (100% over 27bp) |
| GCATTGGTGGTTCAGTGGTAGAATTCTGGAATTCTCGGGTGCCAAGGAACT | 74236 | 0.3660429531948633 | RNA PCR Primer, Index 1 (100% over 25bp) |
| AGCGGCGACTCTGGACGCTGGAATTCTCGGGTGCCAAGGAACTCCAGTCAC | 71145 | 0.35080184688087385 | RNA PCR Primer, Index 1 (100% over 33bp) |
| CCGAGAAGACGATCAAACTGGAATTCTCGGGTGCCAAGGAACTCCAGTCAC | 64992 | 0.3204626274858634 | RNA PCR Primer, Index 1 (100% over 33bp) |
| CGAGAAGACGATCAAACTTGGAATTCTCGGGTGCCAAGGAACTCCAGTCAC | 54366 | 0.268067934605743 | RNA PCR Primer, Index 1 (100% over 33bp) |
| GGCGGCGACTCTGGACGCGTGCCTGGAATTCTCGGGTGCCAAGGAACTCCA | 50709 | 0.25003599484829897 | RNA PCR Primer, Index 1 (100% over 28bp) |
| GCGGCGACTCTGGACGCGTGCCGTGGAATTCTCGGGTGCCAAGGAACTCCA | 44487 | 0.2193565501748462 | RNA PCR Primer, Index 1 (100% over 28bp) |
| AGCGGCGACTCTGGACGCGTGCTGGAATTCTCGGGTGCCAAGGAACTCCAG | 43163 | 0.21282816946966276 | RNA PCR Primer, Index 1 (100% over 29bp) |
| GCAGCGGCGACTCTGGACGCGTGTGGAATTCTCGGGTGCCAAGGAACTCCA | 42698 | 0.21053534694102957 | RNA PCR Primer, Index 1 (100% over 28bp) |
| TTGGCAGGTGAGTAGAGCCGTTCGTGATGGAATTCTCGGGTGCCAAGGAAC | 42044 | 0.20731060299753265 | RNA PCR Primer, Index 1 (100% over 24bp) |
| GGAATACCAGGTGCTGTAAGCTTTGGAATTCTCGGGTGCCAAGGAACTCCA | 41679 | 0.205510860582584 | RNA PCR Primer, Index 1 (100% over 28bp) |
| GCAGCGGCGACTCTGGACGCGTGCTGGAATTCTCGGGTGCCAAGGAACTCC | 41143 | 0.2028679511732348 | RNA PCR Primer, Index 1 (100% over 27bp) |
| TCGGGCTGGGGTGCGAAGCTGGAATTCTCGGGTGCCAAGGAACTCCAGTCA | 36508 | 0.18001368790395586 | RNA PCR Primer, Index 1 (100% over 32bp) |
| GCAGCGGCGACTCTGGACGCTGGAATTCTCGGGTGCCAAGGAACTCCAGTC | 36152 | 0.17825832269923889 | RNA PCR Primer, Index 1 (100% over 31bp) |
| TTTTGGCAGGTGAGTAGAGCCGTTCGTGACATGGAATTCTCGGGTGCCAAG | 33222 | 0.16381107536828152 | No Hit |
| CCGAGAAGACGATCAAACTTGTTGGAATTCTCGGGTGCCAAGGAACTCCAG | 31715 | 0.15638035805505535 | RNA PCR Primer, Index 1 (100% over 29bp) |
| GAGAAGACGATCAAACTTGATGGAATTCTCGGGTGCCAAGGAACTCCAGTC | 31458 | 0.15511314216288605 | RNA PCR Primer, Index 1 (100% over 31bp) |
| GCATTGGTGGTTCAGTGGTAGAATTCTCTGGAATTCTCGGGTGCCAAGGAA | 30178 | 0.1488017167077238 | RNA PCR Primer, Index 1 (100% over 23bp) |
| CTTTTGGCAGGTGAGTAGAGCCGTTCGTGACAGTGGAATTCTCGGGTGCCA | 29505 | 0.1454832875426268 | No Hit |
| GCGTGTCGGCTGAGGTGGGATCCCGACTGGAATTCTCGGGTGCCAAGGAAC | 28683 | 0.14143016900813976 | RNA PCR Primer, Index 1 (100% over 24bp) |
| CTTTTGGCAGGTGAGTAGAGCCGTTCGTGATGGAATTCTCGGGTGCCAAGG | 26805 | 0.13217012447314389 | Illumina Small RNA Adapter 2 (100% over 21bp) |
| TCTGGACGCGTGCCTGGAATTCTCGGGTGCCAAGGAACTCCAGTCACCATG | 26639 | 0.13135161148442753 | RNA PCR Primer, Index 12 (97% over 37bp) |
| TGGTTGGCAGCGGCGACTCTGGACGCGTGCCTGGAATTCTCGGGTGCCAAG | 24065 | 0.11865972935818721 | No Hit |
| CCGAGAAGACGATCAAACTTGTGGAATTCTCGGGTGCCAAGGAACTCCAGT | 23998 | 0.11832936568201854 | RNA PCR Primer, Index 1 (100% over 30bp) |
| CAGCGGCGACTCTGGACGCGTGCTGGAATTCTCGGGTGCCAAGGAACTCCA | 23899 | 0.11784121636947084 | RNA PCR Primer, Index 1 (100% over 28bp) |
| GCGACTCTGGACGCGTGCCTGGAATTCTCGGGTGCCAAGGAACTCCAGTCA | 22382 | 0.11036119104487621 | RNA PCR Primer, Index 1 (100% over 32bp) |
| TGGGAATACCAGGTGCTGTAAGCTTTGGAATTCTCGGGTGCCAAGGAACTC | 21940 | 0.10818177694239049 | RNA PCR Primer, Index 1 (100% over 26bp) |
| CGAGAAGACGATCAAACTGGAATTCTCGGGTGCCAAGGAACTCCAGTCACC | 20296 | 0.10007553987341647 | RNA PCR Primer, Index 2 (100% over 34bp) |

## Adapter Content

Produced by FastQC (version 0.11.9)
