# Supplementary material for: High Throughput Sequencing of MicroRNA in Rainbow Trout Plasma, Mucus, and Surrounding Water Following Acute Stress
Source: Front Physiol. 2021 Jan 13;11:588313. doi: 10.3389/fphys.2020.588313 (PMC7838646; doi:10.3389/fphys.2020.588313)
Supplement: Supplementary file 2 [file Data_Sheet_1.ZIP › Supplemental Quality Control/FastQC_raw_files/mucus_stressed_2_fastqc_raw.html]

SV18263\_0010\_S22\_R1\_001.fastq FastQC Report 

FastQC Report

Thu 7 May 2020  
SV18263\_0010\_S22\_R1\_001.fastq

## Summary

- Basic Statistics
- Per base sequence quality
- Per tile sequence quality
- Per sequence quality scores
- Per base sequence content
- Per sequence GC content
- Per base N content
- Sequence Length Distribution
- Sequence Duplication Levels
- Overrepresented sequences
- Adapter Content

## Basic Statistics

| Measure | Value |
| --- | --- |
| Filename | SV18263\_0010\_S22\_R1\_001.fastq |
| File type | Conventional base calls |
| Encoding | Sanger / Illumina 1.9 |
| Total Sequences | 14400230 |
| Sequences flagged as poor quality | 0 |
| Sequence length | 51 |
| %GC | 53 |

## Per base sequence quality

## Per tile sequence quality

## Per sequence quality scores

## Per base sequence content

## Per sequence GC content

## Per base N content

## Sequence Length Distribution

## Sequence Duplication Levels

## Overrepresented sequences

| Sequence | Count | Percentage | Possible Source |
| --- | --- | --- | --- |
| CCGAGAAGACGATCAAACTTGATGGAATTCTCGGGTGCCAAGGAACTCCAG | 1981772 | 13.762085744463803 | RNA PCR Primer, Index 1 (100% over 29bp) |
| GAGGTGTAGAATAAGTGGGAGGCCCTGGAATTCTCGGGTGCCAAGGAACTC | 707462 | 4.912852086390287 | RNA PCR Primer, Index 1 (100% over 26bp) |
| AGGTGTAGAATAAGTGGGAGGCCCTGGAATTCTCGGGTGCCAAGGAACTCC | 680682 | 4.726882834510282 | RNA PCR Primer, Index 1 (100% over 27bp) |
| TTGGCAGGTGAGTAGAGCCGTTCGTGATGGAATTCTCGGGTGCCAAGGAAC | 530895 | 3.6867119483508253 | RNA PCR Primer, Index 1 (100% over 24bp) |
| GCCGAGAAGACGATCAAACTTGATGGAATTCTCGGGTGCCAAGGAACTCCA | 384483 | 2.6699781878483884 | RNA PCR Primer, Index 1 (100% over 28bp) |
| TTGGCAGGTGAGTAGAGCCGTTCGTGACATGGAATTCTCGGGTGCCAAGGA | 234605 | 1.6291753673378828 | RNA PCR Primer, Index 1 (100% over 22bp) |
| GCATTGGTGGTTCAGTGGTAGAATTCTCGCCTGGAATTCTCGGGTGCCAAG | 179620 | 1.2473411883004648 | No Hit |
| GCATTGGTGGTTCAGTGGTAGAATTCTCGCTGGAATTCTCGGGTGCCAAGG | 155254 | 1.0781355575570668 | Illumina Small RNA Adapter 2 (100% over 21bp) |
| TGAGAACTGAATTCCATAGATGGTGGAATTCTCGGGTGCCAAGGAACTCCA | 151986 | 1.0554414755875425 | RNA PCR Primer, Index 1 (100% over 28bp) |
| AGCGGCGACTCTGGACGCGTGCCTGGAATTCTCGGGTGCCAAGGAACTCCA | 132729 | 0.9217144448387282 | RNA PCR Primer, Index 1 (100% over 28bp) |
| TTTTGGCAGGTGAGTAGAGCCGTTCGTGATGGAATTCTCGGGTGCCAAGGA | 131955 | 0.9163395306880515 | RNA PCR Primer, Index 1 (100% over 22bp) |
| GCGGCGACTCTGGACGCGTGCCTGGAATTCTCGGGTGCCAAGGAACTCCAG | 122788 | 0.8526808252368191 | RNA PCR Primer, Index 1 (100% over 29bp) |
| CGGCGACTCTGGACGCGTGCCTGGAATTCTCGGGTGCCAAGGAACTCCAGT | 95857 | 0.6656629789940854 | RNA PCR Primer, Index 1 (100% over 30bp) |
| GGCGACTCTGGACGCGTGCCTGGAATTCTCGGGTGCCAAGGAACTCCAGTC | 95217 | 0.6612186055361615 | RNA PCR Primer, Index 1 (100% over 31bp) |
| GATCGGGGGCCTGAGTCCTTGGAATTCTCGGGTGCCAAGGAACTCCAGTCA | 82663 | 0.5740394424255724 | RNA PCR Primer, Index 1 (100% over 32bp) |
| CCGAGAAGACGATCAAACTTGTGGAATTCTCGGGTGCCAAGGAACTCCAGT | 72715 | 0.5049572124889672 | RNA PCR Primer, Index 1 (100% over 30bp) |
| CCGAGAAGACGATCAAACTTTGGAATTCTCGGGTGCCAAGGAACTCCAGTC | 68985 | 0.4790548484295043 | RNA PCR Primer, Index 1 (100% over 31bp) |
| GCAGCGGCGACTCTGGACGCGTGCCTGGAATTCTCGGGTGCCAAGGAACTC | 67204 | 0.4666869904161253 | RNA PCR Primer, Index 1 (100% over 26bp) |
| CTTTTGGCAGGTGAGTAGAGCCGTTCGTGATGGAATTCTCGGGTGCCAAGG | 62078 | 0.43109033675156583 | Illumina Small RNA Adapter 2 (100% over 21bp) |
| CCGAGAAGACGATCAAACTTGACTGGAATTCTCGGGTGCCAAGGAACTCCA | 56504 | 0.39238262166645954 | RNA PCR Primer, Index 1 (100% over 28bp) |
| GTGGTTGGCAGCGGCGACTCTGGACGCGTGCCTGGAATTCTCGGGTGCCAA | 50214 | 0.3487027637753008 | No Hit |
| CGGGGGCCTGAGTCCTTGGAATTCTCGGGTGCCAAGGAACTCCAGTCACCC | 50006 | 0.3472583424014755 | RNA PCR Primer, Index 5 (97% over 35bp) |
| TTTTGGCAGGTGAGTAGAGCCGTTCGTGACATGGAATTCTCGGGTGCCAAG | 49750 | 0.34548059301830597 | No Hit |
| TGTGGTCGGATCTGGAATTCTCGGGTGCCAAGGAACTCCAGTCACCCAACA | 47447 | 0.32948779290330776 | RNA PCR Primer, Index 36 (100% over 39bp) |
| TTGGCAGGTGAGTAGAGCCGTTCGTTGGAATTCTCGGGTGCCAAGGAACTC | 45248 | 0.31421720347522225 | RNA PCR Primer, Index 1 (100% over 26bp) |
| TTTGGCAGGTGAGTAGAGCCGTTCGTGATGGAATTCTCGGGTGCCAAGGAA | 44849 | 0.31144641439754783 | RNA PCR Primer, Index 1 (100% over 23bp) |
| GCGTGTCGGCTGAGGTGGGATCCCGTGGAATTCTCGGGTGCCAAGGAACTC | 42325 | 0.2939189165728603 | RNA PCR Primer, Index 1 (100% over 26bp) |
| AGGTGAGTAGAGCCGTTCGTGACATGGAATTCTCGGGTGCCAAGGAACTCC | 40905 | 0.28405796296309155 | RNA PCR Primer, Index 1 (100% over 27bp) |
| AGGTGAGTAGAGCCGTTCGTGATGGAATTCTCGGGTGCCAAGGAACTCCAG | 40680 | 0.2824954879192902 | RNA PCR Primer, Index 1 (100% over 29bp) |
| GCATTGGTGGTTCAGTGGTAGAATTCTGGAATTCTCGGGTGCCAAGGAACT | 32790 | 0.22770469638332164 | RNA PCR Primer, Index 1 (100% over 25bp) |
| TGATGCGCACCGCATGTTTGTGGAGAACCTGGAATTCTCGGGTGCCAAGGA | 31469 | 0.21853123179282555 | RNA PCR Primer, Index 1 (100% over 22bp) |
| GCATTGGTGGTTCAGTGGTAGAATTCTCGCCTTGGAATTCTCGGGTGCCAA | 30368 | 0.21088552057849075 | No Hit |
| CTTTTGGCAGGTGAGTAGAGCCGTTCGTGACATGGAATTCTCGGGTGCCAA | 30176 | 0.2095522085411136 | No Hit |
| TCGGGGGCCTGAGTCCTTGGAATTCTCGGGTGCCAAGGAACTCCAGTCACC | 29211 | 0.20285092668658766 | RNA PCR Primer, Index 2 (100% over 34bp) |
| GGTTGGCAGCGGCGACTCTGGACGCGTGCCTGGAATTCTCGGGTGCCAAGG | 26943 | 0.1871011782450697 | Illumina Small RNA Adapter 2 (100% over 21bp) |
| TGTGGTCGGATCCCCTCGTGGTGGAATTCTCGGGTGCCAAGGAACTCCAGT | 26244 | 0.1822470891089934 | RNA PCR Primer, Index 1 (100% over 30bp) |
| TTGGCAGGTGAGTAGAGCCGTTCGTGACTGGAATTCTCGGGTGCCAAGGAA | 25999 | 0.1805457273946319 | RNA PCR Primer, Index 1 (100% over 23bp) |
| TGTGGTCGGATCCTGGAATTCTCGGGTGCCAAGGAACTCCAGTCACCCAAC | 25811 | 0.17924019269136673 | RNA PCR Primer, Index 36 (100% over 38bp) |
| GATCGGCTCACGTAAACTGGCTGGAATTCTCGGGTGCCAAGGAACTCCAGT | 25057 | 0.17400416521125012 | RNA PCR Primer, Index 1 (100% over 30bp) |
| TGTGGTCGGATCCCTGGAATTCTCGGGTGCCAAGGAACTCCAGTCACCCAA | 24480 | 0.16999728476559053 | RNA PCR Primer, Index 28 (97% over 37bp) |
| TCTTTTGGCAGGTGAGTAGAGCCGTTCGTGATGGAATTCTCGGGTGCCAAG | 23813 | 0.16536541430241045 | No Hit |
| GCATTGGTGGTTCAGTGGTAGAATTCTCTGGAATTCTCGGGTGCCAAGGAA | 23097 | 0.16039327149635804 | RNA PCR Primer, Index 1 (100% over 23bp) |
| AAATTGATTTTTGGAATAGGGATGGAATTCTCGGGTGCCAAGGAACTCCAG | 22939 | 0.15929606679893307 | RNA PCR Primer, Index 1 (100% over 29bp) |
| TGGGAATACCAGGTGCTGTAAGCTTTGGAATTCTCGGGTGCCAAGGAACTC | 22783 | 0.15821275076856411 | RNA PCR Primer, Index 1 (100% over 26bp) |
| CCGCCGGTGAAATACCACTACTCTTATTGGAATTCTCGGGTGCCAAGGAAC | 21333 | 0.1481434671529552 | RNA PCR Primer, Index 1 (100% over 24bp) |
| ATCGGGGGCCTGAGTCCTTGGAATTCTCGGGTGCCAAGGAACTCCAGTCAC | 20738 | 0.14401158870379155 | RNA PCR Primer, Index 1 (100% over 33bp) |
| TTGGCAGGTGAGTAGAGCCGTTCTGGAATTCTCGGGTGCCAAGGAACTCCA | 20207 | 0.14032414760042028 | RNA PCR Primer, Index 1 (100% over 28bp) |
| CGAGAAGACGATCAAACTTGATGGAATTCTCGGGTGCCAAGGAACTCCAGT | 19890 | 0.13812279387204232 | RNA PCR Primer, Index 1 (100% over 30bp) |
| TTTGGCAGGTGAGTAGAGCCGTTCGTGACATGGAATTCTCGGGTGCCAAGG | 18807 | 0.13060208066121168 | Illumina Small RNA Adapter 2 (100% over 21bp) |
| AATTGATTTTTGGAATAGGGATGGAATTCTCGGGTGCCAAGGAACTCCAGT | 18502 | 0.1284840589351698 | RNA PCR Primer, Index 1 (100% over 30bp) |
| CTCCGGGGATGCGTGCATTTATCAGATCTGGAATTCTCGGGTGCCAAGGAA | 18476 | 0.1283035062634416 | RNA PCR Primer, Index 1 (100% over 23bp) |
| GGAATACCAGGTGCTGTAAGCTTTGGAATTCTCGGGTGCCAAGGAACTCCA | 18413 | 0.12786601325117725 | RNA PCR Primer, Index 1 (100% over 28bp) |
| CCGAGAAGACGATCAAACTTGTTGGAATTCTCGGGTGCCAAGGAACTCCAG | 17927 | 0.12449106715656626 | RNA PCR Primer, Index 1 (100% over 29bp) |
| CAGCGGCGACTCTGGACGCGTGCCTGGAATTCTCGGGTGCCAAGGAACTCC | 17714 | 0.12301192411510094 | RNA PCR Primer, Index 1 (100% over 27bp) |
| CCGAGAAGACGATCAAACTTGACTATTGGAATTCTCGGGTGCCAAGGAACT | 17660 | 0.1226369301045886 | RNA PCR Primer, Index 1 (100% over 25bp) |
| TACCCTGTAGAACCGAATTTGTTGGAATTCTCGGGTGCCAAGGAACTCCAG | 17483 | 0.12140778307013153 | RNA PCR Primer, Index 1 (100% over 29bp) |
| CGTTTTTTCACTTACCCGGTGAGGCGGGGAGTGGAATTCTCGGGTGCCAAG | 17364 | 0.1205814073802988 | No Hit |
| CCGAGAAGACGATCAAACTTGGAATTCTCGGGTGCCAAGGAACTCCAGTCA | 17251 | 0.11979669769163409 | RNA PCR Primer, Index 1 (100% over 32bp) |
| CCGCCGGTGAAATACCACTACTCTTATGGAATTCTCGGGTGCCAAGGAACT | 16684 | 0.11585926058125462 | RNA PCR Primer, Index 1 (100% over 25bp) |
| CGAGAAGACGATCAAACTTGACTGGAATTCTCGGGTGCCAAGGAACTCCAG | 16154 | 0.11217876381141134 | RNA PCR Primer, Index 1 (100% over 29bp) |
| AGCGGCGACTCTGGACGCGTGCCGTGGAATTCTCGGGTGCCAAGGAACTCC | 16124 | 0.11197043380557117 | RNA PCR Primer, Index 1 (100% over 27bp) |
| GCGGCGACTCTGGACGCGTGCCGTGGAATTCTCGGGTGCCAAGGAACTCCA | 16011 | 0.11118572411690647 | RNA PCR Primer, Index 1 (100% over 28bp) |
| ATGCGCACCGCATGTTTGTGGAGAACCTGGAATTCTCGGGTGCCAAGGAAC | 15578 | 0.1081788276992798 | RNA PCR Primer, Index 1 (100% over 24bp) |
| TTGGCAGCGGCGACTCTGGACGCGTGCTGGAATTCTCGGGTGCCAAGGAAC | 15371 | 0.10674135065898253 | RNA PCR Primer, Index 1 (100% over 24bp) |
| GCATTGGTGGTTCAGTGGTAGAATTCTCGTGGAATTCTCGGGTGCCAAGGA | 15210 | 0.10562331296097355 | RNA PCR Primer, Index 1 (100% over 22bp) |
| TGATGCGCACCGCATGTTTGTGGAGAACTGGAATTCTCGGGTGCCAAGGAA | 15194 | 0.10551220362452543 | RNA PCR Primer, Index 1 (100% over 23bp) |
| TGCGCACCGCATGTTTGTGGAGAACTGGAATTCTCGGGTGCCAAGGAACTC | 14793 | 0.10272752587979497 | RNA PCR Primer, Index 1 (100% over 26bp) |
| CGTGGAGCTTCGGTTGGCCCGGGATAGCCTGCCTGGAATTCTCGGGTGCCA | 14496 | 0.10066505882197715 | No Hit |

## Adapter Content

Produced by FastQC (version 0.11.9)
