# Supplementary material for: High Throughput Sequencing of MicroRNA in Rainbow Trout Plasma, Mucus, and Surrounding Water Following Acute Stress
Source: Front Physiol. 2021 Jan 13;11:588313. doi: 10.3389/fphys.2020.588313 (PMC7838646; doi:10.3389/fphys.2020.588313)
Supplement: Supplementary file 2 [file Data_Sheet_1.ZIP › Supplemental Quality Control/FastQC_raw_files/mucus_stressed_3_fastqc_raw.html]

SV18263\_0012\_S24\_R1\_001.fastq FastQC Report 

FastQC Report

Thu 7 May 2020  
SV18263\_0012\_S24\_R1\_001.fastq

## Summary

- Basic Statistics
- Per base sequence quality
- Per tile sequence quality
- Per sequence quality scores
- Per base sequence content
- Per sequence GC content
- Per base N content
- Sequence Length Distribution
- Sequence Duplication Levels
- Overrepresented sequences
- Adapter Content

## Basic Statistics

| Measure | Value |
| --- | --- |
| Filename | SV18263\_0012\_S24\_R1\_001.fastq |
| File type | Conventional base calls |
| Encoding | Sanger / Illumina 1.9 |
| Total Sequences | 20698775 |
| Sequences flagged as poor quality | 0 |
| Sequence length | 51 |
| %GC | 53 |

## Per base sequence quality

## Per tile sequence quality

## Per sequence quality scores

## Per base sequence content

## Per sequence GC content

## Per base N content

## Sequence Length Distribution

## Sequence Duplication Levels

## Overrepresented sequences

| Sequence | Count | Percentage | Possible Source |
| --- | --- | --- | --- |
| GCATTGGTGGTTCAGTGGTAGAATTCTCGCCTGGAATTCTCGGGTGCCAAG | 1813033 | 8.759131880992957 | No Hit |
| GCATTGGTGGTTCAGTGGTAGAATTCTCGCTGGAATTCTCGGGTGCCAAGG | 1531685 | 7.3998823601879815 | Illumina Small RNA Adapter 2 (100% over 21bp) |
| CCGAGAAGACGATCAAACTTGATGGAATTCTCGGGTGCCAAGGAACTCCAG | 1094582 | 5.288148694789909 | RNA PCR Primer, Index 1 (100% over 29bp) |
| CTTTTGGCAGGTGAGTAGAGCCGTTCGTGACATGGAATTCTCGGGTGCCAA | 924740 | 4.467607382562495 | No Hit |
| CGAGAAGACGATCAAACTTGATGGAATTCTCGGGTGCCAAGGAACTCCAGT | 797754 | 3.8541121394865154 | RNA PCR Primer, Index 1 (100% over 30bp) |
| GCATTGGTGGTTCAGTGGTAGAATTCTCGCCTTGGAATTCTCGGGTGCCAA | 523900 | 2.5310676598011237 | No Hit |
| TGAGAACTGAATTCCATAGATGGTGGAATTCTCGGGTGCCAAGGAACTCCA | 482732 | 2.3321766626285854 | RNA PCR Primer, Index 1 (100% over 28bp) |
| AGCGGCGACTCTGGACGCGTGCCTGGAATTCTCGGGTGCCAAGGAACTCCA | 366924 | 1.772684615393906 | RNA PCR Primer, Index 1 (100% over 28bp) |
| CGAGAAGACGATCAAACTTGACTATTGGAATTCTCGGGTGCCAAGGAACTC | 322934 | 1.5601599611571215 | RNA PCR Primer, Index 1 (100% over 26bp) |
| CGAGAAGACGATCAAACTTGACTGGAATTCTCGGGTGCCAAGGAACTCCAG | 313200 | 1.513133023572651 | RNA PCR Primer, Index 1 (100% over 29bp) |
| CCGAGAAGACGATCAAACTTGACTATTGGAATTCTCGGGTGCCAAGGAACT | 283803 | 1.3711101260823406 | RNA PCR Primer, Index 1 (100% over 25bp) |
| TTGGCAGGTGAGTAGAGCCGTTCGTGACATGGAATTCTCGGGTGCCAAGGA | 228014 | 1.1015820984575173 | RNA PCR Primer, Index 1 (100% over 22bp) |
| GCGGCGACTCTGGACGCGTGCCTGGAATTCTCGGGTGCCAAGGAACTCCAG | 217754 | 1.0520139476853099 | RNA PCR Primer, Index 1 (100% over 29bp) |
| CCGAGAAGACGATCAAACTTGACTGGAATTCTCGGGTGCCAAGGAACTCCA | 198053 | 0.9568344020358693 | RNA PCR Primer, Index 1 (100% over 28bp) |
| GCATTGGTGGTTCAGTGGTAGAATTCTGGAATTCTCGGGTGCCAAGGAACT | 193010 | 0.9324706413785357 | RNA PCR Primer, Index 1 (100% over 25bp) |
| GGAATACCAGGTGCTGTAAGCTTTGGAATTCTCGGGTGCCAAGGAACTCCA | 187792 | 0.9072614200598828 | RNA PCR Primer, Index 1 (100% over 28bp) |
| GCATTGGTGGTTCAGTGGTAGAATTCTCTGGAATTCTCGGGTGCCAAGGAA | 186896 | 0.9029326614739277 | RNA PCR Primer, Index 1 (100% over 23bp) |
| GCATTGGTGGTTCAGTGGTAGAATTCTCGTGGAATTCTCGGGTGCCAAGGA | 182838 | 0.8833276365388772 | RNA PCR Primer, Index 1 (100% over 22bp) |
| GCAGCGGCGACTCTGGACGCGTGCCTGGAATTCTCGGGTGCCAAGGAACTC | 173888 | 0.8400883627171174 | RNA PCR Primer, Index 1 (100% over 26bp) |
| GCCGAGAAGACGATCAAACTTGATGGAATTCTCGGGTGCCAAGGAACTCCA | 172901 | 0.8353199645872764 | RNA PCR Primer, Index 1 (100% over 28bp) |
| GAGAAGACGATCAAACTTGATGGAATTCTCGGGTGCCAAGGAACTCCAGTC | 128553 | 0.6210657393976213 | RNA PCR Primer, Index 1 (100% over 31bp) |
| GTGGTTGGCAGCGGCGACTCTGGACGCGTGCCTGGAATTCTCGGGTGCCAA | 124329 | 0.6006587346352623 | No Hit |
| TACCCTGTAGAACCGAATTTGTTGGAATTCTCGGGTGCCAAGGAACTCCAG | 122048 | 0.5896387588154371 | RNA PCR Primer, Index 1 (100% over 29bp) |
| CGGCGACTCTGGACGCGTGCCTGGAATTCTCGGGTGCCAAGGAACTCCAGT | 119833 | 0.578937642445024 | RNA PCR Primer, Index 1 (100% over 30bp) |
| GGCGACTCTGGACGCGTGCCTGGAATTCTCGGGTGCCAAGGAACTCCAGTC | 98706 | 0.47686880020677547 | RNA PCR Primer, Index 1 (100% over 31bp) |
| CGAGAAGACGATCAAACTTGGAATTCTCGGGTGCCAAGGAACTCCAGTCAC | 90528 | 0.43735921570237857 | RNA PCR Primer, Index 1 (100% over 33bp) |
| CCGAGAAGACGATCAAACTTGGAATTCTCGGGTGCCAAGGAACTCCAGTCA | 87831 | 0.42432945911050296 | RNA PCR Primer, Index 1 (100% over 32bp) |
| GGTGAGTAGAGCCGTTCGTGACATGGAATTCTCGGGTGCCAAGGAACTCCA | 84307 | 0.4073042969934211 | RNA PCR Primer, Index 1 (100% over 28bp) |
| CTGGCGGAGCGCCGAGAAGACGATCAAACTGGAATTCTCGGGTGCCAAGGA | 82770 | 0.39987873678514796 | RNA PCR Primer, Index 1 (100% over 22bp) |
| TTTTGGCAGGTGAGTAGAGCCGTTCGTGACATGGAATTCTCGGGTGCCAAG | 76713 | 0.3706161354959412 | No Hit |
| CAGGTGAGTAGAGCCGTTCGTGACATGGAATTCTCGGGTGCCAAGGAACTC | 76698 | 0.3705436674392567 | RNA PCR Primer, Index 1 (100% over 26bp) |
| CTTTTGGCAGGTGAGTAGAGCCGTTCGTGACAGTGGAATTCTCGGGTGCCA | 75752 | 0.3659733486643533 | No Hit |
| TGGCGGAGCGCCGAGAAGACGATCAAACTGGAATTCTCGGGTGCCAAGGAA | 63255 | 0.3055977950385953 | RNA PCR Primer, Index 1 (100% over 23bp) |
| AGCGGCGACTCTGGACGCTGGAATTCTCGGGTGCCAAGGAACTCCAGTCAC | 63107 | 0.3048827768793081 | RNA PCR Primer, Index 1 (100% over 33bp) |
| CCGAGAAGACGATCAAACTGGAATTCTCGGGTGCCAAGGAACTCCAGTCAC | 62391 | 0.3014236349735673 | RNA PCR Primer, Index 1 (100% over 33bp) |
| GAGAAGACGATCAAACTTGACTGGAATTCTCGGGTGCCAAGGAACTCCAGT | 57274 | 0.2767023652365901 | RNA PCR Primer, Index 1 (100% over 30bp) |
| CTGGCGGAGCGCCGAGAAGACGATCAAACTTGATGGAATTCTCGGGTGCCA | 56417 | 0.27256202359801485 | No Hit |
| TTGGCAGGTGAGTAGAGCCGTTCGTGATGGAATTCTCGGGTGCCAAGGAAC | 55810 | 0.26962948290418154 | RNA PCR Primer, Index 1 (100% over 24bp) |
| CTTTTGGCAGGTGAGTAGAGCCGTTCGTGATGGAATTCTCGGGTGCCAAGG | 52914 | 0.25563831676029136 | Illumina Small RNA Adapter 2 (100% over 21bp) |
| AGACGATCAAACTTGATGGAATTCTCGGGTGCCAAGGAACTCCAGTCACCT | 52686 | 0.25453680229868675 | RNA PCR Primer, Index 1 (97% over 35bp) |
| CTGGCGGAGCGCCGAGAAGACGATCAAACTTGGAATTCTCGGGTGCCAAGG | 50633 | 0.24461834094046628 | Illumina Small RNA Adapter 2 (100% over 21bp) |
| AGGTGAGTAGAGCCGTTCGTGACATGGAATTCTCGGGTGCCAAGGAACTCC | 50045 | 0.24177759311843333 | RNA PCR Primer, Index 1 (100% over 27bp) |
| GAAGACGATCAAACTTGATGGAATTCTCGGGTGCCAAGGAACTCCAGTCAC | 46327 | 0.22381517746823182 | RNA PCR Primer, Index 1 (100% over 33bp) |
| CCTGGCGGAGCGCCGAGAAGACGATCAAACTGGAATTCTCGGGTGCCAAGG | 45146 | 0.21810952580527107 | Illumina Small RNA Adapter 2 (100% over 21bp) |
| GCGTGTCGGCTGAGGTGGGATCCCGACTGGAATTCTCGGGTGCCAAGGAAC | 44756 | 0.21622535633147372 | RNA PCR Primer, Index 1 (100% over 24bp) |
| CAGCGGCGACTCTGGACGCGTGCCTGGAATTCTCGGGTGCCAAGGAACTCC | 42922 | 0.20736492860084715 | RNA PCR Primer, Index 1 (100% over 27bp) |
| AGAAGACGATCAAACTTGATGGAATTCTCGGGTGCCAAGGAACTCCAGTCA | 42605 | 0.20583343700291443 | RNA PCR Primer, Index 1 (100% over 32bp) |
| GGTTGGCAGCGGCGACTCTGGACGCGTGCCTGGAATTCTCGGGTGCCAAGG | 42258 | 0.20415700929161265 | Illumina Small RNA Adapter 2 (100% over 21bp) |
| TGGGAATACCAGGTGCTGTAAGCTTTGGAATTCTCGGGTGCCAAGGAACTC | 40882 | 0.19750927289175327 | RNA PCR Primer, Index 1 (100% over 26bp) |
| AAGACGATCAAACTTGATGGAATTCTCGGGTGCCAAGGAACTCCAGTCACC | 40304 | 0.19471683710750998 | RNA PCR Primer, Index 2 (100% over 34bp) |
| TCCCATATGGTCTAGCGGTTAGGATTCCTGGAATTCTCGGGTGCCAAGGAA | 39549 | 0.19106927825438946 | RNA PCR Primer, Index 1 (100% over 23bp) |
| TCCCTGGTGGTCTAGTGGTTAGGATTCGGTGGAATTCTCGGGTGCCAAGGA | 37320 | 0.18030052503107066 | RNA PCR Primer, Index 1 (100% over 22bp) |
| CGAGAAGACGATCAAACTGGAATTCTCGGGTGCCAAGGAACTCCAGTCACC | 34112 | 0.16480202330814264 | RNA PCR Primer, Index 2 (100% over 34bp) |
| AGACGATCAAACTTGACTGGAATTCTCGGGTGCCAAGGAACTCCAGTCACC | 33334 | 0.16104334676810583 | RNA PCR Primer, Index 2 (100% over 34bp) |
| GGAGCGCCGAGAAGACGATCAAACTTGATGGAATTCTCGGGTGCCAAGGAA | 32928 | 0.159081878033845 | RNA PCR Primer, Index 1 (100% over 23bp) |
| GCATTGGTGGTTCAGTGGTAGAATTCTTGGAATTCTCGGGTGCCAAGGAAC | 32800 | 0.15846348395013715 | RNA PCR Primer, Index 1 (100% over 24bp) |
| TAACACTGTCTGGTAACGATGTGGAATTCTCGGGTGCCAAGGAACTCCAGT | 31497 | 0.15216842542614237 | RNA PCR Primer, Index 1 (100% over 30bp) |
| TCTCGCAAGGGGCTGCTTATGGGGGTTCATTGTGGAATTCTCGGGTGCCAA | 30506 | 0.14738070248118548 | No Hit |
| TAGCTTATCAGACTGGTGTTGGCTGGAATTCTCGGGTGCCAAGGAACTCCA | 30458 | 0.14714880469979505 | RNA PCR Primer, Index 1 (100% over 28bp) |
| GCAGCGGCGACTCTGGACGCTGGAATTCTCGGGTGCCAAGGAACTCCAGTC | 30123 | 0.14553035143384088 | RNA PCR Primer, Index 1 (100% over 31bp) |
| TGGCGGAGCGCCGAGAAGACGATCAAACTTGATGGAATTCTCGGGTGCCAA | 29793 | 0.14393605418678157 | No Hit |
| AGCGCCGAGAAGACGATCAAACTTGATGGAATTCTCGGGTGCCAAGGAACT | 29663 | 0.1433079976955158 | RNA PCR Primer, Index 1 (100% over 25bp) |
| GTGAAATGTTTAGGACCACTTGTGGAATTCTCGGGTGCCAAGGAACTCCAG | 29549 | 0.14275724046471347 | RNA PCR Primer, Index 1 (100% over 29bp) |
| CCTGGCGGAGCGCCGAGAAGACGATCAAACTTGATGGAATTCTCGGGTGCC | 29108 | 0.14062667959818878 | No Hit |
| TCCCATATGGTCTAGCGGTTAGGATTCCTTGGAATTCTCGGGTGCCAAGGA | 28889 | 0.13956864597059487 | RNA PCR Primer, Index 1 (100% over 22bp) |
| TCTTTTGGCAGGTGAGTAGAGCCGTTCGTGACATGGAATTCTCGGGTGCCA | 28875 | 0.13950100911768934 | No Hit |
| GAGAAGACGATCAAACTTGACTATTGGAATTCTCGGGTGCCAAGGAACTCC | 27190 | 0.13136043075012893 | RNA PCR Primer, Index 1 (100% over 27bp) |
| TGGCGGAGCGCCGAGAAGACGATCAAACTTGGAATTCTCGGGTGCCAAGGA | 27156 | 0.13119616982164403 | RNA PCR Primer, Index 1 (100% over 22bp) |
| TAGCTTATCAGACTGGTGTTGGTGGAATTCTCGGGTGCCAAGGAACTCCAG | 26736 | 0.12916706423447766 | RNA PCR Primer, Index 1 (100% over 29bp) |
| AACCCGTAGATCCGAACTTGTTGGAATTCTCGGGTGCCAAGGAACTCCAGT | 26399 | 0.12753894856096556 | RNA PCR Primer, Index 1 (100% over 30bp) |
| TGAGAACTGAATTCCATAGATGTGGAATTCTCGGGTGCCAAGGAACTCCAG | 25070 | 0.12111827873871764 | RNA PCR Primer, Index 1 (100% over 29bp) |
| AGACGATCAAACTTGACTATTGGAATTCTCGGGTGCCAAGGAACTCCAGTC | 24205 | 0.11693928746991065 | RNA PCR Primer, Index 1 (100% over 31bp) |
| TCTCGCAAGGGGCTGCTTATGGGTGGAATTCTCGGGTGCCAAGGAACTCCA | 24142 | 0.1166349216318357 | RNA PCR Primer, Index 1 (100% over 28bp) |
| GAGGTGTAGAATAAGTGGGAGGCCCTGGAATTCTCGGGTGCCAAGGAACTC | 22031 | 0.10643625045443511 | RNA PCR Primer, Index 1 (100% over 26bp) |
| AGCGGCGACTCTGGACGCGTGCTGGAATTCTCGGGTGCCAAGGAACTCCAG | 21756 | 0.10510766941521901 | RNA PCR Primer, Index 1 (100% over 29bp) |
| CCTGGCGGAGCGCCGAGAAGACGATCAAACTTGGAATTCTCGGGTGCCAAG | 21436 | 0.10356168420594938 | No Hit |
| GTTGTCGTGGCCGAGTGGTTAAGGTGGAATTCTCGGGTGCCAAGGAACTCC | 21145 | 0.1021558039062698 | RNA PCR Primer, Index 1 (100% over 27bp) |
| TCTCGCAAGGGGCTGCTTATGGGGGTGGAATTCTCGGGTGCCAAGGAACTC | 21056 | 0.1017258267699417 | RNA PCR Primer, Index 1 (100% over 26bp) |
| TGAGGTAGTAGATTGAATAGTTTGGAATTCTCGGGTGCCAAGGAACTCCAG | 20946 | 0.10119439435425526 | RNA PCR Primer, Index 1 (100% over 29bp) |

## Adapter Content

Produced by FastQC (version 0.11.9)
