# Supplementary material for: High Throughput Sequencing of MicroRNA in Rainbow Trout Plasma, Mucus, and Surrounding Water Following Acute Stress
Source: Front Physiol. 2021 Jan 13;11:588313. doi: 10.3389/fphys.2020.588313 (PMC7838646; doi:10.3389/fphys.2020.588313)
Supplement: Supplementary file 2 [file Data_Sheet_1.ZIP › Supplemental Quality Control/FastQC_raw_files/plasma_control_1_fastqc_raw.html]

SV18263\_0019\_S9\_R1\_001.fastq FastQC Report 

FastQC Report

Thu 7 May 2020  
SV18263\_0019\_S9\_R1\_001.fastq

## Summary

- Basic Statistics
- Per base sequence quality
- Per tile sequence quality
- Per sequence quality scores
- Per base sequence content
- Per sequence GC content
- Per base N content
- Sequence Length Distribution
- Sequence Duplication Levels
- Overrepresented sequences
- Adapter Content

## Basic Statistics

| Measure | Value |
| --- | --- |
| Filename | SV18263\_0019\_S9\_R1\_001.fastq |
| File type | Conventional base calls |
| Encoding | Sanger / Illumina 1.9 |
| Total Sequences | 25446441 |
| Sequences flagged as poor quality | 0 |
| Sequence length | 51 |
| %GC | 53 |

## Per base sequence quality

## Per tile sequence quality

## Per sequence quality scores

## Per base sequence content

## Per sequence GC content

## Per base N content

## Sequence Length Distribution

## Sequence Duplication Levels

## Overrepresented sequences

| Sequence | Count | Percentage | Possible Source |
| --- | --- | --- | --- |
| GCATTGGTGGTTCAGTGGTAGAATTCTCGCCTTGGAATTCTCGGGTGCCAA | 2832207 | 11.130071195417859 | No Hit |
| GCATTGGTGGTTCAGTGGTAGAATTCTCGCCTGGAATTCTCGGGTGCCAAG | 1550383 | 6.09273021716475 | No Hit |
| TGAGAACTGAATTCCATAGATGGTGGAATTCTCGGGTGCCAAGGAACTCCA | 858369 | 3.373237931386947 | RNA PCR Primer, Index 1 (100% over 28bp) |
| TACCCTGTAGAACCGAATTTGTTGGAATTCTCGGGTGCCAAGGAACTCCAG | 469997 | 1.847004852269911 | RNA PCR Primer, Index 1 (100% over 29bp) |
| GGTTGGCAGCGGCGACTCTGGACGCTGGAATTCTCGGGTGCCAAGGAACTC | 329588 | 1.295222384929979 | RNA PCR Primer, Index 1 (100% over 26bp) |
| AACCCGTAGATCCGAACTTGTGTGGAATTCTCGGGTGCCAAGGAACTCCAG | 302995 | 1.1907166114113954 | RNA PCR Primer, Index 1 (100% over 29bp) |
| GAGCCGCGGCTGGGGGAGCATGGAATTCTCGGGTGCCAAGGAACTCCAGTC | 260628 | 1.0242218155379763 | RNA PCR Primer, Index 1 (100% over 31bp) |
| TAACGGAACCCATAATGCAGCTGTGGAATTCTCGGGTGCCAAGGAACTCCA | 248276 | 0.9756806462640494 | RNA PCR Primer, Index 1 (100% over 28bp) |
| TCCCTGGTGGTCTAGTGGTTAGGATTCGGCGCTTGGAATTCTCGGGTGCCA | 241927 | 0.9507302023100205 | No Hit |
| AACCCGTAGATCCGAACTTGTTGGAATTCTCGGGTGCCAAGGAACTCCAGT | 239822 | 0.9424579256486201 | RNA PCR Primer, Index 1 (100% over 30bp) |
| GCATTGGTGGTTCAGTGGTAGAATTCTCGCCTGTGGAATTCTCGGGTGCCA | 226446 | 0.8898926179892898 | No Hit |
| GCATTGGTGGTTCAGTGGTAGAATTCTCGCTGGAATTCTCGGGTGCCAAGG | 214932 | 0.8446446400893547 | Illumina Small RNA Adapter 2 (100% over 21bp) |
| CGAGCCGCGGCTGGGGGAGCATGGAATTCTCGGGTGCCAAGGAACTCCAGT | 207461 | 0.8152849351310072 | RNA PCR Primer, Index 1 (100% over 30bp) |
| GCATTGTGGTTCAGTGGTAGAATTCTCGCCTTGGAATTCTCGGGTGCCAAG | 176306 | 0.6928513107196406 | No Hit |
| TGAGGTAGTAGGTTGTATAGTTTGGAATTCTCGGGTGCCAAGGAACTCCAG | 161688 | 0.6354051633389518 | RNA PCR Primer, Index 1 (100% over 29bp) |
| TCCCTGGTCTAGTGGTTAGGATTCGGCGCTTGGAATTCTCGGGTGCCAAGG | 147347 | 0.5790475768300958 | Illumina Small RNA Adapter 2 (100% over 21bp) |
| GAGCCGCGGCTGGGGGAGCAGTTTGGAATTCTCGGGTGCCAAGGAACTCCA | 142129 | 0.5585417622841639 | RNA PCR Primer, Index 1 (100% over 28bp) |
| TTCAAGTAATCCAGGATAGGCTTGGAATTCTCGGGTGCCAAGGAACTCCAG | 141419 | 0.5557515882083471 | RNA PCR Primer, Index 1 (100% over 29bp) |
| AACCCGTAGATCCGAACTTGTGATGGAATTCTCGGGTGCCAAGGAACTCCA | 110320 | 0.43353803386493217 | RNA PCR Primer, Index 1 (100% over 28bp) |
| GTTTCCGTAGTGTAGTGGTTATCACGTTCGCCTTGGAATTCTCGGGTGCCA | 104825 | 0.41194365844716746 | No Hit |
| CGAGCCGCGGCTGGGGGAGCAGTTTGGAATTCTCGGGTGCCAAGGAACTCC | 102889 | 0.40433552181226445 | RNA PCR Primer, Index 1 (100% over 27bp) |
| TCGCCACTGCTGGAAGTTCGTTGGAATTCTCGGGTGCCAAGGAACTCCAGT | 100895 | 0.39649945546412557 | RNA PCR Primer, Index 1 (100% over 30bp) |
| TGAGAACTGAATTCCATAGATGGTTGGAATTCTCGGGTGCCAAGGAACTCC | 98130 | 0.38563349585900836 | RNA PCR Primer, Index 1 (100% over 27bp) |
| TAGCTTATCAGACTGGTGTTGGTGGAATTCTCGGGTGCCAAGGAACTCCAG | 97744 | 0.3841165843191981 | RNA PCR Primer, Index 1 (100% over 29bp) |
| GCATTGTGGTTCAGTGGTAGAATTCTCGCCTGGAATTCTCGGGTGCCAAGG | 86364 | 0.3393952026532905 | Illumina Small RNA Adapter 2 (100% over 21bp) |
| TCGTACCGTGAGTAATAATGCATGGAATTCTCGGGTGCCAAGGAACTCCAG | 86263 | 0.338998290566449 | RNA PCR Primer, Index 1 (100% over 29bp) |
| TGAGGTAGTAGATTGAATAGTTTGGAATTCTCGGGTGCCAAGGAACTCCAG | 81224 | 0.31919591427343413 | RNA PCR Primer, Index 1 (100% over 29bp) |
| TGAGGTAGTAGGTTGTATAGTTGGAATTCTCGGGTGCCAAGGAACTCCAGT | 78999 | 0.3104520588949944 | RNA PCR Primer, Index 1 (100% over 30bp) |
| GCATTGGTGGTTCAGTGGTAGAATTCTCTGGAATTCTCGGGTGCCAAGGAA | 77983 | 0.3064593590907271 | RNA PCR Primer, Index 1 (100% over 23bp) |
| CGAGCCGCGGCTGGGGGAGCAGTGGAATTCTCGGGTGCCAAGGAACTCCAG | 76195 | 0.2994328362068393 | RNA PCR Primer, Index 1 (100% over 29bp) |
| TAGCTTATCAGACTGGTGTTGGCTGGAATTCTCGGGTGCCAAGGAACTCCA | 73751 | 0.28982834966980253 | RNA PCR Primer, Index 1 (100% over 28bp) |
| GAGCCGCGGCTGGGGGAGCAGTGGAATTCTCGGGTGCCAAGGAACTCCAGT | 71205 | 0.27982302122328223 | RNA PCR Primer, Index 1 (100% over 30bp) |
| CCGTGTGAAAGTAGGTAATCGTCAGGCTTGGAATTCTCGGGTGCCAAGGAA | 64151 | 0.25210205230664673 | RNA PCR Primer, Index 1 (100% over 23bp) |
| GTAGGTAATCGTCAGGCTTGGAATTCTCGGGTGCCAAGGAACTCCAGTCAC | 63969 | 0.2513868245858036 | RNA PCR Primer, Index 1 (100% over 33bp) |
| GTTTCCGTAGTGTAGTGGTTATCACGTTCGCCTGGAATTCTCGGGTGCCAA | 62739 | 0.24655314273614926 | No Hit |
| AAGTAGGTAATCGTCAGGCTTGGAATTCTCGGGTGCCAAGGAACTCCAGTC | 59553 | 0.2340327277987519 | RNA PCR Primer, Index 1 (100% over 31bp) |
| AAAGTAGGTAATCGTCAGGCTTGGAATTCTCGGGTGCCAAGGAACTCCAGT | 59461 | 0.23367118411568832 | RNA PCR Primer, Index 1 (100% over 30bp) |
| TAACGGAACCCATAAAGCAGCTGTGGAATTCTCGGGTGCCAAGGAACTCCA | 57270 | 0.22506094270707638 | RNA PCR Primer, Index 1 (100% over 28bp) |
| TGAAAGTAGGTAATCGTCAGGCTTGGAATTCTCGGGTGCCAAGGAACTCCA | 56718 | 0.22289168060869494 | RNA PCR Primer, Index 1 (100% over 28bp) |
| GGTTGGCAGCGGCGACTCTGGACGTGGAATTCTCGGGTGCCAAGGAACTCC | 56473 | 0.22192887406140607 | RNA PCR Primer, Index 1 (100% over 27bp) |
| AGTAGGTAATCGTCAGGCTTGGAATTCTCGGGTGCCAAGGAACTCCAGTCA | 53984 | 0.21214754550547954 | RNA PCR Primer, Index 1 (100% over 32bp) |
| GAAAGTAGGTAATCGTCAGGCTTGGAATTCTCGGGTGCCAAGGAACTCCAG | 53273 | 0.2093534416070208 | RNA PCR Primer, Index 1 (100% over 29bp) |
| CCCGTGTGAAAGTAGGTAATCGTCAGGCTTGGAATTCTCGGGTGCCAAGGA | 53156 | 0.20889365235790733 | RNA PCR Primer, Index 1 (100% over 22bp) |
| GTGAAAGTAGGTAATCGTCAGGCTTGGAATTCTCGGGTGCCAAGGAACTCC | 51285 | 0.20154095419473395 | RNA PCR Primer, Index 1 (100% over 27bp) |
| AACCCGTAGATCCGAACTTGTGTTGGAATTCTCGGGTGCCAAGGAACTCCA | 49993 | 0.19646362334127587 | RNA PCR Primer, Index 1 (100% over 28bp) |
| TGAGAACTGAATTCCATAGATGTGGAATTCTCGGGTGCCAAGGAACTCCAG | 46214 | 0.1816128235771753 | RNA PCR Primer, Index 1 (100% over 29bp) |
| GTGTGAAAGTAGGTAATCGTCAGGCTTGGAATTCTCGGGTGCCAAGGAACT | 40771 | 0.16022279893679434 | RNA PCR Primer, Index 1 (100% over 25bp) |
| TGTGAAAGTAGGTAATCGTCAGGCTTGGAATTCTCGGGTGCCAAGGAACTC | 40158 | 0.15781381765725117 | RNA PCR Primer, Index 1 (100% over 26bp) |
| GTGCGAAGCGGGGCTGGGCTTGGAATTCTCGGGTGCCAAGGAACTCCAGTC | 39946 | 0.15698069525714814 | RNA PCR Primer, Index 1 (100% over 31bp) |
| TGCGAGTTCGAGTCTCGCCGTCGGCACCATGGAATTCTCGGGTGCCAAGGA | 34641 | 0.13613298614136257 | RNA PCR Primer, Index 1 (100% over 22bp) |
| AACATTCAACGCTGTCGGTGAGTGGAATTCTCGGGTGCCAAGGAACTCCAG | 34509 | 0.13561424955261916 | RNA PCR Primer, Index 1 (100% over 29bp) |
| GAGCCGCGGCTGGGGGAGCTGGAATTCTCGGGTGCCAAGGAACTCCAGTCA | 34030 | 0.1337318645071034 | RNA PCR Primer, Index 1 (100% over 32bp) |
| AACCCGTAGATCCGATCTTGTTGGAATTCTCGGGTGCCAAGGAACTCCAGT | 34020 | 0.13369256628068343 | RNA PCR Primer, Index 1 (100% over 30bp) |
| TAACGGAACCCATAATGCAGCTTGGAATTCTCGGGTGCCAAGGAACTCCAG | 33110 | 0.1301164276764676 | RNA PCR Primer, Index 1 (100% over 29bp) |
| CGTGTGAAAGTAGGTAATCGTCAGGCTTGGAATTCTCGGGTGCCAAGGAAC | 30985 | 0.12176555456222739 | RNA PCR Primer, Index 1 (100% over 24bp) |
| AGCGCCGAGAAGACGATCAAACTGGAATTCTCGGGTGCCAAGGAACTCCAG | 30461 | 0.11970632749782179 | RNA PCR Primer, Index 1 (100% over 29bp) |
| AAGCTGCCAGCTGAAGAACTGTTGGAATTCTCGGGTGCCAAGGAACTCCAG | 29990 | 0.11785538103344197 | RNA PCR Primer, Index 1 (100% over 29bp) |
| GTTCGATTCCGGCCCTGGGCACCATGGAATTCTCGGGTGCCAAGGAACTCC | 29386 | 0.11548176815767675 | RNA PCR Primer, Index 1 (100% over 27bp) |
| TACCCTGTAGATCCGGATTTGTTGGAATTCTCGGGTGCCAAGGAACTCCAG | 28603 | 0.11240471702899436 | RNA PCR Primer, Index 1 (100% over 29bp) |
| TGAGGTAGTAGATTGAATAGTTGGAATTCTCGGGTGCCAAGGAACTCCAGT | 27920 | 0.10972064816451149 | RNA PCR Primer, Index 1 (100% over 30bp) |
| TCCCTGTGGTCTAGTGGTTAGGATTCGGCGCTTGGAATTCTCGGGTGCCAA | 27705 | 0.10887573629648248 | No Hit |
| AAAGTTAGGGGATGAGCTGTGGAATTCTCGGGTGCCAAGGAACTCCAGTCA | 27228 | 0.10700121089625067 | RNA PCR Primer, Index 1 (100% over 32bp) |
| TATTGCACTTGTCCCGGCCTGTTGGAATTCTCGGGTGCCAAGGAACTCCAG | 27070 | 0.10638029891881541 | RNA PCR Primer, Index 1 (100% over 29bp) |
| CGAGTCTCGCCGTCGGCACCATGGAATTCTCGGGTGCCAAGGAACTCCAGT | 27054 | 0.10631742175654348 | RNA PCR Primer, Index 1 (100% over 30bp) |
| TGGAATTCTCGGGTGCCAAGGAACTCCAGTCACGATCAGATCTCGTATGCC | 27018 | 0.10617594814143164 | RNA PCR Primer, Index 9 (100% over 51bp) |
| ACGTTGAAAAGTTAGGGGATGAGCTGTGGAATTCTCGGGTGCCAAGGAACT | 26979 | 0.10602268505839382 | RNA PCR Primer, Index 1 (100% over 25bp) |
| TGAGATGAAGCACTGTAGCTTGGAATTCTCGGGTGCCAAGGAACTCCAGTC | 26124 | 0.10266268669948776 | RNA PCR Primer, Index 1 (100% over 31bp) |
| CGAGTTCGAGTCTCGCCGTCGGCACCATGGAATTCTCGGGTGCCAAGGAAC | 26035 | 0.10231293248435017 | RNA PCR Primer, Index 1 (100% over 24bp) |
| TGGAATGTAAGGAAGTGTGTGGTGGAATTCTCGGGTGCCAAGGAACTCCAG | 25846 | 0.10157019600501305 | RNA PCR Primer, Index 1 (100% over 29bp) |
| AAGTTAGGGGATGAGCTGTGGAATTCTCGGGTGCCAAGGAACTCCAGTCAC | 25572 | 0.1004934246011063 | RNA PCR Primer, Index 1 (100% over 33bp) |
| ACCCTGTAGAACCGAATTTGTTGGAATTCTCGGGTGCCAAGGAACTCCAGT | 25562 | 0.10045412637468634 | RNA PCR Primer, Index 1 (100% over 30bp) |

## Adapter Content

Produced by FastQC (version 0.11.9)
