# Supplementary material for: High Throughput Sequencing of MicroRNA in Rainbow Trout Plasma, Mucus, and Surrounding Water Following Acute Stress
Source: Front Physiol. 2021 Jan 13;11:588313. doi: 10.3389/fphys.2020.588313 (PMC7838646; doi:10.3389/fphys.2020.588313)
Supplement: Supplementary file 2 [file Data_Sheet_1.ZIP › Supplemental Quality Control/FastQC_raw_files/plasma_control_2_fastqc_raw.html]

SV18263\_0021\_S11\_R1\_001.fastq FastQC Report 

FastQC Report

Thu 7 May 2020  
SV18263\_0021\_S11\_R1\_001.fastq

## Summary

- Basic Statistics
- Per base sequence quality
- Per tile sequence quality
- Per sequence quality scores
- Per base sequence content
- Per sequence GC content
- Per base N content
- Sequence Length Distribution
- Sequence Duplication Levels
- Overrepresented sequences
- Adapter Content

## Basic Statistics

| Measure | Value |
| --- | --- |
| Filename | SV18263\_0021\_S11\_R1\_001.fastq |
| File type | Conventional base calls |
| Encoding | Sanger / Illumina 1.9 |
| Total Sequences | 27421054 |
| Sequences flagged as poor quality | 0 |
| Sequence length | 51 |
| %GC | 52 |

## Per base sequence quality

## Per tile sequence quality

## Per sequence quality scores

## Per base sequence content

## Per sequence GC content

## Per base N content

## Sequence Length Distribution

## Sequence Duplication Levels

## Overrepresented sequences

| Sequence | Count | Percentage | Possible Source |
| --- | --- | --- | --- |
| GCATTGGTGGTTCAGTGGTAGAATTCTCGCCTTGGAATTCTCGGGTGCCAA | 2200450 | 8.024673303951044 | No Hit |
| TGAGAACTGAATTCCATAGATGGTGGAATTCTCGGGTGCCAAGGAACTCCA | 1055932 | 3.8508074853723713 | RNA PCR Primer, Index 1 (100% over 28bp) |
| GCATTGGTGGTTCAGTGGTAGAATTCTCGCCTGGAATTCTCGGGTGCCAAG | 1036104 | 3.778498083990499 | No Hit |
| TAACGGAACCCATAATGCAGCTGTGGAATTCTCGGGTGCCAAGGAACTCCA | 628279 | 2.291228484506832 | RNA PCR Primer, Index 1 (100% over 28bp) |
| AACCCGTAGATCCGAACTTGTGTGGAATTCTCGGGTGCCAAGGAACTCCAG | 576749 | 2.1033071887025203 | RNA PCR Primer, Index 1 (100% over 29bp) |
| TTCAAGTAATCCAGGATAGGCTTGGAATTCTCGGGTGCCAAGGAACTCCAG | 557892 | 2.0345388619999802 | RNA PCR Primer, Index 1 (100% over 29bp) |
| TGAGGTAGTAGGTTGTATAGTTTGGAATTCTCGGGTGCCAAGGAACTCCAG | 411481 | 1.5006024203154262 | RNA PCR Primer, Index 1 (100% over 29bp) |
| AACCCGTAGATCCGAACTTGTTGGAATTCTCGGGTGCCAAGGAACTCCAGT | 314379 | 1.1464876587165467 | RNA PCR Primer, Index 1 (100% over 30bp) |
| TACCCTGTAGAACCGAATTTGTTGGAATTCTCGGGTGCCAAGGAACTCCAG | 291245 | 1.0621218279939204 | RNA PCR Primer, Index 1 (100% over 29bp) |
| AAACCGTTACCATTACTGAGATGGAATTCTCGGGTGCCAAGGAACTCCAGT | 274542 | 1.0012087792103104 | RNA PCR Primer, Index 1 (100% over 30bp) |
| TGAGGTAGTAGATTGAATAGTTTGGAATTCTCGGGTGCCAAGGAACTCCAG | 243152 | 0.8867346966312819 | RNA PCR Primer, Index 1 (100% over 29bp) |
| TGAGAACTGAATTCCATAGATGGTTGGAATTCTCGGGTGCCAAGGAACTCC | 239741 | 0.8742953498432263 | RNA PCR Primer, Index 1 (100% over 27bp) |
| GCATTGGTGGTTCAGTGGTAGAATTCTCGCCTGTGGAATTCTCGGGTGCCA | 213301 | 0.7778730897798458 | No Hit |
| GCATTGGTGGTTCAGTGGTAGAATTCTCGCTGGAATTCTCGGGTGCCAAGG | 182775 | 0.666549870767185 | Illumina Small RNA Adapter 2 (100% over 21bp) |
| TAACGGAACCCATAATGCAGCTTGGAATTCTCGGGTGCCAAGGAACTCCAG | 168293 | 0.6137364377022123 | RNA PCR Primer, Index 1 (100% over 29bp) |
| TCCCTGGTGGTCTAGTGGTTAGGATTCGGCGCTTGGAATTCTCGGGTGCCA | 163861 | 0.5975736745932523 | No Hit |
| TGAGGTAGTAGGTTGTATAGTTGGAATTCTCGGGTGCCAAGGAACTCCAGT | 160067 | 0.5837375908307536 | RNA PCR Primer, Index 1 (100% over 30bp) |
| GCCCGGCTAGCTCAGTCGGTAGAGCATGAGATGGAATTCTCGGGTGCCAAG | 141370 | 0.5155527573812444 | No Hit |
| AACCCGTAGATCCGAACTTGTGATGGAATTCTCGGGTGCCAAGGAACTCCA | 124922 | 0.4555696509696528 | RNA PCR Primer, Index 1 (100% over 28bp) |
| GCATTGTGGTTCAGTGGTAGAATTCTCGCCTTGGAATTCTCGGGTGCCAAG | 119096 | 0.4343232028936598 | No Hit |
| AAACCGTTACCATTACTGAGTTGGAATTCTCGGGTGCCAAGGAACTCCAGT | 112537 | 0.4104036263522183 | RNA PCR Primer, Index 1 (100% over 30bp) |
| TAGCTTATCAGACTGGTGTTGGCTGGAATTCTCGGGTGCCAAGGAACTCCA | 110575 | 0.4032485403369251 | RNA PCR Primer, Index 1 (100% over 28bp) |
| TATGGCACTGGTAGAATTCACTGTGGAATTCTCGGGTGCCAAGGAACTCCA | 110080 | 0.40144335808536025 | RNA PCR Primer, Index 1 (100% over 28bp) |
| CGAGCCGCGGCTGGGGGAGCATGGAATTCTCGGGTGCCAAGGAACTCCAGT | 107861 | 0.39335103603238597 | RNA PCR Primer, Index 1 (100% over 30bp) |
| GAGCCGCGGCTGGGGGAGCATGGAATTCTCGGGTGCCAAGGAACTCCAGTC | 102549 | 0.37397906003175513 | RNA PCR Primer, Index 1 (100% over 31bp) |
| TAGCTTATCAGACTGGTGTTGGTGGAATTCTCGGGTGCCAAGGAACTCCAG | 102248 | 0.3728813633494905 | RNA PCR Primer, Index 1 (100% over 29bp) |
| TGAGGTAGTAGGTTGTATAGTTTTGGAATTCTCGGGTGCCAAGGAACTCCA | 87256 | 0.3182080455404814 | RNA PCR Primer, Index 1 (100% over 28bp) |
| AAACCGTTACCATTACTGAGTGGAATTCTCGGGTGCCAAGGAACTCCAGTC | 82992 | 0.30265795034720405 | RNA PCR Primer, Index 1 (100% over 31bp) |
| TAACGGAACCCATAAAGCAGCTGTGGAATTCTCGGGTGCCAAGGAACTCCA | 79102 | 0.28847177063288665 | RNA PCR Primer, Index 1 (100% over 28bp) |
| AACATTCAACGCTGTCGGTGAGTGGAATTCTCGGGTGCCAAGGAACTCCAG | 78499 | 0.2862727304355259 | RNA PCR Primer, Index 1 (100% over 29bp) |
| TATGGCACTGGTAGAATTCACTTGGAATTCTCGGGTGCCAAGGAACTCCAG | 78207 | 0.28520785524874426 | RNA PCR Primer, Index 1 (100% over 29bp) |
| TGAGAACTGAATTCCATAGATGTGGAATTCTCGGGTGCCAAGGAACTCCAG | 76766 | 0.27995276913863343 | RNA PCR Primer, Index 1 (100% over 29bp) |
| TGAGGTAGTAGATTGAATAGTTGGAATTCTCGGGTGCCAAGGAACTCCAGT | 75468 | 0.275219180123419 | RNA PCR Primer, Index 1 (100% over 30bp) |
| GCCCGGCTAGCTCAGTCGGTAGAGCATGATGGAATTCTCGGGTGCCAAGGA | 75314 | 0.27465756786737666 | RNA PCR Primer, Index 1 (100% over 22bp) |
| GTTTCCGTAGTGTAGTGGTTATCACGTTCGCCTTGGAATTCTCGGGTGCCA | 73072 | 0.2664813686592791 | No Hit |
| GGTTGGCAGCGGCGACTCTGGACGCTGGAATTCTCGGGTGCCAAGGAACTC | 71803 | 0.2618535377961766 | RNA PCR Primer, Index 1 (100% over 26bp) |
| CCGTGTGAAAGTAGGTAATCGTCAGGCTTGGAATTCTCGGGTGCCAAGGAA | 70886 | 0.25850939208974244 | RNA PCR Primer, Index 1 (100% over 23bp) |
| TAACCGTTACCATTACTGAGATGGAATTCTCGGGTGCCAAGGAACTCCAGT | 64667 | 0.23582973871099194 | RNA PCR Primer, Index 1 (100% over 30bp) |
| TCGTACCGTGAGTAATAATGCATGGAATTCTCGGGTGCCAAGGAACTCCAG | 63359 | 0.2310596813674631 | RNA PCR Primer, Index 1 (100% over 29bp) |
| TCGCCACTGCTGGAAGTTCGTTGGAATTCTCGGGTGCCAAGGAACTCCAGT | 62798 | 0.229013808149023 | RNA PCR Primer, Index 1 (100% over 30bp) |
| GTTTCCGTAGTGTAGTGGTTATCACGTTCGCCTGGAATTCTCGGGTGCCAA | 61288 | 0.2235070905735425 | No Hit |
| TCCCTGGTCTAGTGGTTAGGATTCGGCGCTTGGAATTCTCGGGTGCCAAGG | 59189 | 0.21585238846034147 | Illumina Small RNA Adapter 2 (100% over 21bp) |
| TATTGCACTTGTCCCGGCCTGTTGGAATTCTCGGGTGCCAAGGAACTCCAG | 59091 | 0.21549499884285997 | RNA PCR Primer, Index 1 (100% over 29bp) |
| AAAGTAGGTAATCGTCAGGCTTGGAATTCTCGGGTGCCAAGGAACTCCAGT | 56212 | 0.20499576712113254 | RNA PCR Primer, Index 1 (100% over 30bp) |
| GCATTGTGGTTCAGTGGTAGAATTCTCGCCTGGAATTCTCGGGTGCCAAGG | 55968 | 0.20410593991026021 | Illumina Small RNA Adapter 2 (100% over 21bp) |
| AACCCGTAGATCCGAACTTGTGTTGGAATTCTCGGGTGCCAAGGAACTCCA | 55947 | 0.2040293564207999 | RNA PCR Primer, Index 1 (100% over 28bp) |
| TGAGGTAGTAGTTTGTATAGTTTGGAATTCTCGGGTGCCAAGGAACTCCAG | 55848 | 0.2036683199704869 | RNA PCR Primer, Index 1 (100% over 29bp) |
| CCCGTGTGAAAGTAGGTAATCGTCAGGCTTGGAATTCTCGGGTGCCAAGGA | 55280 | 0.2015969189222267 | RNA PCR Primer, Index 1 (100% over 22bp) |
| TGAAAGTAGGTAATCGTCAGGCTTGGAATTCTCGGGTGCCAAGGAACTCCA | 53482 | 0.19503991349129032 | RNA PCR Primer, Index 1 (100% over 28bp) |
| GAAAGTAGGTAATCGTCAGGCTTGGAATTCTCGGGTGCCAAGGAACTCCAG | 51655 | 0.18837714990824203 | RNA PCR Primer, Index 1 (100% over 29bp) |
| AAGCTGCCAGCTGAAGAACTGTTGGAATTCTCGGGTGCCAAGGAACTCCAG | 51522 | 0.18789212114165998 | RNA PCR Primer, Index 1 (100% over 29bp) |
| GAGCCGCGGCTGGGGGAGCAGTTTGGAATTCTCGGGTGCCAAGGAACTCCA | 49814 | 0.18166333066555357 | RNA PCR Primer, Index 1 (100% over 28bp) |
| GTAGGTAATCGTCAGGCTTGGAATTCTCGGGTGCCAAGGAACTCCAGTCAC | 48844 | 0.17812590281905283 | RNA PCR Primer, Index 1 (100% over 33bp) |
| AAGTAGGTAATCGTCAGGCTTGGAATTCTCGGGTGCCAAGGAACTCCAGTC | 48526 | 0.17696620997865362 | RNA PCR Primer, Index 1 (100% over 31bp) |
| GTGAAAGTAGGTAATCGTCAGGCTTGGAATTCTCGGGTGCCAAGGAACTCC | 47406 | 0.17288175720743632 | RNA PCR Primer, Index 1 (100% over 27bp) |
| CGAGCCGCGGCTGGGGGAGCAGTGGAATTCTCGGGTGCCAAGGAACTCCAG | 47000 | 0.17140114307787002 | RNA PCR Primer, Index 1 (100% over 29bp) |
| AACCCGTAGATCCGATCTTGTTGGAATTCTCGGGTGCCAAGGAACTCCAGT | 45025 | 0.16419864823576802 | RNA PCR Primer, Index 1 (100% over 30bp) |
| AGTAGGTAATCGTCAGGCTTGGAATTCTCGGGTGCCAAGGAACTCCAGTCA | 44151 | 0.16101131634108592 | RNA PCR Primer, Index 1 (100% over 32bp) |
| CGAGCCGCGGCTGGGGGAGCAGTTTGGAATTCTCGGGTGCCAAGGAACTCC | 43822 | 0.15981150833954083 | RNA PCR Primer, Index 1 (100% over 27bp) |
| GCCCGGCTAGCTCAGTCGGTAGAGCATGAGTGGAATTCTCGGGTGCCAAGG | 43054 | 0.15701074072499183 | Illumina Small RNA Adapter 2 (100% over 21bp) |
| TAGCAGCACGTAAATATTGGAGTGGAATTCTCGGGTGCCAAGGAACTCCAG | 42999 | 0.15681016491926242 | RNA PCR Primer, Index 1 (100% over 29bp) |
| TTCAAGTAATCCAGGATAGGCTGGAATTCTCGGGTGCCAAGGAACTCCAGT | 41364 | 0.1508475932398514 | RNA PCR Primer, Index 1 (100% over 30bp) |
| GCTGTGTGAGGTCGGACCTATCTGGAATTCTCGGGTGCCAAGGAACTCCAG | 41176 | 0.1501619886675399 | RNA PCR Primer, Index 1 (100% over 29bp) |
| TAACGGAACCCATAATGCAGCTGGAATTCTCGGGTGCCAAGGAACTCCAGT | 39737 | 0.14491419622309196 | RNA PCR Primer, Index 1 (100% over 30bp) |
| GTGTGAAAGTAGGTAATCGTCAGGCTTGGAATTCTCGGGTGCCAAGGAACT | 39183 | 0.14289385083447195 | RNA PCR Primer, Index 1 (100% over 25bp) |
| TTTGGCAATGGTAGAACTCACACTTGGAATTCTCGGGTGCCAAGGAACTCC | 38235 | 0.139436653310263 | RNA PCR Primer, Index 1 (100% over 27bp) |
| TGTGAAAGTAGGTAATCGTCAGGCTTGGAATTCTCGGGTGCCAAGGAACTC | 35285 | 0.12867849645750307 | RNA PCR Primer, Index 1 (100% over 26bp) |
| GAGCCGCGGCTGGGGGAGCAGTGGAATTCTCGGGTGCCAAGGAACTCCAGT | 35200 | 0.1283685156668303 | RNA PCR Primer, Index 1 (100% over 30bp) |
| TTTGGCAATGGTAGAACTCACACTGGAATTCTCGGGTGCCAAGGAACTCCA | 34585 | 0.12612571347549223 | RNA PCR Primer, Index 1 (100% over 28bp) |
| CGTGTGAAAGTAGGTAATCGTCAGGCTTGGAATTCTCGGGTGCCAAGGAAC | 33642 | 0.12268675011544049 | RNA PCR Primer, Index 1 (100% over 24bp) |
| GGAATACCAGGTGCTGTAAGCTTTGGAATTCTCGGGTGCCAAGGAACTCCA | 33470 | 0.12205949486843212 | RNA PCR Primer, Index 1 (100% over 28bp) |
| TAACGGAACCCATAAAGCAGCTTGGAATTCTCGGGTGCCAAGGAACTCCAG | 31613 | 0.11528732630044054 | RNA PCR Primer, Index 1 (100% over 29bp) |
| TATTGCACTTGTCCCGGCCTGTATTGGAATTCTCGGGTGCCAAGGAACTCC | 30426 | 0.11095853572951646 | RNA PCR Primer, Index 1 (100% over 27bp) |
| GAATACCAGGTGCTGTAAGCTTTGGAATTCTCGGGTGCCAAGGAACTCCAG | 30307 | 0.11052456262257461 | RNA PCR Primer, Index 1 (100% over 29bp) |
| TCCCTGTGGTCTAGTGGTTAGGATTCGGCGCTTGGAATTCTCGGGTGCCAA | 30158 | 0.10998118453068943 | No Hit |
| GCCCGGCTAGCTCAGTCGGTAGAGCATGAGACTGGAATTCTCGGGTGCCAA | 29466 | 0.10745757621133016 | No Hit |
| TGCGAGTTCGAGTCTCGCCGTCGGCACCATGGAATTCTCGGGTGCCAAGGA | 29171 | 0.10638176052605419 | RNA PCR Primer, Index 1 (100% over 22bp) |
| TGGAATTCTCGGGTGCCAAGGAACTCCAGTCACGGCTACATCTCGTATGCC | 28834 | 0.10515277786185755 | RNA PCR Primer, Index 11 (100% over 51bp) |
| TACCCTGTAGATCCGGATTTGTTGGAATTCTCGGGTGCCAAGGAACTCCAG | 28631 | 0.10441247079707439 | RNA PCR Primer, Index 1 (100% over 29bp) |
| AAACCGTTACCATTACTGATGGAATTCTCGGGTGCCAAGGAACTCCAGTCA | 28136 | 0.1026072885455096 | RNA PCR Primer, Index 1 (100% over 32bp) |
| GCATTGGTGGTTCAGTGGTAGAATTCTCTGGAATTCTCGGGTGCCAAGGAA | 28057 | 0.10231918875182552 | RNA PCR Primer, Index 1 (100% over 23bp) |

## Adapter Content

Produced by FastQC (version 0.11.9)
