# Supplementary material for: High Throughput Sequencing of MicroRNA in Rainbow Trout Plasma, Mucus, and Surrounding Water Following Acute Stress
Source: Front Physiol. 2021 Jan 13;11:588313. doi: 10.3389/fphys.2020.588313 (PMC7838646; doi:10.3389/fphys.2020.588313)
Supplement: Supplementary file 2 [file Data_Sheet_1.ZIP › Supplemental Quality Control/FastQC_raw_files/plasma_control_3_fastqc_raw.html]

SV18263\_0023\_S13\_R1\_001.fastq FastQC Report 

FastQC Report

Thu 7 May 2020  
SV18263\_0023\_S13\_R1\_001.fastq

## Summary

- Basic Statistics
- Per base sequence quality
- Per tile sequence quality
- Per sequence quality scores
- Per base sequence content
- Per sequence GC content
- Per base N content
- Sequence Length Distribution
- Sequence Duplication Levels
- Overrepresented sequences
- Adapter Content

## Basic Statistics

| Measure | Value |
| --- | --- |
| Filename | SV18263\_0023\_S13\_R1\_001.fastq |
| File type | Conventional base calls |
| Encoding | Sanger / Illumina 1.9 |
| Total Sequences | 18098085 |
| Sequences flagged as poor quality | 0 |
| Sequence length | 51 |
| %GC | 52 |

## Per base sequence quality

## Per tile sequence quality

## Per sequence quality scores

## Per base sequence content

## Per sequence GC content

## Per base N content

## Sequence Length Distribution

## Sequence Duplication Levels

## Overrepresented sequences

| Sequence | Count | Percentage | Possible Source |
| --- | --- | --- | --- |
| AACCCGTAGATCCGAACTTGTGTGGAATTCTCGGGTGCCAAGGAACTCCAG | 1115057 | 6.161187772076438 | RNA PCR Primer, Index 1 (100% over 29bp) |
| GCATTGGTGGTTCAGTGGTAGAATTCTCGCCTTGGAATTCTCGGGTGCCAA | 1049381 | 5.7982985492664 | No Hit |
| TGAGAACTGAATTCCATAGATGGTGGAATTCTCGGGTGCCAAGGAACTCCA | 1008359 | 5.571633683895285 | RNA PCR Primer, Index 1 (100% over 28bp) |
| TCCCTGGTGGTCTAGTGGTTAGGATTCGGCGCTTGGAATTCTCGGGTGCCA | 596995 | 3.2986639194146785 | No Hit |
| GCATTGGTGGTTCAGTGGTAGAATTCTCGCCTGGAATTCTCGGGTGCCAAG | 507795 | 2.8057940936844976 | No Hit |
| TCCCTGGTCTAGTGGTTAGGATTCGGCGCTTGGAATTCTCGGGTGCCAAGG | 418849 | 2.314327731359423 | Illumina Small RNA Adapter 2 (100% over 21bp) |
| TTCAAGTAATCCAGGATAGGCTTGGAATTCTCGGGTGCCAAGGAACTCCAG | 389441 | 2.151835401369813 | RNA PCR Primer, Index 1 (100% over 29bp) |
| AACCCGTAGATCCGAACTTGTTGGAATTCTCGGGTGCCAAGGAACTCCAGT | 292252 | 1.6148227837365112 | RNA PCR Primer, Index 1 (100% over 30bp) |
| TGAGGTAGTAGATTGAATAGTTTGGAATTCTCGGGTGCCAAGGAACTCCAG | 275305 | 1.5211830422942538 | RNA PCR Primer, Index 1 (100% over 29bp) |
| TGAGGTAGTAGGTTGTATAGTTTGGAATTCTCGGGTGCCAAGGAACTCCAG | 264536 | 1.4616795091856403 | RNA PCR Primer, Index 1 (100% over 29bp) |
| TAACGGAACCCATAATGCAGCTGTGGAATTCTCGGGTGCCAAGGAACTCCA | 224388 | 1.2398438840352446 | RNA PCR Primer, Index 1 (100% over 28bp) |
| TGAGAACTGAATTCCATAGATGGTTGGAATTCTCGGGTGCCAAGGAACTCC | 188475 | 1.041408524714079 | RNA PCR Primer, Index 1 (100% over 27bp) |
| TACCCTGTAGAACCGAATTTGTTGGAATTCTCGGGTGCCAAGGAACTCCAG | 171889 | 0.9497634694499445 | RNA PCR Primer, Index 1 (100% over 29bp) |
| GGTTGGCAGCGGCGACTCTGGACGCTGGAATTCTCGGGTGCCAAGGAACTC | 153665 | 0.8490677328568188 | RNA PCR Primer, Index 1 (100% over 26bp) |
| AACATTCAACGCTGTCGGTGAGTGGAATTCTCGGGTGCCAAGGAACTCCAG | 130538 | 0.7212807321879635 | RNA PCR Primer, Index 1 (100% over 29bp) |
| GAGCCGCGGCTGGGGGAGCATGGAATTCTCGGGTGCCAAGGAACTCCAGTC | 120707 | 0.6669600678745845 | RNA PCR Primer, Index 1 (100% over 31bp) |
| GCATTGTGGTTCAGTGGTAGAATTCTCGCCTTGGAATTCTCGGGTGCCAAG | 112406 | 0.621093336670703 | No Hit |
| AACCCGTAGATCCGAACTTGTGATGGAATTCTCGGGTGCCAAGGAACTCCA | 109534 | 0.6052242543893457 | RNA PCR Primer, Index 1 (100% over 28bp) |
| AACCCGTAGATCCGAACTTGTGTTGGAATTCTCGGGTGCCAAGGAACTCCA | 108801 | 0.6011741021218544 | RNA PCR Primer, Index 1 (100% over 28bp) |
| GCATTGGTGGTTCAGTGGTAGAATTCTCGCTGGAATTCTCGGGTGCCAAGG | 101611 | 0.5614461419536929 | Illumina Small RNA Adapter 2 (100% over 21bp) |
| CGAGCCGCGGCTGGGGGAGCATGGAATTCTCGGGTGCCAAGGAACTCCAGT | 100093 | 0.5530585142019169 | RNA PCR Primer, Index 1 (100% over 30bp) |
| GCATTGGTGGTTCAGTGGTAGAATTCTCGCCTGTGGAATTCTCGGGTGCCA | 83437 | 0.4610266776844069 | No Hit |
| TCGCCACTGCTGGAAGTTCGTTGGAATTCTCGGGTGCCAAGGAACTCCAGT | 82610 | 0.4564571334480969 | RNA PCR Primer, Index 1 (100% over 30bp) |
| TAGCTTATCAGACTGGTGTTGGCTGGAATTCTCGGGTGCCAAGGAACTCCA | 79001 | 0.43651579711334104 | RNA PCR Primer, Index 1 (100% over 28bp) |
| TGAGAACTGAATTCCATAGATGTGGAATTCTCGGGTGCCAAGGAACTCCAG | 73509 | 0.40617004506277876 | RNA PCR Primer, Index 1 (100% over 29bp) |
| GAGCCGCGGCTGGGGGAGCAGTTTGGAATTCTCGGGTGCCAAGGAACTCCA | 70823 | 0.3913286958261053 | RNA PCR Primer, Index 1 (100% over 28bp) |
| GCCCGGCTAGCTCAGTCGGTAGAGCATGAGATGGAATTCTCGGGTGCCAAG | 70596 | 0.39007441947587274 | No Hit |
| TGAGGTAGTAGGTTGTATAGTTGGAATTCTCGGGTGCCAAGGAACTCCAGT | 67106 | 0.37079061127185553 | RNA PCR Primer, Index 1 (100% over 30bp) |
| GCCCGGCTAGCTCAGTCGGTAGAGCATGATGGAATTCTCGGGTGCCAAGGA | 64905 | 0.3586291035764281 | RNA PCR Primer, Index 1 (100% over 22bp) |
| TAGCTTATCAGACTGGTGTTGGTGGAATTCTCGGGTGCCAAGGAACTCCAG | 64581 | 0.3568388589179463 | RNA PCR Primer, Index 1 (100% over 29bp) |
| GTTTCCGTAGTGTAGTGGTTATCACGTTCGCCTTGGAATTCTCGGGTGCCA | 62839 | 0.34721353115536807 | No Hit |
| CGAGCCGCGGCTGGGGGAGCAGTTTGGAATTCTCGGGTGCCAAGGAACTCC | 59997 | 0.33151021226831456 | RNA PCR Primer, Index 1 (100% over 27bp) |
| TAACGGAACCCATAAAGCAGCTGTGGAATTCTCGGGTGCCAAGGAACTCCA | 59366 | 0.32802365554145646 | RNA PCR Primer, Index 1 (100% over 28bp) |
| AACCCGTAGATCCGAACTTGTGCTGGAATTCTCGGGTGCCAAGGAACTCCA | 55930 | 0.30903822144718623 | RNA PCR Primer, Index 1 (100% over 28bp) |
| GCATTGTGGTTCAGTGGTAGAATTCTCGCCTGGAATTCTCGGGTGCCAAGG | 51645 | 0.2853616832941165 | Illumina Small RNA Adapter 2 (100% over 21bp) |
| TCGTACCGTGAGTAATAATGCATGGAATTCTCGGGTGCCAAGGAACTCCAG | 46671 | 0.25787811251853443 | RNA PCR Primer, Index 1 (100% over 29bp) |
| TGAGGTAGTAGATTGAATAGTTGGAATTCTCGGGTGCCAAGGAACTCCAGT | 45414 | 0.25093262629720214 | RNA PCR Primer, Index 1 (100% over 30bp) |
| CGAGCCGCGGCTGGGGGAGCAGTGGAATTCTCGGGTGCCAAGGAACTCCAG | 43170 | 0.23853352440327252 | RNA PCR Primer, Index 1 (100% over 29bp) |
| TCCCTGGTGTCTAGTGGTTAGGATTCGGCGCTTGGAATTCTCGGGTGCCAA | 39569 | 0.21863639164033102 | No Hit |
| GAGCCGCGGCTGGGGGAGCAGTGGAATTCTCGGGTGCCAAGGAACTCCAGT | 36118 | 0.1995680758489089 | RNA PCR Primer, Index 1 (100% over 30bp) |
| TAGCAGCACGTAAATATTGGAGTGGAATTCTCGGGTGCCAAGGAACTCCAG | 35982 | 0.19881661512806464 | RNA PCR Primer, Index 1 (100% over 29bp) |
| TCGTTTCCCGGCCAATGCACCATGGAATTCTCGGGTGCCAAGGAACTCCAG | 35329 | 0.19520849857871703 | RNA PCR Primer, Index 1 (100% over 29bp) |
| TAACGGAACCCATAATGCAGCTTGGAATTCTCGGGTGCCAAGGAACTCCAG | 35313 | 0.1951200914350883 | RNA PCR Primer, Index 1 (100% over 29bp) |
| GCATTGGTGGTTCAGTGGTAGAATTCTCTGGAATTCTCGGGTGCCAAGGAA | 33566 | 0.18546713644012613 | RNA PCR Primer, Index 1 (100% over 23bp) |
| TATTGCACTTGTCCCGGCCTGTTGGAATTCTCGGGTGCCAAGGAACTCCAG | 30221 | 0.16698451797524436 | RNA PCR Primer, Index 1 (100% over 29bp) |
| TGAGGTAGTAGGTTGTATAGTTTTGGAATTCTCGGGTGCCAAGGAACTCCA | 29707 | 0.16414443848617133 | RNA PCR Primer, Index 1 (100% over 28bp) |
| TCCCTGTGGTCTAGTGGTTAGGATTCGGCGCTTGGAATTCTCGGGTGCCAA | 28141 | 0.15549158930350918 | No Hit |
| GTTTCCGTAGTGTAGTGGTTATCACGTTCGCCTGGAATTCTCGGGTGCCAA | 28080 | 0.15515453706842464 | No Hit |
| GGTTGGCAGCGGCGACTCTGGACGTGGAATTCTCGGGTGCCAAGGAACTCC | 26603 | 0.14699345262219732 | RNA PCR Primer, Index 1 (100% over 27bp) |
| TGAGGTAGTAGTTTGTATAGTTTGGAATTCTCGGGTGCCAAGGAACTCCAG | 26465 | 0.1462309410083995 | RNA PCR Primer, Index 1 (100% over 29bp) |
| GGAATACCAGGTGCTGTAAGCTTTGGAATTCTCGGGTGCCAAGGAACTCCA | 25888 | 0.14304275839128835 | RNA PCR Primer, Index 1 (100% over 28bp) |
| ACCATCGACCGTTGATTGTACCTGGAATTCTCGGGTGCCAAGGAACTCCAG | 25182 | 0.13914179317867056 | RNA PCR Primer, Index 1 (100% over 29bp) |
| AACCCGTAGATCCGAACTTGTGGAATTCTCGGGTGCCAAGGAACTCCAGTC | 24545 | 0.13562208377295165 | RNA PCR Primer, Index 1 (100% over 31bp) |
| GCCCGGCTAGCTCAGTCGGTAGAGCATGAGTGGAATTCTCGGGTGCCAAGG | 23982 | 0.13251125740651565 | Illumina Small RNA Adapter 2 (100% over 21bp) |
| TTCAAGTAATCCAGGATAGGCTGGAATTCTCGGGTGCCAAGGAACTCCAGT | 23602 | 0.1304115877453333 | RNA PCR Primer, Index 1 (100% over 30bp) |
| GCCCGGCTAGCTCAGTCGGTAGAGCATGAGACTCTTAATCTTGGAATTCTC | 21073 | 0.11643773360551683 | No Hit |
| TCCCTGGTGGTCTAGTGGTTAGGATTCGGCGCTCTGGAATTCTCGGGTGCC | 20194 | 0.11158086615241337 | No Hit |
| TGGAATTCTCGGGTGCCAAGGAACTCCAGTCACAGTCAAATCTCGTATGCC | 19409 | 0.1072433906681287 | RNA PCR Primer, Index 13 (100% over 51bp) |
| CCGTGTGAAAGTAGGTAATCGTCAGGCTTGGAATTCTCGGGTGCCAAGGAA | 19329 | 0.10680135494998504 | RNA PCR Primer, Index 1 (100% over 23bp) |
| AAAGTAGGTAATCGTCAGGCTTGGAATTCTCGGGTGCCAAGGAACTCCAGT | 19197 | 0.106071996015048 | RNA PCR Primer, Index 1 (100% over 30bp) |
| TCGATTCCCGGCCAATGCACCATGGAATTCTCGGGTGCCAAGGAACTCCAG | 18777 | 0.10375130849479379 | RNA PCR Primer, Index 1 (100% over 29bp) |
| GTAGGTAATCGTCAGGCTTGGAATTCTCGGGTGCCAAGGAACTCCAGTCAC | 18265 | 0.10092227989867436 | RNA PCR Primer, Index 1 (100% over 33bp) |

## Adapter Content

Produced by FastQC (version 0.11.9)
