# Supplementary material for: High Throughput Sequencing of MicroRNA in Rainbow Trout Plasma, Mucus, and Surrounding Water Following Acute Stress
Source: Front Physiol. 2021 Jan 13;11:588313. doi: 10.3389/fphys.2020.588313 (PMC7838646; doi:10.3389/fphys.2020.588313)
Supplement: Supplementary file 2 [file Data_Sheet_1.ZIP › Supplemental Quality Control/FastQC_raw_files/plasma_stressed_1_fastqc_raw.html]

SV18263\_0020\_S10\_R1\_001.fastq FastQC Report 

FastQC Report

Thu 7 May 2020  
SV18263\_0020\_S10\_R1\_001.fastq

## Summary

- Basic Statistics
- Per base sequence quality
- Per tile sequence quality
- Per sequence quality scores
- Per base sequence content
- Per sequence GC content
- Per base N content
- Sequence Length Distribution
- Sequence Duplication Levels
- Overrepresented sequences
- Adapter Content

## Basic Statistics

| Measure | Value |
| --- | --- |
| Filename | SV18263\_0020\_S10\_R1\_001.fastq |
| File type | Conventional base calls |
| Encoding | Sanger / Illumina 1.9 |
| Total Sequences | 17978832 |
| Sequences flagged as poor quality | 0 |
| Sequence length | 51 |
| %GC | 52 |

## Per base sequence quality

## Per tile sequence quality

## Per sequence quality scores

## Per base sequence content

## Per sequence GC content

## Per base N content

## Sequence Length Distribution

## Sequence Duplication Levels

## Overrepresented sequences

| Sequence | Count | Percentage | Possible Source |
| --- | --- | --- | --- |
| GCATTGGTGGTTCAGTGGTAGAATTCTCGCCTGGAATTCTCGGGTGCCAAG | 2992752 | 16.645975667384842 | No Hit |
| GCATTGGTGGTTCAGTGGTAGAATTCTCGCCTTGGAATTCTCGGGTGCCAA | 2186278 | 12.1602893892106 | No Hit |
| GCATTGGTGGTTCAGTGGTAGAATTCTCGCTGGAATTCTCGGGTGCCAAGG | 454268 | 2.526682489719021 | Illumina Small RNA Adapter 2 (100% over 21bp) |
| TGAGAACTGAATTCCATAGATGGTGGAATTCTCGGGTGCCAAGGAACTCCA | 439412 | 2.4440519829096794 | RNA PCR Primer, Index 1 (100% over 28bp) |
| AACCCGTAGATCCGAACTTGTGTGGAATTCTCGGGTGCCAAGGAACTCCAG | 361545 | 2.0109482084264427 | RNA PCR Primer, Index 1 (100% over 29bp) |
| TTCAAGTAATCCAGGATAGGCTTGGAATTCTCGGGTGCCAAGGAACTCCAG | 199780 | 1.1111956549791444 | RNA PCR Primer, Index 1 (100% over 29bp) |
| TCCCTGGTGGTCTAGTGGTTAGGATTCGGCGCTTGGAATTCTCGGGTGCCA | 192444 | 1.0703921144599382 | No Hit |
| TAACGGAACCCATAATGCAGCTGTGGAATTCTCGGGTGCCAAGGAACTCCA | 188309 | 1.0473928450969452 | RNA PCR Primer, Index 1 (100% over 28bp) |
| GCATTGTGGTTCAGTGGTAGAATTCTCGCCTGGAATTCTCGGGTGCCAAGG | 179783 | 0.9999704096461883 | Illumina Small RNA Adapter 2 (100% over 21bp) |
| GTTTCCGTAGTGTAGTGGTTATCACGTTCGCCTGGAATTCTCGGGTGCCAA | 153964 | 0.8563626380178645 | No Hit |
| AACCCGTAGATCCGAACTTGTTGGAATTCTCGGGTGCCAAGGAACTCCAGT | 143012 | 0.7954465562612744 | RNA PCR Primer, Index 1 (100% over 30bp) |
| TACCCTGTAGAACCGAATTTGTTGGAATTCTCGGGTGCCAAGGAACTCCAG | 142319 | 0.7915920233305479 | RNA PCR Primer, Index 1 (100% over 29bp) |
| GCATTGTGGTTCAGTGGTAGAATTCTCGCCTTGGAATTCTCGGGTGCCAAG | 129402 | 0.7197464217920274 | No Hit |
| TGAGGTAGTAGGTTGTATAGTTTGGAATTCTCGGGTGCCAAGGAACTCCAG | 121747 | 0.6771685724634392 | RNA PCR Primer, Index 1 (100% over 29bp) |
| TCCCTGGTCTAGTGGTTAGGATTCGGCGCTTGGAATTCTCGGGTGCCAAGG | 95979 | 0.5338444677607533 | Illumina Small RNA Adapter 2 (100% over 21bp) |
| GTTTCCGTAGTGTAGTGGTTATCACGTTCGCCTTGGAATTCTCGGGTGCCA | 92994 | 0.5172416094660655 | No Hit |
| TGAGGTAGTAGATTGAATAGTTTGGAATTCTCGGGTGCCAAGGAACTCCAG | 82779 | 0.46042479288977173 | RNA PCR Primer, Index 1 (100% over 29bp) |
| GCCCGGCTAGCTCAGTCGGTAGAGCATGATGGAATTCTCGGGTGCCAAGGA | 72498 | 0.40324087793912305 | RNA PCR Primer, Index 1 (100% over 22bp) |
| TGAGAACTGAATTCCATAGATGGTTGGAATTCTCGGGTGCCAAGGAACTCC | 67160 | 0.3735504063890246 | RNA PCR Primer, Index 1 (100% over 27bp) |
| TAGCTTATCAGACTGGTGTTGGTGGAATTCTCGGGTGCCAAGGAACTCCAG | 63860 | 0.35519548767127923 | RNA PCR Primer, Index 1 (100% over 29bp) |
| AACATTCAACGCTGTCGGTGAGTGGAATTCTCGGGTGCCAAGGAACTCCAG | 56420 | 0.313813489107635 | RNA PCR Primer, Index 1 (100% over 29bp) |
| GAGCCGCGGCTGGGGGAGCATGGAATTCTCGGGTGCCAAGGAACTCCAGTC | 54764 | 0.3046026571692755 | RNA PCR Primer, Index 1 (100% over 31bp) |
| AACCCGTAGATCCGAACTTGTGATGGAATTCTCGGGTGCCAAGGAACTCCA | 53159 | 0.2956754921565539 | RNA PCR Primer, Index 1 (100% over 28bp) |
| TAGCTTATCAGACTGGTGTTGGCTGGAATTCTCGGGTGCCAAGGAACTCCA | 52254 | 0.29064179475062674 | RNA PCR Primer, Index 1 (100% over 28bp) |
| GCATTGGTGGTTCAGTGGTAGAATTCTCGCCTGTGGAATTCTCGGGTGCCA | 51767 | 0.287933053715614 | No Hit |
| TGAGGTAGTAGGTTGTATAGTTGGAATTCTCGGGTGCCAAGGAACTCCAGT | 49146 | 0.2733547985764593 | RNA PCR Primer, Index 1 (100% over 30bp) |
| GCATTGGTGGTTCAGTGGTAGAATTCTCGGGTGCCAAGGAACTCCAGTCAC | 48876 | 0.27185303249955284 | RNA PCR Primer, Index 1 (96% over 33bp) |
| TCGTACCGTGAGTAATAATGCATGGAATTCTCGGGTGCCAAGGAACTCCAG | 44349 | 0.2466734212767548 | RNA PCR Primer, Index 1 (100% over 29bp) |
| GCCCGGCTAGCTCAGTCGGTAGAGCATGAGATGGAATTCTCGGGTGCCAAG | 43139 | 0.2399432844135815 | No Hit |
| AAGCTGCCAGCTGAAGAACTGTTGGAATTCTCGGGTGCCAAGGAACTCCAG | 42579 | 0.2368285103281459 | RNA PCR Primer, Index 1 (100% over 29bp) |
| TGAGAACTGAATTCCATAGATGTGGAATTCTCGGGTGCCAAGGAACTCCAG | 41542 | 0.23106061617350895 | RNA PCR Primer, Index 1 (100% over 29bp) |
| TAACGGAACCCATAATGCAGCTTGGAATTCTCGGGTGCCAAGGAACTCCAG | 39973 | 0.2223336866377082 | RNA PCR Primer, Index 1 (100% over 29bp) |
| AAACCGTTACCATTACTGAGATGGAATTCTCGGGTGCCAAGGAACTCCAGT | 35121 | 0.1953463940260413 | RNA PCR Primer, Index 1 (100% over 30bp) |
| CCGTGTGAAAGTAGGTAATCGTCAGGCTTGGAATTCTCGGGTGCCAAGGAA | 34391 | 0.19128606352181277 | RNA PCR Primer, Index 1 (100% over 23bp) |
| TAACGGAACCCATAAAGCAGCTGTGGAATTCTCGGGTGCCAAGGAACTCCA | 34280 | 0.19066867080130678 | RNA PCR Primer, Index 1 (100% over 28bp) |
| TATTGCACTTGTCCCGGCCTGTTGGAATTCTCGGGTGCCAAGGAACTCCAG | 33286 | 0.18513994679965862 | RNA PCR Primer, Index 1 (100% over 29bp) |
| CGAGCCGCGGCTGGGGGAGCATGGAATTCTCGGGTGCCAAGGAACTCCAGT | 33272 | 0.18506207744752273 | RNA PCR Primer, Index 1 (100% over 30bp) |
| AAAGTAGGTAATCGTCAGGCTTGGAATTCTCGGGTGCCAAGGAACTCCAGT | 30547 | 0.16990536426392994 | RNA PCR Primer, Index 1 (100% over 30bp) |
| CCCGTGTGAAAGTAGGTAATCGTCAGGCTTGGAATTCTCGGGTGCCAAGGA | 30525 | 0.16978299813914496 | RNA PCR Primer, Index 1 (100% over 22bp) |
| GTAGGTAATCGTCAGGCTTGGAATTCTCGGGTGCCAAGGAACTCCAGTCAC | 30369 | 0.16891531107248792 | RNA PCR Primer, Index 1 (100% over 33bp) |
| TGAAAGTAGGTAATCGTCAGGCTTGGAATTCTCGGGTGCCAAGGAACTCCA | 29414 | 0.16360350883750402 | RNA PCR Primer, Index 1 (100% over 28bp) |
| GAGCCGCGGCTGGGGGAGCTGGAATTCTCGGGTGCCAAGGAACTCCAGTCA | 28998 | 0.16128967665975186 | RNA PCR Primer, Index 1 (100% over 32bp) |
| GAAAGTAGGTAATCGTCAGGCTTGGAATTCTCGGGTGCCAAGGAACTCCAG | 28930 | 0.16091145409223467 | RNA PCR Primer, Index 1 (100% over 29bp) |
| GCATTGTGGTTCAGTGGTAGAATTCTCGCTGGAATTCTCGGGTGCCAAGGA | 28079 | 0.15617810990168884 | RNA PCR Primer, Index 1 (100% over 22bp) |
| AAGTAGGTAATCGTCAGGCTTGGAATTCTCGGGTGCCAAGGAACTCCAGTC | 27671 | 0.15390877449658577 | RNA PCR Primer, Index 1 (100% over 31bp) |
| GCATTGGTGGTTCAGTGGTAGAATTCTCGTGGAATTCTCGGGTGCCAAGGA | 26898 | 0.14960927383936845 | RNA PCR Primer, Index 1 (100% over 22bp) |
| AGTAGGTAATCGTCAGGCTTGGAATTCTCGGGTGCCAAGGAACTCCAGTCA | 26784 | 0.14897519482911906 | RNA PCR Primer, Index 1 (100% over 32bp) |
| TACCCTGTAGATCCGGATTTGTTGGAATTCTCGGGTGCCAAGGAACTCCAG | 26292 | 0.14623864331120062 | RNA PCR Primer, Index 1 (100% over 29bp) |
| GTGAAAGTAGGTAATCGTCAGGCTTGGAATTCTCGGGTGCCAAGGAACTCC | 26037 | 0.1448203086830112 | RNA PCR Primer, Index 1 (100% over 27bp) |
| CGAGCCGCGGCTGGGGGAGCAGTGGAATTCTCGGGTGCCAAGGAACTCCAG | 25963 | 0.14440871353600723 | RNA PCR Primer, Index 1 (100% over 29bp) |
| AACCCGTAGATCCGAACTTGTGTTGGAATTCTCGGGTGCCAAGGAACTCCA | 25300 | 0.14072104350271475 | RNA PCR Primer, Index 1 (100% over 28bp) |
| TGAGGTAGTAGATTGAATAGTTGGAATTCTCGGGTGCCAAGGAACTCCAGT | 24334 | 0.13534805820533835 | RNA PCR Primer, Index 1 (100% over 30bp) |
| GAGCCGCGGCTGGGGGAGCAGTGGAATTCTCGGGTGCCAAGGAACTCCAGT | 23744 | 0.13206642122246873 | RNA PCR Primer, Index 1 (100% over 30bp) |
| GTGTGAAAGTAGGTAATCGTCAGGCTTGGAATTCTCGGGTGCCAAGGAACT | 22296 | 0.12401250537298529 | RNA PCR Primer, Index 1 (100% over 25bp) |
| GCATTGGTGGTTCAGTGGTAGAATTCTCTGGAATTCTCGGGTGCCAAGGAA | 21805 | 0.12128151595164802 | RNA PCR Primer, Index 1 (100% over 23bp) |
| AACTCTTAGCGGTGGATCACTCGGTGGAATTCTCGGGTGCCAAGGAACTCC | 21571 | 0.11997998535166245 | RNA PCR Primer, Index 1 (100% over 27bp) |
| AACCCGTAGATCCGATCTTGTTGGAATTCTCGGGTGCCAAGGAACTCCAGT | 21320 | 0.11858389910979757 | RNA PCR Primer, Index 1 (100% over 30bp) |
| TCCCTGTGGTCTAGTGGTTAGGATTCGGCGCTTGGAATTCTCGGGTGCCAA | 20990 | 0.11674840723802302 | No Hit |
| GCATTGGTGGTTCAGTGGTAGAATTCTCGCCCTGGAATTCTCGGGTGCCAA | 20904 | 0.11627006693204542 | No Hit |
| GCATTGGTGGTTCAGTGGTAGAATTCTCGCCTGGAATCTCGGGTGCCAAGG | 20805 | 0.11571941937051305 | No Hit |
| CCCAGTGTTCAGACTACCTGTTCTGGAATTCTCGGGTGCCAAGGAACTCCA | 20551 | 0.11430664683890476 | RNA PCR Primer, Index 1 (100% over 28bp) |
| GCCCGGCTAGCTCAGTCGGTAGAGCATGAGTGGAATTCTCGGGTGCCAAGG | 19592 | 0.10897259621759633 | Illumina Small RNA Adapter 2 (100% over 21bp) |
| TGTGAAAGTAGGTAATCGTCAGGCTTGGAATTCTCGGGTGCCAAGGAACTC | 18892 | 0.10507912861080185 | RNA PCR Primer, Index 1 (100% over 26bp) |
| CGTGTGAAAGTAGGTAATCGTCAGGCTTGGAATTCTCGGGTGCCAAGGAAC | 18417 | 0.10243713273476275 | RNA PCR Primer, Index 1 (100% over 24bp) |
| AAACCGTTACCATTACTGAGTTGGAATTCTCGGGTGCCAAGGAACTCCAGT | 18199 | 0.10122459568007532 | RNA PCR Primer, Index 1 (100% over 30bp) |
| GGATTCCTGGAAATACTGTTCTTGGAATTCTCGGGTGCCAAGGAACTCCAG | 18149 | 0.10094649085101857 | RNA PCR Primer, Index 1 (100% over 29bp) |

## Adapter Content

Produced by FastQC (version 0.11.9)
