# Supplementary material for: High Throughput Sequencing of MicroRNA in Rainbow Trout Plasma, Mucus, and Surrounding Water Following Acute Stress
Source: Front Physiol. 2021 Jan 13;11:588313. doi: 10.3389/fphys.2020.588313 (PMC7838646; doi:10.3389/fphys.2020.588313)
Supplement: Supplementary file 2 [file Data_Sheet_1.ZIP › Supplemental Quality Control/FastQC_raw_files/plasma_stressed_2_fastqc_raw.html]

SV18263\_0022\_S12\_R1\_001.fastq FastQC Report 

FastQC Report

Thu 7 May 2020  
SV18263\_0022\_S12\_R1\_001.fastq

## Summary

- Basic Statistics
- Per base sequence quality
- Per tile sequence quality
- Per sequence quality scores
- Per base sequence content
- Per sequence GC content
- Per base N content
- Sequence Length Distribution
- Sequence Duplication Levels
- Overrepresented sequences
- Adapter Content

## Basic Statistics

| Measure | Value |
| --- | --- |
| Filename | SV18263\_0022\_S12\_R1\_001.fastq |
| File type | Conventional base calls |
| Encoding | Sanger / Illumina 1.9 |
| Total Sequences | 21978608 |
| Sequences flagged as poor quality | 0 |
| Sequence length | 51 |
| %GC | 51 |

## Per base sequence quality

## Per tile sequence quality

## Per sequence quality scores

## Per base sequence content

## Per sequence GC content

## Per base N content

## Sequence Length Distribution

## Sequence Duplication Levels

## Overrepresented sequences

| Sequence | Count | Percentage | Possible Source |
| --- | --- | --- | --- |
| AACCCGTAGATCCGAACTTGTGTGGAATTCTCGGGTGCCAAGGAACTCCAG | 3093180 | 14.073593741696472 | RNA PCR Primer, Index 1 (100% over 29bp) |
| GCATTGGTGGTTCAGTGGTAGAATTCTCGCCTGGAATTCTCGGGTGCCAAG | 2190510 | 9.966554751784098 | No Hit |
| TGAGAACTGAATTCCATAGATGGTGGAATTCTCGGGTGCCAAGGAACTCCA | 1095900 | 4.986212047641962 | RNA PCR Primer, Index 1 (100% over 28bp) |
| TTCAAGTAATCCAGGATAGGCTTGGAATTCTCGGGTGCCAAGGAACTCCAG | 968368 | 4.405956919564697 | RNA PCR Primer, Index 1 (100% over 29bp) |
| GCATTGGTGGTTCAGTGGTAGAATTCTCGCCTTGGAATTCTCGGGTGCCAA | 912138 | 4.150117241273878 | No Hit |
| AACCCGTAGATCCGAACTTGTTGGAATTCTCGGGTGCCAAGGAACTCCAGT | 722323 | 3.286482019243439 | RNA PCR Primer, Index 1 (100% over 30bp) |
| TGAGGTAGTAGATTGAATAGTTTGGAATTCTCGGGTGCCAAGGAACTCCAG | 683798 | 3.1111979430180474 | RNA PCR Primer, Index 1 (100% over 29bp) |
| TGAGGTAGTAGGTTGTATAGTTTGGAATTCTCGGGTGCCAAGGAACTCCAG | 575593 | 2.618878320228469 | RNA PCR Primer, Index 1 (100% over 29bp) |
| GCATTGGTGGTTCAGTGGTAGAATTCTCGCTGGAATTCTCGGGTGCCAAGG | 398771 | 1.814359671913708 | Illumina Small RNA Adapter 2 (100% over 21bp) |
| AACATTCAACGCTGTCGGTGAGTGGAATTCTCGGGTGCCAAGGAACTCCAG | 389329 | 1.7713997173979354 | RNA PCR Primer, Index 1 (100% over 29bp) |
| TAACGGAACCCATAATGCAGCTGTGGAATTCTCGGGTGCCAAGGAACTCCA | 326379 | 1.484984854363843 | RNA PCR Primer, Index 1 (100% over 28bp) |
| TCCCTGGTGGTCTAGTGGTTAGGATTCGGCGCTTGGAATTCTCGGGTGCCA | 281320 | 1.2799718708300363 | No Hit |
| AACCCGTAGATCCGAACTTGTGTTGGAATTCTCGGGTGCCAAGGAACTCCA | 256732 | 1.168099453796164 | RNA PCR Primer, Index 1 (100% over 28bp) |
| AACCCGTAGATCCGAACTTGTGATGGAATTCTCGGGTGCCAAGGAACTCCA | 240889 | 1.0960157258366863 | RNA PCR Primer, Index 1 (100% over 28bp) |
| GCATTGTGGTTCAGTGGTAGAATTCTCGCCTGGAATTCTCGGGTGCCAAGG | 204719 | 0.9314466139074868 | Illumina Small RNA Adapter 2 (100% over 21bp) |
| TGAGAACTGAATTCCATAGATGGTTGGAATTCTCGGGTGCCAAGGAACTCC | 190678 | 0.8675617673330358 | RNA PCR Primer, Index 1 (100% over 27bp) |
| TCCCTGGTCTAGTGGTTAGGATTCGGCGCTTGGAATTCTCGGGTGCCAAGG | 176523 | 0.803158234588833 | Illumina Small RNA Adapter 2 (100% over 21bp) |
| AACCCGTAGATCCGAACTTGTGCTGGAATTCTCGGGTGCCAAGGAACTCCA | 156093 | 0.7102042131148615 | RNA PCR Primer, Index 1 (100% over 28bp) |
| TACCCTGTAGAACCGAATTTGTTGGAATTCTCGGGTGCCAAGGAACTCCAG | 151389 | 0.6888015837945697 | RNA PCR Primer, Index 1 (100% over 29bp) |
| TGAGGTAGTAGGTTGTATAGTTGGAATTCTCGGGTGCCAAGGAACTCCAGT | 133381 | 0.6068673684884865 | RNA PCR Primer, Index 1 (100% over 30bp) |
| TGAGGTAGTAGATTGAATAGTTGGAATTCTCGGGTGCCAAGGAACTCCAGT | 103008 | 0.46867390327904296 | RNA PCR Primer, Index 1 (100% over 30bp) |
| TAGCTTATCAGACTGGTGTTGGTGGAATTCTCGGGTGCCAAGGAACTCCAG | 101545 | 0.462017430767226 | RNA PCR Primer, Index 1 (100% over 29bp) |
| GTTTCCGTAGTGTAGTGGTTATCACGTTCGCCTGGAATTCTCGGGTGCCAA | 94806 | 0.4313557983289934 | No Hit |
| GCATTGTGGTTCAGTGGTAGAATTCTCGCCTTGGAATTCTCGGGTGCCAAG | 92067 | 0.4188936806189 | No Hit |
| TAGCTTATCAGACTGGTGTTGGCTGGAATTCTCGGGTGCCAAGGAACTCCA | 88943 | 0.4046798596162232 | RNA PCR Primer, Index 1 (100% over 28bp) |
| TGAGAACTGAATTCCATAGATGTGGAATTCTCGGGTGCCAAGGAACTCCAG | 87850 | 0.3997068422167591 | RNA PCR Primer, Index 1 (100% over 29bp) |
| TAGCAGCACGTAAATATTGGAGTGGAATTCTCGGGTGCCAAGGAACTCCAG | 77279 | 0.35161007466896904 | RNA PCR Primer, Index 1 (100% over 29bp) |
| AACCCGTAGATCCGAACTTGTGGAATTCTCGGGTGCCAAGGAACTCCAGTC | 74125 | 0.3372597573058312 | RNA PCR Primer, Index 1 (100% over 31bp) |
| TAACGGAACCCATAAAGCAGCTGTGGAATTCTCGGGTGCCAAGGAACTCCA | 70274 | 0.3197381745013151 | RNA PCR Primer, Index 1 (100% over 28bp) |
| GTTTCCGTAGTGTAGTGGTTATCACGTTCGCCTTGGAATTCTCGGGTGCCA | 63251 | 0.28778437651738453 | No Hit |
| TATTGCACTTGTCCCGGCCTGTTGGAATTCTCGGGTGCCAAGGAACTCCAG | 63079 | 0.28700179738407455 | RNA PCR Primer, Index 1 (100% over 29bp) |
| ACCATCGACCGTTGATTGTACCTGGAATTCTCGGGTGCCAAGGAACTCCAG | 61486 | 0.2797538406435931 | RNA PCR Primer, Index 1 (100% over 29bp) |
| TCGTACCGTGAGTAATAATGCATGGAATTCTCGGGTGCCAAGGAACTCCAG | 60433 | 0.2749628183914104 | RNA PCR Primer, Index 1 (100% over 29bp) |
| TAACGGAACCCATAATGCAGCTTGGAATTCTCGGGTGCCAAGGAACTCCAG | 59017 | 0.2685201901776491 | RNA PCR Primer, Index 1 (100% over 29bp) |
| TTCAAGTAATCCAGGATAGGCTGGAATTCTCGGGTGCCAAGGAACTCCAGT | 51929 | 0.2362706500793863 | RNA PCR Primer, Index 1 (100% over 30bp) |
| AACATTCATTGCTGTCGGTGGGTGGAATTCTCGGGTGCCAAGGAACTCCAG | 49818 | 0.22666585618161078 | RNA PCR Primer, Index 1 (100% over 29bp) |
| GGATTCCTGGAAATACTGTTCTTGGAATTCTCGGGTGCCAAGGAACTCCAG | 47105 | 0.2143220353172503 | RNA PCR Primer, Index 1 (100% over 29bp) |
| TGAGGTAGTAGTTTGTATAGTTTGGAATTCTCGGGTGCCAAGGAACTCCAG | 42386 | 0.19285115781672799 | RNA PCR Primer, Index 1 (100% over 29bp) |
| TGAGGTAGTAGGTTGTATAGTTTTGGAATTCTCGGGTGCCAAGGAACTCCA | 41199 | 0.18745045182115264 | RNA PCR Primer, Index 1 (100% over 28bp) |
| GCATTGTGGTTCAGTGGTAGAATTCTCGCTGGAATTCTCGGGTGCCAAGGA | 40648 | 0.18494346866735145 | RNA PCR Primer, Index 1 (100% over 22bp) |
| TCCCTGAGACCCTAACTTGTGTGGAATTCTCGGGTGCCAAGGAACTCCAGT | 38947 | 0.1772041250292102 | RNA PCR Primer, Index 1 (100% over 30bp) |
| GCCCGGCTAGCTCAGTCGGTAGAGCATGATGGAATTCTCGGGTGCCAAGGA | 35269 | 0.16046967123668615 | RNA PCR Primer, Index 1 (100% over 22bp) |
| TCCATAAAGTAGAAAGCACTATGGAATTCTCGGGTGCCAAGGAACTCCAGT | 34260 | 0.155878843646513 | RNA PCR Primer, Index 1 (100% over 30bp) |
| GCATTGGTGGTTCAGTGGTAGAATTCTCGGGTGCCAAGGAACTCCAGTCAC | 32373 | 0.1472932225735133 | RNA PCR Primer, Index 1 (96% over 33bp) |
| GCATTGGTGGTTCAGTGGTAGAATTCTCGCCTGTGGAATTCTCGGGTGCCA | 32269 | 0.14682003519058168 | No Hit |
| TTCACAGTGGTTAAGTTCTGCTGGAATTCTCGGGTGCCAAGGAACTCCAGT | 29628 | 0.13480380559132774 | RNA PCR Primer, Index 1 (100% over 30bp) |
| TATTGCACTTGTCCCGGCCTGTATTGGAATTCTCGGGTGCCAAGGAACTCC | 27388 | 0.1246120773435697 | RNA PCR Primer, Index 1 (100% over 27bp) |
| AACATTCAACGCTGTCGGTGATGGAATTCTCGGGTGCCAAGGAACTCCAGT | 25441 | 0.11575346354964793 | RNA PCR Primer, Index 1 (100% over 30bp) |
| CACGTTGGGCGCCATGGAATTCTCGGGTGCCAAGGAACTCCAGTCACCTTG | 25333 | 0.11526207665198815 | RNA PCR Primer, Index 12 (100% over 37bp) |
| GCCCGGATAGCTCAGTGGAATTCTCGGGTGCCAAGGAACTCCAGTCACCTT | 25201 | 0.11466149266595957 | RNA PCR Primer, Index 12 (100% over 36bp) |
| CCCTGAGACCCTTAACCTGTGATGGAATTCTCGGGTGCCAAGGAACTCCAG | 24954 | 0.11353767263149696 | RNA PCR Primer, Index 1 (100% over 29bp) |
| GACCATCGACCGTTGATTGTACCTGGAATTCTCGGGTGCCAAGGAACTCCA | 24839 | 0.11301443658306295 | RNA PCR Primer, Index 1 (100% over 28bp) |
| TGAGATGAAGCACTGTAGCTTGGAATTCTCGGGTGCCAAGGAACTCCAGTC | 24382 | 0.11093514202537304 | RNA PCR Primer, Index 1 (100% over 31bp) |
| ATCACATTGCCAGGGATTTCCTGGAATTCTCGGGTGCCAAGGAACTCCAGT | 23403 | 0.10648081079566094 | RNA PCR Primer, Index 1 (100% over 30bp) |
| AAGCTGCCAGCTGAAGAACTGTTGGAATTCTCGGGTGCCAAGGAACTCCAG | 23391 | 0.10642621225147653 | RNA PCR Primer, Index 1 (100% over 29bp) |
| GCCCGGCTAGCTCAGTCGGTAGAGCATGAGATGGAATTCTCGGGTGCCAAG | 23359 | 0.10628061613365142 | No Hit |
| TAACGGAACCCATAAAGCAGCTTGGAATTCTCGGGTGCCAAGGAACTCCAG | 23348 | 0.10623056746814903 | RNA PCR Primer, Index 1 (100% over 29bp) |
| TCCCTGAGACCCTTAACCTGTTGGAATTCTCGGGTGCCAAGGAACTCCAGT | 22479 | 0.10227672289346078 | RNA PCR Primer, Index 1 (100% over 30bp) |
| TCCCTGAGACCCTTAACCTGTGTGGAATTCTCGGGTGCCAAGGAACTCCAG | 22401 | 0.10192183235626207 | RNA PCR Primer, Index 1 (100% over 29bp) |
| AACATTCAACGCTGTCGGTGAGTTGGAATTCTCGGGTGCCAAGGAACTCCA | 22030 | 0.10023382736522714 | RNA PCR Primer, Index 1 (100% over 28bp) |

## Adapter Content

Produced by FastQC (version 0.11.9)
