# Supplementary material for: High Throughput Sequencing of MicroRNA in Rainbow Trout Plasma, Mucus, and Surrounding Water Following Acute Stress
Source: Front Physiol. 2021 Jan 13;11:588313. doi: 10.3389/fphys.2020.588313 (PMC7838646; doi:10.3389/fphys.2020.588313)
Supplement: Supplementary file 2 [file Data_Sheet_1.ZIP › Supplemental Quality Control/FastQC_raw_files/plasma_stressed_3_fastqc_raw.html]

SV18263\_0024\_S14\_R1\_001.fastq FastQC Report 

FastQC Report

Thu 7 May 2020  
SV18263\_0024\_S14\_R1\_001.fastq

## Summary

- Basic Statistics
- Per base sequence quality
- Per tile sequence quality
- Per sequence quality scores
- Per base sequence content
- Per sequence GC content
- Per base N content
- Sequence Length Distribution
- Sequence Duplication Levels
- Overrepresented sequences
- Adapter Content

## Basic Statistics

| Measure | Value |
| --- | --- |
| Filename | SV18263\_0024\_S14\_R1\_001.fastq |
| File type | Conventional base calls |
| Encoding | Sanger / Illumina 1.9 |
| Total Sequences | 18746525 |
| Sequences flagged as poor quality | 0 |
| Sequence length | 51 |
| %GC | 52 |

## Per base sequence quality

## Per tile sequence quality

## Per sequence quality scores

## Per base sequence content

## Per sequence GC content

## Per base N content

## Sequence Length Distribution

## Sequence Duplication Levels

## Overrepresented sequences

| Sequence | Count | Percentage | Possible Source |
| --- | --- | --- | --- |
| GCATTGGTGGTTCAGTGGTAGAATTCTCGCCTGGAATTCTCGGGTGCCAAG | 2329344 | 12.425470853931596 | No Hit |
| GCATTGGTGGTTCAGTGGTAGAATTCTCGCCTTGGAATTCTCGGGTGCCAA | 1379508 | 7.358739819779933 | No Hit |
| AACCCGTAGATCCGAACTTGTGTGGAATTCTCGGGTGCCAAGGAACTCCAG | 701905 | 3.7441872560381193 | RNA PCR Primer, Index 1 (100% over 29bp) |
| GCATTGGTGGTTCAGTGGTAGAATTCTCGCTGGAATTCTCGGGTGCCAAGG | 660845 | 3.525159996319318 | Illumina Small RNA Adapter 2 (100% over 21bp) |
| TGAGAACTGAATTCCATAGATGGTGGAATTCTCGGGTGCCAAGGAACTCCA | 534541 | 2.8514137953567396 | RNA PCR Primer, Index 1 (100% over 28bp) |
| TGAGGTAGTAGGTTGTATAGTTTGGAATTCTCGGGTGCCAAGGAACTCCAG | 393046 | 2.0966339094845576 | RNA PCR Primer, Index 1 (100% over 29bp) |
| TTCAAGTAATCCAGGATAGGCTTGGAATTCTCGGGTGCCAAGGAACTCCAG | 363378 | 1.9383752455455077 | RNA PCR Primer, Index 1 (100% over 29bp) |
| TGAGGTAGTAGATTGAATAGTTTGGAATTCTCGGGTGCCAAGGAACTCCAG | 306516 | 1.6350550301989304 | RNA PCR Primer, Index 1 (100% over 29bp) |
| TCCCTGGTGGTCTAGTGGTTAGGATTCGGCGCTTGGAATTCTCGGGTGCCA | 239792 | 1.2791277316729366 | No Hit |
| TAACGGAACCCATAATGCAGCTGTGGAATTCTCGGGTGCCAAGGAACTCCA | 230623 | 1.2302173336124962 | RNA PCR Primer, Index 1 (100% over 28bp) |
| GCATTGTGGTTCAGTGGTAGAATTCTCGCCTGGAATTCTCGGGTGCCAAGG | 177164 | 0.9450498158992133 | Illumina Small RNA Adapter 2 (100% over 21bp) |
| GTTTCCGTAGTGTAGTGGTTATCACGTTCGCCTGGAATTCTCGGGTGCCAA | 151610 | 0.8087365525077315 | No Hit |
| AACCCGTAGATCCGAACTTGTTGGAATTCTCGGGTGCCAAGGAACTCCAGT | 149472 | 0.7973317721551061 | RNA PCR Primer, Index 1 (100% over 30bp) |
| TCCCTGGTCTAGTGGTTAGGATTCGGCGCTTGGAATTCTCGGGTGCCAAGG | 134814 | 0.7191412808507176 | Illumina Small RNA Adapter 2 (100% over 21bp) |
| AACATTCAACGCTGTCGGTGAGTGGAATTCTCGGGTGCCAAGGAACTCCAG | 112534 | 0.600292587559561 | RNA PCR Primer, Index 1 (100% over 29bp) |
| GCATTGTGGTTCAGTGGTAGAATTCTCGCCTTGGAATTCTCGGGTGCCAAG | 108398 | 0.5782298319288508 | No Hit |
| TGAGGTAGTAGGTTGTATAGTTGGAATTCTCGGGTGCCAAGGAACTCCAGT | 103917 | 0.5543267352215944 | RNA PCR Primer, Index 1 (100% over 30bp) |
| TGAGAACTGAATTCCATAGATGGTTGGAATTCTCGGGTGCCAAGGAACTCC | 102568 | 0.5471307348962008 | RNA PCR Primer, Index 1 (100% over 27bp) |
| TACCCTGTAGAACCGAATTTGTTGGAATTCTCGGGTGCCAAGGAACTCCAG | 97363 | 0.5193655890891778 | RNA PCR Primer, Index 1 (100% over 29bp) |
| GTTTCCGTAGTGTAGTGGTTATCACGTTCGCCTTGGAATTCTCGGGTGCCA | 80001 | 0.42675109120223614 | No Hit |
| TAGCTTATCAGACTGGTGTTGGTGGAATTCTCGGGTGCCAAGGAACTCCAG | 74297 | 0.39632411873667256 | RNA PCR Primer, Index 1 (100% over 29bp) |
| TAGCTTATCAGACTGGTGTTGGCTGGAATTCTCGGGTGCCAAGGAACTCCA | 58633 | 0.31276729953951465 | RNA PCR Primer, Index 1 (100% over 28bp) |
| AACCCGTAGATCCGAACTTGTGATGGAATTCTCGGGTGCCAAGGAACTCCA | 56348 | 0.3005783738586218 | RNA PCR Primer, Index 1 (100% over 28bp) |
| AACCCGTAGATCCGAACTTGTGTTGGAATTCTCGGGTGCCAAGGAACTCCA | 53611 | 0.28597833465135536 | RNA PCR Primer, Index 1 (100% over 28bp) |
| GCCCGGCTAGCTCAGTCGGTAGAGCATGATGGAATTCTCGGGTGCCAAGGA | 52833 | 0.28182823216569475 | RNA PCR Primer, Index 1 (100% over 22bp) |
| GCATTGTGGTTCAGTGGTAGAATTCTCGCTGGAATTCTCGGGTGCCAAGGA | 51172 | 0.272967923388468 | RNA PCR Primer, Index 1 (100% over 22bp) |
| TGAGGTAGTAGATTGAATAGTTGGAATTCTCGGGTGCCAAGGAACTCCAGT | 47944 | 0.2557487320983489 | RNA PCR Primer, Index 1 (100% over 30bp) |
| TAACGGAACCCATAAAGCAGCTGTGGAATTCTCGGGTGCCAAGGAACTCCA | 47816 | 0.25506593888734047 | RNA PCR Primer, Index 1 (100% over 28bp) |
| TGAGAACTGAATTCCATAGATGTGGAATTCTCGGGTGCCAAGGAACTCCAG | 47347 | 0.2525641418876299 | RNA PCR Primer, Index 1 (100% over 29bp) |
| TGAGGTAGTAGGTTGTATAGTTTTGGAATTCTCGGGTGCCAAGGAACTCCA | 43498 | 0.2320323366597276 | RNA PCR Primer, Index 1 (100% over 28bp) |
| GCATTGGTGGTTCAGTGGTAGAATTCTCGCCTGTGGAATTCTCGGGTGCCA | 43419 | 0.23161092522480833 | No Hit |
| GAGCCGCGGCTGGGGGAGCATGGAATTCTCGGGTGCCAAGGAACTCCAGTC | 42678 | 0.22765819265170478 | RNA PCR Primer, Index 1 (100% over 31bp) |
| TAGCAGCACGTAAATATTGGAGTGGAATTCTCGGGTGCCAAGGAACTCCAG | 36160 | 0.19288908210988437 | RNA PCR Primer, Index 1 (100% over 29bp) |
| CGAGCCGCGGCTGGGGGAGCATGGAATTCTCGGGTGCCAAGGAACTCCAGT | 36028 | 0.1921849516110319 | RNA PCR Primer, Index 1 (100% over 30bp) |
| TAACGGAACCCATAATGCAGCTTGGAATTCTCGGGTGCCAAGGAACTCCAG | 35579 | 0.18978984105054136 | RNA PCR Primer, Index 1 (100% over 29bp) |
| TATTGCACTTGTCCCGGCCTGTTGGAATTCTCGGGTGCCAAGGAACTCCAG | 34284 | 0.18288189411104191 | RNA PCR Primer, Index 1 (100% over 29bp) |
| CCGTGTGAAAGTAGGTAATCGTCAGGCTTGGAATTCTCGGGTGCCAAGGAA | 33193 | 0.17706214885158716 | RNA PCR Primer, Index 1 (100% over 23bp) |
| TCGTACCGTGAGTAATAATGCATGGAATTCTCGGGTGCCAAGGAACTCCAG | 33109 | 0.17661406580686287 | RNA PCR Primer, Index 1 (100% over 29bp) |
| AAGCTGCCAGCTGAAGAACTGTTGGAATTCTCGGGTGCCAAGGAACTCCAG | 32262 | 0.17209589510589296 | RNA PCR Primer, Index 1 (100% over 29bp) |
| GCCCGGCTAGCTCAGTCGGTAGAGCATGAGATGGAATTCTCGGGTGCCAAG | 31604 | 0.1685859112555527 | No Hit |
| AACCCGTAGATCCGAACTTGTGCTGGAATTCTCGGGTGCCAAGGAACTCCA | 31490 | 0.1679777985519983 | RNA PCR Primer, Index 1 (100% over 28bp) |
| GCATTGGTGGTTCAGTGGTAGAATTCTCGTGGAATTCTCGGGTGCCAAGGA | 30406 | 0.16219539354627058 | RNA PCR Primer, Index 1 (100% over 22bp) |
| CCCGTGTGAAAGTAGGTAATCGTCAGGCTTGGAATTCTCGGGTGCCAAGGA | 28860 | 0.15394853179455925 | RNA PCR Primer, Index 1 (100% over 22bp) |
| GCATTGGTGGTTCAGTGGTAGAATTCTCTGGAATTCTCGGGTGCCAAGGAA | 28742 | 0.15331908180316087 | RNA PCR Primer, Index 1 (100% over 23bp) |
| AAAGTAGGTAATCGTCAGGCTTGGAATTCTCGGGTGCCAAGGAACTCCAGT | 27206 | 0.14512556327105958 | RNA PCR Primer, Index 1 (100% over 30bp) |
| GGTTGGCAGCGGCGACTCTGGACGCTGGAATTCTCGGGTGCCAAGGAACTC | 26325 | 0.14042602562341555 | RNA PCR Primer, Index 1 (100% over 26bp) |
| TGAAAGTAGGTAATCGTCAGGCTTGGAATTCTCGGGTGCCAAGGAACTCCA | 26054 | 0.1389804243719836 | RNA PCR Primer, Index 1 (100% over 28bp) |
| TGAGGTAGTAGTTTGTATAGTTTGGAATTCTCGGGTGCCAAGGAACTCCAG | 25696 | 0.13707073710994438 | RNA PCR Primer, Index 1 (100% over 29bp) |
| GAAAGTAGGTAATCGTCAGGCTTGGAATTCTCGGGTGCCAAGGAACTCCAG | 24724 | 0.13188577616384903 | RNA PCR Primer, Index 1 (100% over 29bp) |
| ACCATCGACCGTTGATTGTACCTGGAATTCTCGGGTGCCAAGGAACTCCAG | 23863 | 0.12729292495542507 | RNA PCR Primer, Index 1 (100% over 29bp) |
| GTAGGTAATCGTCAGGCTTGGAATTCTCGGGTGCCAAGGAACTCCAGTCAC | 23728 | 0.1265727914906896 | RNA PCR Primer, Index 1 (100% over 33bp) |
| GCCCGGCTAGCTCAGTCGGTAGAGCATGAGACTCTTAATCTTGGAATTCTC | 23084 | 0.1231374881478034 | No Hit |
| AAGTAGGTAATCGTCAGGCTTGGAATTCTCGGGTGCCAAGGAACTCCAGTC | 22969 | 0.122524041122288 | RNA PCR Primer, Index 1 (100% over 31bp) |
| AACATTCATTGCTGTCGGTGGGTGGAATTCTCGGGTGCCAAGGAACTCCAG | 22754 | 0.12137716190067226 | RNA PCR Primer, Index 1 (100% over 29bp) |
| GTGAAAGTAGGTAATCGTCAGGCTTGGAATTCTCGGGTGCCAAGGAACTCC | 21730 | 0.11591481621260474 | RNA PCR Primer, Index 1 (100% over 27bp) |
| GCATTGGTGGTTCAGTGGTAGAATTCTCGGGTGCCAAGGAACTCCAGTCAC | 20110 | 0.10727321463577916 | RNA PCR Primer, Index 1 (96% over 33bp) |
| GGAATACCAGGTGCTGTAAGCTTTGGAATTCTCGGGTGCCAAGGAACTCCA | 20029 | 0.10684113455693789 | RNA PCR Primer, Index 1 (100% over 28bp) |
| AGTAGGTAATCGTCAGGCTTGGAATTCTCGGGTGCCAAGGAACTCCAGTCA | 19848 | 0.10587562228199626 | RNA PCR Primer, Index 1 (100% over 32bp) |
| TCCCTGTGGTCTAGTGGTTAGGATTCGGCGCTTGGAATTCTCGGGTGCCAA | 19630 | 0.1047127400944975 | No Hit |
| TCCCTGGTGGTCTAGTGGTTAGGATTCGGCGCTGGAATTCTCGGGTGCCAA | 19453 | 0.1037685651073999 | No Hit |
| GTGTGAAAGTAGGTAATCGTCAGGCTTGGAATTCTCGGGTGCCAAGGAACT | 19149 | 0.10214693123125486 | RNA PCR Primer, Index 1 (100% over 25bp) |
| GAGCCGCGGCTGGGGGAGCTGGAATTCTCGGGTGCCAAGGAACTCCAGTCA | 18928 | 0.10096804607787309 | RNA PCR Primer, Index 1 (100% over 32bp) |

## Adapter Content

Produced by FastQC (version 0.11.9)
