# Supplementary material for: High Throughput Sequencing of MicroRNA in Rainbow Trout Plasma, Mucus, and Surrounding Water Following Acute Stress
Source: Front Physiol. 2021 Jan 13;11:588313. doi: 10.3389/fphys.2020.588313 (PMC7838646; doi:10.3389/fphys.2020.588313)
Supplement: Supplementary file 2 [file Data_Sheet_1.ZIP › Supplemental Quality Control/FastQC_raw_files/water_control_1_fastqc_raw.html]

SV18263\_0013\_S25\_R1\_001.fastq FastQC Report 

FastQC Report

Thu 7 May 2020  
SV18263\_0013\_S25\_R1\_001.fastq

## Summary

- Basic Statistics
- Per base sequence quality
- Per tile sequence quality
- Per sequence quality scores
- Per base sequence content
- Per sequence GC content
- Per base N content
- Sequence Length Distribution
- Sequence Duplication Levels
- Overrepresented sequences
- Adapter Content

## Basic Statistics

| Measure | Value |
| --- | --- |
| Filename | SV18263\_0013\_S25\_R1\_001.fastq |
| File type | Conventional base calls |
| Encoding | Sanger / Illumina 1.9 |
| Total Sequences | 13307688 |
| Sequences flagged as poor quality | 0 |
| Sequence length | 51 |
| %GC | 52 |

## Per base sequence quality

## Per tile sequence quality

## Per sequence quality scores

## Per base sequence content

## Per sequence GC content

## Per base N content

## Sequence Length Distribution

## Sequence Duplication Levels

## Overrepresented sequences

| Sequence | Count | Percentage | Possible Source |
| --- | --- | --- | --- |
| TGAGAACTGAATTCCATAGATGGTGGAATTCTCGGGTGCCAAGGAACTCCA | 214213 | 1.609693584640698 | RNA PCR Primer, Index 1 (100% over 28bp) |
| CTTCGGAGTCTGTGGTAGGAAACCTGGAATTCTCGGGTGCCAAGGAACTCC | 163969 | 1.2321373930618151 | RNA PCR Primer, Index 1 (100% over 27bp) |
| TCTTTTGGCAGGTGAGTAGAGCCGTTCGTGACTGGAATTCTCGGGTGCCAA | 138296 | 1.0392188335043624 | No Hit |
| TCTTTTGGCAGGTGAGTAGAGCCGTTCGTGATGGAATTCTCGGGTGCCAAG | 108920 | 0.818474253378949 | No Hit |
| TTTTGGCAGGTGAGTAGAGCCGTTCGTGACTGGAATTCTCGGGTGCCAAGG | 94403 | 0.7093869348304529 | Illumina Small RNA Adapter 2 (100% over 21bp) |
| GAGGTGTAGAATAAGTGGGAGGCCCTGGAATTCTCGGGTGCCAAGGAACTC | 90452 | 0.6796973298442224 | RNA PCR Primer, Index 1 (100% over 26bp) |
| TTTTGGCAGGTGAGTAGAGCCGTTCGTGATGGAATTCTCGGGTGCCAAGGA | 88793 | 0.6672308518203913 | RNA PCR Primer, Index 1 (100% over 22bp) |
| AGGTGAGTAGAGCCGTTCGTGACATGGAATTCTCGGGTGCCAAGGAACTCC | 81887 | 0.6153360373342086 | RNA PCR Primer, Index 1 (100% over 27bp) |
| AGGTGAGTAGAGCCGTTCGTGACTGGAATTCTCGGGTGCCAAGGAACTCCA | 81277 | 0.6107522208215281 | RNA PCR Primer, Index 1 (100% over 28bp) |
| CCGAGAAGACGATCAAACTTGATGGAATTCTCGGGTGCCAAGGAACTCCAG | 80966 | 0.608415225845391 | RNA PCR Primer, Index 1 (100% over 29bp) |
| TTTGGCAGGTGAGTAGAGCCGTTCGTGACTGGAATTCTCGGGTGCCAAGGA | 75920 | 0.570497294496234 | RNA PCR Primer, Index 1 (100% over 22bp) |
| TTGGCAGGTGAGTAGAGCCGTTCGTGATGGAATTCTCGGGTGCCAAGGAAC | 74407 | 0.5591279266541266 | RNA PCR Primer, Index 1 (100% over 24bp) |
| AGGTGTAGAATAAGTGGGAGGCCCTGGAATTCTCGGGTGCCAAGGAACTCC | 63296 | 0.4756348360436463 | RNA PCR Primer, Index 1 (100% over 27bp) |
| TTTGGCAGGTGAGTAGAGCCGTTCGTGATGGAATTCTCGGGTGCCAAGGAA | 60761 | 0.45658569692947415 | RNA PCR Primer, Index 1 (100% over 23bp) |
| GGAATACCAGGTGCTGTAAGCTTTGGAATTCTCGGGTGCCAAGGAACTCCA | 60140 | 0.4519192214304994 | RNA PCR Primer, Index 1 (100% over 28bp) |
| GCCGAGAAGACGATCAAACTTGATGGAATTCTCGGGTGCCAAGGAACTCCA | 57552 | 0.432471816291455 | RNA PCR Primer, Index 1 (100% over 28bp) |
| CTTTTGGCAGGTGAGTAGAGCCGTTCGTGACATGGAATTCTCGGGTGCCAA | 54666 | 0.41078510406916663 | No Hit |
| CTTTTGGCAGGTGAGTAGAGCCGTTCGTGACTGGAATTCTCGGGTGCCAAG | 51007 | 0.3832897194463832 | No Hit |
| AGGTGTAGAATAAGTGGGAGGCCCCGTGGAATTCTCGGGTGCCAAGGAACT | 49737 | 0.3737463637560484 | RNA PCR Primer, Index 1 (100% over 25bp) |
| GAGGTGTAGAATAAGTGGGAGGCCCCGTGGAATTCTCGGGTGCCAAGGAAC | 48150 | 0.36182092636977964 | RNA PCR Primer, Index 1 (100% over 24bp) |
| TCTTTTGGCAGGTGAGTAGAGCCGTTCGTGACATGGAATTCTCGGGTGCCA | 46041 | 0.3459729443611843 | No Hit |
| TTGGCAGGTGAGTAGAGCCGTTCGTGACATGGAATTCTCGGGTGCCAAGGA | 44147 | 0.33174056981197636 | RNA PCR Primer, Index 1 (100% over 22bp) |
| TTTTGGCAGGTGAGTAGAGCCGTTCGTGACATGGAATTCTCGGGTGCCAAG | 42619 | 0.3202584851703767 | No Hit |
| CTTTTGGCAGGTGAGTAGAGCCGTTCGTGATGGAATTCTCGGGTGCCAAGG | 38831 | 0.2917937360719608 | Illumina Small RNA Adapter 2 (100% over 21bp) |
| CGTCTGGCGGGCACGGGAAATGTGGTGTATATGGAATTCTCGGGTGCCAAG | 37140 | 0.2790867955425465 | No Hit |
| TTTGGCAGGTGAGTAGAGCCGTTCGTGACATGGAATTCTCGGGTGCCAAGG | 36452 | 0.2739168516725069 | Illumina Small RNA Adapter 2 (100% over 21bp) |
| CTCCGGGGATGCGTGCATTTATCAGATCTGGAATTCTCGGGTGCCAAGGAA | 36283 | 0.2726469090648954 | RNA PCR Primer, Index 1 (100% over 23bp) |
| CAGGTGAGTAGAGCCGTTCGTGACATGGAATTCTCGGGTGCCAAGGAACTC | 35762 | 0.2687318788958683 | RNA PCR Primer, Index 1 (100% over 26bp) |
| CTTCGGAGTCTGTGGTAGGAAACCTTGGAATTCTCGGGTGCCAAGGAACTC | 34459 | 0.2589405462466508 | RNA PCR Primer, Index 1 (100% over 26bp) |
| AGGTGAGTAGAGCCGTTCGTGATGGAATTCTCGGGTGCCAAGGAACTCCAG | 34335 | 0.2580087540375157 | RNA PCR Primer, Index 1 (100% over 29bp) |
| TAGCTTATCAGACTGGTGTTGGTGGAATTCTCGGGTGCCAAGGAACTCCAG | 29370 | 0.22069949340561634 | RNA PCR Primer, Index 1 (100% over 29bp) |
| CAGGTGAGTAGAGCCGTTCGTGACTGGAATTCTCGGGTGCCAAGGAACTCC | 27424 | 0.20607636728483567 | RNA PCR Primer, Index 1 (100% over 27bp) |
| TTGGCAGGTGAGTAGAGCCGTTCGTGACTGGAATTCTCGGGTGCCAAGGAA | 26256 | 0.19729948583104745 | RNA PCR Primer, Index 1 (100% over 23bp) |
| GGTGAGTAGAGCCGTTCGTGACATGGAATTCTCGGGTGCCAAGGAACTCCA | 25126 | 0.1888081536026393 | RNA PCR Primer, Index 1 (100% over 28bp) |
| ATCGGGGGCCTGAGTCCTGGAATTCTCGGGTGCCAAGGAACTCCAGTCACC | 25038 | 0.1881468817122854 | RNA PCR Primer, Index 2 (100% over 34bp) |
| CCGAGAAGACGATCAAACTTGTGGAATTCTCGGGTGCCAAGGAACTCCAGT | 24696 | 0.18557693868386455 | RNA PCR Primer, Index 1 (100% over 30bp) |
| TGTCAACCGGGTCGGACTGTCCTCAGTGCGTACTGGAATTCTCGGGTGCCA | 23600 | 0.17734109786763863 | No Hit |
| GAATACCAGGTGCTGTAAGCTTTGGAATTCTCGGGTGCCAAGGAACTCCAG | 23469 | 0.17635670448540722 | RNA PCR Primer, Index 1 (100% over 29bp) |
| GGTGAGTAGAGCCGTTCGTGACTGGAATTCTCGGGTGCCAAGGAACTCCAG | 22386 | 0.1682185515620745 | RNA PCR Primer, Index 1 (100% over 29bp) |
| ACGGGAAATGTGGTGTATATGGAATTCTCGGGTGCCAAGGAACTCCAGTCA | 22105 | 0.1661069901849217 | RNA PCR Primer, Index 1 (100% over 32bp) |
| ATCGACGATGAAATACCACTACTCCTGTGGAATTCTCGGGTGCCAAGGAAC | 21669 | 0.16283068854635005 | RNA PCR Primer, Index 1 (100% over 24bp) |
| CCGAGAAGACGATCAAACTTGGAATTCTCGGGTGCCAAGGAACTCCAGTCA | 21064 | 0.15828444430016694 | RNA PCR Primer, Index 1 (100% over 32bp) |
| ACGGGAAATGTGGTGTATAGAAGACTGGAATTCTCGGGTGCCAAGGAACTC | 20652 | 0.15518848954078274 | RNA PCR Primer, Index 1 (100% over 26bp) |
| CAGGTGAGTAGAGCCGTTCGTGATGGAATTCTCGGGTGCCAAGGAACTCCA | 19906 | 0.1495827073793735 | RNA PCR Primer, Index 1 (100% over 28bp) |
| GTCTGGCGGGCACGGGAAATGTGGTGTATATGGAATTCTCGGGTGCCAAGG | 18658 | 0.14020466966162717 | Illumina Small RNA Adapter 2 (100% over 21bp) |
| AGGTGTAGAATAAGTGGGAGGCCCCGGTGGAATTCTCGGGTGCCAAGGAAC | 17501 | 0.1315104471941332 | RNA PCR Primer, Index 1 (100% over 24bp) |
| CGAGAAGACGATCAAACTTGATGGAATTCTCGGGTGCCAAGGAACTCCAGT | 16145 | 0.12132084852004345 | RNA PCR Primer, Index 1 (100% over 30bp) |
| GTGTGGTCGGATCCCTGGAATTCTCGGGTGCCAAGGAACTCCAGTCACCTA | 15986 | 0.12012605044542674 | RNA PCR Primer, Index 2 (97% over 36bp) |
| TAGCTTATCAGACTGGTGTTGGCTGGAATTCTCGGGTGCCAAGGAACTCCA | 15885 | 0.11936709066217963 | RNA PCR Primer, Index 1 (100% over 28bp) |
| ACGGGAAATGTGGTGTATAGATGGAATTCTCGGGTGCCAAGGAACTCCAGT | 15668 | 0.1177364542961933 | RNA PCR Primer, Index 1 (100% over 30bp) |
| ACGGGAAATGTGGTGTATAGAAGATGGAATTCTCGGGTGCCAAGGAACTCC | 15385 | 0.11560986401244153 | RNA PCR Primer, Index 1 (100% over 27bp) |
| GAGGTGTAGAATAAGTGGGAGGCCCCGGTGGAATTCTCGGGTGCCAAGGAA | 15383 | 0.11559483510584258 | RNA PCR Primer, Index 1 (100% over 23bp) |
| GCGCGTGTCGGCTGAGGTGGGATCCCGATGGAATTCTCGGGTGCCAAGGAA | 14791 | 0.11114627875255267 | RNA PCR Primer, Index 1 (100% over 23bp) |
| TGGCACTGTGAAGAGACATGAGTGGAATTCTCGGGTGCCAAGGAACTCCAG | 14602 | 0.10972604707895166 | RNA PCR Primer, Index 1 (100% over 29bp) |
| GTGAGTAGAGCCGTTCGTGATGGAATTCTCGGGTGCCAAGGAACTCCAGTC | 14435 | 0.10847113337793912 | RNA PCR Primer, Index 1 (100% over 31bp) |
| TGAGAACTGAATTCCATAGATGGTTGGAATTCTCGGGTGCCAAGGAACTCC | 14395 | 0.10817055524596007 | RNA PCR Primer, Index 1 (100% over 27bp) |
| CGCGTGTCGGCTGAGGTGGGATCCCGTGGAATTCTCGGGTGCCAAGGAACT | 14160 | 0.10640465872058316 | RNA PCR Primer, Index 1 (100% over 25bp) |
| TGTCAACCGGGTCGGACTGTCCTCAGTGCGTATGGAATTCTCGGGTGCCAA | 13729 | 0.10316592934850892 | No Hit |
| TAACGGAACCCATAATGCAGCTGTGGAATTCTCGGGTGCCAAGGAACTCCA | 13703 | 0.10297055356272254 | RNA PCR Primer, Index 1 (100% over 28bp) |
| TGAGAACTGAATTCCATAGATGTGGAATTCTCGGGTGCCAAGGAACTCCAG | 13599 | 0.10218905041957702 | RNA PCR Primer, Index 1 (100% over 29bp) |

## Adapter Content

Produced by FastQC (version 0.11.9)
