# Supplementary material for: High Throughput Sequencing of MicroRNA in Rainbow Trout Plasma, Mucus, and Surrounding Water Following Acute Stress
Source: Front Physiol. 2021 Jan 13;11:588313. doi: 10.3389/fphys.2020.588313 (PMC7838646; doi:10.3389/fphys.2020.588313)
Supplement: Supplementary file 2 [file Data_Sheet_1.ZIP › Supplemental Quality Control/FastQC_raw_files/water_control_2_fastqc_raw.html]

SV18263\_0015\_S27\_R1\_001.fastq FastQC Report 

FastQC Report

Thu 7 May 2020  
SV18263\_0015\_S27\_R1\_001.fastq

## Summary

- Basic Statistics
- Per base sequence quality
- Per tile sequence quality
- Per sequence quality scores
- Per base sequence content
- Per sequence GC content
- Per base N content
- Sequence Length Distribution
- Sequence Duplication Levels
- Overrepresented sequences
- Adapter Content

## Basic Statistics

| Measure | Value |
| --- | --- |
| Filename | SV18263\_0015\_S27\_R1\_001.fastq |
| File type | Conventional base calls |
| Encoding | Sanger / Illumina 1.9 |
| Total Sequences | 24700077 |
| Sequences flagged as poor quality | 0 |
| Sequence length | 51 |
| %GC | 52 |

## Per base sequence quality

## Per tile sequence quality

## Per sequence quality scores

## Per base sequence content

## Per sequence GC content

## Per base N content

## Sequence Length Distribution

## Sequence Duplication Levels

## Overrepresented sequences

| Sequence | Count | Percentage | Possible Source |
| --- | --- | --- | --- |
| AGAATAGTGGAAGGCTCTGGAAAGTGCTGGAATTCTCGGGTGCCAAGGAAC | 404013 | 1.63567506287531 | RNA PCR Primer, Index 1 (100% over 24bp) |
| GAGAATAGTGGAAGGCTCTGGAAAGTGCTGGAATTCTCGGGTGCCAAGGAA | 376150 | 1.5228697465194136 | RNA PCR Primer, Index 1 (100% over 23bp) |
| ATCAAGGCCGAGAACTGATGACGAGTTTGGAATTCTCGGGTGCCAAGGAAC | 275308 | 1.1146038127735391 | RNA PCR Primer, Index 1 (100% over 24bp) |
| AGATTAGCGGAACGCTCTGGAAAGTGCTGGAATTCTCGGGTGCCAAGGAAC | 230048 | 0.9313655176054715 | RNA PCR Primer, Index 1 (100% over 24bp) |
| ATTTGGAATTGTACAGTCAAGGTGTTGGAATTCTCGGGTGCCAAGGAACTC | 219840 | 0.8900377112184711 | RNA PCR Primer, Index 1 (100% over 26bp) |
| TCAAGGCCGAGAACTGATGACGAGTTTGGAATTCTCGGGTGCCAAGGAACT | 211138 | 0.8548070518160733 | RNA PCR Primer, Index 1 (100% over 25bp) |
| GAGATTAGCGGAACGCTCTGGAAAGTGCTGGAATTCTCGGGTGCCAAGGAA | 200005 | 0.8097343178322886 | RNA PCR Primer, Index 1 (100% over 23bp) |
| TCTTTTGGCAGGTGAGTAGAGCCGTTCGTGACTGGAATTCTCGGGTGCCAA | 199274 | 0.806774812888235 | No Hit |
| GCCGAGAACTGATGACGAGTTTGGAATTCTCGGGTGCCAAGGAACTCCAGT | 162765 | 0.6589655570709354 | RNA PCR Primer, Index 1 (100% over 30bp) |
| AGGTGAGTAGAGCCGTTCGTGACTGGAATTCTCGGGTGCCAAGGAACTCCA | 160617 | 0.6502692279056458 | RNA PCR Primer, Index 1 (100% over 28bp) |
| ATCAAGGCCGAGAACTGATGACGAGTTATTGGAATTCTCGGGTGCCAAGGA | 155775 | 0.63066605015037 | RNA PCR Primer, Index 1 (100% over 22bp) |
| TGAGAACTGAATTCCATAGATGGTGGAATTCTCGGGTGCCAAGGAACTCCA | 141084 | 0.5711885027726836 | RNA PCR Primer, Index 1 (100% over 28bp) |
| AAGGCCGAGAACTGATGACGAGTTTGGAATTCTCGGGTGCCAAGGAACTCC | 139105 | 0.5631763820007524 | RNA PCR Primer, Index 1 (100% over 27bp) |
| TCAAGGCCGAGAACTGATGACGAGTTATTGGAATTCTCGGGTGCCAAGGAA | 133127 | 0.5389740282995878 | RNA PCR Primer, Index 1 (100% over 23bp) |
| CAAGGCCGAGAACTGATGACGAGTTTGGAATTCTCGGGTGCCAAGGAACTC | 124990 | 0.5060308111590097 | RNA PCR Primer, Index 1 (100% over 26bp) |
| GAATTAGTGGAAGGCTCTGGAAAGTGCTGGAATTCTCGGGTGCCAAGGAAC | 115348 | 0.4669944956042039 | RNA PCR Primer, Index 1 (100% over 24bp) |
| ATCAAGGCCGAGAACTGATGACGAGTTATGGAATTCTCGGGTGCCAAGGAA | 108002 | 0.43725369763017335 | RNA PCR Primer, Index 1 (100% over 23bp) |
| AGGTGAGTAGAGCCGTTCGTGACATGGAATTCTCGGGTGCCAAGGAACTCC | 105875 | 0.4286423884427567 | RNA PCR Primer, Index 1 (100% over 27bp) |
| GGAATACCAGGTGCTGTAAGCTTTGGAATTCTCGGGTGCCAAGGAACTCCA | 88436 | 0.3580393696748395 | RNA PCR Primer, Index 1 (100% over 28bp) |
| TCAAGGCCGAGAACTGATGACGAGTTATGGAATTCTCGGGTGCCAAGGAAC | 87182 | 0.35296246242471224 | RNA PCR Primer, Index 1 (100% over 24bp) |
| CTAAGACTGAGATACGAGACGAGCCTGGAATTCTCGGGTGCCAAGGAACTC | 83865 | 0.3395333544911621 | RNA PCR Primer, Index 1 (100% over 26bp) |
| CCGAGAAGACGATCAAACTTGGAATTCTCGGGTGCCAAGGAACTCCAGTCA | 80944 | 0.32770748042607317 | RNA PCR Primer, Index 1 (100% over 32bp) |
| TGAGATTAGCGGAACGCTCTGGAAAGTGCTGGAATTCTCGGGTGCCAAGGA | 75487 | 0.3056144318902326 | RNA PCR Primer, Index 1 (100% over 22bp) |
| CAAGGCCGAGAACTGATGACGAGTTATTGGAATTCTCGGGTGCCAAGGAAC | 71394 | 0.28904363334575844 | RNA PCR Primer, Index 1 (100% over 24bp) |
| CCGAGAAGACGATCAAACTTGATGGAATTCTCGGGTGCCAAGGAACTCCAG | 68288 | 0.27646877376131257 | RNA PCR Primer, Index 1 (100% over 29bp) |
| GCACCGAAGCTGTGGACTTGCTGGAATTCTCGGGTGCCAAGGAACTCCAGT | 67774 | 0.2743878085886129 | RNA PCR Primer, Index 1 (100% over 30bp) |
| TCTTTTGGCAGGTGAGTAGAGCCGTTCGTGATGGAATTCTCGGGTGCCAAG | 66699 | 0.2700355954355932 | No Hit |
| GCCGAGAGCTGATGACGAGTTTGGAATTCTCGGGTGCCAAGGAACTCCAGT | 63031 | 0.2551854393004524 | RNA PCR Primer, Index 1 (100% over 30bp) |
| AGGCCGAGAACTGATGACGAGTTTGGAATTCTCGGGTGCCAAGGAACTCCA | 62915 | 0.25471580513696374 | RNA PCR Primer, Index 1 (100% over 28bp) |
| AAGGCCGAGAACTGATGACGAGTTATTGGAATTCTCGGGTGCCAAGGAACT | 61335 | 0.24831906394461845 | RNA PCR Primer, Index 1 (100% over 25bp) |
| GCCGAGAACTGATGACGAGTTATTGGAATTCTCGGGTGCCAAGGAACTCCA | 58904 | 0.23847698936323153 | RNA PCR Primer, Index 1 (100% over 28bp) |
| ATCGGGGGCCTGAGTCCTGGAATTCTCGGGTGCCAAGGAACTCCAGTCACG | 57969 | 0.23469157606269808 | RNA PCR Primer, Index 6 (100% over 34bp) |
| AGACTGAGATACGAGACGAGCCTGGAATTCTCGGGTGCCAAGGAACTCCAG | 57324 | 0.23208024817088627 | RNA PCR Primer, Index 1 (100% over 29bp) |
| TCTTTTGGCAGGTGAGTAGAGCCGTTCGTGACATGGAATTCTCGGGTGCCA | 57313 | 0.23203571389676234 | No Hit |
| GCCGAGAAGACGATCAAACTTGATGGAATTCTCGGGTGCCAAGGAACTCCA | 51057 | 0.206707857631375 | RNA PCR Primer, Index 1 (100% over 28bp) |
| AGGTGAGTAGAGCCGTTCGTGATGGAATTCTCGGGTGCCAAGGAACTCCAG | 50121 | 0.20291839576046666 | RNA PCR Primer, Index 1 (100% over 29bp) |
| CAAGGCCGAGAACTGATGACGAGTTATGGAATTCTCGGGTGCCAAGGAACT | 47663 | 0.19296700977895737 | RNA PCR Primer, Index 1 (100% over 25bp) |
| AAGGCCGAGAACTGATGACGAGTTATGGAATTCTCGGGTGCCAAGGAACTC | 46737 | 0.18921803361179804 | RNA PCR Primer, Index 1 (100% over 26bp) |
| TGAGAATAGTGGAAGGCTCTGGAAAGTGCTGGAATTCTCGGGTGCCAAGGA | 42526 | 0.17216950376308543 | RNA PCR Primer, Index 1 (100% over 22bp) |
| GCCGAGAACTGATGACGAGTTATGGAATTCTCGGGTGCCAAGGAACTCCAG | 41277 | 0.16711283936483276 | RNA PCR Primer, Index 1 (100% over 29bp) |
| AGGTGTAGAATAAGTGGGAGGCCCTGGAATTCTCGGGTGCCAAGGAACTCC | 41228 | 0.16691445941646255 | RNA PCR Primer, Index 1 (100% over 27bp) |
| CTAAGGCCGAGAGCTGATGACGAGTCTGGAATTCTCGGGTGCCAAGGAACT | 39752 | 0.1609387695431071 | RNA PCR Primer, Index 1 (100% over 25bp) |
| CTAAGGCCGAGAGCTGATGACGAGTCATTGGAATTCTCGGGTGCCAAGGAA | 38976 | 0.1577970789321831 | RNA PCR Primer, Index 1 (100% over 23bp) |
| GCACCCGTAGCTCAGCTGGATGGAATTCTCGGGTGCCAAGGAACTCCAGTC | 38652 | 0.15648534213071483 | RNA PCR Primer, Index 1 (100% over 31bp) |
| GAGGTGTAGAATAAGTGGGAGGCCCTGGAATTCTCGGGTGCCAAGGAACTC | 38501 | 0.1558740080041046 | RNA PCR Primer, Index 1 (100% over 26bp) |
| AGAATTAGTGGAAGGCTCTGGAAAGTGCTGGAATTCTCGGGTGCCAAGGAA | 35676 | 0.14443679669500625 | RNA PCR Primer, Index 1 (100% over 23bp) |
| CTAAGGCCGAGAGCTGATGACGAGTCATGGAATTCTCGGGTGCCAAGGAAC | 33701 | 0.13644087020457468 | RNA PCR Primer, Index 1 (100% over 24bp) |
| AATTAGTGGAAGGCTCTGGAAAGTGCTGGAATTCTCGGGTGCCAAGGAACT | 32745 | 0.1325704369261683 | RNA PCR Primer, Index 1 (100% over 25bp) |
| TGAGATTAGCGGAACGCTGGAATTCTCGGGTGCCAAGGAACTCCAGTCACG | 32402 | 0.13118177728757688 | RNA PCR Primer, Index 6 (100% over 34bp) |
| GTTAAGCCGGGAACTTTAAGGATACTGCCTGGAATTCTCGGGTGCCAAGGA | 31108 | 0.1259429272224536 | RNA PCR Primer, Index 1 (100% over 22bp) |
| CTCCGGGGATGCGTGCATTTATCAGATCTGGAATTCTCGGGTGCCAAGGAA | 30427 | 0.12318585079714528 | RNA PCR Primer, Index 1 (100% over 23bp) |
| CAGGTGAGTAGAGCCGTTCGTGACTGGAATTCTCGGGTGCCAAGGAACTCC | 30368 | 0.12294698514502607 | RNA PCR Primer, Index 1 (100% over 27bp) |
| CTTAATGCCGAGAACTGATGACGATCCTTGGAATTCTCGGGTGCCAAGGAA | 29847 | 0.12083767997970209 | RNA PCR Primer, Index 1 (100% over 23bp) |
| AAGACTGAGATACGAGACGAGCCTGGAATTCTCGGGTGCCAAGGAACTCCA | 29564 | 0.1196919345636048 | RNA PCR Primer, Index 1 (100% over 28bp) |
| CCACAAGATTAAGAATGAGGCAATGATTGGAATTCTCGGGTGCCAAGGAAC | 29286 | 0.11856643199938202 | RNA PCR Primer, Index 1 (100% over 24bp) |
| CTTTCGAGGCCCTGTAATTGGAATGAGTATGGAATTCTCGGGTGCCAAGGA | 28740 | 0.1163559125746855 | RNA PCR Primer, Index 1 (100% over 22bp) |
| CCTAAGACTGAGATACGAGACGAGCCTGGAATTCTCGGGTGCCAAGGAACT | 28700 | 0.11619396975968942 | RNA PCR Primer, Index 1 (100% over 25bp) |
| GCCGAGAGCTGATGACGAGTCTGGAATTCTCGGGTGCCAAGGAACTCCAGT | 28699 | 0.11618992118931451 | RNA PCR Primer, Index 1 (100% over 30bp) |
| TGACTGTAAATGGTGATTAAATGCATTGGAATTCTCGGGTGCCAAGGAACT | 28468 | 0.11525470143271213 | RNA PCR Primer, Index 1 (100% over 25bp) |
| GGCCGAGAACTGATGACGAGTTTGGAATTCTCGGGTGCCAAGGAACTCCAG | 27472 | 0.11122232533930967 | RNA PCR Primer, Index 1 (100% over 29bp) |
| AGGCCGAGAACTGATGACGAGTTATTGGAATTCTCGGGTGCCAAGGAACTC | 27184 | 0.11005633707133787 | RNA PCR Primer, Index 1 (100% over 26bp) |
| AGGTGTAGAATAAGTGGGAGGCCCCGTGGAATTCTCGGGTGCCAAGGAACT | 27153 | 0.10993083138971592 | RNA PCR Primer, Index 1 (100% over 25bp) |
| CGTCTGGCGGGCACGGGAAATGTGGTGTATATGGAATTCTCGGGTGCCAAG | 27149 | 0.1099146371082163 | No Hit |
| TTAATGCCGAGAACTGATGACGATCCTTGGAATTCTCGGGTGCCAAGGAAC | 26990 | 0.10927091441860688 | RNA PCR Primer, Index 1 (100% over 24bp) |
| TGCTTGACGACCATAGAGAATGGAATTCTCGGGTGCCAAGGAACTCCAGTC | 26737 | 0.10824662611375664 | RNA PCR Primer, Index 1 (100% over 31bp) |
| GAGATTAGCGGAACGCTGGAATTCTCGGGTGCCAAGGAACTCCAGTCACGA | 26306 | 0.10650169228217385 | RNA PCR Primer, Index 7 (97% over 35bp) |
| TTTTGGCAGGTGAGTAGAGCCGTTCGTGATGGAATTCTCGGGTGCCAAGGA | 25702 | 0.10405635577573301 | RNA PCR Primer, Index 1 (100% over 22bp) |
| GTAAGGCGAACCAGGGGAACTGAAACTGGAATTCTCGGGTGCCAAGGAACT | 25214 | 0.10208065343278079 | RNA PCR Primer, Index 1 (100% over 25bp) |
| GGTGAGTAGAGCCGTTCGTGACTGGAATTCTCGGGTGCCAAGGAACTCCAG | 25127 | 0.1017284278101643 | RNA PCR Primer, Index 1 (100% over 29bp) |
| CGAGAAGACGATCAAACTTGATGGAATTCTCGGGTGCCAAGGAACTCCAGT | 25106 | 0.10164340783229138 | RNA PCR Primer, Index 1 (100% over 30bp) |

## Adapter Content

Produced by FastQC (version 0.11.9)
