# Supplementary material for: High Throughput Sequencing of MicroRNA in Rainbow Trout Plasma, Mucus, and Surrounding Water Following Acute Stress
Source: Front Physiol. 2021 Jan 13;11:588313. doi: 10.3389/fphys.2020.588313 (PMC7838646; doi:10.3389/fphys.2020.588313)
Supplement: Supplementary file 2 [file Data_Sheet_1.ZIP › Supplemental Quality Control/FastQC_raw_files/water_control_3_fastqc_raw.html]

SV18263\_0017\_S29\_R1\_001.fastq FastQC Report 

FastQC Report

Thu 7 May 2020  
SV18263\_0017\_S29\_R1\_001.fastq

## Summary

- Basic Statistics
- Per base sequence quality
- Per tile sequence quality
- Per sequence quality scores
- Per base sequence content
- Per sequence GC content
- Per base N content
- Sequence Length Distribution
- Sequence Duplication Levels
- Overrepresented sequences
- Adapter Content

## Basic Statistics

| Measure | Value |
| --- | --- |
| Filename | SV18263\_0017\_S29\_R1\_001.fastq |
| File type | Conventional base calls |
| Encoding | Sanger / Illumina 1.9 |
| Total Sequences | 12686917 |
| Sequences flagged as poor quality | 0 |
| Sequence length | 51 |
| %GC | 52 |

## Per base sequence quality

## Per tile sequence quality

## Per sequence quality scores

## Per base sequence content

## Per sequence GC content

## Per base N content

## Sequence Length Distribution

## Sequence Duplication Levels

## Overrepresented sequences

| Sequence | Count | Percentage | Possible Source |
| --- | --- | --- | --- |
| AGGTGAGTAGAGCCGTTCGTGACTGGAATTCTCGGGTGCCAAGGAACTCCA | 113504 | 0.8946539178903747 | RNA PCR Primer, Index 1 (100% over 28bp) |
| TGAGAACTGAATTCCATAGATGGTGGAATTCTCGGGTGCCAAGGAACTCCA | 86478 | 0.6816313214628897 | RNA PCR Primer, Index 1 (100% over 28bp) |
| GGAATACCAGGTGCTGTAAGCTTTGGAATTCTCGGGTGCCAAGGAACTCCA | 85477 | 0.6737413037383314 | RNA PCR Primer, Index 1 (100% over 28bp) |
| TCTTTTGGCAGGTGAGTAGAGCCGTTCGTGACTGGAATTCTCGGGTGCCAA | 76510 | 0.6030621939120434 | No Hit |
| ATTTGGAATTGTACAGTCAAGGTGTTGGAATTCTCGGGTGCCAAGGAACTC | 73569 | 0.5798808331448846 | RNA PCR Primer, Index 1 (100% over 26bp) |
| CCGAGAAGACGATCAAACTTGGAATTCTCGGGTGCCAAGGAACTCCAGTCA | 67479 | 0.5318786274080614 | RNA PCR Primer, Index 1 (100% over 32bp) |
| GCACCGAAGCTGTGGACTTGCTGGAATTCTCGGGTGCCAAGGAACTCCAGT | 51708 | 0.40756946703442615 | RNA PCR Primer, Index 1 (100% over 30bp) |
| ATCGGGGGCCTGAGTCCTGGAATTCTCGGGTGCCAAGGAACTCCAGTCACT | 50352 | 0.3968812911757837 | RNA PCR Primer, Index 3 (100% over 34bp) |
| CTAAGACTGAGATACGAGACGAGCCTGGAATTCTCGGGTGCCAAGGAACTC | 47373 | 0.373400409256244 | RNA PCR Primer, Index 1 (100% over 26bp) |
| AGGTGAGTAGAGCCGTTCGTGACATGGAATTCTCGGGTGCCAAGGAACTCC | 45530 | 0.3588736333657736 | RNA PCR Primer, Index 1 (100% over 27bp) |
| AGGTGAGTAGAGCCGTTCGTGATGGAATTCTCGGGTGCCAAGGAACTCCAG | 39165 | 0.3087038403419838 | RNA PCR Primer, Index 1 (100% over 29bp) |
| AGAATAGTGGAAGGCTCTGGAAAGTGCTGGAATTCTCGGGTGCCAAGGAAC | 38954 | 0.3070407097327113 | RNA PCR Primer, Index 1 (100% over 24bp) |
| ATCAAGGCCGAGAACTGATGACGAGTTTGGAATTCTCGGGTGCCAAGGAAC | 34794 | 0.27425102568259885 | RNA PCR Primer, Index 1 (100% over 24bp) |
| GAGAATAGTGGAAGGCTCTGGAAAGTGCTGGAATTCTCGGGTGCCAAGGAA | 34675 | 0.2733130515475115 | RNA PCR Primer, Index 1 (100% over 23bp) |
| GAATTAGTGGAAGGCTCTGGAAAGTGCTGGAATTCTCGGGTGCCAAGGAAC | 32553 | 0.2565871598277186 | RNA PCR Primer, Index 1 (100% over 24bp) |
| ATCAAGGCCGAGAACTGATGACGAGTTATTGGAATTCTCGGGTGCCAAGGA | 30063 | 0.23696064221118496 | RNA PCR Primer, Index 1 (100% over 22bp) |
| CCGAGAAGACGATCAAACTTGATGGAATTCTCGGGTGCCAAGGAACTCCAG | 28608 | 0.2254921349292346 | RNA PCR Primer, Index 1 (100% over 29bp) |
| TCAAGGCCGAGAACTGATGACGAGTTTGGAATTCTCGGGTGCCAAGGAACT | 28096 | 0.2214564815076823 | RNA PCR Primer, Index 1 (100% over 25bp) |
| AGACTGAGATACGAGACGAGCCTGGAATTCTCGGGTGCCAAGGAACTCCAG | 27638 | 0.21784646340793434 | RNA PCR Primer, Index 1 (100% over 29bp) |
| CTGTGAGGATCTGATAGTATGGCGACTTGGAATTCTCGGGTGCCAAGGAAC | 26464 | 0.20859283622648433 | RNA PCR Primer, Index 1 (100% over 24bp) |
| TTCTATACCGAGATCTGATAGCAAGCTTGGAATTCTCGGGTGCCAAGGAAC | 25863 | 0.20385567273751376 | RNA PCR Primer, Index 1 (100% over 24bp) |
| GCCGAGAAGACGATCAAACTTGATGGAATTCTCGGGTGCCAAGGAACTCCA | 24600 | 0.1939005354886455 | RNA PCR Primer, Index 1 (100% over 28bp) |
| TCAAGGCCGAGAACTGATGACGAGTTATTGGAATTCTCGGGTGCCAAGGAA | 24329 | 0.19176447674403482 | RNA PCR Primer, Index 1 (100% over 23bp) |
| GTGTGGTCGGATCCCTGGAATTCTCGGGTGCCAAGGAACTCCAGTCACTAC | 23381 | 0.1842922122056919 | RNA PCR Primer, Index 30 (97% over 36bp) |
| ATCAAGGCCGAGAACTGATGACGAGTTATGGAATTCTCGGGTGCCAAGGAA | 23209 | 0.18293648488438916 | RNA PCR Primer, Index 1 (100% over 23bp) |
| TCTTTTGGCAGGTGAGTAGAGCCGTTCGTGATGGAATTCTCGGGTGCCAAG | 22736 | 0.1792082347508067 | No Hit |
| TACCGAGATCTGATAGCAAGCTTGGAATTCTCGGGTGCCAAGGAACTCCAG | 22320 | 0.17592926634579542 | RNA PCR Primer, Index 1 (100% over 29bp) |
| TCAAGGCCGAGAACTGATGACGAGTTATGGAATTCTCGGGTGCCAAGGAAC | 20642 | 0.16270304282750492 | RNA PCR Primer, Index 1 (100% over 24bp) |
| AGATTAGCGGAACGCTCTGGAAAGTGCTGGAATTCTCGGGTGCCAAGGAAC | 20180 | 0.15906149618540108 | RNA PCR Primer, Index 1 (100% over 24bp) |
| CTCCGGGGATGCGTGCATTTATCAGATCTGGAATTCTCGGGTGCCAAGGAA | 19850 | 0.15646039144104118 | RNA PCR Primer, Index 1 (100% over 23bp) |
| CGAGAAGACGATCAAACTTGACTGGAATTCTCGGGTGCCAAGGAACTCCAG | 19746 | 0.15564064933978838 | RNA PCR Primer, Index 1 (100% over 29bp) |
| TAAGCCGAGCAATACTAATGAATCTGGAATTCTCGGGTGCCAAGGAACTCC | 19724 | 0.15546724235683107 | RNA PCR Primer, Index 1 (100% over 27bp) |
| CGAGAAGACGATCAAACTTGATGGAATTCTCGGGTGCCAAGGAACTCCAGT | 19482 | 0.15355976554430048 | RNA PCR Primer, Index 1 (100% over 30bp) |
| CGAGAAGACGATCAAACTTGACTATTGGAATTCTCGGGTGCCAAGGAACTC | 18624 | 0.1467968932089648 | RNA PCR Primer, Index 1 (100% over 26bp) |
| GAATACCAGGTGCTGTAAGCTTTGGAATTCTCGGGTGCCAAGGAACTCCAG | 18404 | 0.14506282337939155 | RNA PCR Primer, Index 1 (100% over 29bp) |
| CCTAAGACTGAGATACGAGACGAGCCTGGAATTCTCGGGTGCCAAGGAACT | 18120 | 0.14282429687212425 | RNA PCR Primer, Index 1 (100% over 25bp) |
| CGTCTGGCGGGCACGGGAATGGAATTCTCGGGTGCCAAGGAACTCCAGTCA | 17571 | 0.1384970044337801 | RNA PCR Primer, Index 1 (100% over 32bp) |
| AAGGCCGAGAACTGATGACGAGTTTGGAATTCTCGGGTGCCAAGGAACTCC | 17093 | 0.13472934362225275 | RNA PCR Primer, Index 1 (100% over 27bp) |
| CGTCTGGCGGGCACGGGAAATGTGGTGTATATGGAATTCTCGGGTGCCAAG | 16818 | 0.1325617563352862 | No Hit |
| TCTTTTGGCAGGTGAGTAGAGCCGTTCGTGACATGGAATTCTCGGGTGCCA | 16258 | 0.12814776040546336 | No Hit |
| AAGACTGAGATACGAGACGAGCCTGGAATTCTCGGGTGCCAAGGAACTCCA | 15778 | 0.12436433532275809 | RNA PCR Primer, Index 1 (100% over 28bp) |
| CAGGTGAGTAGAGCCGTTCGTGACTGGAATTCTCGGGTGCCAAGGAACTCC | 15423 | 0.12156617718867396 | RNA PCR Primer, Index 1 (100% over 27bp) |
| AGGTGTAGAATAAGTGGGAGGCCCTGGAATTCTCGGGTGCCAAGGAACTCC | 14643 | 0.11541811142927791 | RNA PCR Primer, Index 1 (100% over 27bp) |
| GAGATTAGCGGAACGCTCTGGAAAGTGCTGGAATTCTCGGGTGCCAAGGAA | 14558 | 0.11474812990421551 | RNA PCR Primer, Index 1 (100% over 23bp) |
| GGTGAGTAGAGCCGTTCGTGACTGGAATTCTCGGGTGCCAAGGAACTCCAG | 13863 | 0.10927004566988181 | RNA PCR Primer, Index 1 (100% over 29bp) |
| CAAGGCCGAGAACTGATGACGAGTTTGGAATTCTCGGGTGCCAAGGAACTC | 13366 | 0.10535262428216406 | RNA PCR Primer, Index 1 (100% over 26bp) |
| AGGCCGAGAACTGATGACGAGTTTGGAATTCTCGGGTGCCAAGGAACTCCA | 13318 | 0.10497428177389354 | RNA PCR Primer, Index 1 (100% over 28bp) |
| CTTTCGAGGCCCTGTAATTGGAATGAGTATGGAATTCTCGGGTGCCAAGGA | 12805 | 0.10093074621675227 | RNA PCR Primer, Index 1 (100% over 22bp) |

## Adapter Content

Produced by FastQC (version 0.11.9)
