# Supplementary material for: High Throughput Sequencing of MicroRNA in Rainbow Trout Plasma, Mucus, and Surrounding Water Following Acute Stress
Source: Front Physiol. 2021 Jan 13;11:588313. doi: 10.3389/fphys.2020.588313 (PMC7838646; doi:10.3389/fphys.2020.588313)
Supplement: Supplementary file 2 [file Data_Sheet_1.ZIP › Supplemental Quality Control/FastQC_raw_files/water_stressed_1_fastqc_raw.html]

SV18263\_0014\_S26\_R1\_001.fastq FastQC Report 

FastQC Report

Thu 7 May 2020  
SV18263\_0014\_S26\_R1\_001.fastq

## Summary

- Basic Statistics
- Per base sequence quality
- Per tile sequence quality
- Per sequence quality scores
- Per base sequence content
- Per sequence GC content
- Per base N content
- Sequence Length Distribution
- Sequence Duplication Levels
- Overrepresented sequences
- Adapter Content

## Basic Statistics

| Measure | Value |
| --- | --- |
| Filename | SV18263\_0014\_S26\_R1\_001.fastq |
| File type | Conventional base calls |
| Encoding | Sanger / Illumina 1.9 |
| Total Sequences | 18783772 |
| Sequences flagged as poor quality | 0 |
| Sequence length | 51 |
| %GC | 52 |

## Per base sequence quality

## Per tile sequence quality

## Per sequence quality scores

## Per base sequence content

## Per sequence GC content

## Per base N content

## Sequence Length Distribution

## Sequence Duplication Levels

## Overrepresented sequences

| Sequence | Count | Percentage | Possible Source |
| --- | --- | --- | --- |
| TGAGAACTGAATTCCATAGATGGTGGAATTCTCGGGTGCCAAGGAACTCCA | 1417481 | 7.546306460704484 | RNA PCR Primer, Index 1 (100% over 28bp) |
| CTTTTGGCAGGTGAGTAGAGCCGTTCGTGACATGGAATTCTCGGGTGCCAA | 404328 | 2.152538904326564 | No Hit |
| TTGGCAGGTGAGTAGAGCCGTTCGTGATGGAATTCTCGGGTGCCAAGGAAC | 285762 | 1.521323832082289 | RNA PCR Primer, Index 1 (100% over 24bp) |
| CCGAGAAGACGATCAAACTTGATGGAATTCTCGGGTGCCAAGGAACTCCAG | 260379 | 1.386191229322843 | RNA PCR Primer, Index 1 (100% over 29bp) |
| CAGGTGAGTAGAGCCGTTCGTGACATGGAATTCTCGGGTGCCAAGGAACTC | 230379 | 1.2264788989133812 | RNA PCR Primer, Index 1 (100% over 26bp) |
| GAGGTGTAGAATAAGTGGGAGGCCCTGGAATTCTCGGGTGCCAAGGAACTC | 211852 | 1.1278458873968444 | RNA PCR Primer, Index 1 (100% over 26bp) |
| GCCGAGAAGACGATCAAACTTGATGGAATTCTCGGGTGCCAAGGAACTCCA | 206644 | 1.1001198268377619 | RNA PCR Primer, Index 1 (100% over 28bp) |
| TAGCTTATCAGACTGGTGTTGGTGGAATTCTCGGGTGCCAAGGAACTCCAG | 179505 | 0.9556387290050156 | RNA PCR Primer, Index 1 (100% over 29bp) |
| TACCCTGTAGAACCGAATTTGTTGGAATTCTCGGGTGCCAAGGAACTCCAG | 167762 | 0.8931219991384052 | RNA PCR Primer, Index 1 (100% over 29bp) |
| GGTGAGTAGAGCCGTTCGTGACATGGAATTCTCGGGTGCCAAGGAACTCCA | 155886 | 0.8298972112736462 | RNA PCR Primer, Index 1 (100% over 28bp) |
| TTGGCAGGTGAGTAGAGCCGTTCGTGACATGGAATTCTCGGGTGCCAAGGA | 153528 | 0.8173438221034625 | RNA PCR Primer, Index 1 (100% over 22bp) |
| TTTTGGCAGGTGAGTAGAGCCGTTCGTGACATGGAATTCTCGGGTGCCAAG | 112277 | 0.5977340440461053 | No Hit |
| TTTTGGCAGGTGAGTAGAGCCGTTCGTGATGGAATTCTCGGGTGCCAAGGA | 108122 | 0.5756138862843948 | RNA PCR Primer, Index 1 (100% over 22bp) |
| GGAATACCAGGTGCTGTAAGCTTTGGAATTCTCGGGTGCCAAGGAACTCCA | 106419 | 0.5665475496614844 | RNA PCR Primer, Index 1 (100% over 28bp) |
| AGGTGTAGAATAAGTGGGAGGCCCTGGAATTCTCGGGTGCCAAGGAACTCC | 96029 | 0.5112338458963407 | RNA PCR Primer, Index 1 (100% over 27bp) |
| AACCCGTAGATCCGAACTTGTTGGAATTCTCGGGTGCCAAGGAACTCCAGT | 94950 | 0.5054895257459471 | RNA PCR Primer, Index 1 (100% over 30bp) |
| TGAGAACTGAATTCCATAGATGTGGAATTCTCGGGTGCCAAGGAACTCCAG | 93084 | 0.49555541879447856 | RNA PCR Primer, Index 1 (100% over 29bp) |
| GATCGGGGGCCTGAGTCCTTGGAATTCTCGGGTGCCAAGGAACTCCAGTCA | 92899 | 0.49457052609028684 | RNA PCR Primer, Index 1 (100% over 32bp) |
| TCTTTTGGCAGGTGAGTAGAGCCGTTCGTGATGGAATTCTCGGGTGCCAAG | 85944 | 0.4575438841570266 | No Hit |
| TAGCTTATCAGACTGGTGTTGGCTGGAATTCTCGGGTGCCAAGGAACTCCA | 85607 | 0.4557497823120937 | RNA PCR Primer, Index 1 (100% over 28bp) |
| TAACGGAACCCATAATGCAGCTGTGGAATTCTCGGGTGCCAAGGAACTCCA | 84269 | 0.4486266123758316 | RNA PCR Primer, Index 1 (100% over 28bp) |
| AGGTGAGTAGAGCCGTTCGTGACATGGAATTCTCGGGTGCCAAGGAACTCC | 83965 | 0.44700819409434916 | RNA PCR Primer, Index 1 (100% over 27bp) |
| AACCCGTAGATCCGAACTTGTGTGGAATTCTCGGGTGCCAAGGAACTCCAG | 78914 | 0.4201179613977427 | RNA PCR Primer, Index 1 (100% over 29bp) |
| CTTTTGGCAGGTGAGTAGAGCCGTTCGTGATGGAATTCTCGGGTGCCAAGG | 75468 | 0.4017723383780425 | Illumina Small RNA Adapter 2 (100% over 21bp) |
| TAACACTGTCTGGTAACGATGTGGAATTCTCGGGTGCCAAGGAACTCCAGT | 73527 | 0.3914389506005503 | RNA PCR Primer, Index 1 (100% over 30bp) |
| TGAGAACTGAATTCCATAGATGGTTGGAATTCTCGGGTGCCAAGGAACTCC | 73516 | 0.3913803894127335 | RNA PCR Primer, Index 1 (100% over 27bp) |
| CAGGTGAGTAGAGCCGTTCGTGATGGAATTCTCGGGTGCCAAGGAACTCCA | 68943 | 0.3670349065139845 | RNA PCR Primer, Index 1 (100% over 28bp) |
| TCTTTTGGCAGGTGAGTAGAGCCGTTCGTGACTGGAATTCTCGGGTGCCAA | 68714 | 0.3658157690585256 | No Hit |
| TTTTGGCAGGTGAGTAGAGCCGTTCGTGACTGGAATTCTCGGGTGCCAAGG | 66225 | 0.35256496937888726 | Illumina Small RNA Adapter 2 (100% over 21bp) |
| TGAAATGTTTAGGACCACTCGTGGAATTCTCGGGTGCCAAGGAACTCCAGT | 60467 | 0.32191084942896453 | RNA PCR Primer, Index 1 (100% over 30bp) |
| GAATACCAGGTGCTGTAAGCTTTGGAATTCTCGGGTGCCAAGGAACTCCAG | 59824 | 0.31848768181385506 | RNA PCR Primer, Index 1 (100% over 29bp) |
| CTCCGGGGATGCGTGCATTTATCAGATCTGGAATTCTCGGGTGCCAAGGAA | 56893 | 0.30288378713285063 | RNA PCR Primer, Index 1 (100% over 23bp) |
| CTTTTGGCAGGTGAGTAGAGCCGTTCGTGACTGGAATTCTCGGGTGCCAAG | 52735 | 0.2807476581380992 | No Hit |
| TGGACGGAGAACTGATAAGGTGGAATTCTCGGGTGCCAAGGAACTCCAGTC | 49318 | 0.2625564237044615 | RNA PCR Primer, Index 1 (100% over 31bp) |
| TCTTTTGGCAGGTGAGTAGAGCCGTTCGTGACATGGAATTCTCGGGTGCCA | 48157 | 0.25637555651761534 | No Hit |
| GCATTGGTGGTTCAGTGGTAGAATTCTCGCCTTGGAATTCTCGGGTGCCAA | 44376 | 0.23624647914167615 | No Hit |
| AGGTGAGTAGAGCCGTTCGTGACTGGAATTCTCGGGTGCCAAGGAACTCCA | 43499 | 0.23157755534937285 | RNA PCR Primer, Index 1 (100% over 28bp) |
| TAATACTGCCTGGTAATGATGATGGAATTCTCGGGTGCCAAGGAACTCCAG | 43208 | 0.2300283457444011 | RNA PCR Primer, Index 1 (100% over 29bp) |
| TAACGGAACCCATAAAGCAGCTGTGGAATTCTCGGGTGCCAAGGAACTCCA | 42363 | 0.2255297817712012 | RNA PCR Primer, Index 1 (100% over 28bp) |
| TGTCAACCGGGTCGGACTGTCCTCAGTGCGTACTGGAATTCTCGGGTGCCA | 40449 | 0.21534013509107752 | No Hit |
| CTTCGGAGTCTGTGGTAGGAAACCTGGAATTCTCGGGTGCCAAGGAACTCC | 39167 | 0.20851509483824654 | RNA PCR Primer, Index 1 (100% over 27bp) |
| TGGCGGGCACGGGAAATGTGGTGTATATGGAATTCTCGGGTGCCAAGGAAC | 39124 | 0.2082861738313263 | RNA PCR Primer, Index 1 (100% over 24bp) |
| GTCTGGCGGGCACGGGAAATGTGGTGTATATGGAATTCTCGGGTGCCAAGG | 38133 | 0.2030103431834671 | Illumina Small RNA Adapter 2 (100% over 21bp) |
| TTTGGCAGGTGAGTAGAGCCGTTCGTGATGGAATTCTCGGGTGCCAAGGAA | 37813 | 0.2013067449924328 | RNA PCR Primer, Index 1 (100% over 23bp) |
| GCATTGGTGGTTCAGTGGTAGAATTCTCGCCTGGAATTCTCGGGTGCCAAG | 35854 | 0.19087752981669495 | No Hit |
| GTGGTTGGCAGCGGCGACTCTGGACGCGTGCCTGGAATTCTCGGGTGCCAA | 34789 | 0.18520774208715907 | No Hit |
| GTGAAATGTTTAGGACCACTTGTGGAATTCTCGGGTGCCAAGGAACTCCAG | 33492 | 0.17830284566912333 | RNA PCR Primer, Index 1 (100% over 29bp) |
| GCATTGGTGGTTCAGTGGTAGAATTCTCGCTGGAATTCTCGGGTGCCAAGG | 32188 | 0.1713606830406587 | Illumina Small RNA Adapter 2 (100% over 21bp) |
| TGAGGTAGTAGATTGAATAGTTTGGAATTCTCGGGTGCCAAGGAACTCCAG | 31176 | 0.16597305376151286 | RNA PCR Primer, Index 1 (100% over 29bp) |
| CAGGTGAGTAGAGCCGTTCGTGACTGGAATTCTCGGGTGCCAAGGAACTCC | 30868 | 0.1643333405026424 | RNA PCR Primer, Index 1 (100% over 27bp) |
| TGAGGTAGTAGGTTGTATAGTTTGGAATTCTCGGGTGCCAAGGAACTCCAG | 29961 | 0.15950470437992964 | RNA PCR Primer, Index 1 (100% over 29bp) |
| TTTGGCAGGTGAGTAGAGCCGTTCGTGACATGGAATTCTCGGGTGCCAAGG | 29433 | 0.15669376736472312 | Illumina Small RNA Adapter 2 (100% over 21bp) |
| GTAGAGCCGTTCGTGACATGGAATTCTCGGGTGCCAAGGAACTCCAGTCAC | 28609 | 0.1523070020228099 | RNA PCR Primer, Index 1 (100% over 33bp) |
| GTGAAATGTTTAGGACCACTCGTGGAATTCTCGGGTGCCAAGGAACTCCAG | 28062 | 0.14939491386501072 | RNA PCR Primer, Index 1 (100% over 29bp) |
| CCGAGAAGACGATCAAACTTGTGGAATTCTCGGGTGCCAAGGAACTCCAGT | 27868 | 0.1483621074616962 | RNA PCR Primer, Index 1 (100% over 30bp) |
| CGGATTGAATTAGAATAACTTGGAAAAGTTGGAATTCTCGGGTGCCAAGGA | 26892 | 0.1431661329790417 | RNA PCR Primer, Index 1 (100% over 22bp) |
| TGAAATGTTTAGGACCACTCGATGGAATTCTCGGGTGCCAAGGAACTCCAG | 26801 | 0.14268167224346634 | RNA PCR Primer, Index 1 (100% over 29bp) |
| TTCAAGTAATCCAGGATAGGCTTGGAATTCTCGGGTGCCAAGGAACTCCAG | 26651 | 0.141883110591419 | RNA PCR Primer, Index 1 (100% over 29bp) |
| AGGTGAGTAGAGCCGTTCGTGATGGAATTCTCGGGTGCCAAGGAACTCCAG | 26518 | 0.1411750525932704 | RNA PCR Primer, Index 1 (100% over 29bp) |
| GGTGAGTAGAGCCGTTCGTGACTGGAATTCTCGGGTGCCAAGGAACTCCAG | 25414 | 0.1352976388342022 | RNA PCR Primer, Index 1 (100% over 29bp) |
| GGTGAGTAGAGCCGTTCGTGATGGAATTCTCGGGTGCCAAGGAACTCCAGT | 25342 | 0.1349143292412195 | RNA PCR Primer, Index 1 (100% over 30bp) |
| GCATTGGTGGTTCAGTGGTAGAATTCTCTGGAATTCTCGGGTGCCAAGGAA | 23814 | 0.1267796478790309 | RNA PCR Primer, Index 1 (100% over 23bp) |
| TGAGGTAGTAGGTTGTATAGTTGGAATTCTCGGGTGCCAAGGAACTCCAGT | 23351 | 0.1243147542463782 | RNA PCR Primer, Index 1 (100% over 30bp) |
| TGAGGTAGTAGATTGAATAGTTGGAATTCTCGGGTGCCAAGGAACTCCAGT | 22756 | 0.12114712635992388 | RNA PCR Primer, Index 1 (100% over 30bp) |
| AAATTGATTTTTGGAATAGGGATGGAATTCTCGGGTGCCAAGGAACTCCAG | 22652 | 0.12059345694783773 | RNA PCR Primer, Index 1 (100% over 29bp) |
| GGCTTAGGCTGGCGGATCGTTTGAGCTGGAATTCTCGGGTGCCAAGGAACT | 22162 | 0.11798482221781652 | RNA PCR Primer, Index 1 (100% over 25bp) |
| CGGGAAATGTGGTGTATAGAAGACTGGAATTCTCGGGTGCCAAGGAACTCC | 21783 | 0.11596712311031032 | RNA PCR Primer, Index 1 (100% over 27bp) |
| CTTTTGGCAGGTGAGTAGAGCCGTTCGTGACAGTGGAATTCTCGGGTGCCA | 21118 | 0.11242683311956725 | No Hit |
| TGAGAACTGAATTCCATAGATGTTGGAATTCTCGGGTGCCAAGGAACTCCA | 21109 | 0.11237891942044442 | RNA PCR Primer, Index 1 (100% over 28bp) |
| AGAATTAGTGGAAGGCTCTGGAAAGTGCTGGAATTCTCGGGTGCCAAGGAA | 20955 | 0.11155906279100919 | RNA PCR Primer, Index 1 (100% over 23bp) |
| CTGATGCGCACCGCATGTTTGTGGAGAACCTGGAATTCTCGGGTGCCAAGG | 20851 | 0.11100539337892304 | Illumina Small RNA Adapter 2 (100% over 21bp) |
| AACATTCAACGCTGTCGGTGAGTGGAATTCTCGGGTGCCAAGGAACTCCAG | 20805 | 0.11076050113896187 | RNA PCR Primer, Index 1 (100% over 29bp) |
| TTTGGCAGGTGAGTAGAGCCGTTCGTGACTGGAATTCTCGGGTGCCAAGGA | 20604 | 0.10969042852521846 | RNA PCR Primer, Index 1 (100% over 22bp) |
| AATTGATTTTTGGAATAGGGATGGAATTCTCGGGTGCCAAGGAACTCCAGT | 20465 | 0.10895042806098795 | RNA PCR Primer, Index 1 (100% over 30bp) |
| TCGCGGATCTCCCCAGCTACGGTGCTCGCTGGCTGGAATTCTCGGGTGCCA | 20447 | 0.10885460066274229 | No Hit |
| CGTCTGGCGGGCACGGGAAATGTGGTGTATATGGAATTCTCGGGTGCCAAG | 20431 | 0.10876942075319057 | No Hit |
| TAATACTGCCTGGTAATGATGATTGGAATTCTCGGGTGCCAAGGAACTCCA | 19855 | 0.10570294400932889 | RNA PCR Primer, Index 1 (100% over 28bp) |
| ATCGGGGGCCTGAGTCCTGGAATTCTCGGGTGCCAAGGAACTCCAGTCACC | 19589 | 0.10428682801303167 | RNA PCR Primer, Index 2 (100% over 34bp) |
| GTGTGGTCGGATCCCTGGAATTCTCGGGTGCCAAGGAACTCCAGTCACCTC | 19146 | 0.10192840926731862 | RNA PCR Primer, Index 1 (97% over 36bp) |
| TGGACGGAGAACTGATAAGGGTGGAATTCTCGGGTGCCAAGGAACTCCAGT | 18961 | 0.10094351656312693 | RNA PCR Primer, Index 1 (100% over 30bp) |

## Adapter Content

Produced by FastQC (version 0.11.9)
