# Supplementary material for: High Throughput Sequencing of MicroRNA in Rainbow Trout Plasma, Mucus, and Surrounding Water Following Acute Stress
Source: Front Physiol. 2021 Jan 13;11:588313. doi: 10.3389/fphys.2020.588313 (PMC7838646; doi:10.3389/fphys.2020.588313)
Supplement: Supplementary file 2 [file Data_Sheet_1.ZIP › Supplemental Quality Control/FastQC_raw_files/water_stressed_2_fastqc_raw.html]

SV18263\_0016\_S28\_R1\_001.fastq FastQC Report 

FastQC Report

Thu 7 May 2020  
SV18263\_0016\_S28\_R1\_001.fastq

## Summary

- Basic Statistics
- Per base sequence quality
- Per tile sequence quality
- Per sequence quality scores
- Per base sequence content
- Per sequence GC content
- Per base N content
- Sequence Length Distribution
- Sequence Duplication Levels
- Overrepresented sequences
- Adapter Content

## Basic Statistics

| Measure | Value |
| --- | --- |
| Filename | SV18263\_0016\_S28\_R1\_001.fastq |
| File type | Conventional base calls |
| Encoding | Sanger / Illumina 1.9 |
| Total Sequences | 27056416 |
| Sequences flagged as poor quality | 0 |
| Sequence length | 51 |
| %GC | 52 |

## Per base sequence quality

## Per tile sequence quality

## Per sequence quality scores

## Per base sequence content

## Per sequence GC content

## Per base N content

## Sequence Length Distribution

## Sequence Duplication Levels

## Overrepresented sequences

| Sequence | Count | Percentage | Possible Source |
| --- | --- | --- | --- |
| TGAGAACTGAATTCCATAGATGGTGGAATTCTCGGGTGCCAAGGAACTCCA | 666389 | 2.4629610958081067 | RNA PCR Primer, Index 1 (100% over 28bp) |
| AGAATAGTGGAAGGCTCTGGAAAGTGCTGGAATTCTCGGGTGCCAAGGAAC | 435158 | 1.6083357086171355 | RNA PCR Primer, Index 1 (100% over 24bp) |
| GAGAATAGTGGAAGGCTCTGGAAAGTGCTGGAATTCTCGGGTGCCAAGGAA | 345803 | 1.278081324592289 | RNA PCR Primer, Index 1 (100% over 23bp) |
| TAGCTTATCAGACTGGTGTTGGTGGAATTCTCGGGTGCCAAGGAACTCCAG | 201407 | 0.7443964492562504 | RNA PCR Primer, Index 1 (100% over 29bp) |
| AGATTAGCGGAACGCTCTGGAAAGTGCTGGAATTCTCGGGTGCCAAGGAAC | 195129 | 0.7211930804139025 | RNA PCR Primer, Index 1 (100% over 24bp) |
| GAGATTAGCGGAACGCTCTGGAAAGTGCTGGAATTCTCGGGTGCCAAGGAA | 181037 | 0.6691093158827836 | RNA PCR Primer, Index 1 (100% over 23bp) |
| ATCAAGGCCGAGAACTGATGACGAGTTTGGAATTCTCGGGTGCCAAGGAAC | 155646 | 0.5752646618088664 | RNA PCR Primer, Index 1 (100% over 24bp) |
| GAATTAGTGGAAGGCTCTGGAAAGTGCTGGAATTCTCGGGTGCCAAGGAAC | 142457 | 0.526518368138633 | RNA PCR Primer, Index 1 (100% over 24bp) |
| TCTTTTGGCAGGTGAGTAGAGCCGTTCGTGACTGGAATTCTCGGGTGCCAA | 136903 | 0.5059908895546255 | No Hit |
| TCAAGGCCGAGAACTGATGACGAGTTTGGAATTCTCGGGTGCCAAGGAACT | 130770 | 0.48332343796014965 | RNA PCR Primer, Index 1 (100% over 25bp) |
| GCCGAGAACTGATGACGAGTTTGGAATTCTCGGGTGCCAAGGAACTCCAGT | 113372 | 0.419020760177549 | RNA PCR Primer, Index 1 (100% over 30bp) |
| AGGTGAGTAGAGCCGTTCGTGACTGGAATTCTCGGGTGCCAAGGAACTCCA | 113263 | 0.41861789824639006 | RNA PCR Primer, Index 1 (100% over 28bp) |
| AGGTGAGTAGAGCCGTTCGTGACATGGAATTCTCGGGTGCCAAGGAACTCC | 101890 | 0.3765835061081261 | RNA PCR Primer, Index 1 (100% over 27bp) |
| GGAATACCAGGTGCTGTAAGCTTTGGAATTCTCGGGTGCCAAGGAACTCCA | 100666 | 0.3720596253398824 | RNA PCR Primer, Index 1 (100% over 28bp) |
| TAACGGAACCCATAATGCAGCTGTGGAATTCTCGGGTGCCAAGGAACTCCA | 99809 | 0.36889216960590787 | RNA PCR Primer, Index 1 (100% over 28bp) |
| AAGGCCGAGAACTGATGACGAGTTTGGAATTCTCGGGTGCCAAGGAACTCC | 97652 | 0.36091993854618437 | RNA PCR Primer, Index 1 (100% over 27bp) |
| TACCCTGTAGAACCGAATTTGTTGGAATTCTCGGGTGCCAAGGAACTCCAG | 95517 | 0.35302901906889667 | RNA PCR Primer, Index 1 (100% over 29bp) |
| GCCGAGAAGACGATCAAACTTGATGGAATTCTCGGGTGCCAAGGAACTCCA | 93979 | 0.3473446002604336 | RNA PCR Primer, Index 1 (100% over 28bp) |
| TCTTTTGGCAGGTGAGTAGAGCCGTTCGTGATGGAATTCTCGGGTGCCAAG | 88083 | 0.32555309616765205 | No Hit |
| CTTTTGGCAGGTGAGTAGAGCCGTTCGTGACATGGAATTCTCGGGTGCCAA | 88049 | 0.32542743281297865 | No Hit |
| CCGAGAAGACGATCAAACTTGATGGAATTCTCGGGTGCCAAGGAACTCCAG | 87954 | 0.325076314616097 | RNA PCR Primer, Index 1 (100% over 29bp) |
| ATCAAGGCCGAGAACTGATGACGAGTTATTGGAATTCTCGGGTGCCAAGGA | 87509 | 0.3234316030622829 | RNA PCR Primer, Index 1 (100% over 22bp) |
| CAAGGCCGAGAACTGATGACGAGTTTGGAATTCTCGGGTGCCAAGGAACTC | 85435 | 0.3157661384272034 | RNA PCR Primer, Index 1 (100% over 26bp) |
| ATTTGGAATTGTACAGTCAAGGTGTTGGAATTCTCGGGTGCCAAGGAACTC | 81047 | 0.29954817371229064 | RNA PCR Primer, Index 1 (100% over 26bp) |
| TTTTGGCAGGTGAGTAGAGCCGTTCGTGATGGAATTCTCGGGTGCCAAGGA | 78861 | 0.2914687592029927 | RNA PCR Primer, Index 1 (100% over 22bp) |
| CTAAGACTGAGATACGAGACGAGCCTGGAATTCTCGGGTGCCAAGGAACTC | 77785 | 0.2874918836256805 | RNA PCR Primer, Index 1 (100% over 26bp) |
| TCAAGGCCGAGAACTGATGACGAGTTATTGGAATTCTCGGGTGCCAAGGAA | 77641 | 0.2869596623588283 | RNA PCR Primer, Index 1 (100% over 23bp) |
| AACCCGTAGATCCGAACTTGTTGGAATTCTCGGGTGCCAAGGAACTCCAGT | 73966 | 0.2733769321110379 | RNA PCR Primer, Index 1 (100% over 30bp) |
| CAGGTGAGTAGAGCCGTTCGTGACATGGAATTCTCGGGTGCCAAGGAACTC | 73533 | 0.2717765723294615 | RNA PCR Primer, Index 1 (100% over 26bp) |
| AACCCGTAGATCCGAACTTGTGTGGAATTCTCGGGTGCCAAGGAACTCCAG | 66578 | 0.24607102433670444 | RNA PCR Primer, Index 1 (100% over 29bp) |
| GAGGTGTAGAATAAGTGGGAGGCCCTGGAATTCTCGGGTGCCAAGGAACTC | 65728 | 0.24292944046986858 | RNA PCR Primer, Index 1 (100% over 26bp) |
| TTTTGGCAGGTGAGTAGAGCCGTTCGTGACTGGAATTCTCGGGTGCCAAGG | 64729 | 0.2392371554310815 | Illumina Small RNA Adapter 2 (100% over 21bp) |
| TAGCTTATCAGACTGGTGTTGGCTGGAATTCTCGGGTGCCAAGGAACTCCA | 63538 | 0.23483524203649145 | RNA PCR Primer, Index 1 (100% over 28bp) |
| GCACCGAAGCTGTGGACTTGCTGGAATTCTCGGGTGCCAAGGAACTCCAGT | 60062 | 0.22198801201164264 | RNA PCR Primer, Index 1 (100% over 30bp) |
| ATCAAGGCCGAGAACTGATGACGAGTTATGGAATTCTCGGGTGCCAAGGAA | 56880 | 0.21022740040661705 | RNA PCR Primer, Index 1 (100% over 23bp) |
| GGTGAGTAGAGCCGTTCGTGACATGGAATTCTCGGGTGCCAAGGAACTCCA | 56248 | 0.2078915404020991 | RNA PCR Primer, Index 1 (100% over 28bp) |
| TTGGCAGGTGAGTAGAGCCGTTCGTGATGGAATTCTCGGGTGCCAAGGAAC | 53709 | 0.19850744459280933 | RNA PCR Primer, Index 1 (100% over 24bp) |
| TGAGATTAGCGGAACGCTCTGGAAAGTGCTGGAATTCTCGGGTGCCAAGGA | 52843 | 0.19530672502965654 | RNA PCR Primer, Index 1 (100% over 22bp) |
| TCTTTTGGCAGGTGAGTAGAGCCGTTCGTGACATGGAATTCTCGGGTGCCA | 52220 | 0.19300412885431684 | No Hit |
| TCAAGGCCGAGAACTGATGACGAGTTATGGAATTCTCGGGTGCCAAGGAAC | 51728 | 0.19118570619257186 | RNA PCR Primer, Index 1 (100% over 24bp) |
| AGGCCGAGAACTGATGACGAGTTTGGAATTCTCGGGTGCCAAGGAACTCCA | 51541 | 0.19049455774186796 | RNA PCR Primer, Index 1 (100% over 28bp) |
| ATCGGGGGCCTGAGTCCTGGAATTCTCGGGTGCCAAGGAACTCCAGTCACT | 50748 | 0.1875636447931611 | RNA PCR Primer, Index 3 (100% over 34bp) |
| TAACACTGTCTGGTAACGATGTGGAATTCTCGGGTGCCAAGGAACTCCAGT | 49363 | 0.18244471108072852 | RNA PCR Primer, Index 1 (100% over 30bp) |
| GAATACCAGGTGCTGTAAGCTTTGGAATTCTCGGGTGCCAAGGAACTCCAG | 47941 | 0.17718902607056308 | RNA PCR Primer, Index 1 (100% over 29bp) |
| CTTTTGGCAGGTGAGTAGAGCCGTTCGTGATGGAATTCTCGGGTGCCAAGG | 45832 | 0.1693942020997903 | Illumina Small RNA Adapter 2 (100% over 21bp) |
| TAACGGAACCCATAAAGCAGCTGTGGAATTCTCGGGTGCCAAGGAACTCCA | 44292 | 0.16370239132928766 | RNA PCR Primer, Index 1 (100% over 28bp) |
| CAAGGCCGAGAACTGATGACGAGTTATTGGAATTCTCGGGTGCCAAGGAAC | 44088 | 0.16294841120124706 | RNA PCR Primer, Index 1 (100% over 24bp) |
| TGAGAACTGAATTCCATAGATGTGGAATTCTCGGGTGCCAAGGAACTCCAG | 42671 | 0.15771120609618067 | RNA PCR Primer, Index 1 (100% over 29bp) |
| AGGTGTAGAATAAGTGGGAGGCCCTGGAATTCTCGGGTGCCAAGGAACTCC | 41336 | 0.15277707143473843 | RNA PCR Primer, Index 1 (100% over 27bp) |
| TGAGGTAGTAGGTTGTATAGTTTGGAATTCTCGGGTGCCAAGGAACTCCAG | 40703 | 0.15043751544920067 | RNA PCR Primer, Index 1 (100% over 29bp) |
| CAGGTGAGTAGAGCCGTTCGTGACTGGAATTCTCGGGTGCCAAGGAACTCC | 40314 | 0.14899977883249577 | RNA PCR Primer, Index 1 (100% over 27bp) |
| TTTTGGCAGGTGAGTAGAGCCGTTCGTGACATGGAATTCTCGGGTGCCAAG | 40199 | 0.14857474101521798 | No Hit |
| TGAGAATAGTGGAAGGCTCTGGAAAGTGCTGGAATTCTCGGGTGCCAAGGA | 39635 | 0.1464902077200469 | RNA PCR Primer, Index 1 (100% over 22bp) |
| CTTTTGGCAGGTGAGTAGAGCCGTTCGTGACTGGAATTCTCGGGTGCCAAG | 38300 | 0.14155607305860465 | No Hit |
| AGAATTAGTGGAAGGCTCTGGAAAGTGCTGGAATTCTCGGGTGCCAAGGAA | 38123 | 0.14090188441809884 | RNA PCR Primer, Index 1 (100% over 23bp) |
| CATATACCGAGGCTGTTGATCGAGCGATGGAATTCTCGGGTGCCAAGGAAC | 37592 | 0.13893931849658137 | RNA PCR Primer, Index 1 (100% over 24bp) |
| TAAGCCGAGCAATACTAATGAATCTGGAATTCTCGGGTGCCAAGGAACTCC | 37252 | 0.137682684949847 | RNA PCR Primer, Index 1 (100% over 27bp) |
| TGGACGGAGAACTGATAAGGTGGAATTCTCGGGTGCCAAGGAACTCCAGTC | 36572 | 0.13516941785637832 | RNA PCR Primer, Index 1 (100% over 31bp) |
| AGGTGAGTAGAGCCGTTCGTGATGGAATTCTCGGGTGCCAAGGAACTCCAG | 36489 | 0.13486265143173434 | RNA PCR Primer, Index 1 (100% over 29bp) |
| GGTGAGTAGAGCCGTTCGTGACTGGAATTCTCGGGTGCCAAGGAACTCCAG | 35021 | 0.12943695129465782 | RNA PCR Primer, Index 1 (100% over 29bp) |
| CATATACCGAGGCTGTTGATCGAGCGTGGAATTCTCGGGTGCCAAGGAACT | 34964 | 0.1292262803765288 | RNA PCR Primer, Index 1 (100% over 25bp) |
| TGAAATGTTTAGGACCACTCGTGGAATTCTCGGGTGCCAAGGAACTCCAGT | 34560 | 0.1277331040445268 | RNA PCR Primer, Index 1 (100% over 30bp) |
| AAGGCCGAGAACTGATGACGAGTTATTGGAATTCTCGGGTGCCAAGGAACT | 34465 | 0.12738198584764515 | RNA PCR Primer, Index 1 (100% over 25bp) |
| AATTAGTGGAAGGCTCTGGAAAGTGCTGGAATTCTCGGGTGCCAAGGAACT | 33861 | 0.12514961331168178 | RNA PCR Primer, Index 1 (100% over 25bp) |
| CGGATTGAATTAGAATAACTTGGAAAAGTTGGAATTCTCGGGTGCCAAGGA | 33461 | 0.12367122090375902 | RNA PCR Primer, Index 1 (100% over 22bp) |
| TGAGAACTGAATTCCATAGATGGTTGGAATTCTCGGGTGCCAAGGAACTCC | 33181 | 0.1226363462182131 | RNA PCR Primer, Index 1 (100% over 27bp) |
| TAATACTGCCTGGTAATGATGATGGAATTCTCGGGTGCCAAGGAACTCCAG | 32915 | 0.12165321526694446 | RNA PCR Primer, Index 1 (100% over 29bp) |
| CAGGTGAGTAGAGCCGTTCGTGATGGAATTCTCGGGTGCCAAGGAACTCCA | 32460 | 0.1199715439029323 | RNA PCR Primer, Index 1 (100% over 28bp) |
| TTGGCAGGTGAGTAGAGCCGTTCGTGACATGGAATTCTCGGGTGCCAAGGA | 32155 | 0.1188442696918912 | RNA PCR Primer, Index 1 (100% over 22bp) |
| CTCCGGGGATGCGTGCATTTATCAGATCTGGAATTCTCGGGTGCCAAGGAA | 31372 | 0.1159503165533824 | RNA PCR Primer, Index 1 (100% over 23bp) |
| CTTAATGCCGAGAACTGATGACGATCCTTGGAATTCTCGGGTGCCAAGGAA | 31178 | 0.11523329623553984 | RNA PCR Primer, Index 1 (100% over 23bp) |
| TTAATGCCGAGAACTGATGACGATCCTTGGAATTCTCGGGTGCCAAGGAAC | 31096 | 0.11493022579191568 | RNA PCR Primer, Index 1 (100% over 24bp) |
| TTTAAGTTGAACAGATTGGGAAGTCTTGGAATTCTCGGGTGCCAAGGAACT | 31019 | 0.11464563525339054 | RNA PCR Primer, Index 1 (100% over 25bp) |
| CAAGGCCGAGAACTGATGACGAGTTATGGAATTCTCGGGTGCCAAGGAACT | 30608 | 0.1131265870542499 | RNA PCR Primer, Index 1 (100% over 25bp) |
| TGAGGTAGTAGGTTGTATAGTTGGAATTCTCGGGTGCCAAGGAACTCCAGT | 30540 | 0.11287526034490303 | RNA PCR Primer, Index 1 (100% over 30bp) |
| AGACTGAGATACGAGACGAGCCTGGAATTCTCGGGTGCCAAGGAACTCCAG | 30241 | 0.11177016201998077 | RNA PCR Primer, Index 1 (100% over 29bp) |
| CGAGAAGACGATCAAACTTGATGGAATTCTCGGGTGCCAAGGAACTCCAGT | 30087 | 0.1112009809429305 | RNA PCR Primer, Index 1 (100% over 30bp) |
| TTTGGCAGGTGAGTAGAGCCGTTCGTGATGGAATTCTCGGGTGCCAAGGAA | 28850 | 0.10662905242142937 | RNA PCR Primer, Index 1 (100% over 23bp) |
| AAGGCCGAGAACTGATGACGAGTTATGGAATTCTCGGGTGCCAAGGAACTC | 28825 | 0.1065366528959342 | RNA PCR Primer, Index 1 (100% over 26bp) |
| GCCGAGAACTGATGACGAGTTATTGGAATTCTCGGGTGCCAAGGAACTCCA | 28811 | 0.10648490916165688 | RNA PCR Primer, Index 1 (100% over 28bp) |
| GCATTGGTGGTTCAGTGGTAGAATTCTCGCCTTGGAATTCTCGGGTGCCAA | 27430 | 0.10138075937330354 | No Hit |

## Adapter Content

Produced by FastQC (version 0.11.9)
