# Supplementary material for: High Throughput Sequencing of MicroRNA in Rainbow Trout Plasma, Mucus, and Surrounding Water Following Acute Stress
Source: Front Physiol. 2021 Jan 13;11:588313. doi: 10.3389/fphys.2020.588313 (PMC7838646; doi:10.3389/fphys.2020.588313)
Supplement: Supplementary file 2 [file Data_Sheet_1.ZIP › Supplemental Quality Control/FastQC_raw_files/water_stressed_3_fastqc_raw.html]

SV18263\_0018\_S30\_R1\_001.fastq FastQC Report 

FastQC Report

Thu 7 May 2020  
SV18263\_0018\_S30\_R1\_001.fastq

## Summary

- Basic Statistics
- Per base sequence quality
- Per tile sequence quality
- Per sequence quality scores
- Per base sequence content
- Per sequence GC content
- Per base N content
- Sequence Length Distribution
- Sequence Duplication Levels
- Overrepresented sequences
- Adapter Content

## Basic Statistics

| Measure | Value |
| --- | --- |
| Filename | SV18263\_0018\_S30\_R1\_001.fastq |
| File type | Conventional base calls |
| Encoding | Sanger / Illumina 1.9 |
| Total Sequences | 29422971 |
| Sequences flagged as poor quality | 0 |
| Sequence length | 51 |
| %GC | 51 |

## Per base sequence quality

## Per tile sequence quality

## Per sequence quality scores

## Per base sequence content

## Per sequence GC content

## Per base N content

## Sequence Length Distribution

## Sequence Duplication Levels

## Overrepresented sequences

| Sequence | Count | Percentage | Possible Source |
| --- | --- | --- | --- |
| TGAGAACTGAATTCCATAGATGGTGGAATTCTCGGGTGCCAAGGAACTCCA | 1234054 | 4.194185556584343 | RNA PCR Primer, Index 1 (100% over 28bp) |
| TACCCTGTAGAACCGAATTTGTTGGAATTCTCGGGTGCCAAGGAACTCCAG | 429581 | 1.4600191122779544 | RNA PCR Primer, Index 1 (100% over 29bp) |
| AACCCGTAGATCCGAACTTGTTGGAATTCTCGGGTGCCAAGGAACTCCAGT | 273990 | 0.9312111954975586 | RNA PCR Primer, Index 1 (100% over 30bp) |
| CCGAGAAGACGATCAAACTTGATGGAATTCTCGGGTGCCAAGGAACTCCAG | 211442 | 0.7186289922931305 | RNA PCR Primer, Index 1 (100% over 29bp) |
| GAATTAGTGGAAGGCTCTGGAAAGTGCTGGAATTCTCGGGTGCCAAGGAAC | 201804 | 0.6858722730617517 | RNA PCR Primer, Index 1 (100% over 24bp) |
| GCCGAGAAGACGATCAAACTTGATGGAATTCTCGGGTGCCAAGGAACTCCA | 200219 | 0.6804853255641655 | RNA PCR Primer, Index 1 (100% over 28bp) |
| GGAATACCAGGTGCTGTAAGCTTTGGAATTCTCGGGTGCCAAGGAACTCCA | 199461 | 0.6779091071394524 | RNA PCR Primer, Index 1 (100% over 28bp) |
| CTAAGACTGAGATACGAGACGAGCCTGGAATTCTCGGGTGCCAAGGAACTC | 184268 | 0.6262725813786786 | RNA PCR Primer, Index 1 (100% over 26bp) |
| TACCGAGATCTGATAGCAAGCTTGGAATTCTCGGGTGCCAAGGAACTCCAG | 178354 | 0.606172639737843 | RNA PCR Primer, Index 1 (100% over 29bp) |
| TAGCTTATCAGACTGGTGTTGGTGGAATTCTCGGGTGCCAAGGAACTCCAG | 170699 | 0.580155552612277 | RNA PCR Primer, Index 1 (100% over 29bp) |
| GAGAATAGTGGAAGGCTCTGGAAAGTGCTGGAATTCTCGGGTGCCAAGGAA | 145773 | 0.49543943064077384 | RNA PCR Primer, Index 1 (100% over 23bp) |
| AGAATAGTGGAAGGCTCTGGAAAGTGCTGGAATTCTCGGGTGCCAAGGAAC | 142484 | 0.4842610897451518 | RNA PCR Primer, Index 1 (100% over 24bp) |
| GCACCGAAGCTGTGGACTTGCTGGAATTCTCGGGTGCCAAGGAACTCCAGT | 141156 | 0.4797476094443352 | RNA PCR Primer, Index 1 (100% over 30bp) |
| TCAAGGCCGAGAACTGATGACGAGTTTGGAATTCTCGGGTGCCAAGGAACT | 130138 | 0.44230067724975836 | RNA PCR Primer, Index 1 (100% over 25bp) |
| TCAAGGCCGAGAACTGATGACGAGTTATTGGAATTCTCGGGTGCCAAGGAA | 120361 | 0.409071538017014 | RNA PCR Primer, Index 1 (100% over 23bp) |
| ATCAAGGCCGAGAACTGATGACGAGTTTGGAATTCTCGGGTGCCAAGGAAC | 114908 | 0.3905383994022901 | RNA PCR Primer, Index 1 (100% over 24bp) |
| TAACGGAACCCATAATGCAGCTGTGGAATTCTCGGGTGCCAAGGAACTCCA | 113334 | 0.38518883766020773 | RNA PCR Primer, Index 1 (100% over 28bp) |
| CTTTTGGCAGGTGAGTAGAGCCGTTCGTGACATGGAATTCTCGGGTGCCAA | 110904 | 0.3769299843989242 | No Hit |
| AACCCGTAGATCCGAACTTGTGTGGAATTCTCGGGTGCCAAGGAACTCCAG | 108803 | 0.36978930509770747 | RNA PCR Primer, Index 1 (100% over 29bp) |
| CCGAGAAGACGATCAAACTTGGAATTCTCGGGTGCCAAGGAACTCCAGTCA | 107919 | 0.3667848498372241 | RNA PCR Primer, Index 1 (100% over 32bp) |
| TCAAGGCCGAGAACTGATGACGAGTTATGGAATTCTCGGGTGCCAAGGAAC | 106149 | 0.36076914190616577 | RNA PCR Primer, Index 1 (100% over 24bp) |
| GAGATTAGCGGAACGCTCTGGAAAGTGCTGGAATTCTCGGGTGCCAAGGAA | 100874 | 0.3428409727895935 | RNA PCR Primer, Index 1 (100% over 23bp) |
| TGGACGGAGAACTGATAAGGTGGAATTCTCGGGTGCCAAGGAACTCCAGTC | 99707 | 0.33887468400115 | RNA PCR Primer, Index 1 (100% over 31bp) |
| AAGGCCGAGAACTGATGACGAGTTTGGAATTCTCGGGTGCCAAGGAACTCC | 98663 | 0.3353264359333393 | RNA PCR Primer, Index 1 (100% over 27bp) |
| TCTTTTGGCAGGTGAGTAGAGCCGTTCGTGACTGGAATTCTCGGGTGCCAA | 96805 | 0.3290116419582509 | No Hit |
| AGGCCGAGAACTGATGACGAGTTTGGAATTCTCGGGTGCCAAGGAACTCCA | 96016 | 0.32633006367711814 | RNA PCR Primer, Index 1 (100% over 28bp) |
| ATACCGAGATCTGATAGCAAGCTTGGAATTCTCGGGTGCCAAGGAACTCCA | 90399 | 0.3072395374348838 | RNA PCR Primer, Index 1 (100% over 28bp) |
| ATCAAGGCCGAGAACTGATGACGAGTTATGGAATTCTCGGGTGCCAAGGAA | 89858 | 0.3054008380051083 | RNA PCR Primer, Index 1 (100% over 23bp) |
| ATTTGGAATTGTACAGTCAAGGTGTTGGAATTCTCGGGTGCCAAGGAACTC | 88402 | 0.3004523234584298 | RNA PCR Primer, Index 1 (100% over 26bp) |
| AGGTGAGTAGAGCCGTTCGTGACTGGAATTCTCGGGTGCCAAGGAACTCCA | 88131 | 0.2995312743910192 | RNA PCR Primer, Index 1 (100% over 28bp) |
| AGACTGAGATACGAGACGAGCCTGGAATTCTCGGGTGCCAAGGAACTCCAG | 87581 | 0.29766198661583154 | RNA PCR Primer, Index 1 (100% over 29bp) |
| TCTTTTGGCAGGTGAGTAGAGCCGTTCGTGATGGAATTCTCGGGTGCCAAG | 86817 | 0.29506537596084365 | No Hit |
| ATCAAGGCCGAGAACTGATGACGAGTTATTGGAATTCTCGGGTGCCAAGGA | 83053 | 0.2822726501684687 | RNA PCR Primer, Index 1 (100% over 22bp) |
| AGGTGAGTAGAGCCGTTCGTGACATGGAATTCTCGGGTGCCAAGGAACTCC | 80924 | 0.2750368071259697 | RNA PCR Primer, Index 1 (100% over 27bp) |
| CAGGTGAGTAGAGCCGTTCGTGACATGGAATTCTCGGGTGCCAAGGAACTC | 79394 | 0.2698367884059023 | RNA PCR Primer, Index 1 (100% over 26bp) |
| TTTTGGCAGGTGAGTAGAGCCGTTCGTGATGGAATTCTCGGGTGCCAAGGA | 79145 | 0.2689905108494992 | RNA PCR Primer, Index 1 (100% over 22bp) |
| AGAATTAGTGGAAGGCTCTGGAAAGTGCTGGAATTCTCGGGTGCCAAGGAA | 74687 | 0.25383908375534203 | RNA PCR Primer, Index 1 (100% over 23bp) |
| TAGCTTATCAGACTGGTGTTGGCTGGAATTCTCGGGTGCCAAGGAACTCCA | 74336 | 0.25264613828426774 | RNA PCR Primer, Index 1 (100% over 28bp) |
| TTCTATACCGAGATCTGATAGCAAGCTTGGAATTCTCGGGTGCCAAGGAAC | 73678 | 0.2504097903641342 | RNA PCR Primer, Index 1 (100% over 24bp) |
| CAAGGCCGAGAACTGATGACGAGTTTGGAATTCTCGGGTGCCAAGGAACTC | 73638 | 0.25027384216230236 | RNA PCR Primer, Index 1 (100% over 26bp) |
| TAACACTGTCTGGTAACGATGTGGAATTCTCGGGTGCCAAGGAACTCCAGT | 72853 | 0.24760585870135277 | RNA PCR Primer, Index 1 (100% over 30bp) |
| GAATACCAGGTGCTGTAAGCTTTGGAATTCTCGGGTGCCAAGGAACTCCAG | 72085 | 0.24499565322618166 | RNA PCR Primer, Index 1 (100% over 29bp) |
| GCCGAGAACTGATGACGAGTTTGGAATTCTCGGGTGCCAAGGAACTCCAGT | 66040 | 0.22445048122434677 | RNA PCR Primer, Index 1 (100% over 30bp) |
| TGAGAACTGAATTCCATAGATGTGGAATTCTCGGGTGCCAAGGAACTCCAG | 65806 | 0.2236551842436306 | RNA PCR Primer, Index 1 (100% over 29bp) |
| TGAGAACTGAATTCCATAGATGGTTGGAATTCTCGGGTGCCAAGGAACTCC | 61937 | 0.21050559442144712 | RNA PCR Primer, Index 1 (100% over 27bp) |
| CAAGGCCGAGAACTGATGACGAGTTATTGGAATTCTCGGGTGCCAAGGAAC | 59577 | 0.2024846505133693 | RNA PCR Primer, Index 1 (100% over 24bp) |
| AGATTAGCGGAACGCTCTGGAAAGTGCTGGAATTCTCGGGTGCCAAGGAAC | 58944 | 0.2003332702193806 | RNA PCR Primer, Index 1 (100% over 24bp) |
| CCTAAGACTGAGATACGAGACGAGCCTGGAATTCTCGGGTGCCAAGGAACT | 58185 | 0.1977536530896217 | RNA PCR Primer, Index 1 (100% over 25bp) |
| TAAGCCGAGCAATACTAATGAATCTGGAATTCTCGGGTGCCAAGGAACTCC | 56701 | 0.1927099748016609 | RNA PCR Primer, Index 1 (100% over 27bp) |
| GGTGAGTAGAGCCGTTCGTGACATGGAATTCTCGGGTGCCAAGGAACTCCA | 55727 | 0.18939963608705593 | RNA PCR Primer, Index 1 (100% over 28bp) |
| AATTAGTGGAAGGCTCTGGAAAGTGCTGGAATTCTCGGGTGCCAAGGAACT | 54364 | 0.1847672011096364 | RNA PCR Primer, Index 1 (100% over 25bp) |
| GCATTGGTGGTTCAGTGGTAGAATTCTCGCCTTGGAATTCTCGGGTGCCAA | 53410 | 0.18152483649594733 | No Hit |
| TTGGCAGGTGAGTAGAGCCGTTCGTGATGGAATTCTCGGGTGCCAAGGAAC | 52211 | 0.17744978914603832 | RNA PCR Primer, Index 1 (100% over 24bp) |
| GCACCGAAGCTGTGGACTTGCATGGAATTCTCGGGTGCCAAGGAACTCCAG | 50329 | 0.17105342624985084 | RNA PCR Primer, Index 1 (100% over 29bp) |
| TTTTATACCGAGATCTGATAGCAAGCTTGGAATTCTCGGGTGCCAAGGAAC | 49390 | 0.1678620422118487 | RNA PCR Primer, Index 1 (100% over 24bp) |
| CAAGGCCGAGAACTGATGACGAGTTATGGAATTCTCGGGTGCCAAGGAACT | 48919 | 0.16626125213527893 | RNA PCR Primer, Index 1 (100% over 25bp) |
| CGAGAAGACGATCAAACTTGATGGAATTCTCGGGTGCCAAGGAACTCCAGT | 48080 | 0.16340973860185637 | RNA PCR Primer, Index 1 (100% over 30bp) |
| TCTTTTGGCAGGTGAGTAGAGCCGTTCGTGACATGGAATTCTCGGGTGCCA | 48039 | 0.16327039169497873 | No Hit |
| TTTTGGCAGGTGAGTAGAGCCGTTCGTGACTGGAATTCTCGGGTGCCAAGG | 47490 | 0.1614045026248369 | Illumina Small RNA Adapter 2 (100% over 21bp) |
| TGAAATGTTTAGGACCACTCGTGGAATTCTCGGGTGCCAAGGAACTCCAGT | 47025 | 0.15982410477854192 | RNA PCR Primer, Index 1 (100% over 30bp) |
| CTTTTGGCAGGTGAGTAGAGCCGTTCGTGATGGAATTCTCGGGTGCCAAGG | 46457 | 0.15789364031252995 | Illumina Small RNA Adapter 2 (100% over 21bp) |
| AAGGCCGAGAACTGATGACGAGTTATGGAATTCTCGGGTGCCAAGGAACTC | 45801 | 0.155664089802488 | RNA PCR Primer, Index 1 (100% over 26bp) |
| ATTAGTGGAAGGCTCTGGAAAGTGCTGGAATTCTCGGGTGCCAAGGAACTC | 45410 | 0.15433519612958188 | RNA PCR Primer, Index 1 (100% over 26bp) |
| TTTTGGCAGGTGAGTAGAGCCGTTCGTGACATGGAATTCTCGGGTGCCAAG | 44635 | 0.15170119971909024 | No Hit |
| AGGCCGAGAACTGATGACGAGTTATGGAATTCTCGGGTGCCAAGGAACTCC | 44409 | 0.15093309237874042 | RNA PCR Primer, Index 1 (100% over 27bp) |
| AAGACTGAGATACGAGACGAGCCTGGAATTCTCGGGTGCCAAGGAACTCCA | 43737 | 0.1486491625879657 | RNA PCR Primer, Index 1 (100% over 28bp) |
| TAACGGAACCCATAAAGCAGCTGTGGAATTCTCGGGTGCCAAGGAACTCCA | 43646 | 0.14833988042879828 | RNA PCR Primer, Index 1 (100% over 28bp) |
| GGAATACCAGGTGCTGTAAGCTTGGAATTCTCGGGTGCCAAGGAACTCCAG | 40196 | 0.1366143480208032 | RNA PCR Primer, Index 1 (100% over 29bp) |
| CGAGAAGACGATCAAACTTGACTATTGGAATTCTCGGGTGCCAAGGAACTC | 39930 | 0.13571029247862154 | RNA PCR Primer, Index 1 (100% over 26bp) |
| TAAGACTGAGATACGAGACGAGCCTGGAATTCTCGGGTGCCAAGGAACTCC | 37129 | 0.12619051964534786 | RNA PCR Primer, Index 1 (100% over 27bp) |
| TTGGCAGGTGAGTAGAGCCGTTCGTGACATGGAATTCTCGGGTGCCAAGGA | 35246 | 0.11979075804411458 | RNA PCR Primer, Index 1 (100% over 22bp) |
| AAGCCGAGCAATACTAATGAATCTGGAATTCTCGGGTGCCAAGGAACTCCA | 35225 | 0.11971938523815287 | RNA PCR Primer, Index 1 (100% over 28bp) |
| AACCCGTAGATCCGAACTTGTGTTGGAATTCTCGGGTGCCAAGGAACTCCA | 34647 | 0.11775493372168297 | RNA PCR Primer, Index 1 (100% over 28bp) |
| AAGGCCGAGAACTGATGACGAGTTATTGGAATTCTCGGGTGCCAAGGAACT | 34406 | 0.1169358458056462 | RNA PCR Primer, Index 1 (100% over 25bp) |
| AAGCCGAGTAATACTAATGAATCTGGAATTCTCGGGTGCCAAGGAACTCCA | 34376 | 0.11683388465427234 | RNA PCR Primer, Index 1 (100% over 28bp) |
| GCCGAGAACTGATGACGATCCTGGAATTCTCGGGTGCCAAGGAACTCCAGT | 34316 | 0.11662996235152459 | RNA PCR Primer, Index 1 (100% over 30bp) |
| TACCGAGATCTGATAGCAATGGAATTCTCGGGTGCCAAGGAACTCCAGTCA | 33625 | 0.11428145716487978 | RNA PCR Primer, Index 1 (100% over 32bp) |
| TGAGGTAGTAGGTTGTATAGTTTGGAATTCTCGGGTGCCAAGGAACTCCAG | 33348 | 0.11334001586719437 | RNA PCR Primer, Index 1 (100% over 29bp) |
| CTTTTGGCAGGTGAGTAGAGCCGTTCGTGACTGGAATTCTCGGGTGCCAAG | 32723 | 0.11121582521357208 | No Hit |
| GCATTGGTGGTTCAGTGGTAGAATTCTCGCCTGGAATTCTCGGGTGCCAAG | 32549 | 0.11062445053560363 | No Hit |
| TAATACTGCCTGGTAATGATGATGGAATTCTCGGGTGCCAAGGAACTCCAG | 32348 | 0.10994131082139869 | RNA PCR Primer, Index 1 (100% over 29bp) |
| CAGGTGAGTAGAGCCGTTCGTGACTGGAATTCTCGGGTGCCAAGGAACTCC | 32265 | 0.10965921830259764 | RNA PCR Primer, Index 1 (100% over 27bp) |
| GAGGTGTAGAATAAGTGGGAGGCCCTGGAATTCTCGGGTGCCAAGGAACTC | 31972 | 0.10866339772417952 | RNA PCR Primer, Index 1 (100% over 26bp) |
| AGGCCGAGAACTGATGACGAGTTATTGGAATTCTCGGGTGCCAAGGAACTC | 31786 | 0.10803123858566152 | RNA PCR Primer, Index 1 (100% over 26bp) |
| CAGGTGAGTAGAGCCGTTCGTGATGGAATTCTCGGGTGCCAAGGAACTCCA | 31581 | 0.10733450405127341 | RNA PCR Primer, Index 1 (100% over 28bp) |
| TTTCATACCGAGATCTGATAGCAAGCTTGGAATTCTCGGGTGCCAAGGAAC | 31361 | 0.10658678894119836 | RNA PCR Primer, Index 1 (100% over 24bp) |
| CGAGAAGACGATCAAACTTGACTGGAATTCTCGGGTGCCAAGGAACTCCAG | 31071 | 0.10560116447791762 | RNA PCR Primer, Index 1 (100% over 29bp) |
| ACCGAGATCTGATAGCAAGCTTGGAATTCTCGGGTGCCAAGGAACTCCAGT | 30734 | 0.10445580087748446 | RNA PCR Primer, Index 1 (100% over 30bp) |
| AACCCGTAGATCCGAACTTGTGATGGAATTCTCGGGTGCCAAGGAACTCCA | 30323 | 0.10305893310366245 | RNA PCR Primer, Index 1 (100% over 28bp) |
| TTCAAGTAATCCAGGATAGGCTTGGAATTCTCGGGTGCCAAGGAACTCCAG | 30138 | 0.10243017267019025 | RNA PCR Primer, Index 1 (100% over 29bp) |
| TTTGGCAGGTGAGTAGAGCCGTTCGTGATGGAATTCTCGGGTGCCAAGGAA | 29525 | 0.10034676647711749 | RNA PCR Primer, Index 1 (100% over 23bp) |
| TACCGAGATCTGATAGCAAGCTGGAATTCTCGGGTGCCAAGGAACTCCAGT | 29483 | 0.10020402086519406 | RNA PCR Primer, Index 1 (100% over 30bp) |
| TTAATGCCGAGAACTGATGACGATCCTGGAATTCTCGGGTGCCAAGGAACT | 29477 | 0.1001836286349193 | RNA PCR Primer, Index 1 (100% over 25bp) |
| ATTCTATACCGAGATCTGATAGCAAGCTTGGAATTCTCGGGTGCCAAGGAA | 29474 | 0.10017343251978189 | RNA PCR Primer, Index 1 (100% over 23bp) |

## Adapter Content

Produced by FastQC (version 0.11.9)
