# Supplementary material for: High Throughput Sequencing of MicroRNA in Rainbow Trout Plasma, Mucus, and Surrounding Water Following Acute Stress
Source: Front Physiol. 2021 Jan 13;11:588313. doi: 10.3389/fphys.2020.588313 (PMC7838646; doi:10.3389/fphys.2020.588313)
Supplement: Supplementary file 2 [file Data_Sheet_1.ZIP › Supplemental Quality Control/Supplemental Quality Control Captions.docx]

**Supplemental Quality Control Caption**

FastQC reports for raw (FastQC_raw_files) and processed (FastQC_processed files) reads. Each report is labelled by sample type (plasma, mucus, or water), treatment (control or stressed), rep number (1-3), analysis performed (fastqc), and whether it was run on raw or processed reads. These were analyzed using FastQC 0.11.9 and the configuration files for miARma-Seq found in the supplemental methods.
