# Supplementary material for: High Throughput Sequencing of MicroRNA in Rainbow Trout Plasma, Mucus, and Surrounding Water Following Acute Stress
Source: Front Physiol. 2021 Jan 13;11:588313. doi: 10.3389/fphys.2020.588313 (PMC7838646; doi:10.3389/fphys.2020.588313)
Supplement: Supplementary file 3 [file Data_Sheet_2.ZIP › Supplemental Methods/Supplemental Methods Captions.docx]

**Supplemental Methods Captions**

precutadapt_fastqc_configfile.ini – Configuration file used to conduct quality control checks on raw reads. This analysis uses FastQC through the miARma-Seq pipeline.

cutadapt2.10adapter_removal_and_trimming.sh – Script used to remove the Illumina small RNA-Seq 3’ adapter and to trim bases with a quality score less than 28 using cutadapt.

cutadapt2.10sizetrim.sh – Script used to remove reads that are less than 18 bases or greater than 35 bases in length using cutadapt.

postcutadapt_fastqc_configfile.ini – Configuration file used to conduct quality control checks on trimmed and filtered reads. This analysis uses FastQC through the miARma-Seq pipeline.

miARmaseq_denovo_ssa_configfile.ini – Configuration file used to conduct alignment, annotation, and read counting of trimmed and filtered reads. This analysis uses Bowtie1 and mirDeep2 through the miARma-Seq pipeline.

bowtie1_version.txt – Text file containing details pertaining to the version and build of Bowtie1 used.

script_deseq2.R – R script used to conduct DESeq2 analysis of differentially expressed microRNA. The script uses the read count file that miARma-Seq produces (miARmaseq_denovo_ssa_configfile.ini) and identifies the differentially expressed microRNA between treatments in each sample type (plasma, mucus, water).

session_info_DESeq2.txt – The versions of R, DESeq2, and the required packages used during DESeq2 analysis.

script_edgeR.R – R script used to conduct edgeR analysis of differentially expressed microRNA. The script uses the read count file that miARma-Seq produces (miARmaseq_denovo_ssa_configfile.ini) and identifies the differentially expressed microRNA between treatments in each sample type (plasma, mucus, water).

session_info_edgeR.txt – The versions of R, edgeR, and the required packages used during DESeq2 analysis.

one-miRNA-all-3UTR.miranda_analysis.sh – The script used to determine the predicted 3’UTR (untranslated region) targets of the known significantly altered microRNA in plasma. It compares one microRNA (user input) with all the *Salmo salar* (Atlantic salmon) 3’UTRs. It also formats the results so that they can imported into Excel, filtered, and exported for downstream analysis.
